# Cranial anatomy, palaeoneurology, palaeobiology and stratigraphic age of the large-bodied ornithopod, *Muttaburrasaurus langdoni* Bartholomai and Molnar, 1981, from the mid-Cretaceous of Australia

**DOI:** 10.7717/peerj.20794

**Published:** 2026-04-09

**Authors:** Matthew C. Herne, Joseph J. Bevitt, Luke Milan, Scott A. Hocknull, Alan M. Tait, Charlotte M. Allen, Andrew C. Rozefelds, Ralph E. Molnar, Vera Weisbecker, Phil R. Bell

**Affiliations:** 1 School of Environmental & Rural Science, University of New England, Armidale, NSW, Australia; 2 Biodiversity and Geosciences, Queensland Museum, Brisbane, Queensland, Australia; 3 Australian Centre for Neutron Scattering, Australian Nuclear Science and Technology Organisation, Lucas Heights, New South Wales, Australia; 4 School of Earth, Atmosphere & Environment, Monash University, Melbourne, Victoria, Australia; 5 Central Analytical Research Facility, Research Infrastructure and School of Earth and Atmospheric Sciences, Faculty of Science, Queensland University of Technology, Brisbane, Queensland, Australia; 6 University of California Museum of Paleontology, University of California, Berkeley, Berkeley, CA, United States of America; 7 College of Science and Engineering, Flinders University of South Australia, Adelaide, South Australia, Australia

**Keywords:** Dinosaur, Ornithopod, Australia, Cretaceous, Zircon, Cranium, Osteology, Neuroanatomy, Palaeobiology, Convergence

## Abstract

The holotype of *Muttaburrasaurus langdoni* Bartholomai & Molnar, 1981, a large-bodied ornithopod from the mid-Cretaceous of Australia, consists of an almost complete skull and partial postcranium, and is among the most skeletally complete ornithopods from Gondwana. The taxon was defined by a dorsally inflated muzzle, thought to be formed by the nasals, enlarged mandibular adductor musculature and cheek teeth thought to have a uniquely *en masse* eruption pattern. The rostrum, however, was unknown. The original description of the holotype skull was superficial, which has confounded numerous attempts to resolve the phylogenetic relationships of the taxon. Recently, the holotype quarry was reworked and new craniodental materials were collected. In addition, previously undescribed materials of the holotype skull are now identified. Here, using CT imagery, we extensively revise the craniodental osteology of *Muttaburrasaurus langdoni* and describe its palaeoneurology. From detrital zircons, we date the holotype locality in the Cenomanian at 96.3 ± 8.6 Ma. The premaxillary ramus of the holotype, now discovered, has five well-developed teeth, as in early diverging ornithischians. Modified premaxillary processes exclude the nasals from the nares, convergent with lambeosaurines, and novel paired ossifications, termed prenasals, form the roof and internal septa of the muzzle. Superior airway chambers in the muzzle, descending turbinate support ridges and highly enlarged olfactory bulbs, suggest heightened olfactory acuity. As in other megaherbivorous vertebrates, wide monocular vision potentially aided predator detection and conspecific interaction, such as herding behaviour. Stereoscopic vision in the narrow binocular field potentially assisted target selection, obstacle avoidance and distance timing during locomotion. Low frequency hearing (<1 kHz) could have aided communication in open and closed habitats and under low light conditions. Proportions of the semicircular canals suggest a facultative biped. Cognition appears comparable to non-hadrosaurid iguanodontians. The narrow premaxilla is consistent with selective browsing and caniniform premaxillary dentition potentially aided access to nutritious plant food items encased in cones and possibly invertebrates. The cheek teeth erupted in a wave-like pattern, as in other ornithischians and were configured for grinding mastication. Nasal salt glands were possibly developed, suggesting a diet that included excess salt ingestion. Our findings shed new light on the behavioural and sensory palaeobiology of *Muttaburrasaurus*. We anticipate future phylogenetic analyses of *Muttaburrasaurus* will be better informed from the anatomical information provided herein.

## Introduction

**Figure 1:**
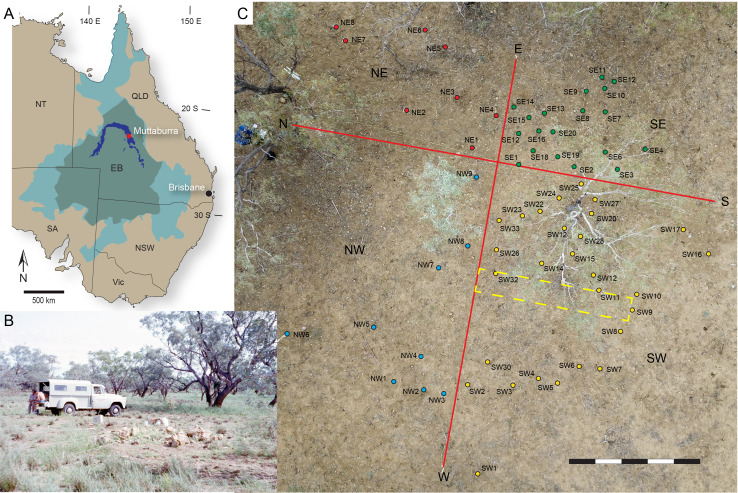
Maps and photographs of geological and locality features relevant to the collection of the *Muttaburrasaurus langdoni* holotype (QMF6140). (A) Map of eastern mainland Australia showing the position of the holotype locality (QML1794) near the Muttaburra township (red star), surface exposures of the Mackunda Formation (dark blue colour; taken from (Exon & Senior, 1976); Queensland Globe State Surface Geology: https://qldglobe.information.qld.gov.au/; Creative Commons Attribution 4.0 International (CC BY 4.0) licence), extent of the Eromanga Basin (olive green shading; taken from Exon & Senior, 1976) and mid-Cretaceous extent of the Eromanga Sea (adapted from from Dettmann et al., 1992). (B) Photograph of the holotype locality in 1963 looking east, showing scattered fossil-bearing concretions during collection (with permission Queensland Museum: photographer, A. Bartholomai). (C) Drone image and map overlay of the holotype locality (2020), showing distribution of fossil bone fragments on the vertosol surface within NE, SE, SW and NW sectors (red, green, yellow, blue dots with field numbers, respectively) divided by red lines. Drone image in C with permission: C. Rohan. Yellow dashed area in C indicates location of 50 cm deep geo-trench (2022). Abbreviations: EB, Eromanga Basin; NSW, New South Wales; NT, Northern Territory; Qld, Queensland; SA, South Australia; Vic, Victoria. Scale bar in C equals 5 m.

The holotype of the large-bodied ornithopod dinosaur, *Muttaburrasaurus langdoni* Bartholomai & Molnar, 1981, from the mid-Cretaceous of Australia, is among the most skeletally complete ornithopods from Gondwana and is the fossil emblem of the state of Queensland. The holotype (QMF6140) was discovered in 1963 near the central western Queensland township of Muttaburra (Iningai Country) by local grazier Doug Langdon (Fig. 1A). Soon after its discovery, the specimen was collected by the Queensland Museum (Fig. 1B) and named in 1981. Following collection of the holotype in 1963, the holotype locality was not reinvestigated. The *Muttaburrasaurus langdoni* holotype was found in rocks of the Mackunda Formation Vine & Day, 1965, that were laid down as shallow marine strata in the epeiric Eromanga Sea, which, during all of the Early Cretaceous and possibly into the beginning of the Late Cretaceous, covered much of central eastern Australia (Gallagher & Lambeck, 1989; Harrington et al., 2019; Matthews et al., 2011; Müller et al., 2016) (Fig. 1A). From U/Pb isotopes in detrital zircon, Tucker et al. (2016) dated the Mackunda Formation to the upper Albian (∼104–102 Ma). However, radiometric dating of the *Muttaburrasaurus langdoni* locality has not been previously undertaken.

Two additional specimens assigned to *Muttaburrasaurus* spp., were discovered in slightly older Eromanga Basin strata of the shallow marine Allaru Formation, which conformably underlies the Mackunda Formation. These specimens include a partial skull with a partial postcranial skeleton (QMF14921); known as the ‘Dunluce’ specimen) and a dentary fragment (QMF12541; known as the ‘Iona’ specimen) from another partial skeleton (Molnar, 1995; Molnar, 1996). The remaining material of this latter specimen is in a private collection and is unavailable for study. Three isolated teeth (AMF81865, QMF14420, QMF14421) were also attributed to *Muttaburrasaurus* from the Griman Creek Formation at Lightning Ridge, New South Wales, by Molnar (1996). Strata of the Griman Creek Formation were laid down in freshwater lagoon deposits bordering the Eromanga Sea during the lower to middle Cenomanian (Bell et al., 2019b).

The cranium of *Muttaburrasaurus* is characterised by an unusually inflated muzzle, a robust, transversely broad skull and an enlarged mandibular adductor chamber that suggested a particularly strong bite force (Bartholomai & Molnar, 1981; Molnar, 1995; Molnar, 1996). The inflated region of the muzzle was described as the ‘nasal bulla’, as it was thought to have been formed from the nasal bones (Bartholomai & Molnar, 1981; Molnar, 1995; Molnar, 1996). Restorations of the *Muttaburrasaurus langdoni* skull tentatively suggested that the external nasal openings (nares) formed anteroposteriorly elongate slots that extended dorsally on the nasal bulla (Bartholomai & Molnar, 1981; Molnar, 1995; Molnar, 1996). The function of the dorsally inflated muzzle of *Muttaburrasaurus* ssp. has been uncertain, although olfaction and phonation were considered possibilities (Bartholomai & Molnar, 1981; Molnar, 1996). From the appearance of the laterally exposed maxillary tooth crowns on the two *Muttaburrasaurus* skulls, Bartholomai & Molnar (1981), Molnar (1995) and Molnar (1996) suggested that cheek tooth replacement in the taxon occurred *en masse*, differing from the staggered sequential replacement pattern seen in other ornithischians. In addition, a transversely exposed broken tooth on the holotype suggested to Molnar (1995) that the cheek teeth formed a sharp, steeply angled, occlusal edge that sheared through tough plant material, as in ceratopsians. In other large ornithopods, the occlusal surfaces on the cheek teeth are more obtuse and functionally arranged for grinding mastication (Weishampel, 1984).

On the skulls of the *Muttaburrasaurus langdoni* holotype and *M*. sp. (QMF14921), the premaxillary rami and predentary that form the rostrum, were missing. Although these rostra were missing, the region preserved more posteriorly on the holotype, Molnar (1995) suggested that the beak was likely to have been narrow, as in ceratopsians, rather than broad and “iguanodont-like”. That study further pointed out that additional information on the feeding behaviour of *Muttaburrasaurus* would be more fully realised when the “snout” is discovered. Notably in Iguanodontia, the premaxillary rami are typically edentulous and bill-like, exemplified by the ‘duck-like’ bill of the hadrosaurids (Norman, 1984). In contrast to the iguanodontian premaxilla, the premaxillary rami of the early diverging (‘basal’) ornithopods, *Hypsilophodon foxii* (Galton, 1974) and *Convolosaurus marri* (Andrzejewski, Winkler & Jacobs, 2019) and early diverging non-ornithopod ornithischians, such as *Thescelosaurus neglectus* (Boyd, 2014) and *Haya griva* (Makovicky et al., 2011), have well-developed teeth and form narrow rostra. Possibly because of its large body size and taxonomic placement in Iguanodontia, at least historically, the premaxillary ramus of *Muttaburrasaurus* has been assumed to be edentulous and bill-like (Bartholomai & Molnar, 1981, fig. 2A).

Cladistic investigations of ornithopod relationships have variously recovered *Muttaburrasaurus* as an elasmarian (Bell et al., 2019a; Fonseca et al., 2024b), a styracosternan (Madzia, Jagt & Mulder, 2020), an iguanodontian sister taxon to Dryosauridae (Boyd, 2015; Herne et al., 2019), a rhabdodontid (Augustin et al., 2022; McDonald, Barrett & Chapman, 2010a), a rhabdodontomorph (Dieudonné et al., 2016; Dieudonné et al., 2021; Zanno et al., 2023) and a rhabdodontoid (Poole, 2022). Using an apomorphy-based approach, Agnolin et al (2010) considered *Muttaburrasaurus langdoni* assignable to Styracosterna. Evidently, phylogenetic analyses have invoked conflicting hypotheses on the evolutionary relationships of *Muttaburrasaurus*, and it would be fair to say that data for this taxon in these analyses has relied upon superficial understanding of the skull provided in the original descriptions, or from low-resolution replicas. Given that a significant portion of characters in ornithischian phylogenetic datasets pertain to the skull, greater anatomical understanding of the *Muttaburrasaurus* cranium is critical to resolving the phylogenetic position of the taxon, such as the contested placements of *Muttaburrasaurus* in the Laurasian-centred clade, Rhabdodontomorpha, or the Gondwanan-centred clade, Elasmaria.

Although the cranium of the *Muttaburrasaurus langdoni* holotype is almost complete, its anatomy has only been superficially described. Notably, the original publications on *Muttaburrasaurus* (Bartholomai & Molnar, 1981; Molnar, 1995; Molnar, 1996) lacked the benefit of computed tomography (CT) and beyond these studies, no detailed anatomical revision of the *Muttaburrasaurus langdoni* holotype cranium, or the ‘Dunluce’ cranium (QMF14921), has been undertaken. This lack of revision is not without reason, as without the aid of CT, deciphering the margins between the adjoining bones and between the bones and matrix on these crania is extremely difficult. Only the outer surfaces of these skulls can be directly viewed and cranial structures imbedded in the carbonate matrix, such as the palatal regions, internal regions of the dorsally inflated muzzle, neurocrania, neural endocasts and many surfaces of the dentition, are inaccessible. The absence of detailed anatomical understanding of the *Muttaburrasaurus* cranium has inhibited accurate cranial comparisons with other ornithischians and the acquisition of much needed cranial phylogenetic data to better analyse the ancestral affiliations of the taxon. As work on other dinosaurs has shown, the behaviour and palaeoecology of *Muttaburrasaurus* will be better informed from an understanding of the neural endocast (regions of the brain and inner ear canals), greater understanding of the dentition and understanding of the internal anatomy of the muzzle (*e.g.*, Caspar et al., 2024; Walsh et al., 2009; Weishampel, 1984; Witmer & Ridgely, 2010; Zelenitsky et al., 2011).

Following recent field work, new craniodental materials of the *Muttaburrasaurus langdoni* holotype were discovered and collected (Fig. 1C). In addition, craniodental remains of the holotype collected in 1963 are now identified from within the Queensland Museum collections that were not previously published. With the aid of CT, we extensively revise the cranial osteology of the *Muttaburrasaurus langdoni* holotype and, for the first time, describe the palaeoneurology of the taxon from the neural endocast. With improved anatomical understanding, this work will provide new insight on the sensory, feeding and locomotory behaviour of *Muttaburrasaurus langdoni* that drove the palaeoecology of this enigmatic taxon. As the stratigraphic age of the *Muttaburrasaurus langdoni* holotype is unknown, we undertake radiometric dating from detrital zircons retrieved from the holotype locality. We anticipate that the anatomical and geochronological findings of this investigation will reveal new information about the evolution of ornithopod dinosaurs across Gondwana and help inform future time-calibrated analyses of ornithischian phylogenetic relationships that include *Muttaburrasaurus*.

## Geology

### Locality and horizon

The *Muttaburrasaurus langdoni* holotype (QMF6140) was discovered in weathered and fragmented carbonate concretions exposed and scattered on the surface of a smectitic-clay rich soil, known colloquially as ‘black soil’, in a lightly wooded agricultural paddock on the eastern side of the Thomson River at Rosebery Downs Station, ∼7.0 km SE of the Muttaburra township (Bartholomai & Molnar, 1981) (Fig. 1B). The locality (Queensland Museum fossil locality, QML1794; coordinates withheld) is in the Mackunda Formation of the Rolling Downs Group (Manuka Subgroup) of the Eromanga Basin (Gray, McKillop & McKellar, 2002; Vine & Day, 1965; BMR GB, 1970) (Fig. 1A). The Mackunda Formation has a total vertical thickness of ∼305 m (based on cross-sections in BMR GB (1970)) and crops-out along a U-shaped region of scattered surface exposures across central Queensland (Fig. 1A). It conformably overlies the Allaru Formation and conformably underlies the Winton Formation. As inferred from the map section (BMR GB, 1970), QML1794 is positioned in the lower half of the Mackunda Formation. Based on U/Pb dating from detrital zircon from a bore sampling the top of the Mackunda Formation (Geological Survey of Queensland bore, Longreach 1–1B, ∼92 km south/southwest of QML1794), Tucker et al. (2016) assessed the youngest depositional age of the Mackunda Formation in the range of 102.5 Ma. Interestingly, from a bore sampling the middle of the Mackunda Formation (Geological Survey of Queensland bore, Maneroo 331, ∼130 km southwest of QML1794), Tucker et al. (2016) assessed an oldest depositional age of ∼130.5 Ma in the Hauterivian (notably, not “Barremian” as reported). From U/Pb samples collected in this study, in the vicinity of the holotype locality of *Muttaburrasaurus langdoni*, a younger depositional age of the Mackunda Formation is estimated herein (see “Geochronology” below).

The Mackunda Formation consists of interbedded labile volcanogenic sandstone, siltstone, mudrock, limestone coquina in shelly layers, intraformational mudclast conglomerate, and cone-in-cone limestone, laid down in shallow marine to paralic environments during the final regressionary phase of the epeiric Eromanga Sea (Vine & Day, 1965; Cook, McKeller & Draper, 2013). Volcanogenic sediment was sourced from the Whitsundays Volcanic Province that extended along the eastern margin of the Australian Plate during the Early Cretaceous and into the beginning of the Late Cretaceous (Bryan et al., 1997; Bryan et al., 2000). The Eromanga Sea covered much of central eastern Australia during the Early Cretaceous (Cook, McKeller & Draper, 2013; Exon & Senior, 1976; Gray, McKillop & McKellar, 2002) caused by a combination of tectonic subsidence of the Australian plate as it moved eastwards over the older subducting east Gondwanan slab (Matthews et al., 2011; Müller et al., 2016) and generally higher global sea levels (up to 240 m) during the Cretaceous (Haq, Hardenbol & Vail, 1987; Miller et al., 2005). The final retreat of the Eromanga Sea occurred during deposition of the Winton Formation in the Turonian. Weathered concretions in the vicinity of the holotype locality host locally abundant molluscs, gastropods and belemnites and rare small ammonites, ophiuroids, fish vertebrae and shark teeth (Fig. S1A), consistent with shallow marine deposition generally reported in the Mackunda Formation. Bedrock is not exposed directly at QML1794 but appears in low ridges at the surface close to the site indicating that the weathered surface of the bedrock is undulating and close to the soil surface. Bedrock crops-out in the banks of the Thomson River, ∼80 m west of QML1794 and occurs as fractured, calcite-cemented, fossil-bearing sandstone concretions in the region surrounding QML1794. Although these fossil-bearing concretions are not stratigraphically *in-situ*, their concentration at the locality suggests they were left isolated roughly in place after the erosion of uncemented bedrock. This mode of *in-situ* concretion preservation is evident from an outcrop of fossil-barren, large bedrock concretions in the riverbank and channel bed ∼275 m from QML1794 (Figs. S1B, S1C). These concretions show hummocky/swaley cross-stratification with low-angle erosion surfaces, characteristic of shallow marine tempestite deposition (Myrow & Southard, 1996) and are typical of exposures across the Mackunda Formation.

The *Muttaburrasaurus langdoni* holotype was hosted by a greyish-green, feldspathic, carbonate-cemented mudrock. The shelly, cemented sandstone layers exposed in concretions in the region surrounding QML1794 (Fig. S1A) contain mixed sizes of marine gastropod and bivalve shells (including layered fragments of large *Inoceramus*) and other bioclasts (wood fragments, bones, teeth, belemnites) potentially derived from storm winnowing of the seafloor, both within the area of deposition and areas closer to shore, with subsequent offshore transport to the depositional area. The mudrock hosting the holotype potentially represents the finest fraction of a tempestite preserved within a large storm-induced scour hollow that formed around the dinosaur carcass. However, ongoing sedimentological investigation aims to address the taphonomy of the *Muttaburrasaurus langdoni* holotype more fully.

### Field collection of the holotype

The original collection of the *Muttaburrasaurus langdoni* holotype in 1963 is recorded by a handful of surviving historic photographic images (*e.g.*, Fig. 1B). These images, such as in Fig. 1B, show multiple fragmented blocks of the holotype skeleton laying on the soil surface. A grid was clearly marked, but a quarry map was not published. During the original collection, subsurface excavation was not undertaken (T. Dahms, pers. comms, 2020). In 2020, our team relocated the holotype locality. The soil horizon was excavated to a depth of ∼30 cm over a rectangular area of ∼20 m by ∼16 m (Fig. 1C). The excavated soil was dry sieved through a one cm screen to capture small bone fragments. During the surface collection, the field positions of the fossils were recorded and mapped within four sectors: northeast, northwest, southeast and southwest. A drone image captures the view of site and the grid layout (Fig. 1C). The materials collected were recorded according to the sector where found. Approximately 1,300 bone fragments were recovered on the surface and from the excavated soil horizon. However, most of the fragments were small (<five cm). The largest fragment was the proximal end of the right humerus. The descriptions of the newly recovered postcranial materials will be presented elsewhere. As can be seen in Fig. 1C, the fossil elements were concentrated in the southeast and southwest sectors, which, from examination of the early site photographs, appear to correlate with the concentration of the fossil blocks of the holotype originally collected. The newly collected craniodental fragments were found mainly in the southeast sector.

Excavations of vertebrate fossils in the Winton Formation have shown that fossil bone fragments found on the soil surface can alert to the presence of bones remaining *in-situ* in the bedrock below the soil layer. Solid objects (such as fossil bone fragments) can be brought to the surface by a process of self-mulching, caused by shrinking and swelling of the smectite-rich soil, termed vertosol (*e.g.*, Grant & Blackmore, 1991), which constitutes the ‘black soil’ plains that cover large areas of inland Queensland. This process naturally exhumes fossil elements into the vertosol from the bedrock below (Hocknull et al., 2009; Hocknull et al., 2021; Poropat et al., 2017). With this knowledge, we attempted to determine if the remains of the *Muttaburrasaurus langdoni* holotype had been exhumed from bedrock below the vertosol, and if more of the skeleton remained *in-situ*. During excavation, few bone fragments were found below 20–30 cm and, due to time limitations, we were unable to hand excavate through the hardened clay to a level more than 40 cm over the site. At this level, bedrock and/or concretions were not encountered. However, at a depth of 50 cm in a hand-dug test trench of 0.8 m × 6 m (Fig. 1C), a whitish sand was encountered as well as the top of a large, indurated concretion. Examination of the upper surface of the concretion gave no indication of fossil material. However, a small fragment of fossil bone, consistent with the holotype fragments found higher in the vertosol, was uncovered perched on top of the concretion. The position of this fragment suggested it had descended through the vertosol to the top of the concretion through self-mulching. This finding suggests that the concretionary layer that hosted the holotype had been at a higher stratigraphic level than that of the concretion in the test trench and had not been exhumed by self-mulching from a lower level. Notably, we have observed many laterally extensive carbonate concretionary layers remaining *in-situ* exposed in the cut banks of rivers in other locations of the Mackunda and Allaru formations. The rock around these hard concretionary layers had been deeply weathered into soft friable layers that developed into soil, leaving the concretions ‘floating’ in the friable layers. The large concretion encountered in our test trench (Fig. 1C) appears to be an example of a concretion remaining *in-situ*. The whitish sand surrounding the concretion is the weathered profile of the Mackunda Formation transitioning to soil. The large quantity of blocks of the holotype originally collected, suggests it unlikely they had been exhumed en masse by self-mulching of the vertosol after weathering out of the bedrock further below. As an alternative possibility, the concretionary layer that hosted the holotype could have descended to the present-day ground level as deflation lag from a higher stratigraphic level over several millions of years of deep weathering and erosion (based on: Holdaway, 2014; Nickling & Neuman, 1995). These elevated and eroding upper stratigraphic levels are evident across the region as flat-topped mesas, colloquially as ‘jump-up’. However, we presently consider that the holotype blocks were more likely stratigraphically in place, rather than transported vertically downwards through deflation. The completeness of the holotype skeleton supports this view.

## Results

### Geochronology

**Figure 2:**
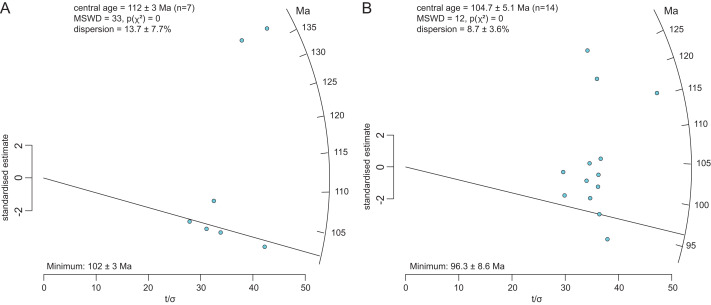
Radial plots of U/Pb ages retrieved from detrital zircons at QML1794N and QML1817. (A, B) Maximum likelihood age (MLA) at (A) QML1794N and (B) QML1817. MLA is read from the solid black line. Ages on the radial axis and relative error are read on *x*-axis. Uncertainty estimates are reported as studentised 95% confidence intervals.

Volcanic tuffs have not been identified in the Mackunda Formation. Due to the lack of tuffs to constrain the stratigraphic age of the Mackunda Formation, we sampled rock from the *Muttaburrasaurus langdoni* holotype locality (QML1794) and two sites in the vicinity of the holotype locality (QML1794N, a marine fossil-bearing sandstone concretion, ∼80 m north of QML1794; QML1817, a marine fossil-bearing sandstone/coquina concretion, ∼50 m southwest of QML1794) to obtain the maximum likelihood age (MLA) (Vermeesch, 2021), which provides a statistically valid estimation of maximum depositional age from concordant U/Pb ages in the detrital zircon population. For the analyses, ^207^Pb/^206^Pb ages are reported as best age for those zircons >1,500 Ma, and ^206^Pb/^238^U ages as best for younger analyses and 208-based common Pb corrections were tested for improved ^207^Pb/^235^U ^206^Pb/^238^U concordance; they were employed if concordance improved (see analytical detail below). Zircons were not recovered from the very fine-grained mudrock sample (QML1794) hosting the holotype, but they were recovered from the sandstones. The data are provided in Data S1. For QML1794N, the mounted zircon sample gave 75 concordant ages from 124 analyses that span from 2,951 ± 73 Ma to 101.1 ± 6.5 Ma (2 s.e.), with a total of seven grains returning a Cretaceous age (U/Pb age radial plot, Fig. 2). For QML1817, 74 concordant ages spanned from 1,406 ± 33.0 Ma to 92.8 ± 4.0 Ma (2 s.e.) with a total of 14 grains returning a Cretaceous age (U/Pb age radial plot, Fig. 2). The sites produced zircon ages that overlap especially in the range of 107 and 101 Ma (*n* = 11). QML1794N gives a MLA of 102 ± 3 Ma, controlled by 5 grains, and QML1817 returns a MLA of 96.3 ± 8.6 Ma. Notably, the youngest single analysis (92.8 ± 4 Ma, 2 s.e.) is within error of the next oldest grains, two at 96.4 ± 4.4 Ma, which strongly contribute to the MLA whose uncertainty (2s.e.) encompasses the single youngest age. The youngest grain could be the best representation of the maximum depositional age. Given the two samples were analysed in a single session it strengthens the supposition that QML1817 contain younger material than QML1794N. A re-evaluation of the pooled detrital zircon U/Pb isotopic data from the previous work of Tucker et al. (2013), Tucker et al. (2016) using the MLA tool (Vermeesch, 2021) for both the Mackunda and Winton formation samples is given here. The pooled detrital zircon age data for the Mackunda Formation returned a MLA of 104.7 ± 1.6 Ma (2 s.e.) from a population of 151 grains (population size almost exactly that of this study). The pooled detrital zircon age for the Winton Formation returned MLA of 93.5 ± 1.2 Ma (2 s.e.) from a total of 614 grains. The Winton Formation age broadly agrees with the maximum depositional age calculation previously estimated (Tucker et al., 2013; Tucker et al., 2016) but this study returned a younger MLA for the Mackunda Formation. The Winton Formation overlies the Mackunda Formation and neither has borne a coherent tuff for sampling. The Mackunda Formation has not been thoroughly sampled and based on our sampling at 2 locations, it is 96.3 ± 8.6 Ma (MLA), which overlaps with but older than that the Winton Formation assessed by Tucker et al. (2013), Tucker et al. (2016). It is notable that the detrital zircon age of the Mackunda Formation reported here based on the coquina at QML1817, is independent of the mudrock hosting the *Muttaburrasaurus langdoni* holotype (*i.e.,* the holotype locality, QML1794). Furthermore, as the flat lying, vertosol covered region gives no vertical stratigraphic association between QML1794 and QML1817, or horizontal facies changes, we are currently unable to determine the exact superpositional relationship between these sites; particularly as they consist of concretions seemingly ‘floating’ in the vertosol (see also under “Locality and horizon” above). Nonetheless, in the absence of fuller stratigraphic data for the formation, we propose that the age of QML1817 (96.3 ± 8.6 Ma) represents the maximum depositional age of the *Muttaburrasaurus langdoni* holotype.

### Systematic palaeontology

**Table:** 

Dinosauria Owen, 1842
Ornithischia Seeley, 1888
Neornithischia Cooper, 1985
Cerapoda Sereno, 1986
Ornithopoda Marsh, 1881

**Genus:** *Muttaburrasaurus* Bartholomai & Molnar, 1981

**Holotype:** QMF6140

**Horizon and Locality:** Rosebery Downs Station, Queensland Museum locality QML1794 (the holotype locality), ∼seven km SE of the Muttaburra township, central western Queensland, Australia. Mackunda Formation, Cenomanian–lower Turonian (96.3 ± 8.6 Ma to 93.51 ± 1.19 Ma).

**Type species:** *Muttaburrasaurus langdoni* (Bartholomai & Molnar, 1981)

**Figure 3:**
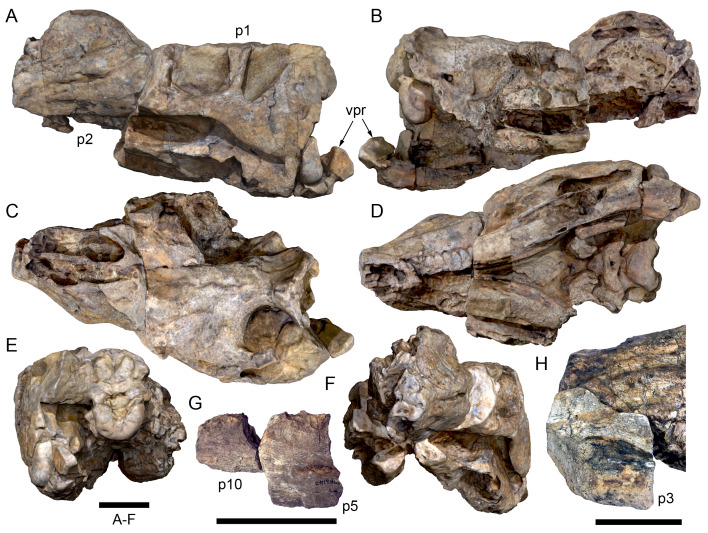
Photogrammetry and photographs of the *Muttaburrasaurus langdoni* (QMF6140) cranium. (A–F) Photogrammetry of main cranial blocks (cranial parts 1 and 2) assembled in (A) left lateral, (B) right lateral, (C) dorsal, (D) ventral, (E) posterior and (F) anterior views. (G) Photograph of left dentary fragments (cranial parts 5 and 10) assembled in left lateral view. (H) Photograph of anterior left and right dentary and predentary fragment (all cranial part 3) in assembly with cranial part 2, in ventrolateral view. Abbreviations: p#, cranial part number; vpr, (uncertain) vertebral process. Scale bars equal 10 cm. DOI: 10.17602/M2M786923.

**Material:** The original description of the holotype skull was based on two primary skull blocks collected in 1963 at the holotype locality (locality now numbered QML1794). For the purposes of the description, these blocks are identified as cranial parts 1 and 2 (Figs. 3A–3E, 3F, 3H). In addition to these blocks, seven further cranial parts collected in 1963 were not previously described. These include: cranial part 3 (Fig. 3H), consisting of a single block hosting fragments of the anterior left and right maxillary tooth crowns, anterior left dentary, anterior right dentary tooth crowns and the left posterolateral process of the predentary; cranial part 4 (not figured), consisting of a poorly preserved anterior right dentary fragment (mostly tooth impressions in the host matrix); cranial part 5 (Fig. 3G), consisting of a mid-left dentary fragment; cranial parts 6, 7, consisting of two connecting, mid-region, left premaxillary dental ramus fragments; cranial part 8 consisting of a right maxillary fragment; and cranial part 11, consisting of a mid-region right dentary fragment. Cranial parts 3 (Fig. 3H), 5 and 11 unequivocally connect with cranial parts 1 and 2. Five additional craniodental fragments were discovered in 2020 at QML1794. These newly discovered parts are referred to the holotype and include a fragment of the anterior right maxilla (cranial part 9), a fragment of the left dentary (cranial part 10), a posterior fragment of the left premaxillary dental ramus (cranial part 12), an anterior fragment of the left premaxillary dental ramus (cranial part 13) and a posterior fragment of the right premaxillary dental ramus (cranial part 14). The newly discovered right maxillary fragment, cranial part 9, connects with the originally collected right maxillary fragment, cranial part 8. Thus, cranial parts 8 and 9 positively locate on the muzzle block (*i.e.,* cranial part 2). The newly discovered left dentary fragment, cranial part 10, inserts between the originally collected left dentary fragments (cranial parts 3 and 5). Detailed criteria identifying the locations of the premaxillary rami fragments are provided within their descriptions, below.

**Revised diagnosis:** A large-bodied non-hadrosauromorph ornithopod characterised by the combination of 15 features of the cranium including 8 potential autapomorphies (*). These include: (1*) posterodorsal and posteroventral processes of the premaxilla that abut along a tight lateral suture to form the anterolateral portion of the enlarged *cavum nasi proprium* within the dorsally inflated muzzle; (2) the nasal excluded from the naris by the premaxilla, convergent with Lambeosaurinae; (3*) novel, paired ossifications of either the premaxillae or neomorphic bones, termed the prenasals, that form the dorsal region of the enlarged *cavum nasi proprium*; (4*) a horizontal septum of the prenasal that divides the *cavum nasi proprium* into inferior (main) and superior airways and a sagittal septum that divides the superior airway into left and right meatuses; (5*) a thickened pendulous support ridge of the prenasal that projects ventrally into the superior airway meatus, partially dividing the airway into sub-chambers; (6) an anterolateral process of the nasal that contributes to the posterolateral part of the dorsally inflated muzzle, convergent with Lambeosaurinae; (7*) a toroidal-shaped fossa at the anterior end of the nasal body forming the posterior end of the superior airway meatus; (8*) a T-shaped lacrimal with a descending process that projects ventrally to the dorsal opening of the maxillary neurovascular tract; (9) a circular fossa on the posteromedial face of the pterygoid process of the quadrate; (10*) the pterygoid lacking palatine contact and vomeral contact on the anterior face of the boss on the pterygoid for the basipterygoid process; (11) a medial process of the squamosal that extends along half of the medial supratemporal fenestral margin; (12) the anteroventral end of the medial process of the squamosal abutting the prootic; (13*) two protuberances formed at the dorsal tip of the ascending process of the supraoccipital accommodated ventrally on the parietals; (14) a dentulous premaxilla with five alveoli, as in early diverging neornithischians; and (15) caniniform premaxillary teeth lacking any substantial constriction between the root and crown, as in some Heterodontosauridae. New postcranial autapomorphies are not proposed.

### Description

For context, some anatomical information provided in the original descriptions of the holotype (Bartholomai & Molnar, 1981; Molnar, 1996) is repeated or amended where necessary. Information from the CT-based, volume rendered reconstructions, is not specifically differentiated from direct external observations, unless where necessary. DOIs for the photogrammetry, crania, dentition and neural endocranium are provided in the Supplemental Information 7.

#### Preservation, deformation and cranial bauplan

**Figure 4:**
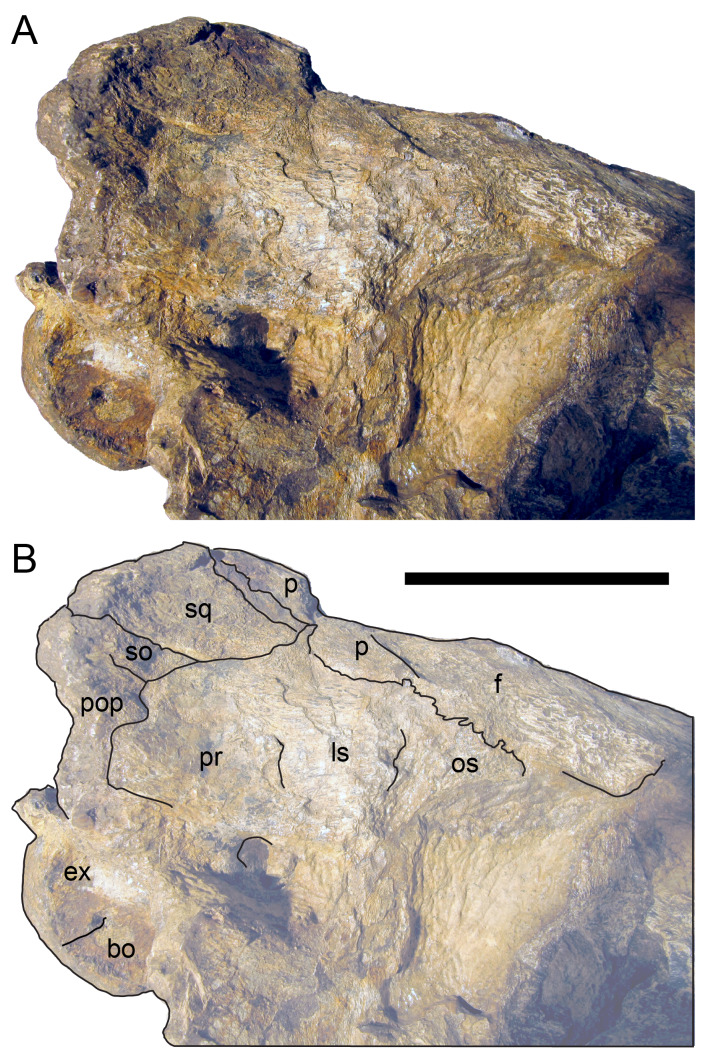
Photograph of the *Muttaburrasaurus langdoni* (QMF6140) neurocranium. (A –B) Photograph (A) of neurocranium (cranial part 1) in right anterolateral view and (B) explanatory schematic overlay of observable bone margins. Abbreviations: bo, basioccipital; ex, exoccipital; f, frontal; ls, laterosphenoid; os, orbitosphenoid; p, parietal; pop, paroccipital process; pr, prootic; so, supraoccipital; sq, squamosal. Scale bar equals 10 cm.

**Figure 5:**
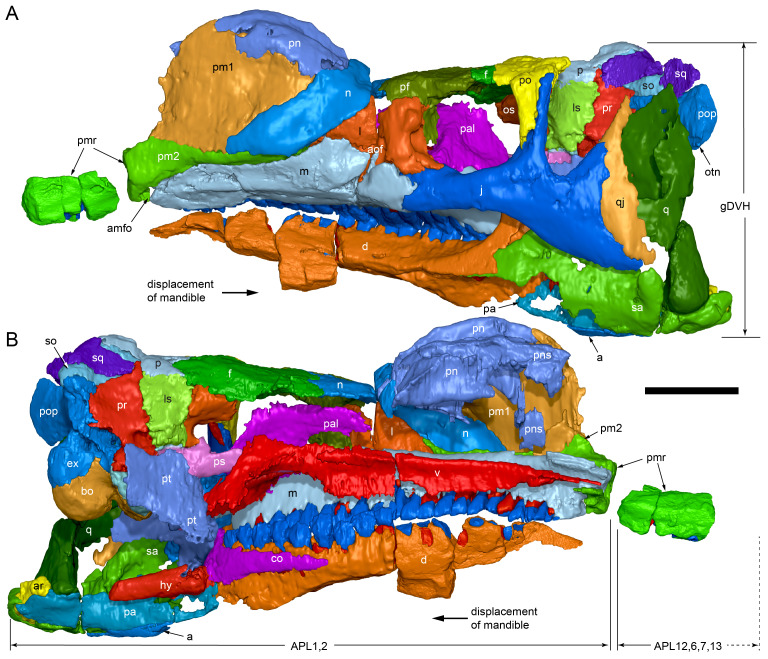
Volume rendered model of the *Muttaburrasaurus langdoni* (QMF6140) cranium. (A, B) Cranium in (A) left lateral and (B) right lateral views (right side cheek region not shown). Abbreviations: a, angular; amfo, anterior maxillary fossa; aof, antorbital fossa; APL#, anteroposterior length and cranial part numbers (dashed line and arrow indicate unknown length anterior to cranial part 13 [not shown]; see Table 1); ar, articular; bo, basioccipital; bs, basisphenoid; co, coronoid; d, dentary; ex, exoccipital; f, frontal; gDVH, greatest dorsoventral height (see Table 1); hy, hyoid; j, jugal; l, lacrimal; ls, laterosphenoid; m, maxilla; n, nasal; os, orbitosphenoid; otn, otic notch; p, parietal; pa, prearticular; pal, palatine; pf, prefrontal; pm1, posterodorsal process of premaxilla; pm2, posteroventral process of premaxilla; pmr, dental ramus of premaxilla; pn, prenasal; pns, prenasal septa; po, postorbital; pop, paroccipital process; pr, prootic; ps, parasphenoid; pt, pterygoid; q, quadrate; qj, quadratojugal; ra, retroarticular; sa, surangular; so, supraoccipital; sq, squamosal; v, vomer. Scale bar equals 10 cm. MorphoSource DOI: 10.17602/M2/M788197.

**Figure 6:**
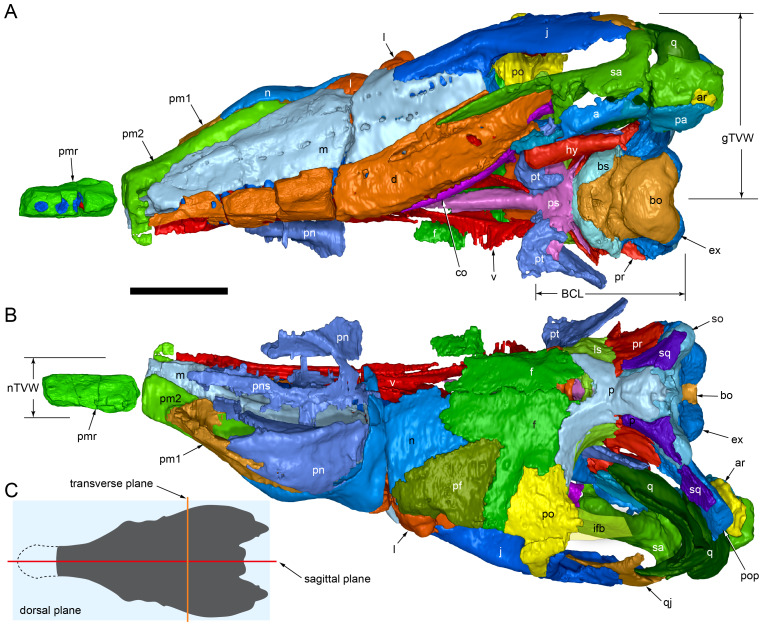
Volume rendered model of the *Muttaburrasaurus langdoni* (QMF6140) cranium and schematic orientation of the anatomical planes. (A, B) Volume rendered model of cranium in (A) dorsal and (B) ventral views (poorly preserved right side cheek region not modelled). (C) Schematic profile of the cranium in dorsal view with anatomical planes indicated (dashed line indicates region represented by left premaxillary fragment, cranial part 13). Abbreviations: a, angular; ar, articular; BCL, basicranial length (see Table 1); bo, basioccipital; bs, basisphenoid; co, coronoid; d, dentary; ex, exoccipital; f, frontal; gTVW, greatest transverse width (left half, see Table 1); hy, hyoid; ifb, interfenestral bar; j, jugal; l, lacrimal; ls, laterosphenoid; m, maxilla; n, nasal; nTVW, narrowest transverse width (left half, see Table 1); p, parietal; pa, prearticular; pf, prefrontal; pm1, posterodorsal process of premaxilla; pm2, posteroventral process of premaxilla; pmr, dental ramus of premaxilla; pn, prenasal; pns, prenasal septa; po, postorbital; pop, paroccipital process; pr, prootic; ps, parasphenoid; pt, pterygoid; q, quadrate; qj, quadratojugal; ra, retroarticular; sa, surangular; so, supraoccipital; sq, squamosal; v, vomer. Scale bar equals 10 cm. MorphoSource DOI: 10.17602/M2/M788197.

**Figure 7:**
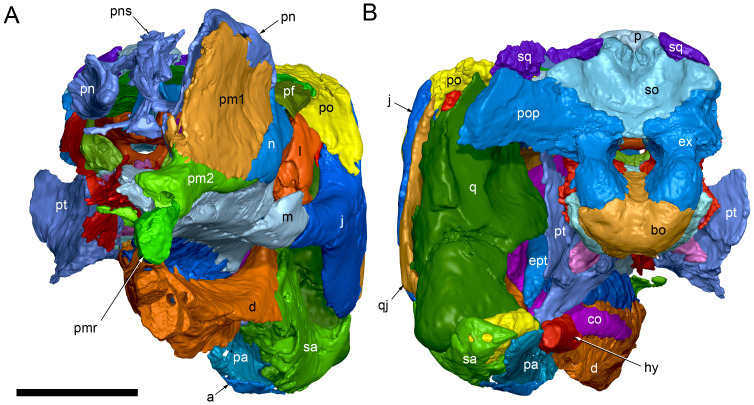
Volume rendered model of the *Muttaburrasaurus langdoni* (QMF6140) cranium. (A, B) Cranium in (A) anterior and (B) posterior views (poorly preserved right side cheek region not modelled). Abbreviations: a, angular; ar, articular; bo, basioccipital; bs, basisphenoid; co, coronoid; d, dentary; ept, ectopterygoid; ex, exoccipital; f, frontal; hy, hyoid; j, jugal; l, lacrimal; m, maxilla; n, nasal; p, parietal; pa, prearticular; pf, prefrontal; pm1, posterodorsal process of premaxilla; pm2, posteroventral process of premaxilla; pmr, dental ramus of premaxilla; pn, prenasal; pns, prenasal septa; po, postorbital; pop, paroccipital process; pr, prootic; ps, parasphenoid; pt, pterygoid; q, quadrate; qj, quadratojugal; ra, retroarticular; sa, surangular; so, supraoccipital; sq, squamosal. Scale bar equals 10 cm. MorphoSource DOI: 10.17602/M2/M788197.

The margins between many of the bones on the main cranial blocks (cranial parts 1 and 2; Fig. 3) are difficult to identify solely from external observations. In addition, many of the neurocranial (braincase) bones are fused or at least partially fused (Fig. 4), suggesting that the individual was near somatic maturity but possibly not fully mature. Many of the sutural, fused and abutting margins between the bones were clarified from the CT imagery. The left side of the skull is more complete than the right. The bauplan of the skull is shown in Figs. 5–7, noting the right-side maxillofacial region was omitted from volume rendering due to being incomplete and degraded. Selected measurements of the skull and individual cranial elements are provided in Table 1. The anatomical planes of reference are indicated in Fig. 6C. Bones on the main cranial blocks are generally well preserved, although some stripping/shedding of the bone surfaces is apparent, possibly from hydraulic abrasion and/or recent subaerial weathering. The neurocranium is virtually complete. On the main blocks, the ventral portions of the mandibles, the dorsal-most region of the nasal cavity, some dorsal surfaces of the cranial roof bones and posterolateral parts of the cranium are eroded or missing. The skull has been dorsoventrally compacted, with compaction increasing obliquely towards the right anterolateral corner. Deformation appears to have been plastic occurring during fossilisation rather than through brittle fracture prior to burial (see Arbour & Currie, 2012). Viewed anteriorly, the left maxilla, jugal, quadratojugal, palatine and ectopterygoid succumbed to clockwise deformational rotation. Viewed dorsally, these bones, along with the vomera, were additionally rotated in a clockwise direction relative to the sagittal axis. As a result, the anterior end of the left maxilla is skewed to the right. Compaction further resulted in dorsal displacement of the left quadrate by ∼20–25 mm, relative to the left pterygoid and neurocranium. The left quadrate appears to have been displaced posteriorly relative to the jugal and quadratojugal by up to 25 mm, as indicated by the left mandible (see further below).

**Table 1:** Table of primary measurements of the *Muttaburrasaurus langdoni* (QMF6140) cranium and stylopodial elements. Illustrated views of the cranial and premaxillary ramus anteroposterior lengths and greatest dorsoventral height of the cranium at the parietals, see Fig. 5. Illustrated views of greatest and narrowest cranial transverse widths (left side only), see Fig. 6. Anteroposterior length of the basicranium is taken from the posterior-most tip of the occipital condyle to the anterior end of the parasphenoid, excluding the cultriform process (see Fig. 6). Anteroposterior length of the temporal fossa taken between the posterior margin of the postorbital descending (jugal) process and the lateral-most point on the medial surface of the paroccipital process. Transverse width of temporal fossa taken between the laterosphenoid-prootic suture and medial surface of the quadratojugal. Transverse width of occiput taken from lateral-most tip of the paroccipital process to sagittal midline on the left side.

Element	Measurement (mm)
Skull, anteroposterior length of cranial parts 1 + 2 (Fig. 5)	630.0
Skull, greatest transverse width, based on left side x 2 (Fig. 6)	400.0
Skull, narrowest transverse width, based on left side x 2 (Fig. 6)	122.0
Skull, greatest dorsoventral height (Fig. 5)	326.0
Premaxillary ramus, anteroposterior length based on cranial parts 6, 7, 12, 13 on left side	162.0inc
Basicranium, anteroposterior length (measured from distal-most margin of basioccipital to anterior-most point of parasphenoid, excluding cultriform process)	146.9
Left orbit, anteroposterior length/dorsoventral height	1.5.0/102.0
Right antorbital fossa/antorbital fenestra, anteroposterior length/dorsoventral height	32.0/24.5
Left temporal fossa, greatest anteroposterior length/transverse width (anteroposterior length, taken from posterior margin of the postorbital descending (jugal) process to lateral-most point on the medial surface of the paroccipital process; transverse width taken from laterosphenoid-prootic suture to medial surface of the quadratojugal)	∼160.0/∼130.0
Occiput, transverse width based on left side x 2 (taken from lateral-most tip of the left paroccipital process to sagittal midline)	290.0
Left Jugal, anteroposterior length	241.6
Left frontal, anteroposterior length/transverse width	141.2/136.0
Left quadrate, dorsoventral height	228.0e
Left maxilla, anteroposterior length/dorsoventral height	371.0/93.0
Left maxilla/dentary, alveolar axis length	330.0/335.0
Left, fourth, unerupted premaxillary tooth crown mesiodistal length; labiolingual width; apicobasal height	12.1; 9.8; 14.0
Left erupted maxillary crown, alveolar position/mesiodistal width	m1/5.6; m2/6.6; m4/10.3; m6/14.0; m7/14.8; m9/19.3; m13/16.8; m14/17.8; m15/16.9; m17/17.7; m20/15.6; m21/14.6
Z spacing, left maxillary dentition	m4-m5, 2.3; m9-m10, 2.1; m12-13, 1.8; m14-m15, 2.2; m17-m17, 2.0
Left mandible, anteroposterior length/greatest dorsoventral height	654.5inc/135.0
Left erupted dentary crown, alveolar position/mesiodistal width	d4/19.5e; d5/18.2e; d6/18.2e; d11/24.7; d13/27.5; d15/25.7; d18/10.0
Femur, minimum diaphyseal circumference (l, left; r, right; mean)	460.0 ± 10 l; 500.0 ± 10 r; 480.0 ± 10
Humerus, minimum diaphyseal circumference on left side	280.0 ± 2.0

The mandibular corpora were displaced post-mortem, indicated by rotation along their anteroposterior axes. Viewed anteriorly, the left mandibular corpus was rotated anticlockwise by 31° relative to the maxilla (see under “Maxilla” below). As a result, the occlusal surfaces of the maxillary and dentary teeth are misaligned and the bones of the mandibular corpus and the cranial cheek region show some compaction. The right mandible rotated in the clockwise direction to a greater extent than the left side, by at least 40°. In addition to rotation, examination of the dentary and maxillary tooth row alignment suggests that the left mandibular corpus (along with the left quadrate) was displaced posteriorly relative to the maxilla by up to 25 mm (Fig. 5B). In life, the predentary process of the dentary would have aligned more closely with the dental ramus of the premaxilla than preserved (see further under “Predentary”). In addition to rotational displacement, the left surangular was possibly displaced posteriorly from the dentary and coronoid by ∼8 mm. A dorsally directed bony protuberance is present at the posterior end of the left mandibular corpus in the location of the retroarticular process (Figs. 3A, 3B). CT examination, however, reveals a layer of matrix attaching the protuberance to the posterior end of the retroarticular process and the anomalous bone is assessed as a displaced vertebral process fragment of currently uncertain location.

Viewed dorsoventrally, the skull has a strongly triangular profile (Figs. 3, 6), being broadest transversely across the infratemporal region of the jugals/quadratojugals. With the addition of the newly identified parts of the left premaxillary ramus (cranial parts 6, 7, 12, 13), a total anteroposterior length of 790 mm is estimated for the cranium from the posterior tip of the retroarticular process to the assumed anterior tip of the premaxilla, taken at the anterior end of cranial part 13. This estimate includes the subtraction of ∼20 mm for posterior displacement of the mandible and quadrate. The dorsoventral height of the skull from the ventral-most margin of the left angular to the dorsal-most point on the parietals is ∼326 mm. The skull is transversely broad, with a width of 400 mm estimated by doubling the left half and measured between the quadratojugals. The total dorsoventral height (*i.e.,* including the mandible) is ∼81% that of the maximum transverse width. The estimated transverse width of the skull is 51% of the total anteroposterior length. The dental ramus of the premaxilla is transversely narrow with the narrowest part at the truncated anterior end of the muzzle block (cranial part 2) ∼31% of the maximum transverse width.

#### Nasal complex overview

**Figure 8:**
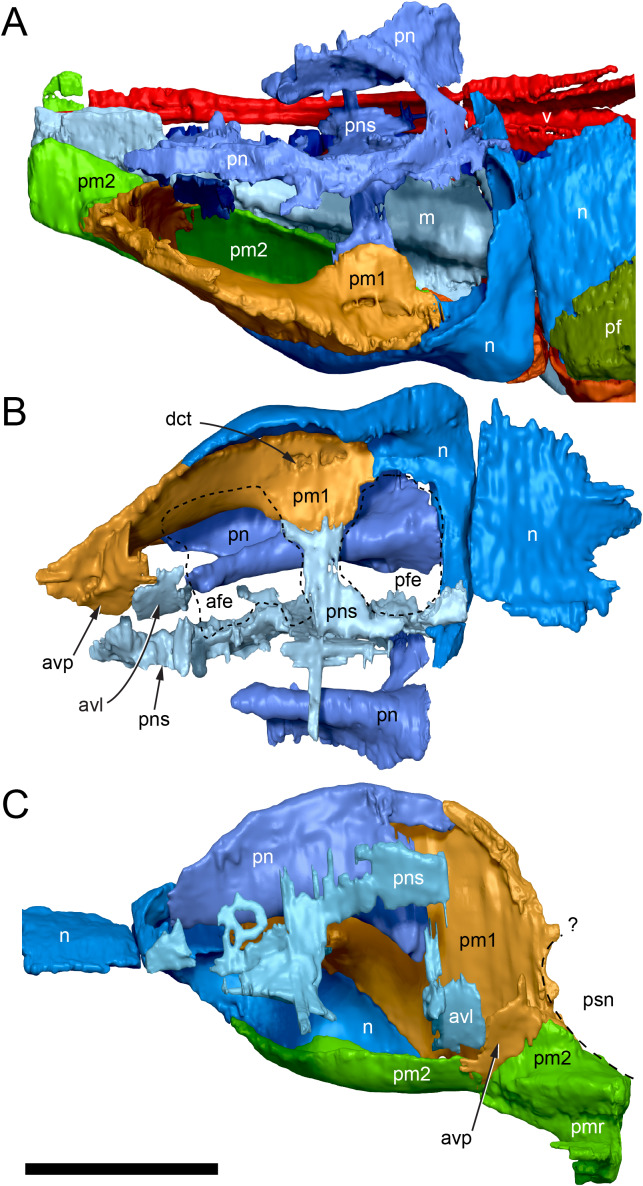
Volume rendered model of the *Muttaburrasaurus langdoni* (QMF6140) hypertrophied muzzle complex. (A–C) Muzzle complex in (A) dorsal view with dorsolateral portion of the left prenasal removed and bones ventral to the muzzle complex (left maxilla and vomers) included, (B) ventral view with the left posteroventral process and posterior portion of the dental ramus of the premaxilla removed (dashed lines indicate fenestral margins between the inferior [main] and superior airways) and (C) medial view of left side with dorsal portion of the right prenasal removed (dashed line indicates pseudonarial margin). Abbreviations: afe, anterior fenestra; avl, anteroventral septal lamina; avp, anteroventral process; dct, duct; m, maxilla; n, nasal; nar, naris; pf, prefrontal; pfe, posterior fenestra; pm1, posterodorsal process of premaxilla; pm2, posteroventral process of premaxilla; pmr, dental ramus of premaxilla; pn, prenasal; pns, septa of prenasal; psn, pseudonares; v, vomer.; ?, uncertain osteology. Scale bar equals 10 cm.

A dorsally inflated muzzle is a characteristic feature of *Muttaburrasaurus langdoni* and originally thought to be formed by the nasals (Bartholomai & Molnar, 1981; Molnar, 1996). However, CT data now show that the dorsally hypertrophied region of the muzzle is formed by a bone complex rather than solely by the nasals (Fig. 8). The paired nasals form the posterodorsal end and posterolateral sides of the muzzle complex. Two extensive posterior processes of the premaxillae form the anterodorsal and anterolateral regions of the muzzle and an additional pair of unique ossifications, termed the prenasals, form the posterodorsal roof of the muzzle. The prenasals are either novel dorsal processes of the premaxilla or novel neomorphic bones (see further under “Prenasal” below). Internally, the nasal cavity is divided into dorsal and ventral meatuses that are separated by thin horizontal bony septa and processes. The ventral meatus constitutes the main airway, which is further termed the inferior airway. The dorsal airway, enclosed by the dorsally protrusive bones of the muzzle, is divided into paired chambers by sagittal (vertical) septa of the prenasals. The paired dorsal airways are further termed, the superior airways. The inferior and superior airways communicate through two fenestrae—one located anteriorly and the other posteriorly—which are divided by the horizontal septa and processes (Fig. 8B). The features dividing and connecting the inferior and superior airways will be described in detail in the descriptions that follow. The inferior airway connects the nares and choanae, as in all dinosaurs. However, the multi-chambered superior airways are uniquely formed in *Muttaburrasaurus langdoni* by the internal septa and ridges of the prenasal ossifications (Fig. 8).

#### Premaxilla

**Figure 9:**
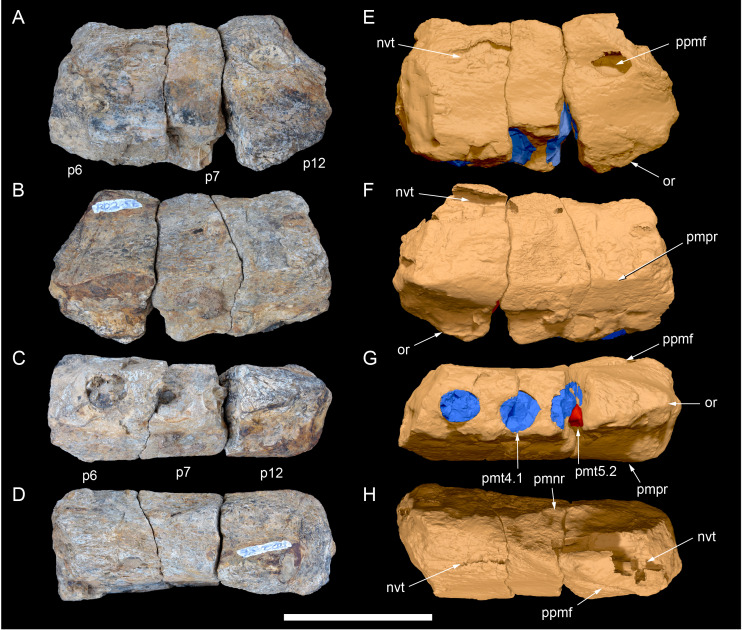
Photographs and volume rendered model of the *Muttaburrasaurus langdoni* (QMF6140) left premaxillary dental ramus (cranial parts 6, 7, 12). (A–D) Photographs of partial left premaxillary dental ramus in (A) lateral, (B) medial, (C) ventral and (D) dorsal views. (E–H) Volume rendered model of partial left premaxillary dental ramus in (E) lateral, (F) medial, (G) ventral and (H) dorsal views. Abbreviations: dia, diastema; nvt, neurovascular tract; or, oral ridge; p#, cranial part number; pmpr, palatal ridge of premaxilla; pmt-#, premaxillary tooth position/family and development number (.1 = functional tooth [blue]; .2 = germ tooth [red]); ppmf, posterolateral premaxillary foramen. Scale bar equals five cm. MorphoSource DOI: 10.17602/M2/M786859; 10.17602/M2/M786862; 10.17602/M2/M786887.

**Figure 10:**
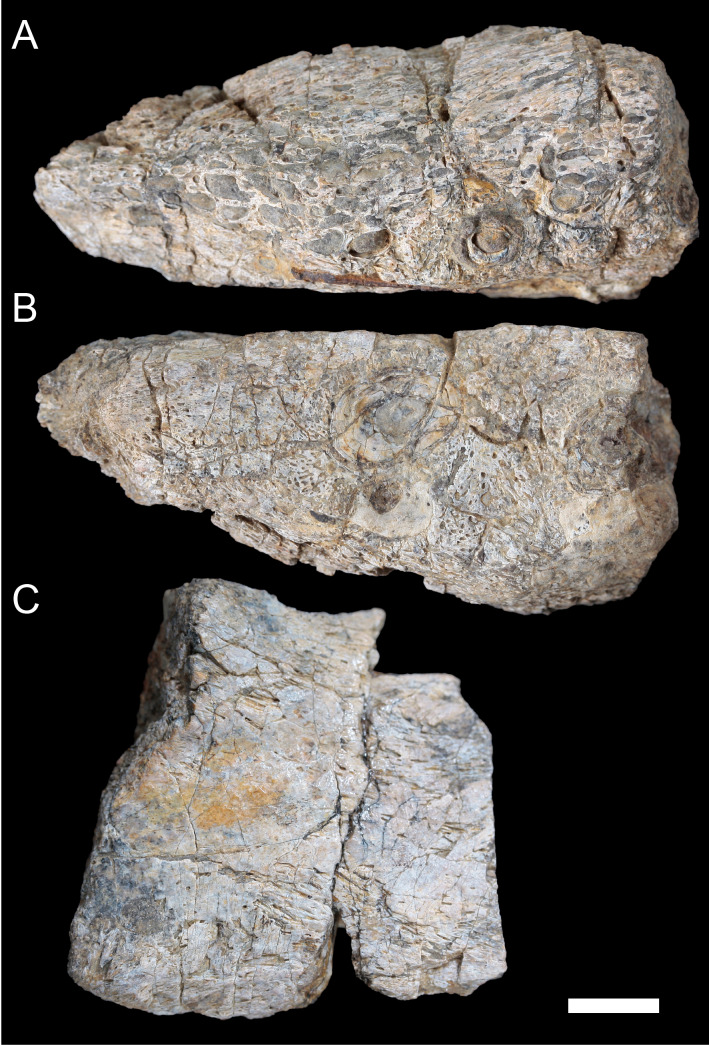
Photographs of *Muttaburrasaurus langdoni* (QMF6140) premaxillary dental ramus fragments. (A, B) Left anterior-most dental ramus fragment (cranial part 13) in (A) dorsal and (B) ventral views. (C) Right posterior-most dental ramus fragment (cranial part 14) in lateral view. Scale bar equals one cm.

**Figure 11:**
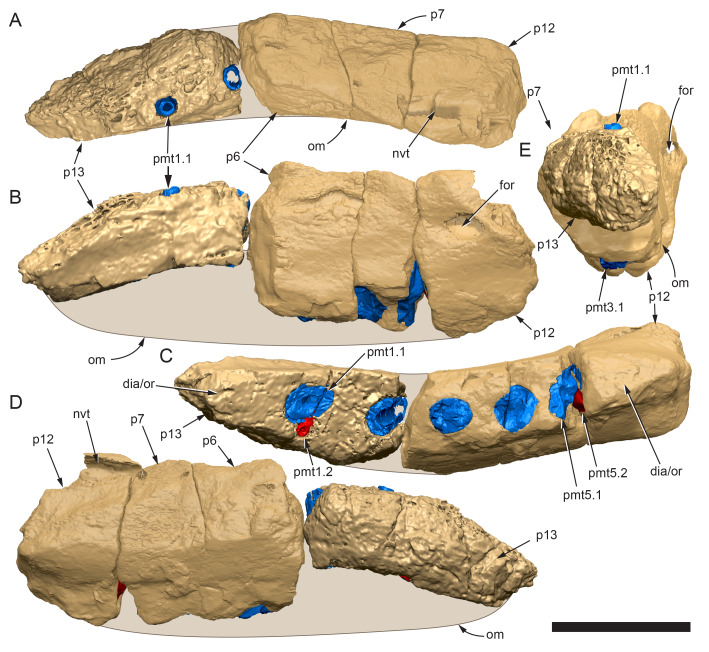
Volume rendered model of the* Muttaburrasaurus langdoni* (QMF6140) left premaxillary dental ramus. (A–E) Left premaxillary dental ramus (cranial parts 6, 7, 12, 13) in (A) dorsal, (B) lateral, (C) ventral, (D) medial and (E) anterior views. Brown shaded areas suggest missing bone. Abbreviations: dia, diastema; for, foramen; nvt, neurovascular tract; om, oral margin; or, oral ridge; p#, cranial part number; pmt-#, premaxillary tooth position/family and development number (.1 = functional tooth [blue]; .2 = germ tooth [red]). Scale bar equals five cm. MorphoSource DOI: 10.17602/M2/M786859; 10.17602/M2/M786862; 10.17602/M2/M786887; 10.17602/M2/M786850; 10.17602/M2/M786853; 10.17602/M2/M786856.

**Figure 12:**
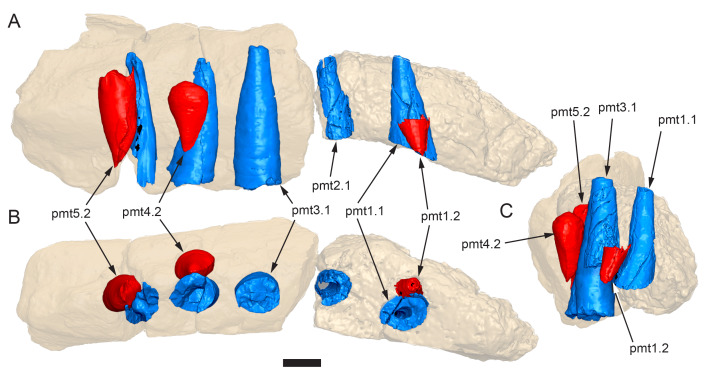
Volume rendered model of *Muttaburrasaurus langdoni* (QMF6140) premaxillary dentition. (A–C) Left premaxillary dentition (cranial parts 6, 7, 12, 13) in (A) medial, (B) ventral and (C) posterior views. Bone of the dental ramus shown faded. Abbreviation: pmt#, premaxillary tooth position/family and development number (.1 = functional tooth [blue]; .2 = germ tooth [red]). Scale bar equals one cm. MorphoSource DOI: 10.17602/M2/M786859; 10.17602/M2/M786862; 10.17602/M2/M786887; 10.17602/M2/M786850; 10.17602/M2/M786853; 10.17602/M2/M786856.

**Figure 13:**
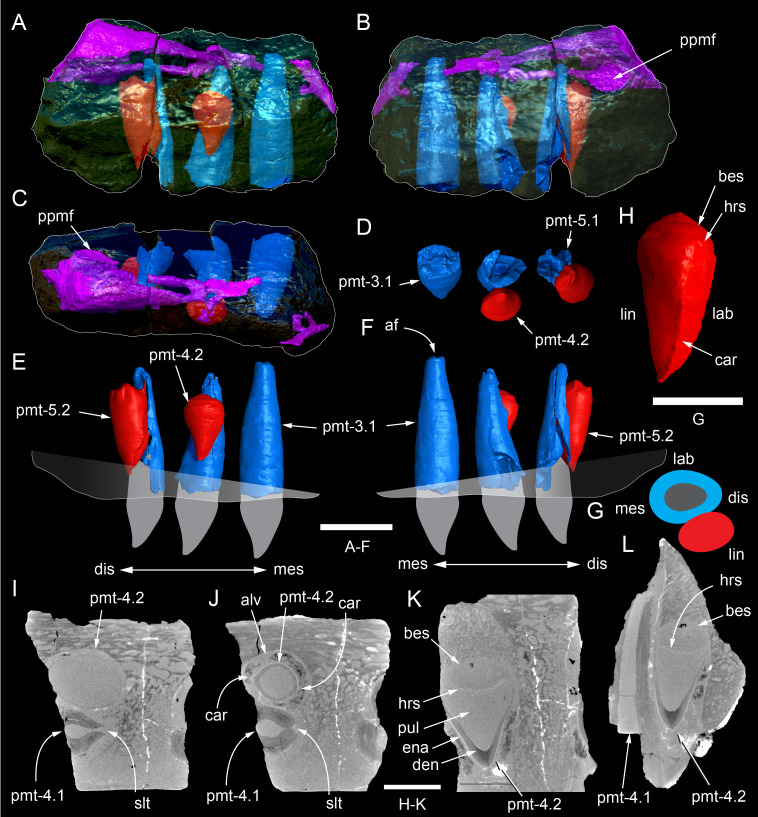
Volume rendered model and CT generated radiographs of the *Muttaburrasaurus langdoni* (QMF6140) left premaxillary dental ramus and dentition. (A–D) Volume rendered model of left premaxillary dental ramus in (A) medial, (B) lateral, (C) dorsal and (D) ventral views with bone of the dental ramus digitally clarified to expose dentition internally and the neurovascular tract (coloured magenta). (E, F) Volume rendered model of dentition in (E) medial and (F) lateral views showing reconstruction of the missing functional crowns (coloured grey) with ventral extent of the ramus suggested for context. (G) Diagram illustrating the ‘egg-shaped’ asymmetry of the root in dorsal cross section; useful in determining mesiodistal direction of the tooth and premaxillary ramus, particularly in the absence of a crown (blue shading functional tooth root; red shading, developing crown; grey shading, pulp cavity). (H) Volume rendered model of germ tooth at pmt4 in mesial view. (I, J) Radiographs of cranial part 7 in the dorsal plane showing tooth root and crown histological features in the region of pmt4 the (I) bell and (J) developed crown base levels. (K, L) Micro-CT generated radiograph of cranial part 7 showing tooth root and crown histological features in the region of pmt4 in (K) parasagittal and (L) coronal views. Abbreviations: af, apical foramen of root; alv, alveolus; bes, bell stage; car, carina; den, dentine; dis, distal; ena, enamel; hrs, (diaphragm of) Hertwig’s epithelial root sheath; lab, labial; lin, lingual; mes, mesial; pmt-#, premaxillary tooth position/family and development number (.1 = functional tooth [blue]; .2 = germ tooth [red]); ppmf, posterolateral premaxillary foramen; pul, pulp; slt, slot; ?, missing region of ramus. Scale bar A–F equals two cm and G equals one cm. MorphoSource DOI: 10.17602/M2/M786859; 10.17602/M2/M786862; 10.17602/M2/M786887.

**Figure 14:**
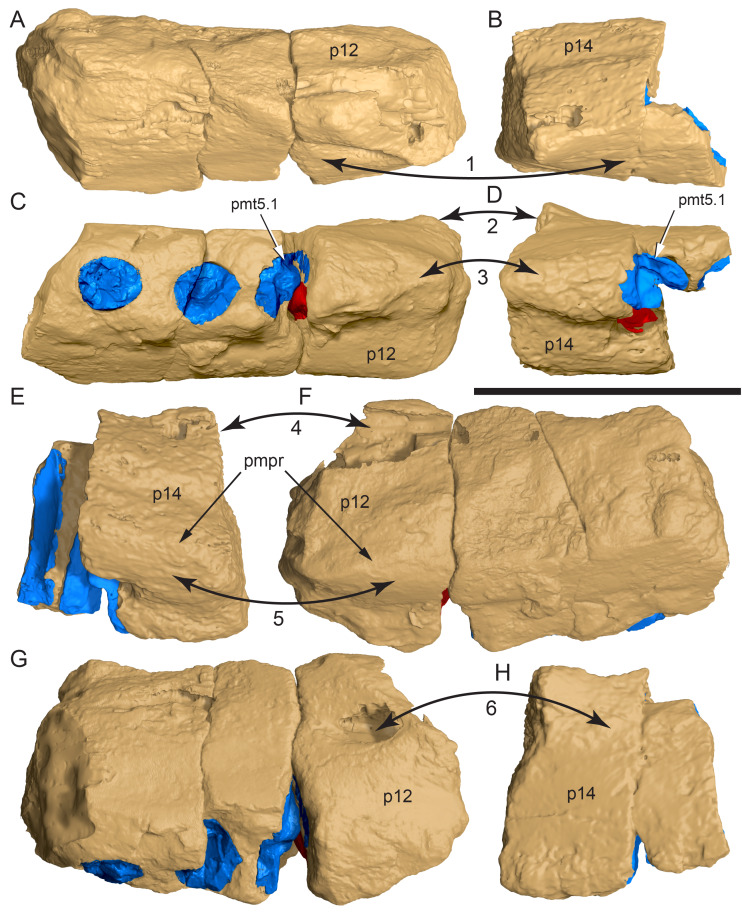
Comparative volume rendered models of the left and right premaxillary dental rami of *Muttaburrasaurus langdoni* (QMF6140). (A, B) Left (A) and right (B) dental rami in dorsal view. (C, D) Left (C) and right (D) dental rami in ventral view. (E, F) right (E) and left (F) dental rami in medial view. (G, H) right (G) and (H) left dental rami in lateral view. Arrows indicate congruence between the left and right sides at (1) lateral surface profiles, (2) region of dorsolateral flaring, (3) oral ridges and posterior diastemas, (4) position of the internal neurovascular canal and (5) shape of the medial palatal shelf. Arrow (6) indicates the occurrence of the neurovascular foramen and fossa on the left side and absence of feature on the right side. Abbreviations: p#, cranial part number; pmpr, palatal ridge of premaxilla; pmt#, premaxillary tooth position/family and development number (.1 = functional tooth [blue]; .2 = germ tooth [red]). Scale bar equals five cm. MorphoSource DOI: 10.17602/M2/M786859; 10.17602/M2/M786862; 10.17602/M2/M786887; 10.17602/M2/M786841; 10.17602/M2/M786844; 10.17602/M2/M786847.

**Figure 15:**
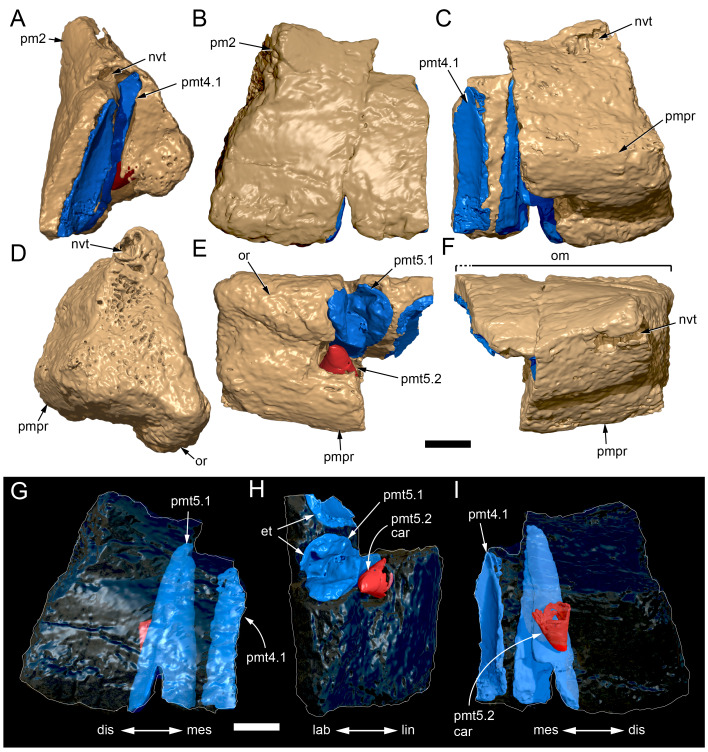
Volume rendered model of the *Muttaburrasaurus langdoni* (QMF6140) right, posterior premaxillary dental ramus fragment. (A–F) Right, posterior premaxillary dental ramus (cranial part 14) in (A) anterior, (B) lateral, (C) medial, (D) posterior, (E) ventral and (F) dorsal views. (G–I) Cranial part 14 with bone digitally clarified to show dentition in (G) lateral, (H) ventral and (I) medial views. Abbreviations: car, carina; dis, distal; et, functional tooth; lab, labial; lin, lingual; mes, mesial; nvt, neurovascular tract; om, oral margin; or, oral ridge; pm2, posteroventral process of premaxilla; pmpr, palatal ridge of premaxilla; pmt#, premaxillary tooth position/family and development number (.1 = functional tooth [blue]; .2 = germ tooth [red]). Scale bars equal one cm. MorphoSource DOI: 10.17602/M2/M786841; 10.17602/M2/M786844; 10.17602/M2/M786847.

Among the craniodental fragments of the holotype skull collected in 1963 and 2020, incomplete left and right premaxillary dental rami are identified (cranial parts 6, 7, 12–14; Figs. 9–15). Functional crowns are not preserved in any of the alveoli. However, their roots are present, and the crowns of several germ teeth are revealed in the CT data. Cranial parts 6, 7 and 12 connect along their broken ends to form a substantial middle and posterior portion of the left premaxillary dental ramus (Fig. 9). Cranial part 13 appears to form the anterior end of the left dental ramus, anterior to cranial part 6 (Figs. 10A, 10B, 11, 12), although direct connection between these parts is not evident. Only the subnarial parts of the left and right dental rami are preserved and the dorsal regions are missing. The anterior-most end of the left dental ramus is additionally missing anterior to the oral ridge on cranial part 13 (Fig. 11) and cranial part 14 only consists of the posterior end of the right dental ramus (Fig. 10C).

Identification of the left dental ramus (cranial parts 6, 7, 12, 13; Figs. 9, 10A, 11–13) is based on the combination of five features: (1) identification of the lateral and medial surfaces; (2) the lingual position of the germ teeth; (3) distal/posterior offset of the germ crowns relative to the roots of the functional teeth, as in ornithischians such as, *Changchunsaurus parvus* (Jin et al., 2010, fig. 2) and *Yinlong downsi* (Han et al., 2016, fig. 7); (4) distally directed recurvature of the germ crowns; and (5) continuity of the internal neurovascular tract between the muzzle block (cranial part 2) and cranial part 12. The profile of the broken posterior end of the left dental ramus (*i.e.,* cranial part 12) is roughly concordant with the broken surface of the premaxilla at the end of the muzzle block (cranial part 2). However, because of bone loss through breakage and erosion, the two ends do not directly connect.

Although cranial part 13 does not directly connect with cranial part 6 (possibly owing to breakage and erosion), three specific features place it at the anterior end of the left dental ramus: (1) a stronger carina mesially than distally on the crown, comparable to the mesial carinae on the germ crowns in the posterior region of the dental ramus; (2) directionality of the egg-shaped cross-section of the root (Fig. 13G; see further details under the description of the premaxillary dentition, below); and (3) continuity of the lateral surfaces of the ramus and the dentition between the parts (Figs. 11, 12). However, this placement is provisional. The distance of 11.5 mm between the two alveoli on part 13 is substantially greater than the distance of 5.5 mm between the three alveoli on the posterior fragment (*i.e.,* cranial parts 6, 7, 12). Nevertheless, placement of cranial part 13 on the holotype skull, other than anterior to cranial part 6, is problematic. Notably, if cranial part 13 was positioned as the posterior part of the dental ramus on the right side, that position would overlap with cranial part 14, which is identified in that position (see further below). Hence, restoration of the left dental ramus places cranial part 13 anteriorly (Fig. 11). The left dental ramus possesses five alveoli (Fig. 11), as in a range of early diverging neornithischians, such as, *Agilisaurus louderbacki* (Peng, 1990), *Bugenasaura infernalis* (Galton, 1999), *Changchunsaurus parvus* (Jin et al., 2010), *Orodromeus makelai* (Scheetz, 1999) and *Zephyrosaurus schaffi* (Sues, 1980) and the early diverging ornithopod, *Hypsilophodon foxii* (Galton, 1974).

Viewed dorsoventrally, the lateral subnarial surface of the left dental ramus is anteroposteriorly concave (Figs. 11A, 11C). Although much of the left oral margin is missing, the lateral surface as preserved on cranial part 12, suggests that the oral margin was at best only slightly everted (Figs. 11A, 11E), as in *Hypsilophodon foxii* (Galton, 1974) and *Thescelosaurus neglectus* (Boyd, 2014). Thus, the subnarial surface forms a shallow lateral fossa. Viewed ventrally, the alveolar axis is laterally concave (Fig. 11C). The oral ridge, which surrounds the alveoli ventral to the palatal ridge, pinches out both anteriorly and posteriorly, forming diastemas of approximately two alveolar lengths at each end (Figs. 9A–9C, 11C). Unlike *Tenontosaurus tilleti* (Thomas, 2015), rhabdodontomorphs (Weishampel et al., 2003; Zanno et al., 2023) and dryomorphs, generally (see Norman, 2004), a subnarial fossa is absent. In this aspect, the dental ramus is comparable to early diverging neornithischians, such as *Thescelosaurus neglectus* (Boyd, 2014), *Changchunsaurus parvus* (Jin et al., 2010), *Haya griva* (Barta & Norell, 2021) and the early diverging ornithopod, *Hypsilophodon foxii* (Galton, 1974).

Posteriorly on the left dental ramus, a large, elliptical neurovascular foramen occurs laterally, inset within a fossa (Fig. 9A). The foramen branches from the internal neurovascular tract, which extends anteroposteriorly through the ramus at the level of the root apices (Figs. 13B, 13C). The neurovascular tract carried the maxillary branch of the trigeminal nerve (cn V). The enlarged neurovascular foramen is an unusual feature not previously described in an ornithopod. Posterior to the foramen, the surface flares dorsolaterally towards the posteroventral process, as preserved on cranial part 2 (Figs. 11C, 11E). Medially, the palatal ridge is thickened (“pmpr” in Figs. 9, 14) and appears to have lacked development of a distinct shelf, although breakage could have caused loss of bone medially. However, a well-developed palatal shelf occurs on the posterior-most portion of the left premaxillary ramus, as preserved on the muzzle block (cranial part 2; see further below). The neurovascular tract expands towards the broken posterior end of the dental ramus (Figs. 13A–13C). The size and position of the neurovascular tract at the broken end of cranial part 12 is congruent with the neurovascular tract exposed through breakage on the muzzle block (cranial part 2; see further below).

Cranial part 14 is identified as the posterior fragment of the right premaxillary dental ramus (Figs. 10C, 14, 15). The fragment preserves two alveoli with roots of the functional dentition and one germ tooth crown (Fig. 15). Identification of part 14 as the right dental ramus is based on five features congruent with the left side (see Figs. 14A–14F): (1) anteroposterior convexity of the lateral surface; (2) posterolateral flaring of the dental ramus in the direction of the posteroventral process (see further below); (3) shape and size of the posterior diastema on the oral ridge; (4) location of the internal neurovascular tract; and (5) shape and size of the thickened palatal ridge. In addition, the functional tooth roots and posterior germ crown on cranial part 14, mirror those of the left dental ramus (Figs. 13D, 13E, 13H, 15E, 15H, 15I). The distance between the alveoli of 6.0 mm is congruent with the distance between the alveoli of 5.5 mm on the left cranial parts 6, 7 and 12. The posterior region of the right dental ramus differs from the left by lacking a posterolateral neurovascular foramen (Figs. 14G, 14H, 15B). Morphological disparity in this feature between the right and left premaxillary rami is considered here as the result of naturally occurring asymmetry of the vascular pattern between the two sides, which is known to occur in the cranial vasculature among individuals of birds (Baumel, 1967) and crocodilians, such as *Alligator mississippiensis* (Porter, Sedlmayr & Witmer, 2016). The right dental ramus has a roughly triangular transverse cross-section with the ventromedial surface of the thickened palatal ridge convex, as on the left side (Figs. 14E, 15A, 15D).

**Figure 16:**
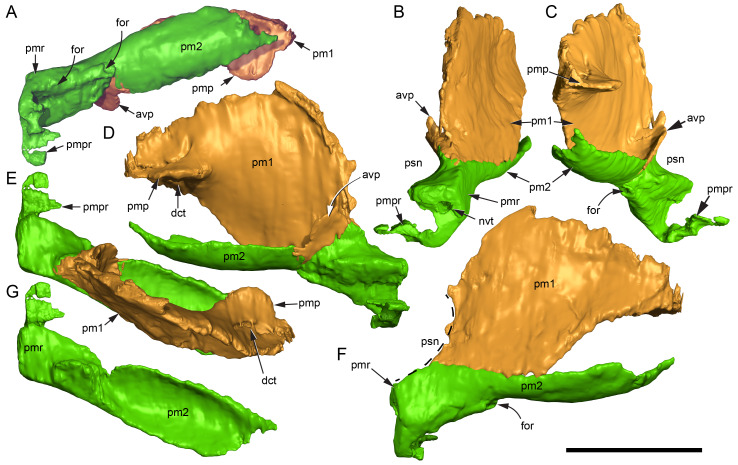
Volume rendered model of the *Muttaburrasaurus langdoni* (QMF6140) posterior processes of the premaxilla. (A–F) Left posterodorsal (yellow) and posteroventral (green) premaxillary processes in (A) ventral, (B) anterior, (C) posterior, (D) medial, (E) dorsal and (F) lateral views. (G) Left posteroventral process in dorsal view. Abbreviations: avp, anteroventral process; dct, duct; for, foramen; nvt, neurovascular tract; pm1, posterodorsal process of premaxilla; pm2, posteroventral process of premaxilla; pmp, posteromedial process; pmr, dental ramus of premaxilla; pmpr, palatal ridge of premaxilla; psn, pseudonaris. Scale bar equals 10 cm. MorphoSource DOI: 10.17602/M2/M787709; 10.17602/M2/M78771.

At the anterior end of the muzzle block (cranial part 2; Fig. 16), the posterior portion of the left premaxillary dental ramus and shelf-like palatal ridge are preserved. In this posterior region of the dental ramus, the palatal ridge is dorsoventrally thin, shallowly concave ventrally and extends to the midline, as indicated by a small fragment of the right palatal ridge remaining in place. Viewed anteriorly, the dorsal surface of the palatal ridge is W-shaped (Fig. 16B). The palatal shelf forms the ventral part of the slot-like medial groove that accommodates the premaxillary process of the maxilla and the anterior end of the vomera. The medial slot is loosely fitting around the premaxillary process of the maxilla forming a gap that suggests the original presence of thick cartilage in this region. The neurovascular tract from the maxilla enters the posterior end of the premaxillary dental ramus *via* two foramina (Figs. 13A–16A–16C, 16F).

**Figure 17:**
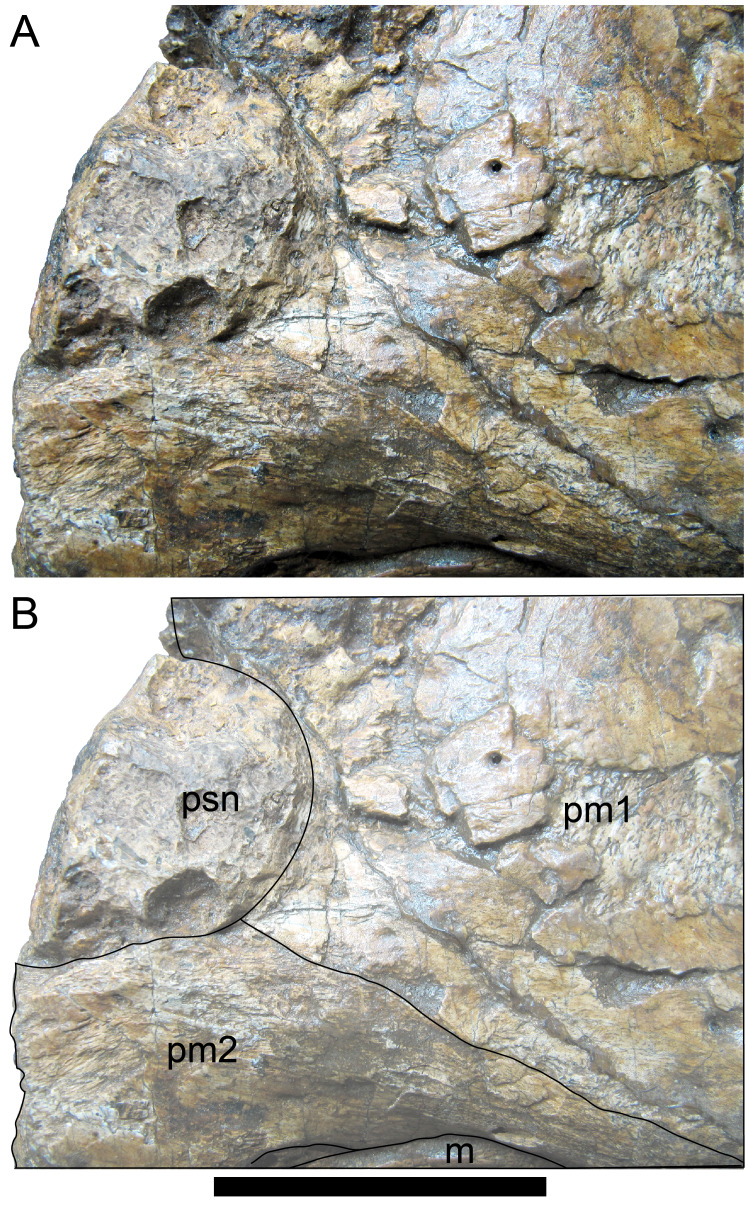
Photograph of *Muttaburrasaurus langdoni* (QMF6140) in region of the pseudonares (cranial part 2). (A, B) Pseudonarial region in (A) left lateral view and (B) explanatory schematic overlay. Abbreviations: m, maxilla; pm1, posterodorsal process of premaxilla; pm2, posteroventral process of premaxilla; psn, pseudonaris. Scale bar equals five cm.

Typically, in dinosaurs, the ascending process of the premaxilla extends dorsally from the premaxillary ramus at the anterior-most end of the premaxilla to contact the nasal, and the lateral process extends posteriorly from the ramus to wedge between the anterodorsal margin of the maxilla and the ventral margin of the nasal. The ascending and lateral processes of the premaxilla together with the nasal typically form the naris. In many early diverging ornithischians, the ascending processes of the paired premaxillae are ankylosed, but are normally unfused in more derived taxa (*e.g.*, Barta & Norell, 2021). Lambeosaurines present an exception to the typical morphology in dinosaurs. In lambeosaurines, complex, tightly abutting processes of the premaxilla extend posteriorly from the premaxillary ramus to form tubular crests. In addition, the nasal is also excluded from the naris (*e.g.*, Evans, 2006; Evans & Reisz, 2007; Evans, 2010; Prieto-Márquez & Wagner, 2013). As a result, the naris in lambeosaurines has been termed a “pseudonaris” (*sensu* Prieto-Márquez & Wagner, 2013). The ascending process is not preserved on the premaxilla of the *Muttaburrasaurus langdoni* holotype, or on the skull of *Muttaburrasaurus* sp. (QMF14921). However, processes on the left premaxilla of the holotype, posterior to the dental ramus, indicate derived morphology comparable to lambeosaurines. Close examination of the CT data and subsequent volume rendering indicate that the premaxilla developed complex posterodorsal and posteroventral processes and, as the nasals are excluded from the narial opening, a ‘pseudonaris’ is identified (Figs. 16, 17). Contrary to previous observations of a dorsally extending narial slot (Bartholomai & Molnar, 1981; Molnar, 1995; Molnar, 1996), the posterior margin of the pseudonarial opening is laterally placed (Figs. 16, 17) (see further under “Skull openings” below). How the posterodorsal and posteroventral processes of the premaxilla diverged anteriorly is presently unknown.

The terminology used here for the processes of the premaxilla in *Muttaburrasaurus langdoni* slightly differs from the terms previously used by some authors in lambeosaurines. The posterodorsal process herein, is equivalent to the “dorsal” process in Evans (2010) and “medial” process in Prieto-Márquez & Wagner (2013), and the posteroventral process herein, is equivalent to the “caudolateral” process in Evans & Reisz (2007) and “lateral” process in Evans (2010) and Prieto-Márquez & Wagner (2013). The posteroventral process is strap-like and forms a dorsally concave trough that floors the ventral region of the lateral sub-chamber of the nasal cavity complex (Figs. 8, 16). Viewed dorsally, the posteroventral process has an elliptical outline (Fig. 16). The ascending process of the maxilla accommodates the convex ventral surface and its tapered posterior tip contacts the lacrimal (see further under “Lacrimal” below). As apparent in the CT imagery, the posterodorsal process is mediolaterally thin, dorsoventrally broad, and forms a laterally concave sheet that tightly abuts the dorsal surface of the posteroventral process along a straight, roughly horizontal contact. Externally, the margin is visible as a distinct change in the bone texture between the two processes (Fig. 17). The external surface of the posterodorsal process is smoother than the posteroventral process. The posteroventral and posterodorsal processes form the concave posterior margin of the pseudonarial opening anteriorly on the muzzle block (cranial part 2) (Figs. 8C, 16, 17). Posteriorly, the posterodorsal process forms a dorsoventrally tall, wedge-shaped bony sheet between the anterolateral process of the nasal and the lateral margin of the prenasal ossification (see below; Figs. 8, 16A–16F). The medial surface of the posterodorsal process forms two thin processes or septa (Figs. 16A–16E). The first is a dorsally directed anteroventral process, which is continuous with the trough-like dorsal surface of the posteroventral process and encloses the anteroventral end of the superior airway; a function shared with the adjoining vertical septum of the prenasal ossification (Figs. 8, 16). The second process, termed herein, the posteromedial process, is posteriorly positioned and forms a medially projecting horizontal shelf, which is roughly semi-circular in dorsal view and tapers medially (Fig. 16). The medial margin of the transversely oriented posteromedial process contacts the horizontal septum of the prenasal ossification (Fig. 8) and together form a bridge that divides the anterior and posterior horizontal fenestrae between the inferior (main) and superior airways. A slot-like, dorsoventrally oriented duct passes through the thickened base of the posteromedial process (Fig. 16).

#### Premaxillary dentition

Although incomplete, the teeth are well developed (Figs. 12, 13, 15), as in all early diverging ornithischians, such as Heterodontosauridae (Sereno, 2012), early diverging neornithischians, such as *Changchunsaurus parvus* (Jin et al., 2010), *Haya griva* Makovicky et al., 2011 (Barta & Norell, 2021), *Jeholosaurus shangyuanensis* (Hu et al., 2024), *Orodromeus makelai* (Scheetz, 1999) and *Thescelosaurus neglectus* (Boyd, 2014), the early diverging ornithopods, *Convolosaurus marri* (Andrzejewski, Winkler & Jacobs, 2019) and *Hypsilophodon foxii* (Galton, 1974) and the early diverging iguanodontian *Iani smithi* (Zanno et al., 2023). In contrast to *Muttaburrasaurus langdoni* and the early diverging ornithischians, premaxillary teeth are vestigial in the early diverging iguanodontian, *Tenontosaurus dossi* (Winkler, Murry & Jacobs, 1997) and the elasmarian *Talenkauen santacrucensis* (Rozadilla, Agnolín & Novas, 2019) and their premaxillary teeth have yet to be described.

The roots of the functional crowns are preserved in place in the premaxillary dental rami but the crowns are missing (Figs. 12, 13, 15). The fully developed roots are apicobasally elongate, ‘bottle-shaped’ (*i.e.,* tapering apically; noting that the terms apical and basal used here for the root, are directionally opposite to apical and basal on the crown) and relatively uniform in size along the tooth row (Fig. 12). The largest root diameter is ∼13.0 mm. One germ (replacement) tooth is present in each tooth family (being “…the teeth which sequentially occupy a tooth position”; Osborn, 1972, p. 601). On the complete left side, germ teeth are present at premaxillary tooth positions (pmt)1, pmt4 and pmt5 (Figs. 12, 13A–13F). On the posterior right dental ramus fragment, a germ tooth is present at pmt5 (Figs. 15G–15I). Based on the relative degree of crown development at pmt4 and pmt5 on the left side, tooth replacement appears to have been posterior to anterior (Figs. 12A, 13E). An apical foramen is developed on the root. The root cross-section is egg-shaped, broader mesially than distally, but is round in the basal (ventral) third of the root (Fig. 13G). Notably, this egg-shaped cross-section of the root helps to identify the orientation and location of the dental ramus, such as the location of the highly fragmentary cranial part 13 at the anterior end of the left dental ramus (Fig. 11–Fig. 13). As indicated by the left functional tooth root, pmt5, the linguodistal margin of the root was resorbed as the germ tooth developed (Figs. 12A, 12B, 13A, 13E), as reported in *Changchunsaurus parvus* (Jun et al., 2018) and *Jeholosaurus shangyuanensis* (Hu et al., 2024). The functional crown would have been retained during resorption of its root and replaced soon after being shed, thereby maintaining (as far as possible) a full complement of the working crowns. On the apical (ventral) half of the functional tooth root left pmt4, a distinct slot is developed distally through to the root (Figs. 13I, 13J). This feature is not observed on any of the other roots. The germ crowns, best seen at the left pmt4 and right pmt5, are caniniform, slightly labiolingually compressed and recurved, both distally and lingually (Figs. 13D, 13E, 13H, 15H, 15I; see dimensions, Table 1). A distinct carina is present on the mesial edge of the germ crown and a weaker carina is developed distally (Figs. 12C, 13H, 13J, 15H, 15I). Denticles on the carinae are not apparent, although fine serrations could be present near the apex of the mesial carina on left pmt4. Denticles are similarly absent on the premaxillary tooth crowns of *Changchunsaurus parvus* (Jin et al., 2010); thus, differing from *Lesothosaurus diagnosticus* (Sereno, 1991; Thulborn, 1970), the early diverging ornithopods, *Convolosaurus marri* (Andrzejewski, Winkler & Jacobs, 2019) and *Hypsilophodon foxii* (Galton, 1974), and the rhabdodontomorph, *Iani smithi* (Zanno et al., 2023), where denticles are present. Labial ornamentation on the crown is absent. Unlike *Jeholosaurus shangyuanensis*, *Lesothosaurus diagnosticus* and *Thescelosaurus neglectus* (Boyd, 2014; Thulborn, 1970), an apicobasal groove is absent lingually adjacent to the distal carina. The enamel is evenly concentric and ∼140 µm thick (Fig. 13K). Constriction between the crown and root is virtually absent—a condition more closely resembling the heterodontosaurids, *Heterodontosaurus tucki* and *Abrictosaurus consors* (Sereno, 2012), than the early diverging neornithischians, *Changchunsaurus parvus* (Jin et al., 2010), *Convolosaurus marri* (Andrzejewski, Winkler & Jacobs, 2019), *Haya griva* Makovicky et al., 2011 (Barta & Norell, 2021; Norell & Barta, 2016), *Jeholosaurus shangyuanensis* (Barrett & Han, 2009; Hu et al., 2024), *Thescelosaurus neglectus* (Boyd, 2014) and *Zephyrosaurus schaffi* (Sues, 1980), the early diverging ornithopod, *Hypsilophodon foxii* (Galton, 1974) and the heterodontosaurid, *Echinodon becklesii* (Sereno, 2012), where the crowns distinctly bulge relative to the adjoining bases of their roots. The germ tooth at left pmt4, is at the advanced bell-stage within its dental follicle (Figs. 13D, 13H, 13K, 13L), indicating that the enamel and dentine were fully developed prior to root development, as reported in mammals (Huang & Chai, 2012; Huang et al., 2009). An apically concave margin in the matrix on the same developing tooth provisionally suggests the line of Hertwig’s epithelial root sheath diaphragm that forms the boundary of the pulp cavity containing the mesenchymal cells (Hertwig, 1874; Luan, Ito & Diekwisch, 2006). The passage of the neurovascular tract into the root apices (Fig. 13F), suggests the supply of nutrients to the developing tooth occurred through the apical foramen.

#### Prenasal

**Figure 18:**
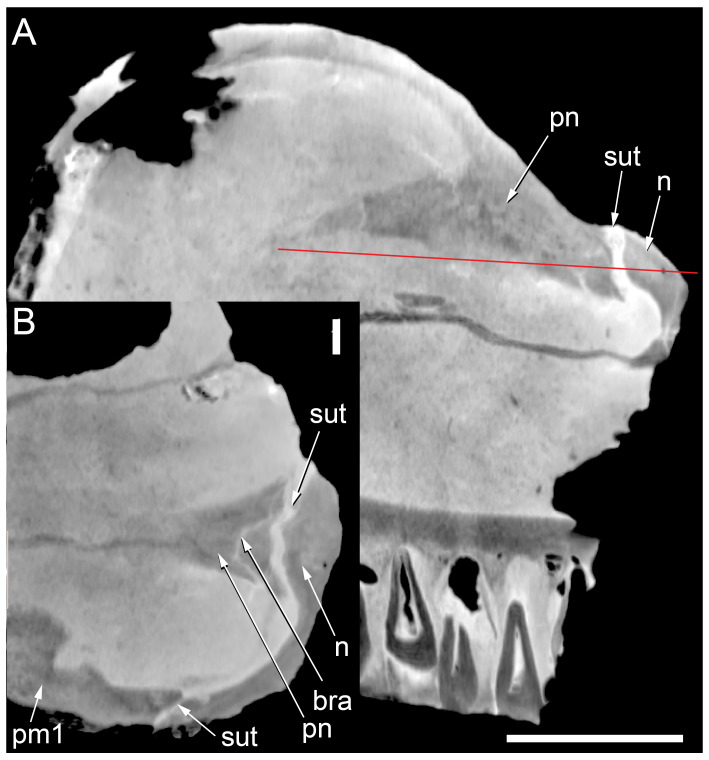
Radiographs of the *Muttaburrasaurus langdoni* holotype muzzle (cranial part 2) in the region of the left nasal and prenasal ossification. (A, B) Radiographic sections of muzzle in the (A) sagittal and (B) dorsal planes. Note, the darker material is fossil bone and lighter grey material is carbonate matrix. Abbreviations: bra, breakage; n, nasal; pm1, posterodorsal process of premaxilla; pn, prenasal; sut, suture. Red line in A indicates the dorsal plane in B. Scale bar A equals five cm and B equals one cm.

**Figure 19:**
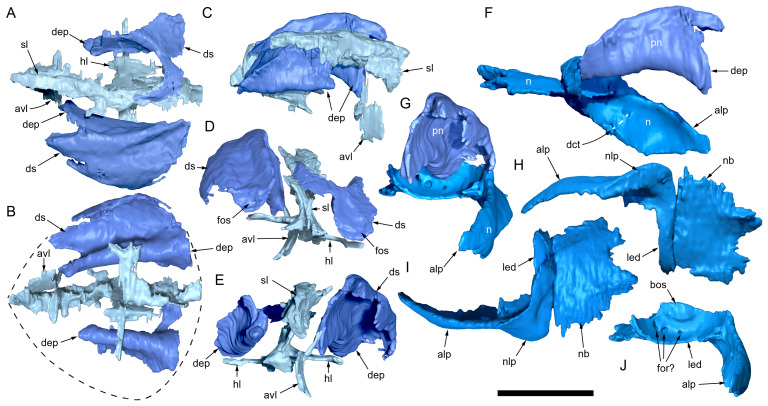
Volume rendered model of the *Muttaburrasaurus langdoni* (QMF6140) prenasal ossifications and left nasal. (A–E) Left and right prenasal ossifications in (A) dorsal, (B) ventral, (C) right lateral, (D) posterior and (E) anterior views (prenasal septal laminae in light blue). (F, G) Articulated left prenasal and nasal (note, prenasal septal lamina removed) in medial (F) and anterior (G) views. (H–J) Left nasal in (H) ventral, (I) dorsal and (J) anterior views. Dashed line in B suggests the dorsal profile of the complete prenasal. Abbreviations: alp, anterolateral process; avl, anteroventral septal lamina; bos, boss; dct, duct; dep, descending process; ds, dorsal sheet; for, foramen; fos, fossa; hl, horizontal septal lamina; led, ledge; n, nasal; nb, nasal body; nlp, lacrimal process of nasal; pn, prenasal; sl, sagittal/medial septal lamina. Scale bar equals five cm. MorphoSource DOI: 10.17602/M2/M787694; 10.17602/M2/M787691; 10.17602/M2/M787697; 10.17602/M2/M787700; (right nasal fragment not figured) 10.17602/M2/M787706.

Unusual paired ossifications, termed here prenasals, form the domed dorsal to posterodorsal portion of the muzzle (Figs. 8, 18 and 19). Each prenasal abuts the nasal posteriorly along a well*-* defined and slightly displaced sutural margin; clearly visible in the CT generated radiograph (Fig. 18A). Sutural margins are further evident between the anterolateral process of the nasal and the posterolateral process of the premaxilla (Fig. 18B) and between the dorsolateral edge of the prenasal and the posterodorsal process of the premaxilla. However, the anatomical relationship anteriorly between the premaxilla and prenasal ossification is presently unknown. As a result, separation between these regions as distinct ossification centres cannot be categorically demonstrated. Thus, the prenasal could constitute either a third, very complex, posterior process of the premaxilla (*i.e.,* in addition to the posteroventral and posterodorsal processes), or a neomorphic bone independent of the premaxilla. However, the occurrence of three distinct processes of the premaxilla extending posteriorly from the premaxillary ramus is unknown in any other dinosaur or tetrapod. For this reason, we consider the prenasal more likely to constitute a neomorphic bone, rather than an additional process of the premaxilla. Nevertheless, until further evidence comes to light, we regard the prenasal as an ossification centre of uncertain association.

The prenasal is complex. Dorsally, it consists of an anteroposteriorly elongate dorsal bony sheet that forms the posterodorsal portion of the dorsally inflated muzzle (Figs. 5A, 6B, 19A, 19B, 19D, 19E, 19G). However, the medial halves of the dorsal sheets on both prenasals are missing and the internal spaces in this region are dorsally exposed. These dorsal openings appear to result from damage and/or erosion of the thin original dorsal sheet, rather than having been naturally formed. When complete, the paired dorsal sheets, as proposed, would have been mediolaterally and anteroposteriorly arched and potentially delta-shaped in dorsoventral view (Fig. 19B), while noting that the anterior-most extent of the prenasals are unknown. As evident on the left side, furrow extends anteroposteriorly on the lateral half of the dorsal surface. On each prenasal, a thin bony septum, termed here the prenasal septum, extends ventrally from the medial edge of the dorsal sheet (Figs. 8, 19A–19E). A fragment of the domed dorsal sheet preserved on the right side confirms the connection of the vertical septum and dorsal sheet of the prenasal (Figs. 8A, 19A, 19B, 19D). The paired medial septa descend into and divide the superior airway into left and right meatuses. Each septum sharply angles laterally at its base forming a discontinuous, horizontal component. Thus, the complete prenasal septum forms an L-shaped cross-section (Figs. 19D, 19E). Close examination of the CT imagery indicates that the paired vertical components of the prenasal septa are closely adjoining medial elements, as opposed to being a single sagittal element. However, fusion of the paired septa is possible in some places. The vertical components of the prenasal septa are incomplete, which could be due in part to breakage or poor CT resolution of the thin bone. Gaps in the vertical septa could also indicate the original existence of cartilage *in vivo*. The horizontal septum forms a narrow horizontal bridge that connects and slightly underlaps the transversely oriented posteromedial process of the premaxilla (Figs. 8A, 8B, 19A, 19B, 19D, 19E). In addition, the L-shaped posterior ends of the paired, abutting prenasal septa slot between the anteromedial corners of the paired nasals (Figs. 8B, 8C).

A distinct, anteroposteriorly oriented descending process extends ventrally into the superior airway from the ventral surface of the dorsal sheet, lateral to the eroded dorsal openings (Figs. 8, 19A–19G). The process is pendulous, thickening ventrally. The ventral margin of the descending process is concave in mediolateral view (Figs. 8C, 19F). Viewed ventrally, the descending process expands posteriorly and recurves medially in the anterior direction (Figs. 8B, 19A, 19B). The posterior end forms a fossa that accommodates a boss on the nasal (Figs. 18, 19D, 19J). The anteroventral septal lamina extends from the anterior end of the descending process to form a thin, roughly vertical septum that adjoins the pocket-like anteroventral process on the posterodorsal process of the premaxilla (Figs. 8C, 16, 19A–19E). Notably, the inside corner of the L-shaped prenasal septum forms a smoothly curved channel that conforms to the curved space surrounding the descending process (Fig. 19E), as well as the smooth toroidal channel formed on the anterior face of the nasal body (see further under “Nasal” below).

Taken together, the anteroventral septal lamina of the prenasal, the horizontal bridge of the prenasal septum, the transversely oriented posteromedial process of the premaxilla and contribution of the internal horizontal ledge of the nasal (see “Nasal” below), separate the *cavum nasi proprium* into superior and inferior airways (see further in “Results and Discussion”). However, the inferior and superior airways are connected *via* enlarged fenestra. As evident on the left side, two fenestrae (divided by the septal bridge) pass between the inferior and superior airways (Fig. 8B). The posterior fenestra is elliptical in shape. The anterior fenestra is more extensive than the posterior; however, its shape is difficult to establish with certainty, possibly owing to damage of the horizontal septum or the original existence of cartilage.

#### Nasal

**Figure 20:**
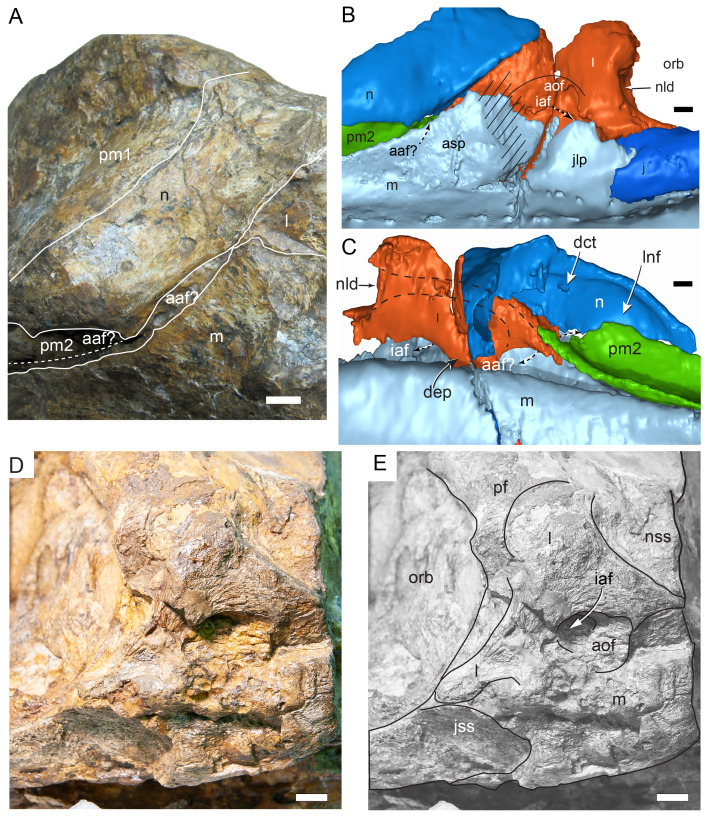
Photographs and volume rendered model of the *Muttaburrasaurus langdoni* (QMF6140) antorbital region. (A) Photograph in region of the left accessory antorbital fenestra in lateral view with schemetic overlay of bone margin contacts. (B, C) Volume rendered model of the left antorbital region in (B) lateral and (C) medial views. (D, E) Photograph (D) of right antorbital region in lateral view and (E) explanatory line overlay. Dotted line in A indicates possible line of breakage on the nasal. Dashed arrows in B and C indicate openings between bones. Solid line in B indicates dorsal margin of the external antorbital fenestra (based on the right side). Cross-hatching in B indicates probable area of bone loss anterior to the antorbital fossa. Dashed lines in C indicate internal path of the nasolacrimal duct. Abbreviations: aaf, accessory antorbital fenestra; aof, antorbital fossa and external fenestra; asp, ascending process of maxilla; dct, duct; dep, descending process of lacrimal; iaf, internal antorbital fenestra; j, jugal; jlp, jugolacrimal process of maxilla; jss, jugal sutural surface and jugal fragment; l, lacrimal; lnf, lateral nasal fossa; m, maxilla; n, nasal; nss, nasal sutural surface; orb, orbital; pf, prefrontal; pm1, posterodorsal process of premaxilla; pm2, posteroventral process of premaxilla. Scale bars equal one cm.

The left nasal is almost complete but only a small medial portion of the right nasal is preserved (Figs. 6B, 19F–19J). The posterior portion of the better-preserved left nasal body is eroded and differentiation of its ventral surface from the CT imagery is unclear. In addition, the left nasal body has been taphonomically compacted ventrally towards the midline (along with all the right region of the muzzle), resulting in its distorted appearance (see Fig. 20). The body of the nasal is plate-like and roughly quadrangular in dorsoventral profile and an elongate, sheet-like, anterolateral process extends from the nasal body (Figs. 19F–19J). Notably, the anterolateral process was originally considered as the anterior process of the prefrontal (Bartholomai & Molnar, 1981). The posterior margin of the nasal body forms an M-shaped notch for the frontal and the lateral margin of the nasal body ventrally underlies the medial margin of the prefrontal (Figs. 6B, 19; see further under “Prefrontal” and “Frontal” below). CT data clearly show that the nasal and prenasal ossification are separate bones (Fig. 18; see also “Prenasal” above). The anterior region of the nasal body forms the posterior wall of the superior airway (Figs. 8, Fig. 19). A mediolaterally broad fossa at the anterior end of the nasal body forms a toroidal shaped channel, at the centre of which a domed boss is formed that inserts into a fossa at the posterior end of the descending process of the prenasal (Figs. 18, 19J). The ventral base of the fossa forms the posterior margin of the posterior fenestra between the inferior and superior airways. Three foramina appear to pass through the posterior wall of the nasal into the toroidal fossa (Figs. 19G, 19J). However, some or all these openings could have resulted from erosion at the posterior end of the muzzle block (cranial part 2). Continuation of these ‘foramina’ could not be detected on the mating part of the left nasal on cranial part 1. However, preservation in this region is poor. The sheet-like anterolateral process forms a bulbous, lateral nasal protuberance (Figs. 5–8, 19F–19J, 20). The dorsal margin of the lateral process abuts the posterodorsal process of the premaxilla and the ventral margin abuts the maxilla and lacrimal (Figs. 5A, 7A, 20A). The sutural margins between these four elements are identified in the CT data. In addition, the anterolateral process laterally overlaps the posteroventral process of the premaxilla (Figs. 5A, 20). The ventral-most portion of the anterolateral process appears to be broken and missing, exposing the posteroventral process of the premaxilla in this region (Figs. 20A, 20B). Separation between the anterolateral process and the maxilla is possibly an accessory antorbital fenestra (Fig. 20) (see Witmer, 1997a), although this idenification is uncertain. Internally on the anterolateral process, a vertical duct is present midway along the medial wall (Fig. 19F).

#### Maxilla

**Figure 21:**
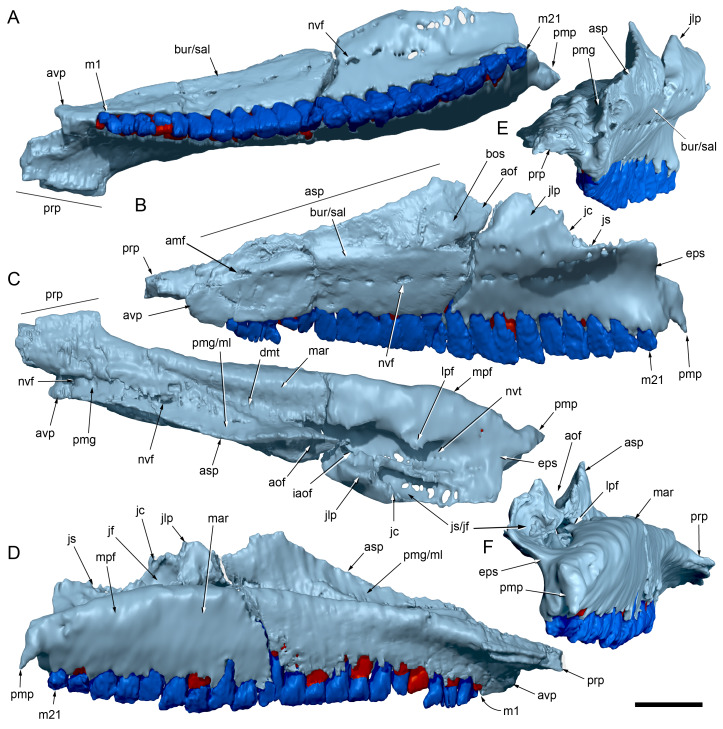
Volume rendered model of the *Muttaburrasaurus langdoni* (QMF6140) left maxilla. (A–F) Left maxilla in (A) ventral, (B) lateral, (C) dorsal, (D) medial, (E) anterior and (F) posterior views. Functional teeth shown blue; germ teeth shown red. Abbreviations: amf, maxillary foramen; aof, antorbital fossa; asp, ascending process; avp, anteroventral process; bos, boss; bur, buccal ridge; dmt, dorsal maxillary trough; eps, ectopterygoid shelf; iaof, internal antorbital fenestra; jc jugal crista; jf, jugal fossa; jlp jugolacrimal process; js, jugal shelf; lpf, lateral palatine flange; m#, maxillary tooth position/family; mar, maxillary ramus; ml, medial lamina; mpf, medial palatine facet; nvf, neurovascular foramen; nvt neurovascular tract; pmg, premaxillary groove (channel); pmp, posteromedial process; prp, premaxillary process ; sal supralveolar lamina. Scale bar equals five cm. MorphoSource DOI: 10.17602/M2/M787646; 10.17602/M2/M787655; 10.17602/M2/M787661; 10.17602/M2/M787649; 10.17602/M2/M787652; 10.17602/M2/M787658.

**Figure 22:**
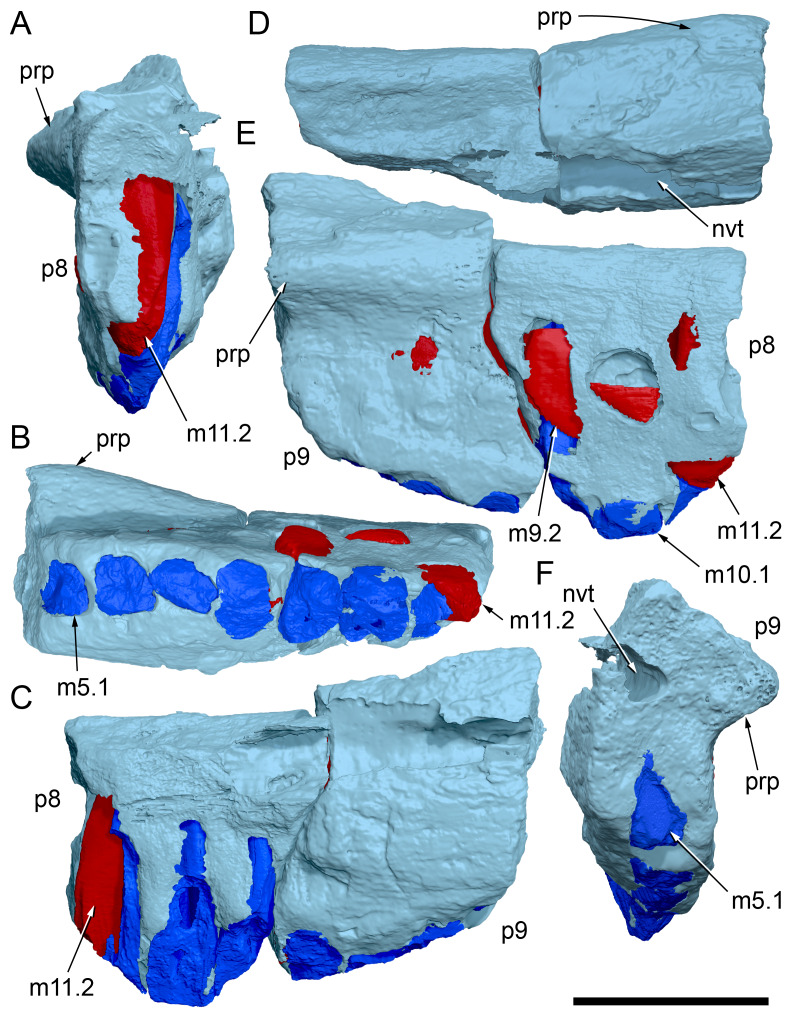
Volume rendered model of *Muttaburrasaurus langdoni* (QMF6140) anterior maxillary dental ramus fragments (cranial parts 8, 9). (A–F) Right maxillary dental ramus in (A) posterior, (B) ventral, (C) lateral, (D) dorsal, (E) medial and (F) anterior views. Abbreviations: m#, maxillary tooth position/family and development number (. 1 = functional tooth [blue]; .2 = germ tooth [red]); nvt, neurovascular tract; p#, cranial part and number; prp, premaxillary process. Scale bar equals five cm. MorphoSource DOI: 10.17602/M2/M786911; 10.17602/M2/M786915; 10.17602/M2/M786919; 10.17602/M2/M771412; 10.17602/M2/M786868; 10.17602/M2/M786871.

Complete on the left side and incomplete on the right, the maxilla is formed from the dental ramus, the supralveolar lamina (= facial lamina), the medial lamina and the premaxillary process (Fig. 21). The ascending process could be formed from convergence of the supralveolar and medial laminae (based on Herne et al., 2019), but differentiation of these laminae is not observed. The description is based mainly on the left maxilla; however, the previously undescribed fragment of the right dental ramus (cranial part 8) and the newly discovered right dental ramus fragment (cranial part 9) provide additional information (Fig. 22). These new parts locate on the muzzle block (cranial part 2). Viewed mediolaterally (Figs. 21B, 21D), the maxilla forms a scalene triangle with the peak of the ascending process positioned approximately midway on the dental ramus (see measurements Table 1). Viewed dorsoventrally (Figs. 21A, 21C), the maxilla is wedge shaped, broader posteriorly and narrower anteriorly, posterior to the premaxillary process. The left dental ramus contains 21 alveoli, the anteroposterior axis of which forms a continuous, laterally concave/medially convex) arc in ventral view (Fig. 21A). Ten posterior-most alveoli are present on the right maxillary dental ramus preserved on cranial part 1, which, based upon the left side, are maxillary tooth positions (m#) m12–21. Alveoli on the right maxillary dental ramus fragments (cranial parts 8 and 9) are provisionally identified as m5–m11 (Fig. 22). The anteroventral process is a small, rounded nub, laterally offset from the premaxillary process by a sulcus. The anteroventral process forms a short diastema (slightly longer than the length of the anterior-most alveolus), anteriorly to the first alveolus (m1) and forms a step, upon which, the premaxilla abuts (Figs. 21A, 21B, 21C).

**Figure 23:**
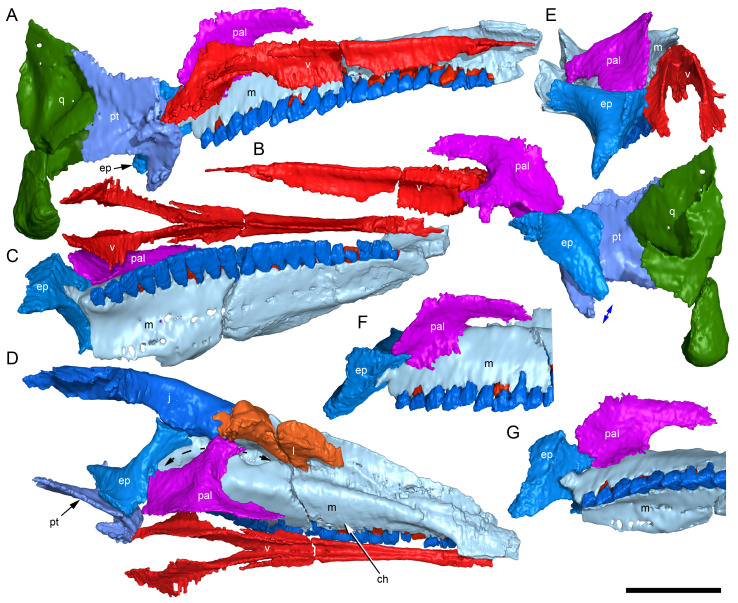
Volume rendered models of the *Muttaburrasaurus langdoni* (QMF6140) cheek, suspensorium and palatal regions in articulation. (A–G) Suspensorium and palatal regions in (A) medial (with pterygoid and quadrate vertically realigned), (B) lateral (with pterygoid and quadrate vertically realigned; blue arrow indicates pterygoid/ectopterygoid displacement), (C) ventral, (D) dorsal, (E) posterior, (F) medial and (G) ventral views. Functional teeth shown blue and germ teeth shown red. Dashed line in D indicates the path of the neurovascular tract at the posterior end of the maxilla (the anterior-most arrowhead indicates entry/exit of the neurovascular tract at the posterior neurovascular foramen). Abbreviations: ch, region of choana; ep, ectopterygoid; j, jugal; l, lacrimal; m, maxilla; pal, palatine; pt, pterygoid; q, quadrate; v, vomer/vomera. Scale bar equals 10 cm.

The anteromedially located premaxillary process forms a dorsoventrally compressed, medially expanded flange that is rectangular (anteroposteriorly elongate) in dorsoventral view (Figs. 21A, 21C), although the distal (anterior) tip is missing due to erosion (cranial part 2; Fig. 3). A V-shaped groove is developed along the entire medial face of the premaxillary process (Figs. 21D, 21F). The premaxillary process is ventrally positioned along the main axis of the maxilla, unlike the dorsally elevated position in non-lambeosaurine styracosternans, such as, *Iguanodon bernissartensis* (Norman, 1980), *Prosaurolophus blackfeetensis* (Horner, 1992) and *Edmontosaurus regalis* (Heaton, 1972; Lambe, 1920; Takasaki et al., 2020), where the premaxillary process inserts more dorsally between the premaxillae (*e.g.*, Horner, 1992, plate 25). A premaxillary process is not developed in lambeosaurines (*e.g.*, Heaton, 1972; Horner, Weishampel & Forster, 2004). The shape of the premaxillary process resembles that of *Dysalotosaurus lettowvorbecki* (Janensch, 1955), *Galleonosaurus dorisae* Herne et al., 2019, *Talenkauen santacrucensis* (Rozadilla, Agnolín & Novas, 2019) and *Zalmoxes robustus* (Weishampel et al., 2003) (M. Herne, pers. obs., 2009), but in these taxa, the processes lack the transversely broad, flange-like form in *Muttaburrasaurus langdoni*. Furthermore, the premaxillary process is less developed medially in *Camptosaurus dispar* (YPM VP 1886; Herne et al., 2019, fig. 9), *Hypsilophodon foxii* (NHMUK R2477; (Galton, 1974, fig. 5C; M. Herne, pers. obs., 2009) and *Tenontosaurus tilletti* (Thomas, 2015) than in *Muttaburrasaurus langdoni*. The anterior end of the fused vomera lies ventral to the anteroventral process (Fig. 23), as in *Thescelosaurus neglectus* (Boyd, 2014) and not inserted between them, as in *Hypsilophodon foxii* (Galton, 1974) and *Tenontosaurus tilletti* (Thomas, 2015).

Viewed laterally (Fig. 21B), the ascending process has a dorsally concave anterior edge, sloping 17° relative to the alveolar margin. A similarly shallow angle occurs in styracosternans (*e.g.*, *Mantellisaurus atherfieldensis* (Norman, 1986); *Ouranosaurus nigeriensis* (Taquet, 1976)). A trough for the posteroventral process of the premaxilla is present on the medial surface of the ascending process (Figs. 21C, 21D, 21E). The trough is narrow, anteriorly broadening in its middle part and further posteriorly, twisting more vertically towards the lacrimal after which it narrows and pinches out. A large, rounded foramen connecting the neurovascular tract penetrates the narrow anterior part of the trough (Fig. 21C). A further slot-like foramen penetrates the middle region of the trough.

The dorsal tips of the ascending and jugolacrimal processes extensively overlap the lacrimal laterally (Figs. 21B, 21C, 21D, 23). In addition, the supralveolar lamina walls the ventral portion of the antorbital fossa (again, overlapping the lacrimal laterally). The posterior end of the ascending process deflects medially into the antorbital fossa (Figs. 20A, 20B, 21B, 21C, 23). As a result, the anterior margin of the external antorbital fenestra is not sharply bordered by the supralveolar lamina and the antorbital fossa lacks anterolateral enclosure by the ascending process (Fig. 20B). This morphology is confirmed on the right side (Fig. 20B), indicating that the maxillary surface leading anteriorly into the antorbital fossa is sloping (confirmed by the CT imagery) and that the supralveolar lamina does not enclose the fossa (see further under “Lacrimal” and “Skull openings” below). A thickened boss on the supralveolar lamina occurs at the anterior entrance of the antorbital fossa (Fig. 21B). Anteriorly, the buccal ridge is dorsoventrally rounded and merges with the jugal shelf posteriorly (Figs. 21A, 21B, 21E). Ventral to the buccal ridge, a series of ∼24 neurovascular foramina (see further below) penetrate the supralveolar lamina. The buccal ridge converges with and terminates anteriorly at the anterior-most foramen of the neurovascular tract (Fig. 21B).

Articulation with the jugal is complex. The level of the laterally projecting jugal shelf is aligned with the buccal ridge. The shelf is mediolaterally broad and mediolaterally concave and pierced by a series of neurovascular foramina, as in *Zalmoxes robustus* Weishampel et al., 2003 (Figs. 21A, 21B, 21C, 21F, 21E). Anterior to the jugal shelf, the maxilla ascends to form the jugolacrimal process, which has a deep fossa on its medial side and a vertical crista posteriorly that inserts in a groove in the jugal (Figs. 21B, 21C, 21D; see also “Jugal” below). Thus, tongue-and-groove articulation with the jugal is apparent. The jugal fossa accommodates the anteromedial process of the jugal and the jugal shelf supports the ventral margin of the jugal (Figs. 21B, 21C). Ventrally, the jugal forms a fossa that deeply overhangs the dentition. The jugomaxillary connection differs from the comparatively simple abutting, scarfed or ventrally slotted connections in most other ornithopods (*e.g.*, *Hypsilophodon foxii* (Galton, 1974); *Iguanodon bernissartensis* (Norman, 1980); Styracosterna (Norman, 1980; McDonald et al., 2012b; Xing, Mallon & Currie, 2017); the Victorian ornithopods, *Atlascopcosaurus loadsi*, *Leaellynasaura amicagraphica* (Herne et al., 2019) and *Galleonosaurus dorisae* Herne et al., 2019; the rhabdodontid, *Zalmoxes robustus* Weishampel et al., 2003). Complex articulation between the maxilla and jugal appears like that reported in *Ouranosaurus nigeriensis* Taquet, 1976, although a detailed revision of this feature is needed in that taxon. *Muttaburrasaurus langdoni* lacks the freely-projecting, finger-like jugal process on the maxilla reported in styracosternans, such as *Altirhinus kurzanovi* (Norman, 1998), *Iguanodon bernissartensis* (Norman, 1980) and possibly *Ouranosaurus nigeriensis* (Taquet, 1976, fig. 17).

Ventral to the jugal shelf, the posterolateral margin of the maxilla is dorsoventrally deep and viewed anteroposteriorly, the margin is laterally concave (Figs. 21B, 21E). A posterolateral margin of similar appearance occurs in *Zalmoxes robustus* Weishampel et al., 2003 (BMNH R.4901; M. Herne, pers. obs., 2009), *Camptosaurus dispar* (Gilmore, 1909) and *Ouranosaurus nigerensis* (Taquet, 1976, fig. 17a). Viewed dorsoventrally, the posterior profile of the maxilla is transversely broad, unlike the posterior ends in most styracosternans, where the lateral and medial margins are posteriorly convergent (*e.g.*, *Iguanodon bernissartensis* (Norman, 1980); *Edmontosaurus regalis* (Xing, Mallon & Currie, 2017)). The posterior end of the maxilla in *Ouranosaurus nigeriensis* (Taquet, 1976, fig. 17b) appears to resemble that of *Muttaburrasaurus langdoni*. The ectopterygoid attaches to the posterior shelf on the maxilla (Figs. 21B, 21C, 23A, 23C–21G), as in non-styracosternan ornithopods, such as *Camptosaurus dispar* (YPM VP1886; based on images in Herne et al., 2019), *Hypsilophodon foxii* (Galton, 1974), dryosaurids (Galton, 1983), rhabdodontids (Weishampel et al., 2003; Zanno et al., 2023) and the Victorian ornithopods, *Atlascopcosaurus loadsi*, *Leaellynasaura amicagraphica* and *Galleonosaurus dorisae* Herne et al., 2019. A prong-like ectopterygoid process projects from the posteromedial corner of the dental ramus and is accommodated in a fossa on the ectopterygoid (Figs. 21B, 21C, 23A, 23C–23G; see also under “Ectopterygoid” below). A similar ectopterygoid process occurs in *Camptosaurus dispar* (Herne et al., 2019) and *Galleonosaurus dorisae* (Herne et al., 2019), *Tenontosaurus tilletti* (Thomas, 2015) and *Zalmoxes robustus* Weishampel et al., 2003, although in the latter taxon, the process is thickened and more robust than prong-like (BMNH R.4901; M. Herne, pers. obs., 2009). A posteromedial ectopterygoid process similar to that in *Muttaburrasaurus langdoni* is apparent in *Ouranosaurus nigeriensis* (Taquet, 1976, fig. 17b) but typically absent in Styracosterna, where the ectopterygoid wraps around the posterior end of the maxilla and attaches to the lateral surface and the pterygoid attaches posteriorly on the maxilla (*e.g.*, *Iguanodon bernissartensis* (Norman, 1986); hadrosaurids (Heaton, 1972; Horner, 1992; Lambe, 1920; Takasaki et al., 2020; Xing, Mallon & Currie, 2017)). However, the nature of the contact is uncertain in *Ouranosaurus nigeriensis*, although the presence of an ectopterygoid process posteromedially on the maxilla, as in *Muttaburrasaurus langdoni*, suggests a similar form of contact. How the ectopterygoid attached to the maxilla in the Argentinian elasmarians, *Anabisetia saldiviai*, *Gasparinisaura cincosaltensis* and *Talenkauen santacrusensis*, is uncertain.

**Figure 24:**
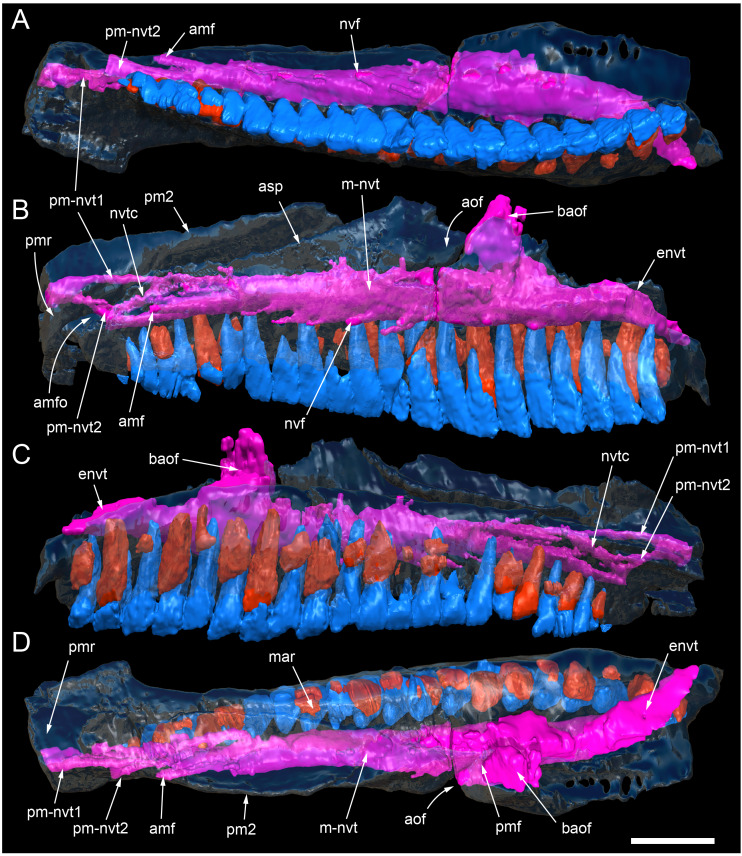
Volume rendered model of the *Muttaburrasaurus langdoni* (QMF6140) left premaxillary and maxillary neurovascular tract endocast. (A–D) Left neurovascular tract (coloured purple) in (A) ventral, (B) lateral, (C) medial and (D) dorsal views. Functional teeth shown blue, germ teeth shown red and neurovascular tract in magenta. Abbreviations: amf, anterior maxillary foramen; amfo, anterior maxillary fossa; aof, antorbital fossa; asp, ascending process; baof, branch of endocast connecting neurovascular tract to antorbital fossa through internal antorbital fenestra; envt, dorsally exposed channel of neurovascular tract; mar, maxillary dental ramus; nvf, neurovascular foramen; nvtc, cross-branch of neurovascular tract; m-nvt, neurovascular tract of maxilla; pm2, posteroventral process of premaxilla; pmf, posterior maxillary foramen; pm-nvt1, first branch of neurovascular tract to premaxilla; pm-nvt2, second branch of neurovascular tract to premaxilla; pmr, premaxillary (dental) ramus. Scale bar equals five cm. MorphoSource DOI: 10.17602/M2/M787712; 10.17602/M2/M787646; 10.17602/M2/M787655; 10.17602/M2/M787661; 10.17602/M2/M787649; 10.17602/M2/M787652; 10.17602/M2/M787658.

The palatine body contacts the medial palatine facet near the posterior end of the dental ramus, anterior to the ectopterygoid (Figs. 21C, 21D, 23A, 23C–23G). The lateral ramus of the palatine traverses the dorsal surface of the dental ramus and is supported at its distal end by a tab-like flange of the maxilla that partly projects above the dorsally open region of the neurovascular tract (see further below; Figs. 21C, 21F, 23D, 23E, 24), as in the Australian small-bodied ornithopods, *Atlascopcosaurus loadsi*, *Galleonosaurus dorisae* (Herne et al., 2019) and *Leaellynasaura amicagraphica* and possibly in *Camptosaurus dispar* (Herne et al., 2019). *Muttaburrasaurus langdoni* lacks the dorsally elevated process/pedestal for the palatine, occurring on the maxilla of *Hypsilophodon foxii* (Herne et al., 2019) and the maxillae of hadrosaurids (*e.g.*, Heaton, 1972; Horner, 1992; Xing, Mallon & Currie, 2017). A pedestal for the palatine is also absent in the Australian small-bodied ornithopods, as mentioned, as well as *Camptosaurus dispar* (Herne et al., 2019) and most likely *Dysalotosaurus lettowvorbecki* (based on Janensch, 1955; Galton, 1983).

**Figure 25:**
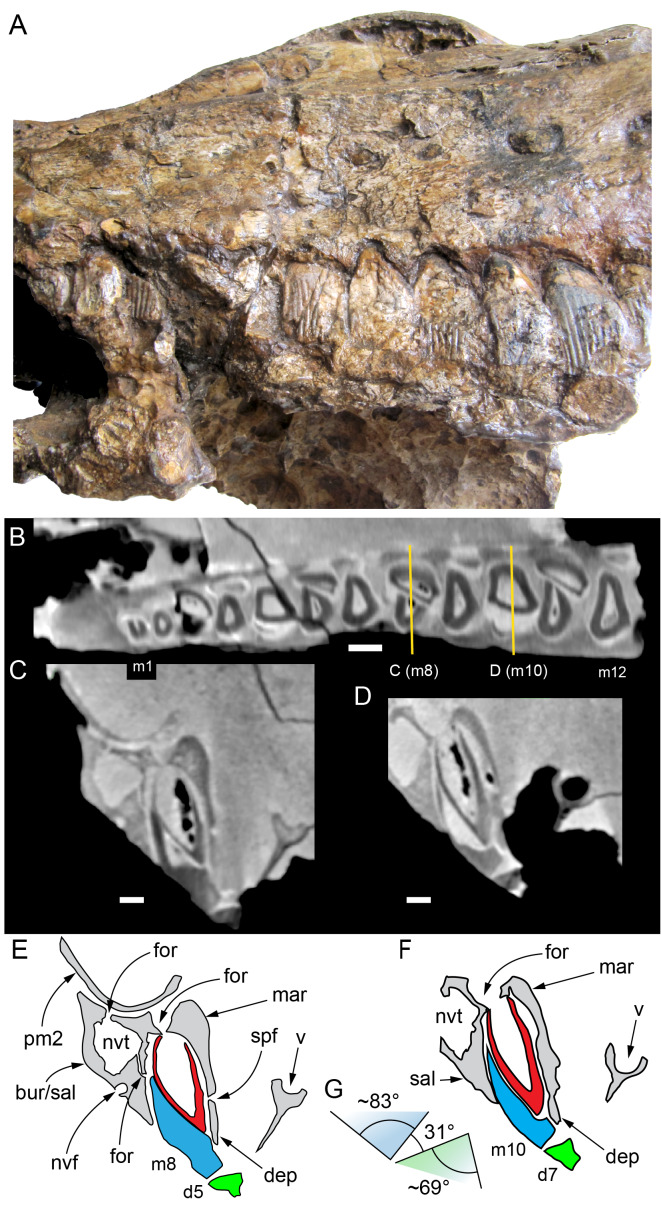
Photograph, radiographs and tooth occlusal angles for the *Muttaburrasaurus langdoni* (QMF6140) anterior left cheek dentition (cranial part 2). (A) Photograph of dentition in left lateroventral view, showing the functional maxillary tooth crown arcade from m1–m12. (B) Dorsal radiograph through roots and developing crowns from m1–m12. Yellow lines in B indicate transverse sections in C and E. (C, D) Transverse radiographs at (C) alveolar positions m8 and d5 and (D) alveolar positions m10 and d7. (E, F) Explanatory schematics for C and D. (G) Transverse schematic section of functional cheek tooth crown showing occlusal angles at m10 and d7 and angle of postmortem dentary rotation indicated by dislocation of the occlusal surfaces. Red shade in E and F, developing maxillary tooth crowns, blue shade in E, F and G, indicating functional maxillary tooth crowns and green shade in E, F and G, indicating apical part of functional dentary tooth crowns. Abbreviations: bur, buccal ridge; d#, dentary tooth position; dep, dental parapet; for, foramen; m#, maxillary alveolus/tooth position; mar, maxillary (dental) ramus; nvf, neurovascular foramen; nvt, neurovascular tract; pm2, posteroventral process of premaxilla; sal, supralveolar lamina; spf, ‘special’ (nutrient) foramen; v, vomera. Scale bars equal one cm.

The neurovascular tract (Fig. 24) carried the maxillary branch of the trigeminal nerve (cn V). In the posterior region of the maxilla, and medial to the lacrimal, the neurovascular tract presents as a dorsally open channel (Figs. 21C, 23D, 24). In this region, the neurovascular tract is partly roofed by the flange for the lateral ramus of the palatine, as well as the lateral-most end of the lateral ramus of the palatine (Figs. 21C, 23D). The neurovascular tract connects the antorbital fossa through a dorsal fenestra that opens into the internal antorbital fenestra between the lateral flange for the palatine and the posterior neurovascular tract foramen (Figs. 23D, 24B, 24C). The neurovascular tract is internalised within the maxilla anteriorly from the posterior neurovascular tract foramen, which is partly roofed dorsally by the descending process of the lacrimal (Figs. 20C, 21C, 22D, 23D, 24, 25C, 25D). Ventrolaterally oriented branches from the neurovascular tract open laterally in a line of approximately 12 neurovascular foramina ventral to the buccal ridge (Figs. 21A, 21B). Anteriorly, the neurovascular tract exits the maxilla in three branches (Fig. 24). The lateral branch exits through the anterolaterally oriented maxillary foramen located at the anterior end of the buccal ridge (Figs. 21B, 24A, 24B, 24D), and two branches exit in the elongate slot for the premaxilla (Figs. 21B, 24B–24D). These two latter branches pass into the dental ramus of the premaxilla (Fig. 24). The two neurovascular tract branches for the premaxilla are further linked by an internal cross-branch (Figs. 24B, 24C). Small additional branches exit dorsally from the neurovascular tract into the dorsal maxillary groove (Fig. 25C) and further small branches appear to enter the alveoli, but do not connect the apical root foramina (Fig. 25C). Apical foramina of the roots penetrate the dorsal maxillary trough (Figs. 25C, 25D; noting these are not clearly shown in the volume rendered models), suggesting that the course of the neurovascular root canal extends along the dorsal maxillary trough, including in the region between the trough and the posteroventral process of the premaxilla. Nutrient foramina, known as “special foramina” (Edmund, 1957), penetrate the thin (∼3 mm) medial alveolar wall (dental parapet) corresponding to the developing crowns, as in other ornithischians (Figs. 25C, 25D).

#### Maxillary dentition

**Figure 26:**
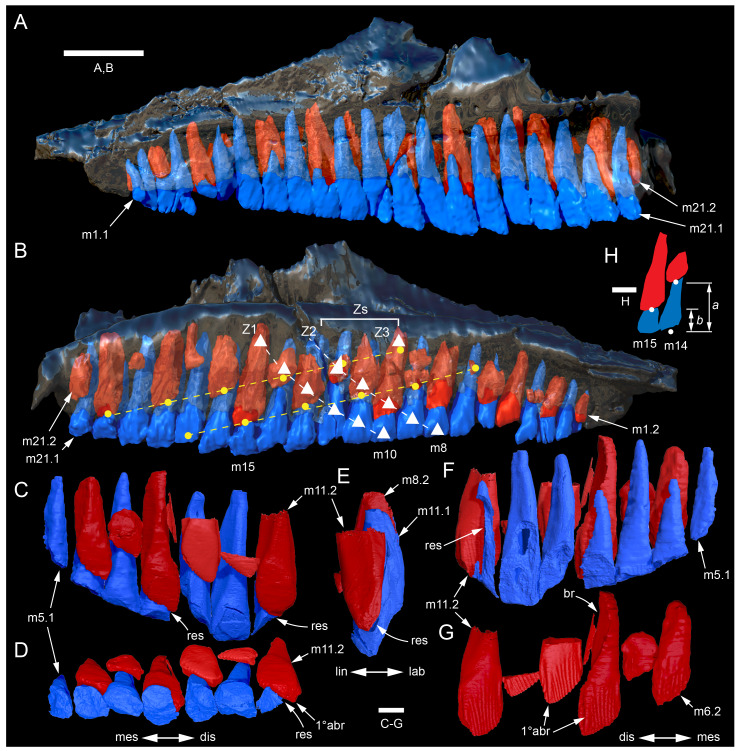
Volume rendered models of the *Muttaburrasaurus langdoni* (QMF6140) maxillary dentition. (A, B) Left maxillary dentition in (A) lateral/labial and (B) medial/lingual views with bone of the maxilla digitally clarified. (C–F) Right maxillary dentition (cranial parts 8, 9) in (C) lingual, (D) apical, (E) distal and (F) labial views (bone of the maxilla removed. (G) Germ teeth of the right maxilla (cranial parts 8, 9) in labial view. (H) Method of calculating Zahnreihe (Z) spacing based on Osborn (1974): Z = *a*/*b*, where “*b* = time taken between eruption of a tooth and that posteriorly adjacent to it, and *a* = time taken to replace a tooth [in the tooth family]” (example taken at m14 and m15 with white dots shown indicate the points of reference; note that ‘time’ is dimensionless until a numerical rate value is applied). (H–L) Right maxillary germ tooth m7.2 in (H) distal, (I) labial, (J) mesial, (K) lingual and (L) crown apical views. Yellow dashed lines with dots in B indicate posterior to anterior replacement waves across alternate tooth families (two only shown) (following (Edmund, 1960; Osborn, 1977)). White dashed lines with triangles in (B) and marked with Z1–Z3 indicate Zahnreihen extending across adjacent tooth families (based on information in (Edmund, 1960)). Z1 is a complete Zahnreihe commencing at tooth position m10.1 and ending at an expected point of initiation of a new germ tooth at m15. Z2 is the Zahnreihe including m10.2. Z3 is the expected point of initiation of a new germ tooth at m10 (continuation of the Zahnreihe anterior to this point not shown). White bracket in B marked with “Zs” indicates Z spacing (∼2.5 and <3) (based on (Edmund, 1960)). Abbreviations: 1° abr, primary apicobasal ridge; 2° abr, secondary apicobasal ridge; br, broken surface; cin, cingulum; cv, cingular vertex; dbr, distal bounding ridge; dis, distal direction; dpr, distal paracingular fossa; gc, growth channel; lab, labial; lin, lingual; m#, maxillary tooth position/family and development number (.1 = functional tooth [blue]; .2 = germ tooth [red]); mbr, mesial bounding ridge; mes, mesial direction; pcv, pulp cavity endocast; res, margin of resorption. Scale bar A, B equals five cm, G equals two cm and C–F, H–L equals one cm. MorphoSource DOI: 10.17602/M2/M787646; 10.17602/M2/M787655; 10.17602/M2/M787661; 10.17602/M2/M787649; 10.17602/M2/M787652; 10.17602/M2/M787658; 10.17602/M2/M786915; 10.17602/M2/M78691; 10.17602/M2/M786868; 10.17602/M2/M786871.

**Figure 27:**
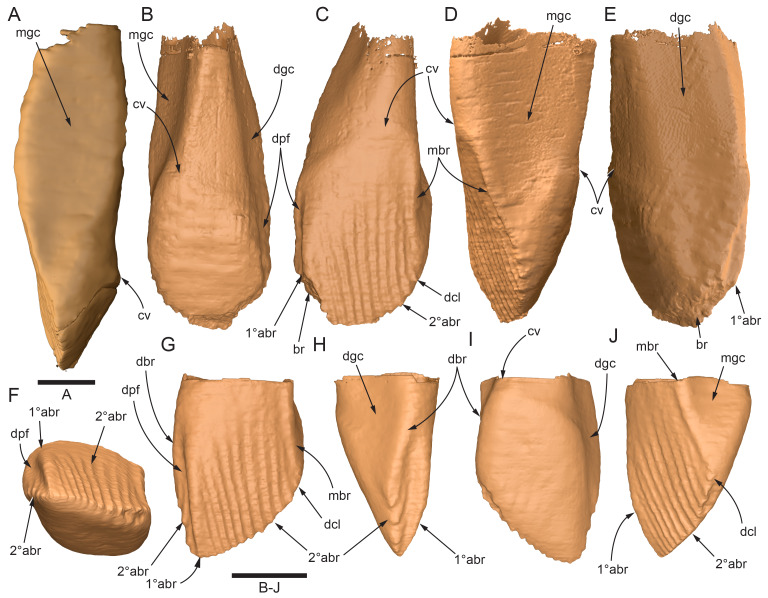
Volume rendered models of the *Muttaburrasaurus langdoni* (QMF6140) right maxillary dentition. (A) Right maxillary germ tooth m8. 2 (cranial part 9) in mesial view. (B–E) Right maxillary germ tooth m11.2 (cranial part 8) in (B) lingual, (C) labial, (D) mesial and (E) distal views. (F–J) Right maxillary germ tooth m9.2 (cranial part 8) in (F) apical, (G) labial, (H) distal, (I) lingual and (J) mesial views. Abbreviations: 1° abr, primary ridge; 2° abr, secondary apicobasal ridge; br, breakage; cv, cingular vertex; dbr, distal bounding ridge; dgc, distal growth channel; dpf, distal paracingular fossa; dcl, denticle; mbr, mesial bounding ridge; mgc, mesial growth channel. Scale bars equal one cm. MorphoSource DOI: 10.17602/M2/M786915; 10.17602/M2/M786868.

Functional maxillary teeth are exposed labially on the left side of the main skull blocks (*i.e.,* cranial parts 1 and 2; Figs. 5, 21, 25A). However, all the functional crowns on these parts are damaged and/or eroded to some degree making their morphologies difficult to assess with certainty. In addition, medical CT imagery of the main skull blocks lacked enough resolution to define the fine ornamentation of the maxillary tooth crowns (Figs. 26A, 26B). However, detailed visualization of the tooth morphology was enabled by higher resolution micro-CT imagery (see “Methods”) and volume rendered models from the newly discovered right maxillary dental ramus fragments (cranial parts 8 and 9), which preserve roots of the functional teeth and the germ crowns (Figs. 22, 26C–26F, 27). Thus, in the following description of the maxillary dentition, the gross morphology of the dental growth pattern is provided by the dentition of left maxilla (*i.e.,* cranial parts 1 and 2). However, from the teeth in the right dental ramus fragments (cranial parts 8 and 9), detailed morphologies of the crowns and roots are provided.

One working tooth and one replacement tooth occur at each alveolus (Figs. 25B–25C, 26). The root is straight in mesiodistal view and has a mesiodistally compressed, roughly lunate-triangular cross section, with the mesial side depressed and the distal side ridged (Figs. 25B, 27A). The mesial and linguodistal surfaces of the root and onto the crown base are depressed forming growth channels (Fig. 27). At mid-height along the root, the root bulges labiolingually and, at this point, is labiolingually broader than the crown (Figs. 26E, 27A). Labiolingual swelling of the maxillary tooth root is shared with *Galleonosaurus dorisae* Herne et al., 2019, noting that torsion of the root in the latter taxon is not present in *Muttaburrasaurus langdoni*. Labiolingual swelling of the root differs from the narrow, elongate roots in iguanodontians, such as *Iguanodon bernissartensis* (Norman, 1980), *Rhabdodon* sp. (Chanthasit, 2010), *Tenontosaurus tilletti* (Thomas, 2015) and *Zalmoxes robustus* Weishampel et al., 2003. In addition to the narrow maxillary tooth roots in the above iguanodontians, the roots of Styracosterna differ from those of *Muttaburrasaurus langdoni* in being to some extent curved (*e.g.*, *Mantellisaurus atherfieldensis* (Norman, 1986); *Ouranosaurus nigeriensis* (Sereno, 2012); *Brachylophosaurus canadensis* (Prieto-Márquez, 2001); hadrosaurids in general (LeBlanc et al., 2016))—likely linked to close packing of the diamond-to-lanceolate shaped crowns, which, in hadrosaurids, is a distinctive feature of their dental batteries. As in *Muttaburrasaurus langdoni*, the roots in at least some early diverging ornithischians are apicobasally (dorsoventrally) straight in mesiodistal view (*sensu* Boyd, 2014). Differing from *Muttaburrasaurus langdoni* the maxillary tooth root of the heterodontosaurid, *Tianyulong confuciusi*, is tapered and unswollen, whereas the root is slightly swollen, labiolingually, in *Heterodontosaurus tucki* (Sereno, 2012). The roots of the adjacent tooth families are closely packed (Figs. 25B, 26), as in other ornithopods, and differing from early diverging neornithischians, such as *Thescelosaurus neglectus* (Boyd, 2014), where the roots are more widely separated. An apical foramen is present on the developing roots (Figs. 25C, 25D).

A posterior-to-anterior wave of tooth replacement across alternating tooth families is apparent (yellow dashed lines and dots in Fig. 26B), as typically occurs in reptiles including dinosaurs (Edmund, 1960; Osborn, 1974). A single replacement wave appears to extend over 10 tooth families (Fig. 26B). The overlapping waves, in concert with the anterior to posterior Zahnreihen eruption pattern (white dashed lines and triangles in Fig. 26B), ensured that the duration of gaps between the working teeth was minimal (Edmund, 1960). The relative rate of tooth replacement is found by Zahnreihe (Z) spacing, mapped on the sequence of tooth families (Fig. 26B) (see Edmund, 1960; Edmund, 1969; Osborn, 1977). In the mid-region of the tooth row, a Zahnreihe replacement sequence extends across six adjacent tooth families, when the last shed tooth is included in the count, whereas in the anterior and posterior regions of the dentition, replacement appears to occur over five tooth families. Z spacing was calculated for several tooth positions (Table 1) using the method outlined by Osborn (1974), which calculates the rate of replacement based on the distances between erupting teeth at adjacent tooth families (see further details in Fig. 26B and caption). Using this method, a Zahnreihe spacing of 1.8–2.3 is estimated, with a mean of approximately 2. The method, outlined by Edmund (1960), suggests a Z spacing of ∼2.5–3.0 (see in Fig. 26B, and caption). The rate of maxillary tooth replacement in *Muttaburrasaurus langdoni*, suggested from the Z spacing (∼2.5), is consistent with other ornithischians (based on Edmund, 1960). The rate of replacement in terms of days was not assessed.

Description of the maxillary tooth crown morphology and ornamentation is taken from visual observations of the functional crowns exposed on the left side of the muzzle block (cranial part 2; Fig. 25A) and the volume rendered right germ crowns m8, m9 and m11 on cranial parts 8 and 9 (Figs. 26H–26L). The crown is spatulate (*i.e.,* viewed labiolingually, the apical profile of the unworn crown is mesially and distally convex and the basal profile is V-shaped; following (Herne et al., 2019, fig. 3)) and is strongly imbricated, with the distal margin labially overlapping the mesial margin of the succeeding crown (Figs. 21A, 25B, 26A, 26B; crown dimensions, Table 1). Labially, the cingulum (forming the crown base) merges with the root, whereas lingually, a slight step is formed from the cingulum to the root (Fig. 27). A narrow, finger-like, mesial bounding ridge is present (Fig. 27). A primary ridge on the maxillary crowns of the holotype was originally thought to be absent (“carina” in Bartholomai & Molnar, 1981; Molnar, 1996). Furthermore, a primary ridge is not evident on the exposed, eroded, maxillary crowns on the holotype muzzle (see Fig. 25A). However, from the CT imagery, a primary ridge is clearly present on the right maxillary germ crowns (Figs. 26D, 26G, 27), as on the crowns of *Muttaburrasaurus* sp. (QMF14921) (Herne et al., 2019).

The crown is strongly asymmetrical, resulting from strong distal offset of the labial primary ridge and mesial offset of the labial and lingual cingular vertices (Figs. 27B, 27C, 27I). Labially, the primary ridge is low, apicobasally straight and labially rounded (Figs. 27C, 27F, 27G). The primary ridge separates the mesial and distal paracingular fossae (Figs. 27C, 27F–27H). The distal paracingular fossa slightly undercuts the primary ridge. Labially on right m9, 11 secondary ridges are developed in the mesial paracingular fossa, mesial to the primary ridge (Figs. 27F, 27G, 27J). However, the mesial-most ridge is short and virtually just a denticle. A similar number of secondary apicobasal ridges may be present on right m8 and m11; however, breakage on the apical edges of these crowns prevents a conclusive count of the ridges (Figs. 26G, 27C). The number of secondary apicobasal ridges on the other maxillary crowns is presently uncertain. The five distal-most, mesial secondary ridges on right m8, m9 and m11 are obliquely angled relative to the primary ridge and convergent with the latter towards their bases (Figs. 27C, 27F, 27G). The ridges mesial to the five mentioned are slightly arcuate (mesially concave) along their length and in this aspect, more vertically oriented. The three mesial-most secondary ridges are convergent with the mesial bounding ridge towards their bases. Three or possibly four, secondary apicobasal ridges are evident in the narrow distal paracingular fossa of right m9 (Figs. 27F–27H). The distal bounding ridge, only observed on right m9, is poorly developed and lacks any degree of labial protrusion (Figs. 27G, 27H). Nevertheless, the distal bounding ridge forms a distinct border between the distal paracingular fossa and the distal growth channel (“dpf” and “dgc” in Fig. 27). Lingually, the crown surface is smooth without secondary ridges (Figs. 26C, 27B). In transverse section (Figs. 25C, 25D), the angle between the labial and lingual faces of the germ crowns is acute (∼56° ). However, the working occlusal surfaces on the functional crowns (measured at m7, m10, m14 on the left side) form a blunt angle with a mean of ∼73° relative to the labial crown surface and ∼40° to the horizontal plane (Figs. 25C, 25D); noting that identification of the horizontal plane (as previously used by some workers to comparatively assess the occlusal angle between the cheek teeth of other ornithischian taxa; *e.g.*, Weishampel, 1984) on the left maxilla is uncertain, potentially owing to postmortem rotation.

During germ crown growth, the primary ridge inserts between the roots of the mesially, and distally adjacent functional teeth and resorption of the functional tooth root is on the mesial side of the primary ridge of the growing germ tooth crown (Figs. 26C–26F). Thus, growth of the germ tooth is distally offset in its alveolus with respect to the developed working tooth at the same tooth position. This pattern contrasts with maxillary tooth growth in the dental batteries of hadrosaurids, where the centrally positioned primary ridge of the successive crown grows into a channel on the lingual surface of the earlier developed tooth (LeBlanc et al., 2016).

Labially, distal offset of the primary ridge and closely abutting secondary apicobasal ridges converging mesially with the mesial bounding ridge without separation by a channel, most resemble the crowns of taxa assigned to Elasmaria from Victoria, *Atlascopcosaurus loadsi*, *Galleonosaurus dorisae* and *Leaellynasaura amicagraphica* (Duncan et al., 2021; Herne et al., 2019) and Argentina, *Gasparinisaura cincosaltensis* Coria & Salgado, 1996 (MUCPv-208; M. Herne, pers. obs., 2008) and *Talenkauen santacrucensis* (Cambiaso, 2007; Rozadilla, Agnolín & Novas, 2019). As in *Muttaburrasaurus langdoni* and Elasmaria, the primary ridge is strongly offset distally on the maxillary crowns of the early diverging ankylopollexians *Camptosaurus dispar* (Galton, 2009, fig. 18O) and *Cumnoria prestwichii* (Maidment et al., 2022, fig. 10) but differs from *Muttaburrasaurus langdoni* in lacking a mesially offset cingular vertex and closely abutting secondary ridges. The crowns of *Camptosaurus dispar* and *Cumnoria prestwichii* further differ from *Muttaburrasaurus langdoni* in possessing unsupported apical denticles and a smooth channel separating the secondary ridges and the mesial bounding ridge. Furthermore, the secondary ridges on the crowns of *Camptosaurus dispar* and *Cumnoria prestwichii* run parallel to the primary ridge and are separated from it by a channel, whereas the secondary ridges closest to the primary ridge are convergent in *Muttaburrasaurus langdoni*; thus, closely resembling the condition in Elasmaria (Herne et al., 2019). Strong labial offset of the cingular vertex on the crowns of *Muttaburrasaurus langdoni* is shared with *Muttaburrasaurus* sp. (QMF14921) (Herne et al., 2019, fig. 17). Differing from *Muttaburrasaurus langdoni*, the secondary ridges are more strongly developed in the distal paracingular fossa of *Muttaburrasaurus* sp. (QMF14921), as in many of the small-bodied Victorian ornithopods (Herne et al., 2019, fig. 17) and the Argentinian taxa *Gasparinisaura cincosaltensis* (MUCPv-208; M. Herne, pers. obs., 2008) and *Talenkauen santacrucensis* (Cambiaso, 2007, fig. 17B; Rozadilla, Agnolín & Novas, 2019). In addition, the primary ridge is more strongly developed in *Muttaburrasaurus* sp. (QMF14921) than the relatively weak primary ridge on the *M. langdoni* holotype. Notably, apart from their differences in size and the number of secondary ridges, the crowns of *Muttaburrasaurus langdoni*, *Muttaburrasaurus* sp. (QMF14921) and the Victorian small-bodied ornithopod, *Atlascopcosaurus loadsi* (NMV P157390) are morphologically similar (Herne et al., 2019). The number of secondary ridges on the maxillary crowns of *Muttaburrasaurus langdoni* (up to 11 mesial to the primary ridge and 3 or 4 distal to the primary ridge) is lower than the number in rhabdodontids of up to 18–20 ridges (Maidment et al., 2022), noting, also, that the crowns of the latter clade lack a primary ridge and are relatively symmetrical (*e.g.*, Zalmoxes; Weishampel et al., 2003; M. Herne, pers. obs., 2009).

#### Lacrimal

**Figure 28:**
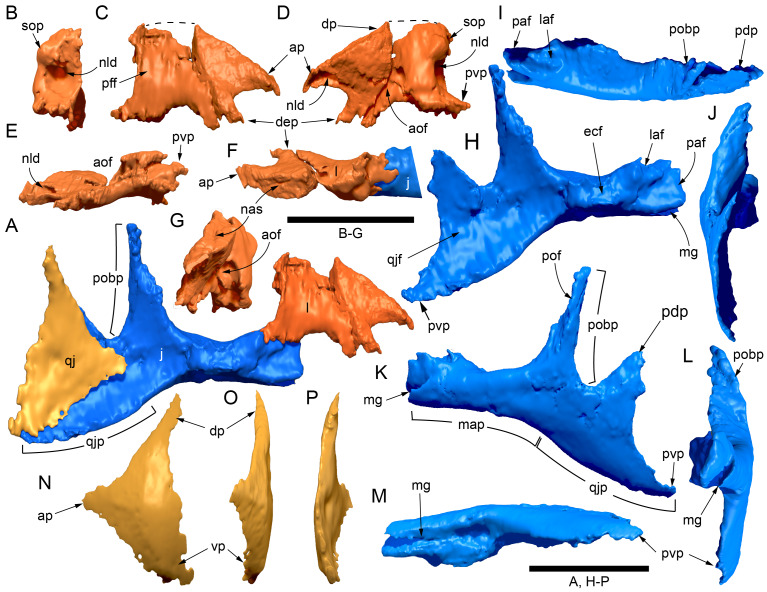
Volume rendered models of the *Muttaburrasaurus langdoni* (QMF6140) bones of the left cheek. (A) Left lacrimal, jugal and quadratojugal articulated in medial view. (B–G) Left lacrimal in (B) posterior, (C) medial, (D) lateral, (E) ventral, (F) dorsal adjoining jugal (blue) and (G) anterior views. (H–I) Left jugal in (H) medial, (I) dorsal, (J) posterior, (K) lateral, (L) anterior and (M) ventral views. (N–P) Left quadratojugal in (N) lateral, (O) anterior and (P) posterior views (dashed line indicates missing bone). Abbreviations: aof, antorbital fossa; ap, anterior process; dep, descending process; dp, dorsal process; ecf, ectopterygoid facet; j, jugal; l, lacrimal; laf, lacrimal facet; map, maxillary (anterior) process; mg, maxillary groove; nas, nasal suture; nld, nasolacrimal duct; paf, palatine facet; pdp, posterodorsal process; pff, prefrontal facet; pobp, postorbital process; pof, postorbital facet; pvp, posteroventral process; qj, quadratojugal; qjf, quadratojugal facet; qjp, quadratojugal process; sop, supraorbital protuberance; vp, ventral process. Scale bars equal 10 cm. MorphoSource DOI: 10.17602/M2/M787721; 10.17602/M2/M787718; 10.17602/M2/M787734; 10.17602/M2/M787743; 10.17602/M2/M787715.

The left lacrimal is virtually complete, although transversely divided along the break between cranial parts 1 and 2, and the dorsal and some of the lateral regions are also eroded (Figs. 5A, 28A–28F). Only the dorsal region of the right lacrimal is preserved. In articulation with the surrounding cranial elements, the lacrimal is triangular in lateral view (Figs. 28C, 28D). It consists of the thickened body centrally, from which the jugal process extends posteriorly (= pedical, Thomas, 2015), the premaxillary process anteriorly and the descending process ventrally (= anteroventral process; Thomas, 2015) (Figs. 28A–28G). The lacrimal forms the posterodorsal portion of the antorbital fossa and external antorbital fenestra (Figs. 20B, 20D, 28D, 28E, 28G). The dorsal margin of the antorbital fossa, best preserved on the right side, indicates an elliptical profile (Figs. 20B, 20D; see further in “Skull openings” below). Ventrolaterally, the lacrimal is overlapped by the ascending and jugolacrimal processes of the maxilla, including within the antorbital fossa (Fig. 20B). Viewed laterally, contact with the premaxilla appears to be prevented by nasomaxillary contact (Fig. 20B). However, viewed internally, the posteroventral process of the lacrimal forms a flange that contacts and underlaps the tapered posterior end of the posteroventral process of the premaxilla (Fig. 20C). The descending process (which is obscured by the maxilla when articulated) is anteroventrally oriented and, unusually, inserts into the dorsal opening of the maxillary neurovascular tract (Figs. 23D, 20C, 28C, 28D, 28F). Posteroventral contact with the jugal is convoluted (Figs. 28A, 28F). The jugal process is formed from three sub-processes that interdigitate with the jugal. The nasolacrimal duct passes internally within the body of the lacrimal, from its transversely broad orbital opening to the ventrally directed opening within the nasal cavity, anterior to the descending process, and onto the dorsal maxillary trough (Figs. 20B, 20C, 28D). The sloping anterodorsal surface, upon which the nasal abuts, is continuous with the dorsal margin of the ascending process on the maxilla. The dorsal tip of the lacrimal contacts the prefrontal (Fig. 5A). A domed lateral boss occurs on the orbital margin, which potentially formed the surface of attachment for the palpebral (Figs. 5A, 28A, 28D), as in *Ouranosaurus nigeriensis* Taquet, 1976. However, the palpebral in *Muttaburrasaurus langdoni* is presently unknown.

The extensive descending process, which inserts into the dorsal opening of the neurovascular tract of the maxilla, is unknown in any other ornithopod from the descriptions available. Nevertheless, the overall form of the lacrimal resembles that of *Dysalotosaurus lettowvorbecki* (Galton, 1983, fig. 3); particularly in the anteroventrally oriented anterior opening of the nasolacrimal duct and anteroventrally oriented descending process ventral to the opening of the nasolacrimal duct. Among ornithopods, nasomaxillary contact preventing lacrimopremaxillary contact occurs in less derived ornithopods, such as *Convolosaurus marri* Andrzejewski, Winkler & Jacobs, 2019, *Hypsilophodon foxii* (Galton, 1974) and was likely absent in *Zalmoxes robustus* Weishampel et al., 2003. Lacrimopremaxillary contact is variable among early diverging neornithischians. Contact is prevented by nasomaxillary contact in *Haya griva* Makovicky et al., 2011 and *Thescelosaurus neglectus* (Boyd, 2014) but present in some specimens of *Jeholosaurus shangyuanensis* (Barrett & Han, 2009). Lacrimopremaxillary contact is uncertain in *Tenontosaurus tilletti* (Thomas, 2015). Among ornithopods, nasopremaxillary contact in *Muttaburrasaurus langdoni* suggests closer affinities with derived ornithopods at the level of Dryomorpha (= Ankylopollexia and Dryosauridae; Sereno, 1986) (Galton, 1983; Thomas, 2015) than with early diverging ornithopods, where the two bones are separated by the nasal.

#### Jugal

**Figure 29:**
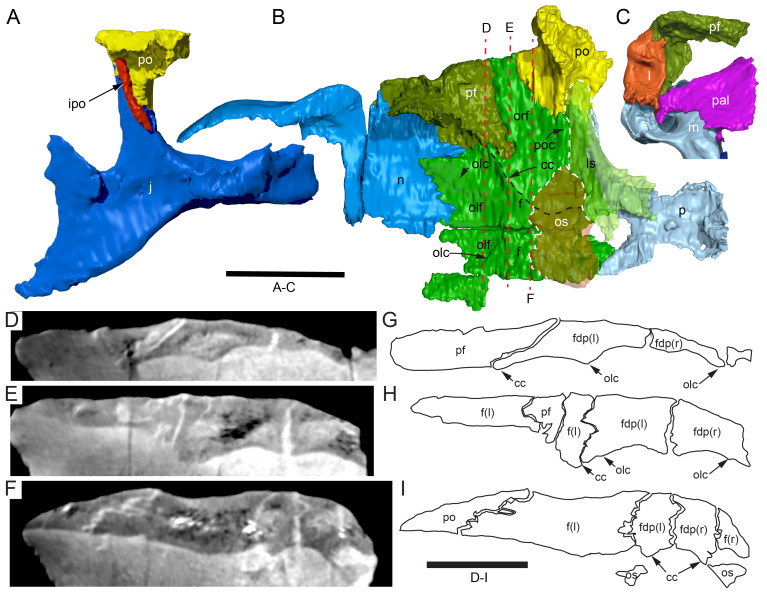
Volume rendered models and CT radiographs of the *Muttaburrasaurus langdoni* (QMF6140) maxillofacial and cranial roof regions. (A) Left jugal, postorbital and intrapostorbital in medial view. (B) Paired nasals and frontals, left prefrontal, postorbital and laterosphenoid, orbitosphenoid and fused parietals in ventral view. Dashed lines in B indicate margins of the orbitosphenoid and right laterosphenoid (shown semi-transparent). (C) Left maxilla, lacrimal, palatine and prefrontal in posterior view. (D–F) Transverse radiographs through the cranial roof showing dorsal plate margins of the frontals and (G–I) explanatory schematics. Radiographic sections in D–F correspond to red dashed lines in B. Abbreviations: cc, *crista cranii*; f, frontal (l, left; r, right); fdp, dorsal plate of frontal (l, left; r, right); ipo, intrapostorbital (intrasutural element); j, jugal; l, lacrimal; ls, laterosphenoid (translucent overlay); m, maxilla; n, nasal; olc, olfactory crista; olf, olfactory fossa; orf, orbital fossa; os, orbitosphenoid (translucent overlay); p, parietal; pal, palatine; pf, prefrontal; po, postorbital; poc, posterior orbital crista. Scale bar A–C equals 10 cm and D–I equals five cm. MorphoSource DOI: left intrapostorbital, 10.17602/M2/M788131.

The jugal is formed of a central body from which project the maxillary (anterior), postorbital (dorsal) and quadratojugal (posterior) processes (Figs. 28A, 28I–28M; Table 1). Viewed dorsoventrally, the jugal is laterally convex and medially concave. Viewed posteriorly, it is partially cylindrical (laterally convex/medially concave). Viewed mediolaterally, the ventral margin is strongly sinuous. The anterior end of the thickened maxillary process forms a complex double tongue in groove joint with the maxilla (Figs. 28H, 28K, 28L, 28M). Cristae and grooves on the dorsal surface of the maxillary process further interdigitate with the lacrimal (Figs. 28A, 28F). Medially on the maxillary process, a facet is present for the lateral process of the palatine and a horizontal ridge forms a sutural facet for the ectopterygoid (Figs. 23D, 28I, 28H). The dorsally tapering postorbital process is angled at ∼100° relative to the maxillary process (Figs. 28H, 28K). A wedge-shaped facet at approximately mid-height on the anterolateral edge of the postorbital process accommodates the postorbital. More dorsally, a fossa on the anterior surface of the postorbital process accommodates an unusual, dorsoventrally elongate, supernumerary, intrasutural ossification (Fig. 29A). The triangular, sheet-like quadratojugal process is strongly deflected ventrally (Figs. 28A, 28H, 28K, 28L). The posterior margin is concave. The posteroventral end of the quadratojugal process forms a spur that ventrally subtends a slot on the quadratojugal. The posterodorsal corner of the quadratojugal process is triangular and together with the quadratojugal forms the V-shaped posteroventral margin of the infratemporal fenestra (Figs. 5A, 28A, 28H).

The interlocking form of connection between the jugal and maxilla appears like that reported in *Ouranosaurus nigeriensis* Taquet, 1976. Marked ventral deflection of the ventral margin of the jugal is typically present across a range of non-hadrosaurid iguanodontians (Norman, 2004) and a spur-like posteroventral corner of the quadratojugal process is shared with the basal styracosternans, *Hippodraco scutidens* (McDonald et al., 2010b) and *Theiophytalia kerri* (Brill & Carpenter, 2006) and the rhabdodontid *Zalmoxes robustus* Weishampel et al., 2003. However, the combination of a spur-like posteroventral corner and dorsally spinose quadratojugal process is shared with *Theiophytalia kerri*. This region of the jugal is presently unknown in *Camptosaurus dispar*.

#### Quadratojugal

The quadratojugal is sheet-like, forming an isosceles triangle in mediolateral view (Figs. 28A, 28N–28P). The lateral surface forms a shallow dome, with the anteroventral and anterodorsal faces overlapped laterally by the adjoining jugal. The medial surface is shallowly concave—the posterior portion of which overlaps the quadrate (Figs. 5A, 6B, 7B). The posterior margin is slightly sinuous in mediolateral aspect (Figs. 28A, 28N). The dorsal process is spinose where it extends towards the squamosal; however, as the distal (dorsal) end is eroded, the nature of contact with the squamosal is unknown. Unlike *Hypsilophodon foxii* (Galton, 1974) and *Tenontosaurus tilletti* (Thomas, 2015) no foramina penetrate the bone. The large size of the quadratojugal is consistent with non-styracosternan ornithopods. The quadratojugal is relatively reduced in Styracosterna (Norman, 2004). However, the overall shape of the quadratojugal resembles that of *Ouranosaurus nigeriensis* (Taquet, 1976), which, relative to other Styracosterna, is enlarged.

#### Quadrate

**Figure 30:**
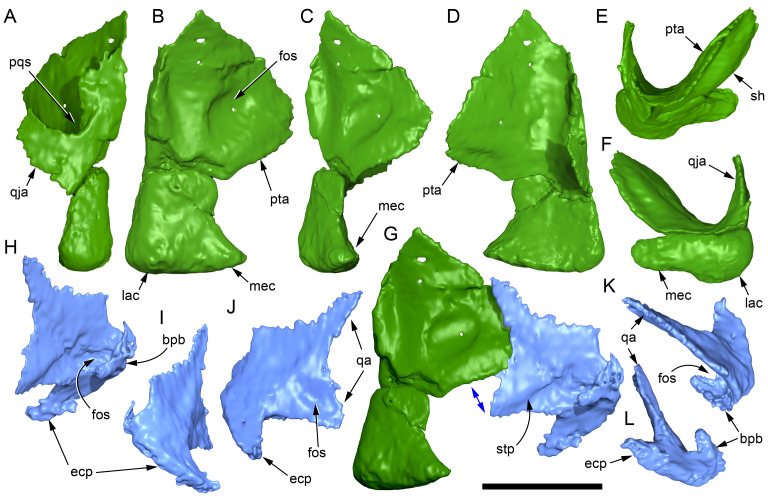
Volume rendered models of the *Muttaburrasaurus langdoni* (QMF6140) left quadrate and pterygoid. (A–F) Left quadrate in (A) lateral, (B) posteromedial, (C) medial, (D) anteromedial, (E) dorsal and (F) ventral views. (G) Articulated left quadrate and pterygoid in posteromedial view (arrow indicates postmortem displacement). (H–L) Left pterygoid in (H) posteromedial, (I) anterior, (J) lateral, (K) dorsal and (L) ventral views. Abbreviations: bpb, basipterygoid boss; ecp, ectopterygoid process; fos, fossa; lac, lateral condyle; mec, medial condyle; pqs, paraquadratic sulcus; qa, quadrate ala; qja, quadratojugal ala; pta, pterygoid ala; sh, shelf; stp, step. Scale bar equals 10 cm. MorphoSource DOI: 10.17602/M2/M788110; 10.17602/M2/M787756; right pterygoid (not figured), 10.17602/M2/M787749.

An element of the splanchnocranium, the quadrate consists of the dorsoventrally elongate column, from which projects the quadratojugal and pterygoid alae (Figs. 30A–30G; Table 1). The right quadrate is absent, and the dorsal portion of the left quadrate is missing. Ventrally, the mandibular condyle consists of lateral and medial moieties (Figs. 30B, 30F) with the medial anteroposteriorly narrower than the lateral. Viewed laterally, the ventral portion of the quadratojugal ala is tab-like and projects anterodorsally to form the ventral margin of a deep paraquadratic sulcus (Fig. 30A), as in Dryomorpha (Norman, 2004). The portion of the quadratojugal ala dorsal to the paraquadratic sulcus is eroded and missing, as is the squamosal condyle. Viewed dorsoventrally, the ventral part of the quadratojugal ala curves anteromedially (Figs. 30E, 30F). The occurrence of a deep paraquadratic sulcus suggests that the paraquadratic foramen opened laterally, as in Dryomorpha (*e.g.*, Norman, 2004). However, confirmation of a laterally open paraquadratic foramen is curtailed by posterior displacement of the quadrate relative to the quadratojugal. The pterygoid ala is anteromedially directed, triangular in posterior view with a rounded anterior end (Figs. 30B–30D). Viewed posteromedially, the middle portion of the pterygoid ala is depressed by an obliquely angled elliptical fossa, the ventral margin of which, forms a dorsoventrally rounded shelf (Figs. 30B, 30C). This morphology appears unique for an ornithopod and resembles that reported in *Stegosaurus* (Galton & Upchurch, 2004). Differing from *Fostoria dhimbangunmal* (Bell et al., 2019a), a foramen in the column ventral to the quadratojugal ala is absent, the lateral condyle is more developed, and the column is possibly more robust.

#### Pterygoid

Best preserved on the left side, the pterygoid is formed from the quadrate (posterior) ala, ectopterygoid (= ventrolateral, mandibular) process and basipterygoid boss (Figs. 30G–30L). The ectopterygoid process also constitutes the pterygoid flange. The left pterygoid was displaced postmortem by ∼20mm posteroventrally from the ectopterygoid and ∼30mm ventrally from the quadrate (Figs. 23A, 23B, 30G). Unusually, an anteriorly projecting palatine process, typically developed in early diverging neornithischians, such as *Changchunsaurus parvus* (Jin et al., 2010), *Lesothosaurus diagnosticus* (Porro, Witmer & Barrett, 2015) and *Thescelosaurus neglectus* (Boyd, 2014), ornithopods, such as, *Hypsilophodon foxii* (Galton, 1974), *Talenkauen santacrucensis* (Rozadilla, Agnolín & Novas, 2019), *Tenontosaurus tilletti* (Thomas, 2015) and hadrosaurids (Heaton, 1972), is absent and the pterygoid lacks contact with the palatine (Fig. 23A). The sheet-like and dorsoventrally deep quadrate ala broadly contacts the posteromedial surface of its counterpart on the quadrate. The sheet of the quadrate ala is dorsoventrally sinuous, matching the profile of the originally abutting, but now displaced, ala on the left quadrate (Fig. 23A). The posterior margin of the quadrate ala has a swallowtail shape. The posterodorsally directed, dorsal-most part of the quadrate ala is narrow, and a triangular process projects dorsally from midway along the dorsal margin of the quadrate ala (Figs. 30H, 30J, 30G). A fossa occurs on the lateral surface of the ventral part of the quadrate ala, the step-like ventral edge of which (the fossa) aligns with the ventral edge of the pterygoid ala on the quadrate (Figs. 30H, 30J, 30G). This step-like ventral edge is also visible as a low ridge on the medial surface of the left quadrate ala (Fig. 30G). The ectopterygoid process is thickened, postero-lateroventrally oriented, triangular in cross-section and tapered towards its distal end (Figs. 30H–30J). The articular face for the ectopterygoid is dorsolaterally facing. The basipterygoid boss is formed anteriorly (Figs. 30H, 30K, 30L). The boss forms a fossa that enwraps the basipterygoid process of the parabasisphenoid (Figs. 6A, 7B). A narrow, dorsally convex shelf projects medially from the basipterygoid boss for the descending process of the vomer (Figs. 23A, 23D). Differing from styracosternans (*e.g.*, Heaton, 1972; Norman, 1980), contact between the pterygoid and maxilla is absent. Contact between the pterygoid and vomer has been previously described in *Tenontosaurus tilletti* (Thomas, 2015) and potentially occurs in other ornithopods, such as *Dysalotosaurus lettowvorbecki*, *Edmontosaurus regalis* and *Iguanodon bernissartensis* (see Thomas, 2015) but currently lacking clarification. Nevertheless, sole contact between the pterygoid and vomer in *Muttaburrasaurus langdoni*, without pterygoid-palatine contact, appears unique. *Muttaburrasaurus langdoni* lacks the medially-projecting, horizontal flange, developed ventrally on the quadrate ala of *Dysalotosaurus lettowvorbecki* (Hübner & Rauhut, 2010) and *Gryposaurus notabillus* (ROM 873; M. Herne, pers. obs., 2019).

#### Ectopterygoid

**Figure 31:**
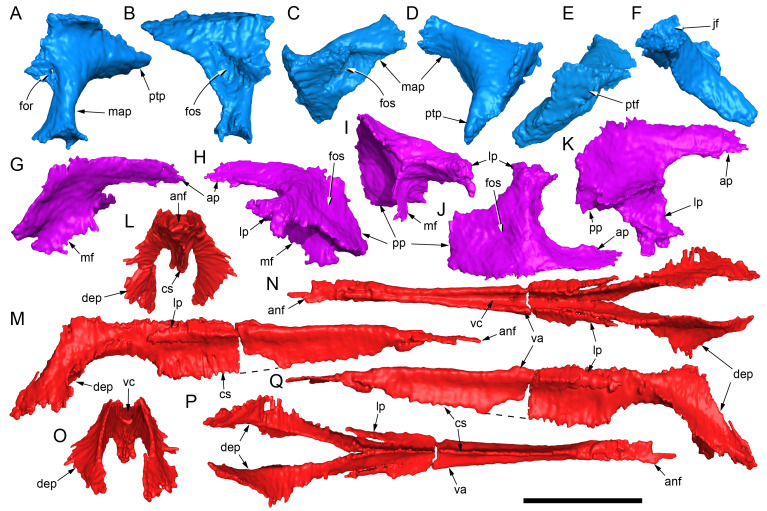
Volume rendered models of the *Muttaburrasaurus langdoni* (QMF6140) palatal bones. (A–F) Left ectopterygoid in (A) dorsal, (B) ventral, (C) anterior, (D) posterior, (E) medial and (F) lateral views. (G–K) Left palatine in (G) medial, (H) lateral, (I) anterior, (J) dorsal and (K) ventral views. (L–Q) Vomera in (L) anterior, (M) right lateral (dashed line indicates reconstructed edge), (N) dorsal, (O) posterior, (P) ventral and (Q) left lateral (dashed line indicates reconstructed edge) views. Abbreviations: anf, anterior flange; ap, anterior process; cs, choanal septum; dep, descending process; for, foramen; fos, fossa; jf, jugal facet; lp, lateral process; map, maxillary process; mf, maxillary flange; pp, posterior process; ptf, pterygoid facet; ptp, pterygoid process; va, vomerine ala; vc, vomerine channel. Scale bar equals 10 cm. MorphoSource DOI: 10.17602/M2/M787746; 10.17602/M2/M790005; 10.17602/M2/M790002.

L-shaped in dorsoventral view, the ectopterygoid consists of a posteroventrally sloping pterygoid process, laterally projecting maxillary process and anteriorly projecting palatine process (Figs. 23, 31A–31F). The left ectopterygoid was displaced ∼30mm posterodorsally from the pterygoid (Fig. 23B). The ectopterygoid abuts the posterior end of the palatine (Fig. 23) and the distal end of the maxillary process abuts the jugal. The convex dorsal surface and dorsally upturned medial crest are continuous with the dorsal surface of the palatine. Together, these coaligned surfaces likely subtended the (musculus) *m. pterygoideus dorsalis* (Holliday, 2009; Holliday & Witmer, 2007). The bar-like maxillary process has a triangular cross-section; flaring at its distal end to form a sutural flange for the jugal (Fig. 23D). The anteroventral surface of the maxillary process tightly articulates with the posterior margin of the maxilla. A deep fossa ventrally on the maxillary process accommodates the spinose posteromedial process of the maxilla (Figs. 23C–23G), as in *Tenontosaurus tilletti* (Thomas, 2015). The anterior end of the palatine process forms a shallow notch that accommodates the posterior end of the palatine (Fig. 23D, 23E) and a small foramen is present at the base of the palatine process (Fig. 31A). Articulation between the ectopterygoid and maxilla is restricted to the posterior shelf on the maxilla, differing from lateral articulation on the maxilla in styracosternans (Norman, 1980), which is particularly extensive in hadrosauroids (Heaton, 1972). As in *Muttaburrasaurus langdoni*, the ectopterygoid and palatine connect in at least *Tenontosaurus tilletti* (Thomas, 2015) and *Thescelosaurus neglectus* (Boyd, 2014). In contrast, the ectopterygoid and palatine do not appear to connect in the early diverging neornithischians *Changchunsaurus parvus* (Jin et al., 2010), *Lesothosaurus diagnosticus* (Porro, Witmer & Barrett, 2015) and *Hypsilophodon foxii* (Galton, 1974). The nature of contact between the ectopterygoid and palatine in early diverging styracosternans is uncertain, but contact is lacking in the hadrosaurids (*e.g.*, *Edmontosaurus regalis* (Heaton, 1972; Lambe, 1920)). Contact between the ectopterygoid and jugal is minimal in *Mantellisaurus atherfieldensis* (Norman, 1986) and absent in the hadrosaurids (Heaton, 1972). The ectopterygoid and palatine in *Muttaburrasaurus langdoni* are superficially similar in shape. However, the ventral fossa on the ectopterygoid and the lack of a maxillary flange differentiate the ectopterygoid from the palatine. It is notable that the isolated element, tentatively identified as the palatine in *Ouranosaurus nigeriensis* (*sensu* Taquet, 1976; GDF 300 cast in MNHN; M. Herne, pers. obs., 2009), more closely resembles the ectopterygoid of *Muttaburrasaurus langdoni* than the palatine.

#### Palatine

The palatine consists of sheet-like anterior and posterior processes, the strut-like lateral process and the maxillary flange (Figs. 31G–31K). The maxillary flange adjoins the maxilla on the dorsomedial surface close to the posterior end of the dental ramus (Figs. 23D, 23F). The transversely-broad posterior process is posteroventrally sloping, quadrangular in dorsal view, and depressed dorsally by a fossa that forms the origin of the *m. pterygoideus dorsalis* (Holliday & Witmer, 2007; Witmer, 1997a) (Figs. 31H–31K). The posterior end of the posterior process contacts the ectopterygoid (Figs. 23B–23G). The medial margin of the posterior process is upturned forming a sharp crista that continues to the anterior process. The ventral surface of the posterior process is shallowly concave and merges with the vertically oriented sheet of the maxillary flange (Figs. 31G, 31I). The anterior process is strap-like and anteriorly tapering, merging with the posterior process to form the dorsally arching alar (= medial) ramus that forms the osseous posterodorsal wall of the *ductus nasopharyngeus* (see in “Results and Discussion”). The left and right palatines would have been separated by a broad midline gap (Fig. 23D). The lateral process is comparatively robust, anterolaterally-directed, and triangular in cross-section (as in the ectopterygoid) (Figs. 31A–31D). The dorsal apex of this triangle forms a low ridge that extends the length of the lateral process to the upturned medial margin of the palatine. Ventrally, the lateral process extends horizontally across the dorsal surface of the dental ramus of the maxilla and connects ventrally at its distal end with the lateral palatine flange on the maxilla (Figs. 21C, 23D, 23E), comparable to the morphology in *Galleonosaurus dorisae* Herne et al., 2019, but differing from the oblique/sub-vertical sutural surface between the lateral process of the palatine and maxilla in *Hypsilophodon foxii* (see Herne et al., 2019), *Tenontosaurus tilletti* (Thomas, 2015) and hadrosaurids (Heaton, 1972). The palatines of the non-hadrosaurid Styracosterna, *Altirhinus kurzanovi*, *Iguanodon bernissartensis* and *Mantellisaurus atherfieldensis*, lack a laterally extending maxillary process (Norman, 1998; Norman, 1980; Norman, 1986). Distally, the lateral process contacts the medial surfaces of the jugal and lacrimal and the ventral process of the prefrontal, as in *Tenontosaurus tilletti* (Thomas, 2015) and possibly in *Hypsilophodon foxii* (Galton, 1974; NHMUK R2477; M. Herne, pers. obs., 2009). The palatine lacks contact with the pterygoid (see also under “Pterygoid” above).

#### Vomer

The vomera are well preserved on the holotype. The bones are fused along the anterior two-thirds of their length to form a midline vomerine shaft, posteriorly from which, they diverge into co-lateral descending processes (Figs. 31L–31Q) (= triangular processes in *Tenontosaurus tilletti*; (Thomas, 2015)). In dorsoventral view, the fused vomera is a Y-shaped element and approximately equal the anteroposterior length of the maxilla (Figs. 23A, 23C, 23D, 31M, 31N, 31P). The shaft is spearhead-shaped in lateral view (Figs. 23C, 23D, 31M, 31MQ) and Y-shaped in transverse section, forming a deep U-shaped vomerine channel (Figs. 23D, 25C–25F). Ventrally, the thin, midline choanal septum deepens posteriorly (Figs. 31L–31Q). Spinose lateral processes project posteriorly from the lateral margins of the vomerine alae (Figs. 31M, 31N, 31P, 31Q). The vomerine channel is aligned with the channel dorsally on the cultriform process of the parasphenoid. A cartilaginous nasal ethmoid would have attached to the vomerine channel (see further in “Results and Discussion”). The anterior end of the vomerine shaft forms a dorsoventrally flattened anterior flange that locates in the space between the palatal shelf of the left and right premaxillae and the premaxillary processes of the maxilla (Figs. 23A, 23C, 23D). This condition is possibly shared with *Thescelosaurus neglectus* (Boyd, 2014) and differs from *Hypsilophodon foxii*, where the vomera appear to insert between the anterior processes of the maxillae (Galton, 1974). It also differs from *Tenontosaurus tilletti*, where the vomera firmly connect with the premaxillary palate (Thomas, 2015) and hadrosaurids, where the vomera pass between the anterior ends of the maxillae to insert in the premaxillae (Heaton, 1972). There is the possibility that the anterior end of the vomera in the holotype slotted in the medial groove of the premaxillary process of the maxilla and slipped out of articulation postmortem. However, this condition seems highly unlikely owing to the transversely broad flange on the vomera.

The vomerine shaft forms the osseous medial margin of the *fenestra choanalis* (Fig. 23D). The shaft extends posteriorly from the premaxilla to a point roughly coinciding with the anterior tip of the cultriform process of the parasphenoid (Fig. 6A). Medial to the lateral processes, the descending processes diverge posterolaterally (Figs. 31L–31Q). The anterior-most parts of the diverging descending processes, form bridge-like, smoothly rounded, horizontal bars of bone (Figs. 31M, 31Q), assessed here to be the osseous medial support of the fleshy *fenestra endochoanalis* (see further in “Results and Discussion”). The descending processes are posterolaterally flaring, posteroventrally sloping and triangular in dorsoventral view (Figs. 23C, 23D, 31L–31Q). The ragged lateral margins of the descending processes suggest cartilaginous sheets continued beyond the osseous margins, as suggested in *Tenontosaurus tilletti* (Thomas, 2015). The flared sheets of the descending processes would have formed the posterior wall of the *oropharynx* (see further in “Results and Discussion”; see also Thomas, 2015). The distal (ventral) ends of the descending processes contact the pterygoids (Figs. 23A, 23D). A large gap is present between the vomer and palatine on the left side (Figs. 23A, 23D–23E) and it seems unlikely that these bones would have originally made contact.

Detailed descriptions of the vomera are lacking in many ornithopods, making extensive comparisons difficult. Continuation of the Y-shaped cross-section of the vomerine shaft anteriorly to the premaxilla differs from *Tenontosaurus tilletti*, where the choanal septum is developed only in the posterior portion of the shaft (Thomas, 2015) and the anterior portion instead forms a broad shallow channel. Differing from *Muttaburrasaurus langdoni*, hadrosaurid vomera, as well as in *Mantellisaurus atherfieldensis*, are bifurcated along most of their length and join anteriorly where they insert into the premaxilla (Heaton, 1972; Norman, 1986). Similarly to *Muttaburrasaurus langdoni*, the choanal septum in *Gryposaurus* sp. (= ‘cf. Kritosaurus’; Horner, 1992, plates 23–24; YPM-PU16970, Prieto-Márquez, 2014) is developed up to premaxillary contact. However, unlike *Muttaburrasaurus langdoni*, the choanal septum in *Gryposaurus* sp. remains dorsoventrally deep, forming a keel extending to the posteroventral termination of the vomera. Vomera similar in shape to those of *Gryposaurus* sp., occur in other hadrosaurids, such as *Brachylophosaurus canadensis* and *Corythosaurus excavatus* (Heaton, 1972). In *Muttaburrasaurus langdoni*, the choanal septum terminates at the divergence of the descending processes, as mentioned. In *Tenontosaurus tilletti*, however, the choanal septum, rather than the vomerine alae, appears to diverge to form the left and right descending processes around the cultriform process (Thomas, 2015, figs. 9, 10.1). The posterior end of the vomer in *Hypsilophodon foxii* has not been documented (following Galton, 1974). A deep vomerine channel occurs in *Hypsilophodon foxii* (Galton, 1974), as in *Muttaburrasaurus langdoni*, but absent in hadrosaurids. The vomerine channel occurs more posteriorly in *Tenontosaurus tilletti* (Thomas, 2015) than in *Muttaburrasaurus langdoni*. The vomer and pterygoid likely connect in all Archosauria (*sensu*, Sereno, 1991), although the nature of connection between these bones is poorly known in many ornithischians. Well-developed, broadly diverging, descending processes in *Muttaburrasaurus langdoni*, articulating at their distal ends with the pterygoids, are comparable to the triangular processes in *Tenontosaurus tilletti* (Thomas, 2015). Similar processes could occur in other ornithopods (see Thomas, 2015) but not presently described.

#### Prefrontal

**Figure 32:**
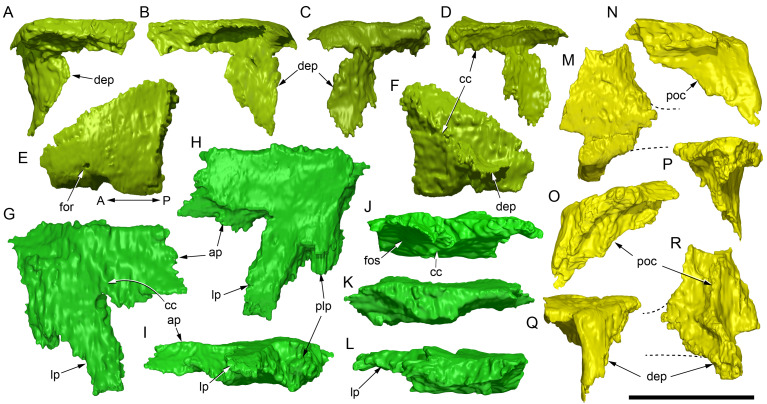
Volume rendered models of the *Muttaburrasaurus langdoni* (QMF6140) bones of the cranial roof. (A–F) Left prefrontal in (A) posterior, (B) anterior, (C) lateral, (D) medial, (E) dorsal and (F) ventral views. (G–L) Left frontal in (G) ventral, (H) dorsal, (I) lateral, (J) anterior, (K) medial and (L) posterior views. (M–R) Left postorbital in (M) dorsal, (N) anterior, (O) posterior, (P) medial, (Q) lateral and (R) ventral views (dashed lines indicate missing squamosal process). Abbreviations: A, anterior; ap, anterior process; cc, *crista cranii*; dep, descending process; for, foramen; fos, fossa; lp, lateral process; plp, posterolateral process; poc, posterior orbital crista; vod, ventral orbital ridge. Scale bar equals 10 cm. MorphoSource DOI: 10.17602/M2/M788113; 10.17602/M2/M788116; 10.17602/M2/M788124; 10.17602/M2/M788121.

The prefrontal consists of an isosceles triangular-shaped, plate-like body, ventrally from which, extends a lengthy descending process (Figs. 32A–32F). The dorsal surface of the prefrontal body is shallowly convex, and the posteromedial tip rounded. The body thickens towards its posteromedial apex. Viewed laterally, the anterolateral tip is dorsally convex and ventrally concave where it abuts the lacrimal (Figs. 5A, 32C). The medial and posterior margins insert into a deep anterolateral notch formed by the nasal and frontal, whereas the medial margin overlies both the nasal and frontal along a bevelled margin (Figs. 6B, 29B). A foramen passes through the anterolateral portion of the prefrontal body (Fig. 32E). The ventral surface in this region, anterior to the descending process, is concave (Fig. 32B). The descending process is sheet-like and transversely broad, although obliquely angled along its dorsoventral axis and mediolaterally tapered (Figs. 32A–32D). The descending process closely abuts the medial surface of the lacrimal to form the medial part of the anterior orbital margin (Figs. 5A, 29C). The ventral tip of the descending process contacts the lateral ramus of the palatine (Fig. 29C), as in *Tenontosaurus tilletti* (Thomas, 2015) and most likely *Hypsilophodon foxii* (NHMUK R2477; M. Herne, pers. obs., 2009). Viewed ventrally, the orbital crista extends from the descending process to the posteromedial tip of the prefrontal body (Figs. 30B, 32F). The crista divides the ventral surface into two fossae. The dorsoventral shape of the prefrontal varies among ornithopods. However, a strap-like shape with distinct anterior and posterior processes and posteriorly-tapering posterior process, generally occurs in less derived ornithopods (*Convolosaurus marri* Andrzejewski, Winkler & Jacobs, 2019; *Hypsilophodon foxii* (Galton, 1974); *Zalmoxes robustus* Weishampel et al., 2003) and in early diverging neornithischians (*e.g.*, *Haya griva* (Barta & Norell, 2021); *Thescelosaurus neglectus* (Boyd, 2014)), whereas a broader, plate-like prefrontal typically occurs in derived iguanodontians (*e.g.*, *Tenontosaurus tilletti* (Thomas, 2015); *Edmontosaurus regalis* (Xing, Mallon & Currie, 2017)), although, not in all (*Camptosaurus dispar* (Carpenter & Lamanna, 2015); *Dysalotosaurus lettowvorbecki* (Janensch, 1955)). The shape of the prefrontal body, broadening transversely in the posterior direction, resembles that of *Ouranosaurus nigeriensis* Taquet, 1976. In the original description, Bartholomai & Molnar (1981) suggested that the prefrontal extended along the posterolateral surface of the muzzle and contacted the premaxilla anteriorly. However, this region of bone is now reassessed as part of the nasal.

#### Frontal

**Figure 33:**
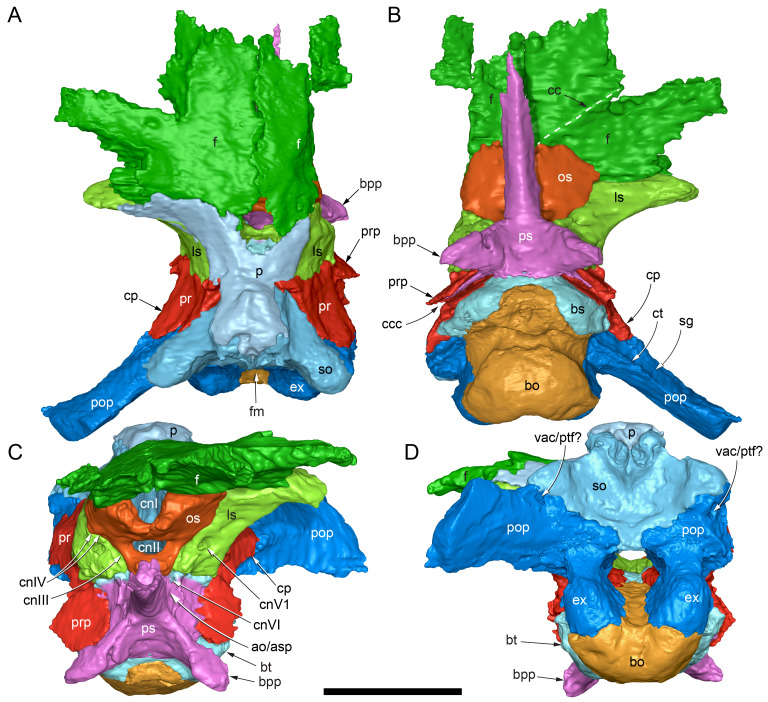
Volume rendered model of the *Muttaburrasaurus langdoni* (QMF6140) neurocranium. (A–D) Neurocranium in (A) dorsal, (B) ventral, (C) anterior and (D) posterior views. Abbreviations: ao, (foramen of) ophthalimic artery; aoc, (foramen of) occipital artery; asp, sphenoid artery; bo, basioccipital; bpp, basipterygoid process; bs, basisphenoid; bt, basal tubera; cc, *crista cranii*; ccc, cerebral carotid canal; cn#, cranial nerve and number; cp, *crista prootica*; ct, *crista tuberalis*; ex, exoccipital; f, frontal; fm, foramen magnum; ls, laterosphenoid; os, orbitosphenoid; p, parietal; pop, paroccipital process; pr, prootic; prp, prootic pendant; ps, parasphenoid; ptf?, posttemporal fenestra?; sg, stapedial groove (connecting the tympanic fossa); so, supraoccipital; vac, vascular canal. Scale bar equals 10 cm.

**Figure 34:**
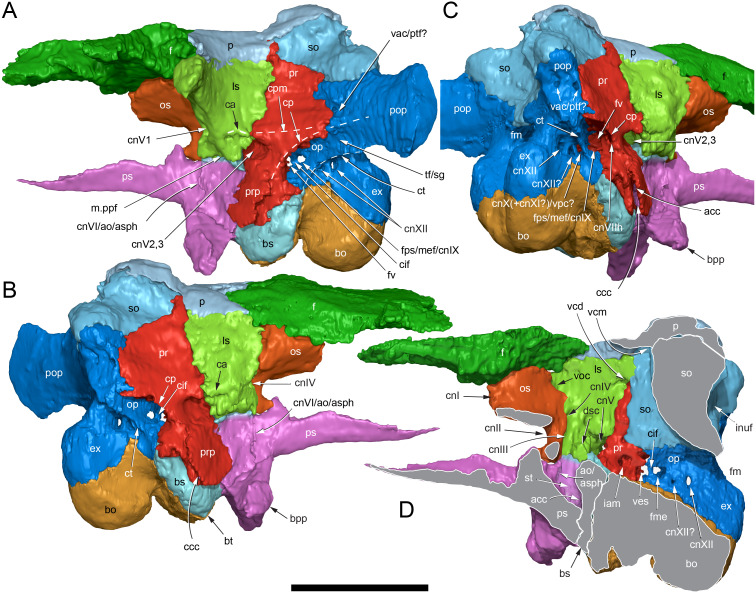
Volume rendered model of the *Muttaburrasaurus langdoni* (QMF6140) neurocranium. (A–D) Neurocranium in (A) left lateral, (B) right lateral and (C) posterolateral views. (D) Parasagittal section of left side. Abbreviations: acc, (foramen of) cerebral carotid artery; ao, (foramen of) ophthalimic artery; asph, (foramen of) sphenoidal artery; bo, basioccipital; bpp, basipterygoid process; bs, basisphenoid; bt, basal tubera; ca, *crista antotica*; ccc, cerebral carotid canal; cif, crista interfenestralis; cn#, foramen of the cranial nerve and number; cp, *crista prootica* (= otosphenoidal crest); cpm, *crista prootica media*; ct, *crista tuberalis (*=* c. metotica)*; dsc, *dorsum sellae cristae*; ex, exoccipital; f, frontal; fm, foramen magnum; fme, *fissura metotica* (passage of cnIX–XI/posterior cephalic vein); fps, fenestra pseudorotunda; fv, fenestra vestibularis; iam, internal auditory meatus; inuf, inferior nuchal fossa; ls, laterosphenoid; mef, metotic foramen; m.ppf, fossa and superior crista for *musculus protractor pterygoideus*; op, opisthotic; os, orbitosphenoid; p, parietal; pop, paroccipital process; pr, prootic; prp, prootic pendant; ps, parasphenoid; ptf?, posttemporal fenestra?; sg, stapedial groove (connecting the tympanic fossa); so, supraoccipital; st, *sella turcica*; tf, tympanic fossa; vac, vascular canal; vcd, (foramen of) *vena capitis dorsali* s; vcm, (foramen of) *vena capitis medialis*; ves, auditory vestibule; voc, (foramen of) orbitocerebral vein; vpc, (foramen of) posterior cephalic vein?. Scale bar equals 10 cm.

The frontals form the anterodorsal part of the neurocranial-sphenethmoid series of bones. Viewed dorsoventrally, the frontal is roughly L-shaped and consists of a plate-like body and prominent anterior and lateral processes. A smaller posterolateral process is also present (see further below) (Figs. 32G, 32H; for dorsal dimensions, see Table 1). The dorsal surface of the better preserved left frontal appears to have been partially abraded or subaerially weathered but is mostly complete. The dorsal surface is planar overall, although undulating. The anterior process is mediolaterally broad but dorsoventrally thin and quadrangular in dorsal view (Figs. 6B, 29B, 32G, 32H). The anterior margin forms an obtuse angle of 115° relative to the medial border, and a broad scarf joint is formed laterally for the nasal. The lateral process is narrow and slightly anterolaterally oriented, extending to the orbital margin (contra Bell et al., 2019a) between the prefrontal and postorbital (Figs. 6B, 29B, 32G, 32H). The frontal contributes ∼20% of the dorsal orbital margin (Figs. 32G, 32H). A small posterolateral process extends from near the base of the lateral process. A notch formed between the lateral and posterolateral processes accommodates the anteromedial end of the postorbital. The postorbital articulates along the posterior margin of the lateral process and lateral margin of the posterolateral process (Figs. 6B, 29B, 32G–32L). The posterolateral process forms an abutting scarf joint with the postorbital (Figs. 6B, 29B, 32H, 32I); morphology consistent with more derived ornithopods than the peg-and-socket joint of early diverging ornithopods (see Weishampel, 1984). The frontal body sutures with the parietal posteriorly and the laterosphenoid along the posteroventral margin (Figs. 29B, 33A–33C). An external opening along the sagittal margin at the junction of the paired frontals and parietals and extending into the cranial fossa, likely resulted from erosion (Fig. 23A). Ventrally on the paired frontals, the *crista cranii* form an hourglass shape, ventral to which, the orbitosphenoid and the laterosphenoids attach (Fig. 29B). The *crista cranii,* which form the lateral margins of the olfactory tracts, extend anterolaterally from the orbitosphenoid facet to the apex of the notch for the prefrontal, from which point, they continue as the descending processes of the prefrontals (Figs. 29B, 33A–33C). The orbital cartilage would have attached to the *crista cranii* (based on (Ali et al., 2008)). A ventrally depressed, flattened ridge in the ‘waist’ of the hour-glass-shaped fossa between the paired *crista cranii* overlies the path of the olfactory tracts (Fig. 34D). Thus, the olfactory tracts were ventrally offset relative to the dorsal surfaces of the cerebrum and the olfactory bulbs (see further in “Palaeoneurology” below). From the CT radiographs, a vertical suture is visible extending anterolaterally through the frontal on both sides coinciding with the *crista cranii* (Figs. 29D–29F). The interdigitating appearance of this sutural margin and its occurrence on both frontals mitigate against its presence being from breakage and is consistent with the sutural margins described in the frontals of some theropods (Ali et al., 2008). This suture appears to identify a separate medial ossification referred to in theropods as the dorsal plate of the frontal (Ali et al., 2008). The dorsal plates appear to form the complete anterior region of the paired frontals between the *crista cranii* and have not been previously described in any other ornithischian. Furthermore, the presence of dorsal plates on the frontals in *Muttaburrasaurus langdoni* and Theropoda suggests morphology that was more broadly distributed across Dinosauria. A secondary ridge ventrally on the frontals anteromedial to the *crista cranii*, divides two distinct fossae on each side (Fig. 31B). One fossa is located between the secondary ridge and the *crista cranii* and the other fossa is between the secondary ridge and the midline suture (Figs. 29B, 29D, 29E). The paired secondary ridges are considered here to form the lateral boundaries of the olfactory bulbs, rather than being formed by the *crista cranii*, which appear too laterally divergent for this function. The secondary ridges are termed herein, olfactory crista. The posterior orbital crista extends laterally from the orbitosphenoid suture to the adjoining ridge on the postorbital (Fig. 29B). The anteroposterior length and mediolateral width of the frontal are sub-equal (Table 1), as in styracosternans (*e.g.*, *Altirhinus kurzanovi* (Norman, 1998); *Edmontosaurus regalis* (Lambe, 1920); *Iguanodon bernissartensis* (Norman, 1980); *Ouranosaurus nigeriensis* Taquet, 1976). The combination of frontal proportions, an elongate lateral process and the narrow degree of orbital contribution by the frontal is comparable to *Edmontosaurus regalis* (Lambe, 1920), *Gryposaurus notabilis* (Prieto-Márquez, 2010a) and *Ouranosaurus nigeriensis* (Taquet, 1976). The frontal of *Fostoria dhimbangunmal* differs from *Muttaburrasaurus langdoni* in lacking an extended lateral process and a deep notch at the posterolateral end of the frontal for the postorbital (Bell et al., 2019a).

#### Postorbital

The left postorbital is well preserved but missing most of the posterior (squamosal) process (Figs. 29A, 29B, 32M–32R). The plate-like dorsal body of the postorbital is quadrangular, shallowly convex dorsally and slopes lateroventrally. It abuts the frontal anteriorly and medially, the parietal posteromedially and the laterosphenoid posteroventrally (Fig. 29B). The descending process tapers ventrally and abuts the anterior margin of the dorsal process of the jugal (Fig. 29A). The lateral margin of the descending process is laterally protrusive relative to the dorsal orbital margin (Figs. 5A, 6B, 32M, 32R). The posterior orbital crista forms a transversely broad ridge from the descending process and continues along the ventral surface of the postorbital body to its medial contact with the frontal (Figs. 29B, 32N–32P, 32R). A fossa is formed on the posterior side of the posterior orbital crista and the descending process that accommodates a supernumerary, intrapostorbital ossification (Fig. 29A; see “Intrapostorbital” below). In anteroposterior view, the descending process is angled at 120° relative to the body (Figs. 32N, 32O). The abrupt deflection of the descending process from the body differs from the comparatively rounded transition in other ornithopods, such as *Hypsilophodon foxii* (Galton, 1974), *Iguanodon bernissartensis* (Norman, 1980), *Tenontosaurus tilletti* (Thomas, 2015) and *Zalmoxes robustus* Weishampel et al., 2003.

#### Intrapostorbital ossification

A thin elongate ossification, evident in the CT imagery, extends within a shallow fossa on the medially surface of the postorbital process of the jugal and the descending process of the postorbital (Fig. 29A). The way in which this element is continuous across the sutural margin between the jugal and postorbital, supports the identification of a separate ossification centre from the latter two bones. Thus, an elongate supernumerary ossification is proposed, not previously described in any other dinosaur, and termed the intrapostorbital ossification.

#### Squamosal

Only the posterior regions of the squamosals are preserved on the left and right sides (Figs. 5, 6B, 7B) with the left more complete than the right. The anterior (postorbital) process, quadrate cotylus and the prequadratic and postquadratic processes are missing on both sides. The medial process extends along the dorsal margins of the paroccipital process and the supraoccipital and tightly abuts the posterolateral corner of the parietal (Figs. 5, 6B, 7B), as in *Camptosaurus dispar* (Carpenter & Lamanna, 2015; Gilmore, 1909), *Hypsilophodon foxii* (Galton, 1974), *Iguanodon bernissartensis* (Norman, 1980), *Mantellisaurus atherfieldensis* (Norman, 1986), *Tenontosaurus tilletti* (Thomas, 2015) and hadrosaurids (*e.g.*, Ostrom, 1961; Xing, Mallon & Currie, 2017). Notably, the medial process may not have reached the parietal in the rhabdodontid *Zalmoxes robustus* (Weishampel et al., 2003), or *Iani smithi* Zanno et al., 2023 (fig. 3). The anteroventral corner of the medial process uniquely contacts the prootic (Figs. 5, 6B). In addition, the anterior end of the medial process is truncated by the narrow dorsal process of the prootic (Figs. 5, 6B). The medial process contributes to approximately half of the supratemporal fenestral margin, which is possibly a greater contribution than in other ornithopods. Differing from *Tenontosaurus tilletti* and styracosternans, except for *Ouranosaurus nigeriensis*, the paired squamosals are broadly separated medially by the parietals and fail to roof the nucal fossa. In iguanodontians, such as *Tenontosaurus tilletti* (Thomas, 2015), styracosternans, such as *Iguanodon bernissartensis* (Norman, 1980) and the hadrosaurids (*e.g.*, *Corythosaurus casuarius* (ROM 00776; M. Herne, pers. obs., 2019); *Gryposaurus notabilis* (ROM 873; M. Herne, pers. obs., 2019); *Edmontosaurus regalis* (Xing, Mallon & Currie, 2017)), the medial process is anteroposteriorly broad and dorsoventrally deep and the left and right squamosals nearly contact each other at the midline; thus, roofing a deep nuchal fossa floored by the supraoccipital. Differing from the rhabdodontomorphs, the posttemporal foramen in *Muttaburrasaurus* passes through the paroccipital process, not the squamosal (Zanno et al., 2023).

#### Neurocranium overview

Bones of the neurocranium include the frontals (described above), fused parietals and parasphenoid of the dermatocranium, the laterosphenoids and midline orbitosphenoid, supraoccipital and basisphenoid of the chondrocranium and prootics, basioccipital and fused opisthotic-exoccipital-paroccipital process (= otoccipital) of the splanchnocranium (Figs. 29B, 33, 34). From external observations and the CT imagery, many of the sutural margins between the bones of the neurocranium, other than between the frontals and fused parietals, show fusion (see Fig. 4), as in somatically mature iguanodontians (Norman, 2004). However, fusion of the margins between many of the adjoining bones (laterosphenoids, prootics, opisthotics, supraoccipital, basioccipital, basisphenoid, parasphenoid) was either incomplete or showed a texture suggesting that the margins were in the process of knitting together. In these cases, during volume rendering, the partially ankylosed margins between bones were interpolated (see also in “Methods”). Typical of other dinosaurs, fusion between the opisthotic, exoccipital and paroccipital process was complete on both sides, which likely occurred at the embryonic stage (Horner & Currie, 1994).

#### Parietal

**Figure 35:**
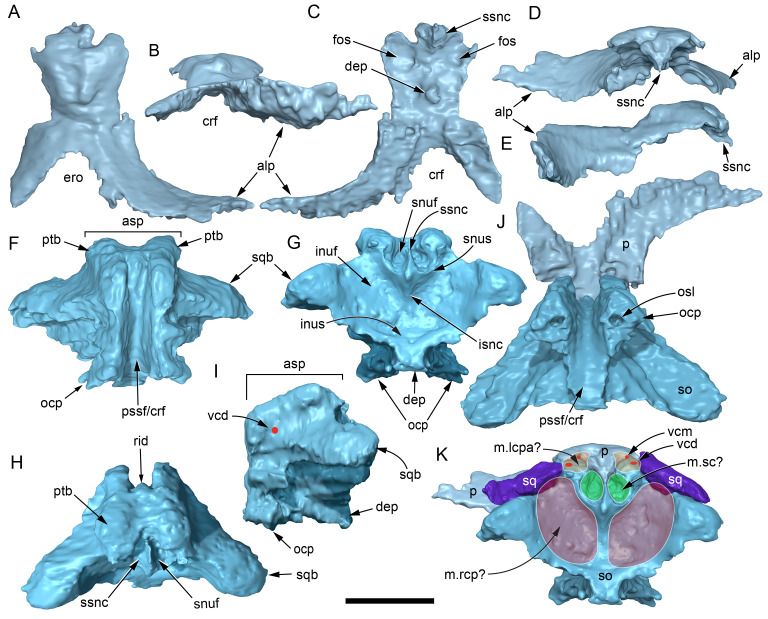
Volume rendered models of *Muttaburrasaurus langdoni* (QMF6140) neurocrania. (A–E) Fused parietals in (A) dorsal, (B) anterior, (C) ventral, (D) posterior and (E) left lateral views. (F–I) Supraoccipital in (F) anterior, (G) posterior, (H) dorsal and (I) left lateral views. (J) Supraoccipital articulated with parietals in ventral view. (K) Supraoccipital articulated with parietals and squamosals in posterior view showing areas of attachment of cranio-axial musculature. Abbreviations: alp, anterolateral process; asp, ascending process (of supraoccipital); crf, cranial fossa; dep, descending process; ero, erosion; fos, fossa; inus, inferior nuchal shelf; inuf, inferior nuchal fossa; isnc, inferior sagittal nuchal crest; m. lcpa?, (potential) insertion surface for *musculus longissimus capitis, pars articuloparietalis* (orange shading); m. rcp?, (potential) insertion surface for *musclulus rectus capitis posterior* (red shading) m.sc?, (potential) insertion surface for *musclulus spinalis capitis* (green shading); ocp, otic capsule process; osl, osseous labyrinth; p, parietal; pssf, posterior sagittal sinus fossa; ptb, protuberance; rid, ridge; sqb, squamosal boss; snuf, superior nuchal fossa; snus, superior nuchal shelf; so, supraoccipital; ssnc, superior sagittal nuchal crest; sq, squamosal; vcd, (foramen of) *vena capitis dorsalis*; vcm, (foramen of) *vena capitis medialis*. Scale bar equals five cm. MorphoSource DOI: 10.17602/M2/M788134; 10.17602/M2/M788137.

The fused parietals are incomplete, missing the right anterolateral process and are eroded dorsally, partially exposing the cranial endocast (Figs. 29B, 33A, 34D, 35A, 35B). When complete, the parietals would have been Y-shaped in dorsal view. The parietals form the anteromedial surfaces of the supratemporal fenestrae. The body of the parietals roof the cerebellar region of the rhombencephalon (= hindbrain; see further under “Palaeoneurology” below). The anterolateral process contacts the frontal anteriorly, the laterosphenoid ventrally and the postorbital at its anterolateral end (Figs. 6B, 33A, 34). The parietal-frontal suture is interdigitating, although the midline region has been lost to erosion (Figs. 33A, 34). Posterior to laterosphenoid contact, the posterior region of the parietal body is dorsally offset from the anterior region, forming a distinct step in lateral view (Figs. 33A, 34A, 34B, 34D, 35D, 35E). In this posterior region, the parietal body contacts a dorsal process of the prootics and dorsally caps the supraoccipital. Contact between the parietal body and the prootics is consistent with Dryomorpha. Posteriorly, the parietal body forms a domed, sub-rounded cap dorsally to the supraoccipital and, generally differing from other ornithopods, lacks posterolateral processes and a transverse nuchal crest (Figs. 33A, 34, 35). Marked doming of the parietals to accommodate the ascending process of the supraoccipital is unusual for an ornithopod and possibly Ornithischia, but reported in abelisaurids (Paulina Carabajal, 2011a, Paulina Carabajal, 2011b; Sampson & Witmer, 2007). The posterolateral margin aligns with the medial processes of the squamosals, but these margins are not closely abutting (Fig. 6B). Ventrally, articulation with the supraoccipital is unusual. The ventral surface is excavated by two fossae that cap twin dorsal protuberances on the ascending process of the supraoccipital. This contrasts with the single midline process found in other ornithopods (see also under “Supraoccipital” below) (Figs. 35C–35E). Internally in the cranial fossa, a small knob-like median descending process on the parietals inserts into a fossa on the supraoccipital (“dep” in Fig. 35C). Posteriorly within the nuchal fossa, the parietals form the dorsal portion of the superior sagittal nuchal crest (for clarification of the term ‘superior sagittal nuchal crest’ and other divisions of the nuchal fossa identified in this study, see “Supraoccipital” below) (Figs. 35C–35E). The dorsal portion of the superior sagittal nuchal crest on the parietals contacts the ventral portion of the superior sagittal nuchal crest formed on the supraoccipital (see further under “Supraoccipital” below). A descending process on the parietals is reported in this location in *Dysalotosaurus lettowvorbecki* (Janensch, 1955, fig. 6b).

#### Supraoccipital

**Figure 36:**
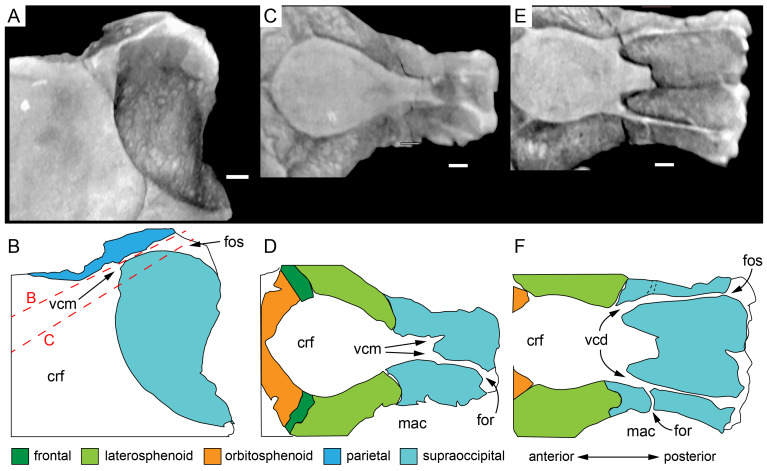
Radiographic sections through the posterior neurocranium of *Muttaburrasaurus langdoni* (QMF6140) showing paths of the posterodorsal cranial veins. (A, B) Parasagittal radiograph through (A) right canal of the *vena capitis dorsalis* and (B) explanatory schematic. (C, D) Dorsal radiograph through (C) canals of the *vena capitis dorsalis* and (D) explanatory schematic. (E, F) Dorsal radiograph through (E) canals of the posterior middle cerebral vein and (F) explanatory schematic. Red dashed lines in B indicate planes of the radiographic sections in C–F and radiographic planes in C–F are aligned to the canals. Abbreviations: crf, cranial fossa; for, foramen; fos, fossa; mac, mandibular adductor chamber; vcd, (canal of) *vena capitis dorsalis*; vcm, (canal of) *vena capitis medialis*. Scale bars equal one cm.

**Figure 37:**
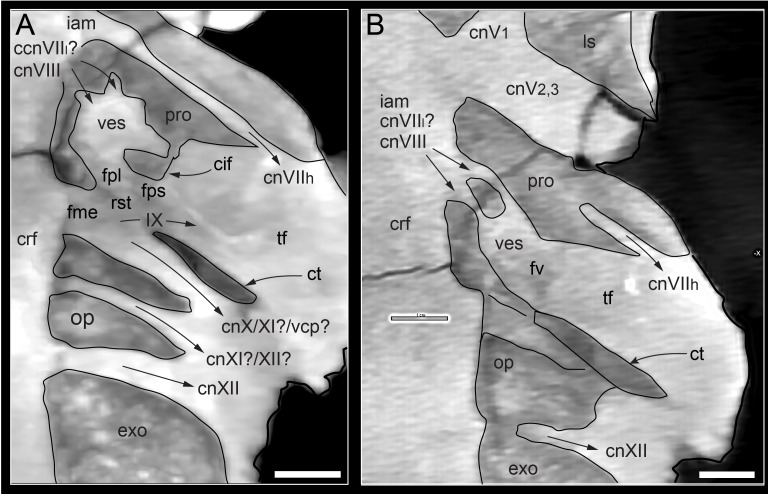
Dorsal radiographic sections with schematic overlays of the *Muttaburrasaurus langdoni* (QMF6140) right lateroventral neurocranium, showing paths of the lateral cranial nerves and posterior cephalic vein. (A, B) Dorsal radiograph at (A) level of the *fissura metotica* and *recessus scalae tympani* and (B) level of the vestibulocochlea nerve rami (note, *crista interfenestralis* is inferior/ventral to this section). Abbreviations: cif, *crista interfenestralis*; cn#, cranial nerve and number (h, hyomandibular ramus); crf, cranial fossa; ct, *crista tuberalis*; exo, exoccipital; fme, *fissura metotica*; fpl, *foramen perilymphaticum*; fps, *fenestra pseudorotunda*; fv, *fenestra vestibularis*; iam, internal auditory meatus; ls, laterosphenoid; op, opisthotic; vcp, posterior cephalic vein; pro, prootic; rst, *recessus scalae tympani* (*apertura lateralis recessus scalae tympani*); tf, tympanic fossa; ves, region of the vestibule. Scale bars equal one cm.

A midline bone of the chondrocranium, the supraoccipital of *Muttaburrasaurus langdoni* is complex. The bone is V-shaped in dorsoventral view, ‘+’-shaped in anteroposterior view and roughly quadrangular in lateral view (Figs. 35F–35K). Viewed laterally (Figs. 34A–34C), the supraoccipital wedges between the parietal, otoccipital and prootic. The stout ascending process is bilobed, the two protuberances inserting into fossae ventrally on the parietals (Figs. 34D, 35). In most other ornithopods, the ascending process is not bilobed and typically narrows dorsally (*e.g.*, *Camptosaurus dispar* (Carpenter & Lamanna, 2015; Gilmore, 1909); *Dryosaurus altus* (Galton, 1983); *Edmontosaurus* sp. (ROM 53494; M. Herne, pers. obs., 2019); *Hypsilophodon foxii* (Galton, 1974); *Iani smithi* Zanno et al., 2023; *Rhabdodon* sp. (Chanthasit, 2010); *Tenontosaurus tilletti* (Thomas, 2015); *Zalmoxes robustus* Weishampel et al., 2003). Additionally, in most ornithopods, the nuchal shelf slopes obliquely in the anterodorsal direction from the posterior margin of the nuchal fossa, and viewed posteriorly, the supraoccipital forms an inverted T-shape; thus, differing from the shape in *Muttaburrasaurus langdoni*. Viewed laterally, the dorsal margin of the ascending process slopes anteroventrally (Fig. 35I), which is opposite to that in other ornithopods where reported, such as *Thescelosaurus* spp. (Boyd, 2014; Brown, Boyd & Russell, 2011) and the early diverging ornithischian *Heterodontosaurus tucki* (Norman et al., 2011). Anteriorly, a midline channel for the posterior sagittal sinus (= dorsal sagittal sinus) extends the full height of the supraoccipital (Figs. 34D, 35F, 35H, 35J). In sagittal cross-section, the channel is anteroventrally convex (Fig. 34D) and U-shaped in dorsoventral view (Figs. 35H, 35J), except dorsally where a small ridge is formed accommodated in a notch on the parietal body (Figs. 35H, 35J, 36B, 36C). The fossa for the posterior sagittal sinus is walled laterally by bilateral otic processes that together with the prootics and otoccipital form the dorsal regions of the endosseous labyrinths of the otic capsules (Figs. 34D, 35F, 35G, 35I, 35J, 37), as noted in *Tenontosaurus tilletti* (Thomas, 2015). The roof for the dural peak of the brain, formed posteriorly by the supraoccipital and dorsally by the parietals, forms an angle of 88° (Fig. 33D). Apart from parasaurolophins, dural peak angle in hadrosaurids is typically greater (≥100°; *e.g.*, Horner, 1992), which could be phylogenetically significant for lambeosaurines (Cruzado-Caballero et al., 2015). The angle close to 90° in *Muttaburrasaurus langdoni* suggests the plesiomorphic state for an ornithischian (see also under “Flexures” below).

Viewed posteriorly, the squamosal bosses are lobate, prominent and triangular (Figs. 33A, 33D, 34, 35F–35K). The region of the nuchal fossa consists of two parts, considered here as forming superior and inferior sub-fossae, with each rimmed ventrally by U-shaped nuchal shelves (Figs. 35G, 35H). The smaller, superior sub-fossa is anteroposteriorly deep and set within the ascending process (Figs. 33D, 35G, Fig. 35H). The parietals partly roof the superior sub-fossa, but notably, not the squamosals (Figs. 7B, 33A, 33D). The superior sub-fossa is divided by a narrow superior sagittal nuchal crest (Figs. 33D, 35D, 35H), the dorsal part of which is formed by the parietals. The anteroposteriorly shallow inferior sub-fossa is transversely broad and dorsoventrally deep (Figs. 34D, 35G). The squamosal bosses form the lateral walls of the inferior sub-fossa. The squamosals and superior nuchal shelf roof the inferior sub-fossa whereas the inferior nuchal shelf floors the fossa (Figs. 34D, 35G, 35K). A subtle sagittal nuchal crest divides the dorsal part of the inferior sub-fossa into bilateral moieties. The crest pinches out ventrally towards the inferior nuchal shelf. From the inferior shelf of the nuchal fossa, a median descending process extends to the dorsal margin of the foramen magnum (Figs. 34D, 35G). The descending process contributes to 20% of the foramen magnum and excludes medial contact between the left and right otoccipitals (Figs. 33D, 35G, 35I).

Following early assessments (Lull & Wright, 1942; Ostrom, 1961), the medial axial bracing ligament, *ligamentum nuchae*, potentially attached within the superior nuchal fossa, while the inferior fossa possibly provided insertion for the superficial epaxial spinal muscle *m. spinalis capitis* (Ostrom, 1961). However, work focussed mainly on marginocephalians (Tsuihiji, 2010) and utilising phylogenetic bracketing, more convincingly suggests that the *m. spinalis capitis* of *Muttaburrasaurus langdoni* inserted in the superior nuchal fossa and the larger inferior fossa was the site of insertion of the *m. rectus capitis posterior* (Fig. 35K). In addition, cervical musculature of the *m. longissimus capitis, pars articuloparietalis*, together with *m. spinalis capitis* (in Crocodilia forming a single muscle mass) and *m. transversospinalis capitis* (following Tsuihiji, 2005; Tsuihiji, 2010), potentially inserted in the fossa posteriorly between the parietals and supraoccipital (Fig. 35K).

The *vena capitis medialis* (= caudal, middle cerebral vein) issues from the cranial fossa dorsolateral to the dural peak (following Evans, Ridgely & Witmer, 2009; Witmer & Ridgely, 2008b; Witmer & Ridgely, 2009) and courses through a canal between the supraoccipital and parietals to exit posterodorsally in the fossa, as mentioned, for insertion of the *m. longissimus capitis, pars articuloparietalis* (Figs. 34D, 35K, Fig. 36C). The main trunk of the *vena capitis dorsalis* (= dorsal head vein) issues from the mid-lateral metencephalic (hindbrain) region of the cranial fossa (based on: Evans, Ridgely & Witmer, 2009; Witmer & Ridgely, 2008b; Witmer & Ridgely, 2009) at the junction of the supraoccipital, prootic and laterosphenoid and courses posterodorsally through the supraoccipital to exit posteriorly at a foramen in a common fossa with the *vena capitis medialis*, although ventrolateral to the latter (Figs. 34D, 36A, 36B). In addition, a lateral branch of the *vena capitis dorsalis* exits in the adductor chamber (Figs. 34D, 35I, 36C), as typically occurs in dinosaurs (Ornithopoda (Evans, Ridgely & Witmer, 2009; Norman, 1980; Sobral, Hipsley & Müller, 2012); Ceratopsia (Witmer & Ridgely, 2008b); Thyreophora (Janensch, 1936); Saurischia and Archosauria in general (Sampson & Witmer, 2007; Witmer & Ridgely, 2009; Witmer et al., 2008)). Sampson & Witmer (2007) and Witmer & Ridgely (2008b) described the *vena capitis dorsalis* and *vena capitis medialis* in the theropods *Majungasaurus cranatissimus* and *Tyrannosaurus rex* as connecting *via* external grooves posteriorly on the occiput at the posttemporal foramen. A common posterior fossa for the *vena capitis dorsalis* and *vena capitis medialis* also occurs in *Muttaburrasaurus langdoni*, although these veins course internally, rather than along external grooves (Figs. 35K, 36). It is of interest that two foramina originally identified by Janensch (1955) in *Dysalotosaurus lettowvorbecki* as “post-parietal gaps”, between the supraoccipital and parietal, alongside the dural peak, were reinterpreted by Sobral, Hipsley & Müller (2012) as pineal foramina. However, the location of these foramina and their communication between the cranial fossa and the dorsal occiput, alternatively suggest they are foramina of the *vena capitis medialis*, as in *Muttaburrasaurus langdoni*.

As in *Muttaburrasaurus langdoni*, supraoccipital contribution to the foramen magnum has been considered a condition more generally retained in early diverging ornithischians (Norman, 2004) and non-styracosternan ornithopods, including *Camptosaurus dispar* (Carpenter & Lamanna, 2015; Gilmore, 1909), *Cumnoria prestwichii* (Maidment et al., 2022), *Convolosaurus marri* Andrzejewski, Winkler & Jacobs, 2019, *Dysalotosaurus lettowvorbecki* (Janensch, 1955), *Gasparinisaura cincosaltensis* (MUCPv-208; M. Herne, pers. obs., 2008), *Hypsilophodon foxii* (Galton, 1974) and Rhabdodontomorpha (Chanthasit, 2010). However, the supraoccipital is excluded from the foramen magnum in *Thescelosaurus* spp. (Boyd, 2014; Brown, Boyd & Russell, 2011), *Tenontosaurus tilletti* (Galton, 1989; Thomas, 2015) and typically in styracosternans (*e.g.*, *Iguanodon bernissartensis* (Norman, 1980); *Proa valdearinnoensis* (McDonald et al., 2012b); hadrosaurids (McDonald et al., 2012a; Ostrom, 1961; Xing, Mallon & Currie, 2017)) but not in all (*Batyrosaurus rozhdestvenski* Godefroit et al., 2012). Contribution of the supraoccipital to the foramen magnum in *Fostoria dhimbangunmal* is uncertain (Bell et al., 2019a).

The occurrence of superior and inferior nuchal sub-fossae in *Muttaburrasaurus langdoni* differs from the singular fossa in all other ornithopods (early diverging ornithopods (Andrzejewski, Winkler & Jacobs, 2019; Galton, 1989); Rhabdodontomorpha (Zanno et al., 2023; Chanthasit, 2010); Dryosauridae (Galton, 1989; Janensch, 1955; Sobral, Hipsley & Müller, 2012); early diverging Ankylopollexia (Carpenter & Lamanna, 2015; Gilmore, 1909); Styracosterna (Horner, 1992; McDonald et al., 2012b; Norman, 1980; Ostrom, 1961; Xing, Mallon & Currie, 2017). Differing from *Muttaburrasaurus langdoni*, the anteroposteriorly deep nuchal fossa in styracosternans (Norman, 1986; Ostrom, 1961) and *Tenontosaurus tilletti* (Thomas, 2015) is roofed by the squamosals, forming a tunnel-like fossa. The superior nuchal sub-fossa in *Muttaburrasaurus langdoni* is possibly equivalent to the total region of the singular nuchal fossa in other ornithopods. However, in other ornithopods the nuchal fossa is typically larger (both transversely and anteroposteriorly) and the ventral floor of the fossa, as mentioned, as well as the dorsal margin of the ascending process, slope anterodorsally from the posterior end of the supraoccipital (*e.g.*, *Camptosaurus dispar* (Carpenter & Lamanna, 2015); *Cumnoria prestwichii* (Maidment et al., 2022); hadrosauroids (Godefroit et al., 2012; Ostrom, 1961); *Hypsilophodon foxii* (Galton, 1989); *Iguanodon bernissartensis* (Norman, 1980); *Rhabdodon* sp. (Chanthasit, 2010); *Tenontosaurus tilletti* (Thomas, 2015)). The anterodorsally sloping floor of the nuchal fossa also occurs in *Heterodontosaurus tucki* (Norman et al., 2011), indicating that the posteriorly vertical aspect of the supraoccipital in *Muttaburrasaurus langdoni* is derived.

Paired, transversely narrow nuchal fossae, reported in the supraoccipital of *Ouranosaurus nigeriensis* Taquet, 1976, appear to be divided by a relatively vertical nuchal crest, as in *Muttaburrasaurus langdoni*. In addition, the parietals of *Ouranosaurus nigeriensis* appear to roof the nuchal fossa, as in *Muttaburrasaurus langdoni*, without the squamosals; thus, differing from Styracosterna. In these aspects, the nuchal fossa of *Ouranosaurus nigeriensis* is comparable to the superior sub-fossa in *Muttaburrasaurus langdoni*. However, divided superior and inferior sub-fossae are not described in *Ouranosaurus nigeriensis*, although, it is notable that the dorsoventrally deep region, identified as the exoccipitals in *Ouranosaurus nigeriensis* Taquet, 1976, resemble the surfaces for muscular attachment on the supraoccipital of *Muttaburrasaurus langdoni*. Perhaps, the supraoccipital in *Ouranosaurus nigeriensis* extends to this region, as Taquet (1976) noted that sutural definition between the bones of occiput could not be adequately differentiated owing to fusion.

Differing from *Muttaburrasaurus langdoni*, a single, poorly developed nuchal fossa, lacking a distinct nuchal shelf, is apparent in *Fostoria dhimbangunmal* (LRF 3050). The fossa in *Fostoria dhimbangunmal* slopes anteroventrally in the direction of the foramen magnum; thus, differing from the relatively vertical orientation of the fossae in *Muttaburrasaurus langdoni* and opposite to the direction of the slope in most other ornithopods, as mentioned above. Squamosal bosses are apparently absent in *Fostoria dhimbangunmal,* differing from the pronounced bosses in *Muttaburrasaurus langdoni*. In *Fostoria dhimbangunmal*, a sinuous, near vertical sutural contact with the otoccipital occurs in posterior view (Bell et al., 2019a, fig. 3G, H), differing from the ventromedially sloping contact in *Muttaburrasaurus langdoni*, shared with *Gasparinisaura cincosaltensis* Coria & Salgado, 1996, *Hypsilophodon foxii* (Galton, 1974) and *Rhabdodon* sp. (Chanthasit, 2010). As in *Fostoria dhimbangunmal* (LRF 3050) and differing from *Muttaburrasaurus langdoni*, the single nuchal fossa in *Gasparinisaura cincosaltensis* (MUCPv-208; M. Herne, pers. obs., 2008) is shallow and lacks a distinct nuchal shelf. Squamosal bosses are present on the supraoccipital of *Gasparinisaura cincosaltensis* (MUCPv-208; M. Herne, pers. obs., 2008), but in comparison to *Muttaburrasaurus langdoni*, are poorly developed. Pronounced squamosal bosses are absent in most ornithopods (*e.g.*, *Camptosaurus dispar* (Gilmore, 1909); *Hypsilophodon foxii* (Galton, 1974); *Iguanodon bernissartensis* (Norman, 1980); *Edmontosaurus regalis* (Xing, Mallon & Currie, 2017)) and in this aspect are possibly unique in *Muttaburrasaurus langdoni*. Squamosal bosses are present in *Tenontosaurus tilletti* (Thomas, 2015, fig. 1), but not as pronounced as in *Muttaburrasaurus langdoni*. As in *Muttaburrasaurus langdoni*, roughly vertical nuchal surfaces are developed in the early diverging neornithischians, *Lesothosaurus diagnosticus* and *Haya griva* (Barta & Norell, 2021; Sereno, 1991); however, how this condition in early diverging neornithischians relate to the lineage of *Muttaburrasaurus langdoni* is beyond assessment in this current study. From our present comparisons, supraoccipital morphology in *Muttaburrasaurus langdoni* substantially differs from all other ornithopods.

#### Otoccipital

**Figure 38:**
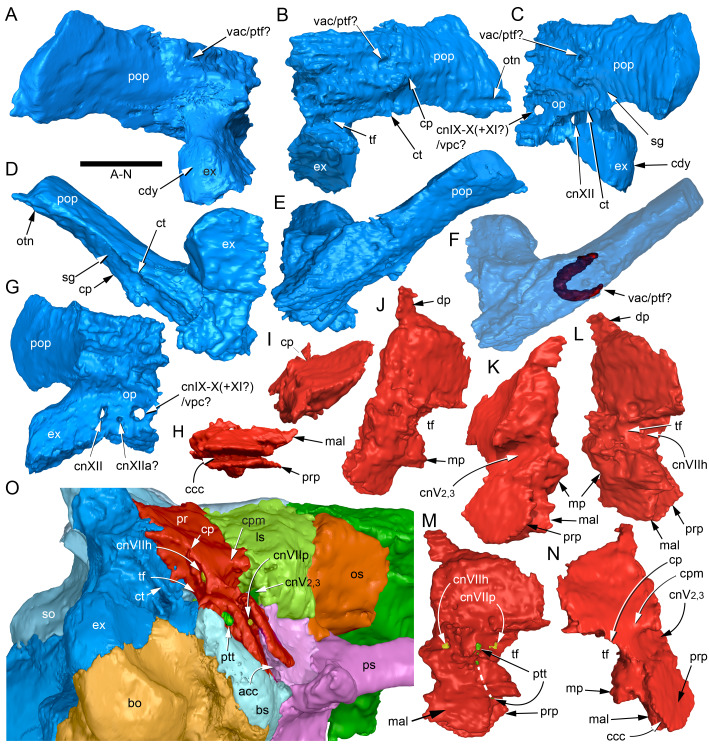
Volume rendered models of the *Muttaburrasaurus langdoni* (QMF6140) neurocranium and neurocrania. (A–G) Left otoccipital (exoccipital, opisthotic, paraoccipital process complex) in (A) posterior, (B) anterior, (C) lateral, (D) ventral, (E) dorsal, (F) dorsal (translucent to show internal occipital artery) and (G) medial views. (H–N) Right prootic in (H) ventral, (I) dorsal, (J) medial, (K) lateral (L) anteromedial, (M) posteromedial and (N) anterolateral views. (O) Neurocranium in right lateroventral (slightly posterior) view. Abbreviations: acc, (foramen of) cerebral carotid artery; aoc, canal of the occipital artery; bo, basioccipital; bs, basisphenoid; ccc, cerebral carotid canal; cdy, condylid; cn#, cranial nerve and number (a, accessory; h, hyomandibular branch; p, palatine branch); cp, *crista prootica*; cpm, *crista prootica media*; ct, *crista tuberalis*; dp, dorsal process; ex, exoccipital; for, foramen; ls, laterosphenoid; mal, medial ala of the prootic; mp, medial process; op, opisthotic; os, orbitosphenoid; otn, otic notch; pop, paroccipital process; pr, prootic; prp, prootic pendant; ps, parasphenoid; ptf?, posttemporal foramen?; ptt, foramen of the pharyngotympanic tube (green); sg, stapedial groove; so, supraoccipital; tf, tympanic fossa; vac, vascular canal; vpc, (foramen of) posterior cephalic vein?. Scale bar equals five cm. MorphoSource DOI: 10.17602/M2/M788152; 10.17602/M2/M788149; 10.17602/M2/M788155; 10.17602/M2/M788158.

**Figure 39:**
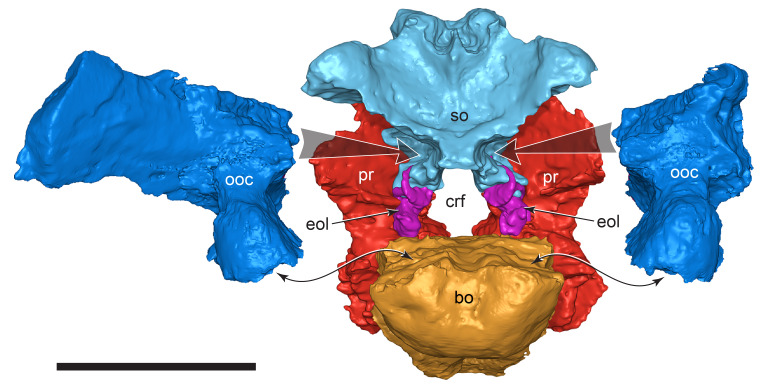
Volume rendered model of *Muttaburrasaurus langdoni* (QMF6140) posterior neurocranium in exploded view (parietals removed). Large arrows indicate direction of otoccipital articulation within the posterolaterally-oriented prootic-supraoccipital fossa. Small curved arrows indicate sutural surface between the otoccipital (opisthotic-exoccipital) and basioccipital. Abbreviations: bo, basioccipital; crf, cranial fossa; eol, endosseous labyrinth; ooc, otoccipital; pr, prootic; so, supraoccipital. Scale bar equals 10 cm.

The opisthotics, paroccipital processes and exoccipitals are indistinguishably fused, forming an otoccipital (Figs. 38A–38G). Notably, the paroccipital processes have been shown in extant Squamata to ossify from extension of the *crista prootica* of the opisthotic (Jerez, Sánchez-Martínez & Guerra-Fuentes, 2015). Norman (1980) further suggested that the paroccipital processes and opisthotics in *Iguanodon bernissartensis* were a single ossification centre. Although the margins between the elements of the otoccipital in *Muttaburrasaurus langdoni* cannot be delimited, they are described here in terms of regions. The otoccipital forms the posterolateral portion of the neurocranium, posterior to the prootics, lateral and ventral to the supraoccipital and dorsal to the basioccipital (Figs. 34, 37, 38A–38G). The otoccipital inserts in a wedge-shaped, lateroposterially oriented fossa formed by the prootic and supraoccipital (Fig. 39). Along with the basioccipital ventrally, the opisthotic housed the myelencephalic region of the brain, including much of the endosseous labyrinth (described in detail under “Palaeoneurology” below) and the passages for cranial nerves (cn) IX–XII. The opisthotic and anterior-most end of the paroccipital process, insert in a deep, wedge-shaped, posterolaterally oriented fossa formed by the supraoccipital and prootic (Fig. 37). The ventral margins of the opisthotics-exoccipitals are mostly ankylosed with the basioccipital, making these margins difficult to identify with certainty. Nevertheless, the knitted margin between these elements is provisionally identified (Fig. 37). The dorsal surface of the opisthotic tightly abuts the supraoccipital, with some of the margin suggesting fusion. Laterally, the *crista prootica* continues along the anterior surface of the paroccipital process (Fig. 34A), noting that the latter element was potentially formed from ossification of an extended *crista prootica* (Jerez, Sánchez-Martínez & Guerra-Fuentes, 2015).

Tracking the sutural margins in the otic region from the CT imagery was challenging. Nevertheless, the margins identified indicate that the opisthotic shares the otic capsule with the prootic and supraoccipital (Fig. 37). The otic vestibular regions of the saccule, utricle and posterior ends of the posterior and lateral semi-circular canals are within the opisthotic. The opisthotic forms the posterior wall of the tympanic fossa (= stapedial recess) (Figs. 34A, 34C, 38B). The *crista tuberalis* (= *crista metotica*, or metotic strut; see Witmer & Ridgely, 2008b) extends in a posterodorsally directed arc laterally on the opisthotic from its ventral contact with the basal tubera of the basisphenoid and basioccipital to interject between the metotic and tympanic fossae, and then continues along the ventral edge of the paroccipital process (Figs. 34A, 34C, 38B–38D, 38N). The channel formed on the paroccipital process between the *crista tuberalis* and *crista prootica* constitutes the stapedial groove extending laterally from the tympanic fossa (Figs. 34A, 38C, 38D). A distinct notch at the lateral end of the stapedial groove is interpreted as the otic notch that supported the cartilaginous middle ear tube containing the stapes (Figs. 5A, 38B, 38D). It is notable that the *crista tuberalis* identified in *Tenontosaurus dossi* by Winkler, Murry & Jacobs (1997), is equivalent to the position of this feature we identify in *Muttaburrasaurus langdoni*. However, the *crista tuberalis* identified in *Tenontosaurus tilletti* by Thomas (2015) is equavalent to the *crista prootica* we identify in *Muttaburrasaurus langdoni*.

Three cranial nerve foramina pierce the medial wall of the otoccipital in a roughly horizontal line. The anterior-most opening, which is also the largest, marks the canal of the *fissura metotica* that divides into two branches laterally. The anterior-most branch merges with an opening to the *recessus scalae tympani*, anterior to the *crista tuberalis*, and exits in the metotic foramen within the tympanic fossa (Figs. 34D, 37A). The posterior branch continues as the vagus canal posterior to the *crista tuberalis*. Confusing terminology has been applied to the divided *fissura metotica* (for detailed discussions see (Gower & Weber, 1998; Rieppel, 1985; Sobral, Hipsley & Müller, 2012); however, it is clear that this structure allowed the combined passage of the glossopharyngeal nerve (cnIX), vagus nerve (cnX) and possibly the accessory nerve (cnXI), along with the posterior cephalic vein (Gower & Weber, 1998; Rieppel, 1985) (Figs. 34D, 37). The glossopharyngeal (cnIX), vagus (cnX) and accessory (cn XI) nerves, potentially separated again at the *recessus scalae tympani* (Fig. 37). The *fissura metotica* in *Muttaburrasaurus langdoni* was likely invaded anteriorly by a perilymphatic duct/saccule from the vestibule through the *foramen perilymphaticum*, which led to the *fenestra pseudorotunda* within the *recessus scalae tympani* (= *apertura lateralis recessus scalae tympani*) at the medial end of the tympanic fossa (see Gower & Weber, 1998, pp. 381, 382; Rieppel, 1985) (Fig. 37A). The glossopharyngeal nerve likely coursed into the metotic foramen alongside the *fenestra pseudorotunda* (Figs. 34, 37, 38C) (Rieppel, 1985)—morphology likely occurring in all other ornithopods (Sobral, Hipsley & Müller, 2012; Thomas, 2015). The vagus nerve (cn X) and accessory nerve (cn XI, if it was present), along with the posterior cephalic vein, coursed through the anterior-most foramen posterior to the *crista tuberalis* (*i.e.,* the vagus canal; Barker et al., 2023; Bever et al., 2013; Rieppel, 1985; Sampson & Witmer, 2007; Sobral, Hipsley & Müller, 2012; Thomas, 2015; Xing, Mallon & Currie, 2017) (Fig. 37). Two rami of the hypoglossal nerve (cn XII) coursed through the two posterior-most foramina (Figs. 34 and 37, 38C), while the cnXI could have coursed through the anterior-most of these two foramina (*e.g.*, Godefroit et al., 2012).

The exoccipitals laterally wall the foramen magnum and are prevented from midline contact by the supraoccipital (Figs. 34, 38A–38G, 38O). In derived ornithopods, the exoccipitals prevent supraoccipital contribution to the foramen magnum (see “Supraoccipital” above). The hemispherical condylids of the exoccipitals form the dorsal-most 45% of the occipital condyle. The paroccipital processes buttress the rear part of the cranium and anchor the lateral flexor muscles of the cranium to the neck (potentially *m. obliquus capitis magnus* and *m. transversalis capitis* in Ostrom (1961) and Tsuihiji (2010)) and/or *m. longissimus capitus superficialis*, as assessed in Theropoda by Snively & Russell, 2007). Although the left element is almost complete, the distal ends of both paroccipital processes are missing (Figs. 7B, 33 and 38). The paroccipital process projects posterolaterally and slightly dorsally and lacks the typically curved ventral margin found in styracosternans. The end of the paroccipital process is only moderately expanded dorsally and ventrally, as in *Fostoria dhimbangunmal* (Bell et al., 2019a) and *Rhabdodon* sp. (Chanthasit, 2010). The distal end of the process is also expanded in *Camptosaurus dispar* (Carpenter & Lamanna, 2015; Gilmore, 1909), although to a greater degree. Unlike *Tenontosaurus tilletti* (Thomas, 2015) and styracosternans (*e.g.*, Norman, 1980; Ostrom, 1961; Taquet, 1976), ventral extension of the distal end of the paroccipital process is absent.

Posteriorly on the dorsal margin of the paroccipital process, a notch occurs close to the supraoccipital margin (Figs. 33D, 38A). Within the notch, a foramen is present, from which extends a strongly U-shaped canal (in dorsal view) within and through the paraoccipital process to the mandibular adductor chamber (Figs. 34C, 38A–38C, 38F). From the foramen in the adductor chamber, a posterolaterally directed groove continues along the anterior surface of the paraoccipital process. A canal also extends into the paroccipital process in the ornithopods *Camptosaurus dispar* (USNM 5473, (Gilmore, 1909)), *Hypsilophodon foxii* (Galton, 1974) and *Gasparinisaura cincosaltensis* (Coria & Salgado, 1996), the early diverging neornithischian *Lesothosaurus diagnosticus* (Sereno, 1991) and the early diverging ornithischian *Heterodontosaurus tucki* (Norman et al., 2011), suggesting the plesiomorphic state for an ornithischian. This canal has been considered as the remnant of the posttemporal fenestra in at least *Hypsilophodon foxii* (Galton, 1974) and *Lesothosaurus diagnosticus* (Sereno, 1991). Norman et al. (2011), less committedly considered the canal in *Heterodontosaurus tucki* as the “occipital vascular canal”.

Differing from the internal canal through the paroccipital process in *Muttaburrasaurus langdoni*, a notch/sulcus crosses the dorsal margin of the paroccipital process in *Dryosaurus elderae* (Carpenter & Lamanna, 2015), *Dysalotosaurus lettowvorbecki* (Sobral, Hipsley & Müller, 2012), the early diverging neornithischians, *Thescelosaurus assiniboiensis* and *T*. *neglectus* (Boyd, 2014; Brown, Boyd & Russell, 2011) and an immature individual of *Camptosaurus dispar* (Carpenter & Lamanna, 2015). This dorsal notch has been assessed in these taxa as the passage of the *vena capitis dorsalis*. Following an analysis of the exit of a lateral branch of the *vena capitis dorsalis* into the mandibular adductor chamber, Sobral, Hipsley & Müller (2012) more specifically considered that the dorsal notch accommodated the lateral branch of the *vena capitis dorsalis*, with the main trunk of the vein running through the supraoccipital and exiting posteriorly on the occiput. Notably, branching of the *vena capitis dorsalis* also occurs in *Muttaburrasaurus langdoni* (see under “Supraoccipital” above).

While it is possible that the lateral branch of the *vena capitis dorsalis* coursed through the paroccipital process in *Muttaburrasaurus langdoni*, the strong anterolateral orientation of the canal on the anterior surface of the paroccipital process is not immediately conducive to such a connection. Furthermore, according to Langer (2004), the occipital branch of the ophthalmic artery passed through the posttemporal fenestra in basal saurischians. Possibly the canal in *Muttaburrasaurus langdoni* is the remnant of the posttemporal fenestra. However, given our uncertainty of the function and homology of the paroccipital foramen in *Muttaburrasaurus langdoni*, we use the term “occipital vascular canal” (following Norman et al., 2011). In rhabdodontomorphs, the posttemporal fenestra passes through the squamosal, with that location regarded as synapomorphic for the clade (Zanno et al., 2023). If the vascular canal through the paroccipital process in *Muttaburrasaurus langdoni* is considered homologous to the fenestra through the squamosal of rhabdodontomorphs, then the lineage of *Muttaburrasaurus langdoni* is separable from the latter clade in that aspect.

#### Prootic

The prootic is a complex, plate-like, posterodorsally-anteroventrally-elongate bone forming the anterior part of the otoccipital region and the mid-lateral region of the neurocranium (Figs. 33 and 34, 38H–38O, 39). It abuts the laterosphenoid anteriorly, the basisphenoid ventrally and the supraoccipital both dorsally and anteromedially. A narrow dorsal process inserts between the parietal and supraoccipital and contacts the anterior-most end of the medial process of the squamosal (Figs. 5, 6B, 33A, 34A–34B, 38J–38N). The prootic forms the posterior wall of the foramen for the maxillary and mandibular rami of the trigeminal nerve (cnV_2-3_), being the largest of the cranial nerve foramina, and forms the anterior margin of the tympanic fossa (Figs. 34, 38K, 38N, 38O). Laterally, the *crista prootica* (= otosphenoidal crest: Maryańska & Osmólska, 1974; Sampson & Witmer, 2007) extends in a posterodorsally directed arc from the prootic pendant (see further below) to the posterior end of the prootic, roofing the tympanic fossa (Figs. 34A–34C, 38K, 38N, 38O). The *crista prootica* continues posteriorly on the otoccipital (see “Otoccipital” above).

It is notable that the locations of the *crista prootica* identified here, as well as the *crista tuberalis* (see “Otoccipital” above), differ from their placement in *Tenontosaurus tilletti* by Thomas (2015). The *crista prootica* in *Tenontosaurus tilletti* (*sensu* Thomas, 2015) and in *Dysalotosaurus lettowvorbecki* (*sensu* Sobral, Hipsley & Müller, 2012) were identified as the horizontal ridge dividing the dorsal and ventral halves of the prootic. This ridge in *Tenontosaurus* spp is especially prominent, where it forms a deep shelf overhanging the neurocranial openings ventrally in the prootic and opisthotic (Thomas, 2015; Winkler, Murry & Jacobs, 1997). The equivalent ridge in *Muttaburrasaurus langdoni* is weakly developed. Thus, a terminological issue is apparent. The relatively subdued and unidentified crista on the neurocranium of *Tenontosaurus tilletti* (*sensu* Thomas, 2015, fig. 35, marked as “?”), is the more protrusive ridge we identify in *Muttaburrasaurus langdoni* as the *crista prootica* bordering the tympanic fossa, as in other dinosaurs (*e.g.*, Marsh & Rowe, 2020, fig. 56.1,2; = otosphenoidal crest in Maryańska & Osmólska, 1974 and Sampson & Witmer, 2007, fig. 14A) and more generally in Lepidosauria (Paulina-Carabajal et al., 2023), posteroventral to the trigeminal nerve foramen, dorsal to the tympanic fossa and dorsolaterally overlying the canal and foramina of the facial nerve (cnVII). The location and arcuate form of the *crista prootica* on the prootic of *Muttaburrasaurus langdoni* is comparable to the form described in Pachycephalosauridae (Maryańska & Osmólska, 1974). It is notable that the *crista prootica* and *crista alaris* identified in *Dysalotosaurus lettowvorbecki* by Sobral, Hipsley & Müller (2012), appear to be co-termed for the same feature, which, in its anterior part, roofs the lateral foramen of the trigeminal nerve (cnV_2,3_). The equivalent horizontal crest in *Muttaburrasaurus langdoni* is the posterior continuation of the *crista antotica* from the laterosphenoid (see further below). This horizontal crest dividing the prootic into dorsal and ventral halves was unnamed in Lepidosauria (following Paulina-Carabajal et al., 2023), but is termed here for clarity, ‘*crista prootica media*’ (Figs. 34A, 38N, 38O).

**Figure 40:**
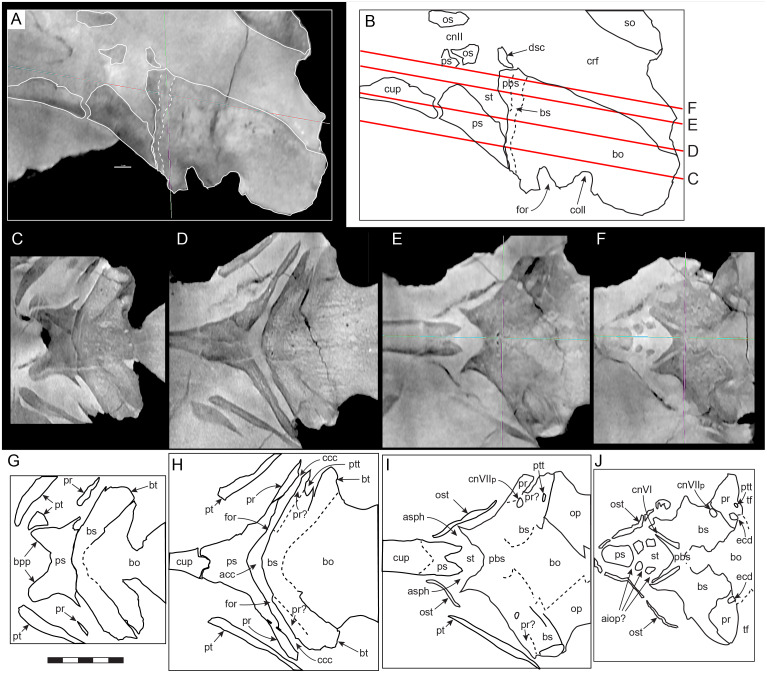
Radiographic sections of the *Muttaburrasaurus langdoni* (QMF6140) posteroventral neurocranium. (A, B) Sagittal radiograph (A) through posteroventral neurocranium and (B) explanatory schematic. (C–F) Dorsal radiographs corresponding to the planes (red lines) in B. (G–J) Explanatory schematics for C–F. Dashed lines in A, B, G–J indicate approximate locations of ankylosed or uncertain margins. Abbreviations: acc, void for the cerebral carotid artery (within the *sella turcica*); aiop, foramina and slot-like canals of the internal ophthalmic artery branches?; asph, foramen of the sphenoidal artery; bo, basioccipital; bpp, basipterygoid process; bs, basisphenoid; bt, basal tubera; coll, collum; ccc, cerebral carotid canal; cn#, cranial nerve and number (p, palatine branch); crf, cranial fossa; cup, cultriform process of parasphenoid; dsc, *dorsum sellae crista*; ecd, endosseous cochlear duct; for, foramen; op, opisthotic; os, orbitosphenoid; ost, possible mineralised osteoid; pbs, parabasisphenoid; pr, prootic; ps, parasphenoid; pt, pterygoid; ptt, foramen of the pharyngotympanic tube; so, supraoccipital; st, *sella turcica*; tf, tympanic fossa. Scale bar increments equal one cm.

Complete differentiation between the prootic and the basisphenoid was limited, possibly owing to partial fusion between the bones and/or the limits of CT scan resolution. In ornithischians, a roughly horizontal margin typically occurs between the ventral base of the prootic and the dorsal surface of the basisphenoid. However, close examination of the CT imagery indicates that the prootic in the holotype forms two parallel alae that extend ventrally, anterolateral to the basisphenoid (Figs. 38H–38O). The medial-most of these two alae (Figs. 33B, 33C, 34A–34C, ‘mal’ in Figs. 38H, 38J–38O) tightly abuts the basisphenoid. The medial ala was difficult to differentiate from the latter bone in places on the left and right sides; thus, its identification as a ventral extension of the prootic is provisional. The lateral-most of the two alae is identified as the preotic pendant (‘prp’ in Figs. 38H, 38J–38O). Among ornithischians, a wing-like preotic pendant (= ala basisphenoid and alar process in sauropsids: Holliday, 2009; Oelrich, 1956) occurs in some derived styracosternans, but reportedly formed from the basisphenoid (*e.g.*, Holliday, 2009, fig. 5A, 5B; Prieto-Márquez, 2010a; Taquet, 1976; Thomas, 2015; Xing, Mallon & Currie, 2017). However, a ventrally extending process of the prootic occurs in *Prosaurolophus blackfeetensis* (Horner, 1992) and *Transylvanosaurus platycephalus* (Augustin et al., 2022), although the process in these taxa is comparatively robust, rather than being sheet-like in *Muttaburrasaurus langdoni*. Nevertheless, the preotic pendant in *Muttaburrasaurus langdoni* is comparable to the ventrally extending alae/process of the prootic reported in the hadrosaur, *Prosaurolophus blackfeetensis* (Horner, 1992, although broken distally) and the hadrosauroid, *Transylvanosaurus platycephalus* (= ala process in Augustin et al., 2022). A ventrally extending process of the prootic was also noted in *Dysalotosaurus lettowvorbecki* by Galton (1989) and possibly occurs in the basal styracosternan *Ouranosaurus nigeriensis* Taquet, 1976 (fig. 14), noting that Taquet was unable to distinguish many of the margins between the neurocrania on the *Ouranosaurus nigeriensis* holotype. A similar ventrally extending ala of the prootic, termed the “prootic plate”, was reported in Pachycephalosauridae (Maryańska & Osmólska, 1974). The preotic pendant appears more extensively developed in *Muttaburrasaurus langdoni* than in other ornithopods. Furthermore, the preotic pendant in *Muttaburrasaurus langdoni* closely contacts a mating ala on the parabasisphenoid (most likely formed by the parasphenoid, see in “Parabasisphenoid” below); thus, forming a bilaminate structure. The mating alae of the prootic and parabasisphenoid form the anterolateral wall of a narrow, slot-like canal for the cerebral carotid artery (= vidian canal of some authors) (‘ccc’ in Figs. 33B, 34B, 34C, 38H, 38N, 38O)—morphology comparable to that described in Pachycephalosauridae by Maryańska & Osmólska (1974), although contribution to the bilaminate structure of the preotic pendant in Pachycephalosauridae is from the basisphenoid, rather than from the parasphenoid in *Muttaburrasaurus langdoni*. The posterior wall of the cerebral carotid canal in *Muttaburrasaurus langdoni* is formed by the posteromedial ala of the prootic (‘mal’ in 38H, 38J–38O). The lateral edge of the preotic pendant lies medial to the quadrate ala of the pterygoid (Fig. 40), as similarly reported in pachycephalosaurs (Maryańska & Osmólska, 1974). The lateral surface of the preotic pendant potentially formed the origin of the superior part of the *m. protractor pterygoideus*, as in other sauropsids (Holliday, 2009).

The prootic forms the anterior and lateral surfaces of the osseus labyrinth (Figs. 36A, 37, 38, 39A) (described in detail in “Palaeoneurology” below). The anteroventral region of the osseous labyrinth and the internal auditory meatus carrying the proximal rami of the facial and vestibulocochlea cranial nerves (cnVII and cnVIII, respectively) from the brain are within the prootic (Figs. 34D, 37, 38H, 38N, 38O; see “Cranial nerves” below). The single medial foramen for the facial nerve (cnVII) branches within the prootic to form two rami—an anteroventrally directed palatine ramus (cnVII_p_) and posterolaterally directed hyomandibular ramus (cnVII_h_). The foramen for the palatine ramus (cnVII_p_) exits the braincase through the dorsal surface of the cerebral carotid canal, and the hyomandibular ramus (cnVII_h_) exits from the anterior wall of the tympanic fossa (Figs. 37, 38M, 38O, 40). A canal coursing from the proximal end of the tympanic fossa, close to the vestibule, to a lateroventral notch between the prootic and basisphenoid, is interpreted as the pharyngotympanic tube (= eustachian tube in mammals) (‘ptt’ in Figs. 38O, 40C, 40D, 40E).

#### Laterosphenoid

**Figure 41:**
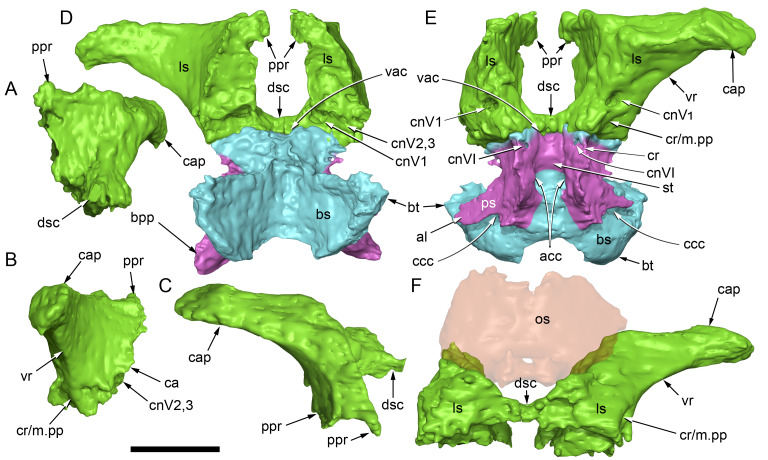
Volume rendered models of the *Muttaburrasaurus langdoni* (QMF6140) anterior neurocranium and neurocrania. (A–C) Left laterosphenoid in (A) medial, (B) lateral and (C) dorsal views. (D) Laterosphenoids articulated with parabasisphenoid in posterior view, with anterior part of parasphenoid (purple) removed. (E) Laterosphenoids articulated with parabasisphenoid in anterior view, with anterior part of parasphenoid (purple) removed to show *sella turcica*. (F) Laterosphenoids articulated with orbitosphenoid (semi-transparent) in ventral view. Abbreviations: acc, (foramen of) cerebral carotid artery; al, ala; asp, ascending process; bo, basioccipital; bpp, basipterygoid process; bs, basisphenoid; bt, basal tubera; ca, *crista antotica*; cap, capitulum; ccc, cerebral carotid canal; cn#, cranial nerve and number; cr, crista; crf, cranial fossa; dsc, *dorsum sellae crista*; fos, fossa; ls, laterosphenoid; m.pp, *musculus protractor pterygoideus*; os, orbitosphenoid; ppr, posterior process; ps, parasphenoid; st, *sella turcica*; vac, vascular canal; vr, ventral ridge. Scale bar equals five cm. MorphoSource DOI: 10.17602/M2/M789996; 10.17602/M2/M789999; 10.17602/M2/M788143.

As a chondrocranial element of the sphenethmoid, the laterosphenoid forms the anterolateral region of the neurocranium (Figs. 33B, 33C, 34, 38O, 41). The bone is roughly L-shaped in dorsoventral view and triangular in lateral and anteroposterior views (Fig. 41). The thickened body of the laterosphenoid abuts the orbitosphenoid anteriorly, the prootic posteriorly and the basisphenoid ventrally (Figs. 33 and 34, 36B, 36C, 38O, 41). The laterally projecting capitate process subtends the posterior margin of the frontal and the anterolateral process of the parietal. Medially, a small dorsal process projects posteriorly to contact the supraoccipital (Fig. 41). The sutural margins between the laterosphenoids and the prootics are difficult to differentiate in some places and are provisional. Laterally, the *crista antotica* forms a sub-horizontal crest dorsal to the enlarged foramen for rami two and three of the trigeminal nerve (cnV_2,3_) (Figs. 34, 41B). The crest is the anterior continuation of that on the prootic dorsal to cnV_2,3_, which has been termed herein the *crista prootica media* (see “Prootic” above). Laterally, a dorsoventrally oriented ridge, termed here the ‘ventral ridge’, extends from the ventral base of the laterosphenoid to the capitate process, dividing the laterosphenoid into lateral and anterior surfaces (Fig. 41). In addition, an obliquely oriented crista at the ventral base of the laterosphenoid forms a shelf that roofs a shallow recess that extends ventrally onto the parabasisphenoid (Figs. 34A, 41). The recess is the likely origin of the *m. protractor pterygoideus* (based on (Holliday, 2009)). The robust, laterally tapering capitate process forms an anterolaterally facing flange at its distal end that locates in a loose fossa on the postorbital (Figs. 29B, 41). A gap of ∼13 mm between the capitate process and the fossa surface, suggests an interjecting cartilage could have allowed metakinetic flexibility in this region (see (Sues, 1980); although see “Cranial kinematics and mastication” in “Results and Discussion”). Narrow ventromedial processes on each side traverse the floor of the cranial fossa at the posterior margin of the *sella turcica* (= pituitary/hypophyseal fossa) to meet at the midline (‘dsc’ in Figs. 34D, 40A, 41). These paired processes constitute the *dorsum sellae crista*, as in *Tenontosaurus tilletti* (Thomas, 2015). The *dorsum sellae crista* projects ∼10.0 mm dorsally into the cranial fossa and overlies the parabasisphenoid (see further under “Basisphenoid” below). A small vascular canal passes ventral to the *dorsum sellae crista* on each side, communicating between the cranial fossa posteriorly to the *dorsum sellae crista* and the *sella turcica* anteriorly (Figs. 41D, 41E). The laterosphenoid forms the anterior margin of the large lateral foramen for the combined maxillary and mandibular rami of cnV_2,3_ (Figs. 34A, 34C, 37B, 41D–41B). A foramen extends anteriorly within the laterosphenoid for the ophthalmic ramus of the trigeminal nerve (cnV_1_) (Figs. 33C, 37B, 41D–41E), therein differing from nearly all other ornithopods currently reported, where the ophthalmic ramus typically extends in a shallow groove or slot-like foramen on the lateral surface of the laterosphenoid (*e.g.*, Bolotsky & Godefroit, 2004; Galton, 1989; Godefroit, Bolotsky & Van Itterbeeck, 2004; Holliday, 2009; Prieto-Marquez, 2010a; Thomas, 2015; Xing, Mallon & Currie, 2017). Among ornithischians, enclosure of cnV_1_ is evident in *Lambeosaurus magnicristatus* (Evans & Reisz, 2007) and at least some ankylosaurians and ceratopsians. Among theropods, enclosure and separation of cnV_1_ by the laterosphenoid is variable (*e.g.*, Brusatte et al., 2010; Holliday, 2009; Paulina-Carabajal, Ezcurra & Novas, 2019; Sampson & Witmer, 2007). However, cnV_1_ appears to pass externally (laterally) on the laterosphenoid in at least the early diverging neotheropodan *Zupaysaurus rougieri* (Paulina-Carabajal, Ezcurra & Novas, 2019) and the early diverging ornithischians *Heterodontosaurus tucki* and *Lesothosaurus diagnosticus* (Norman et al., 2011; Porro, Witmer & Barrett, 2015), albeit inconclusively. Hence, osseous enclosure of cnV_1_ in the laterosphenoid of *Muttaburrasaurus langdoni* suggests the derived state for an ornithischian, potentially linked to the thickened condition of the neurocranial bones. Medially, the notch mid-way along the anterior margin is assessed as the opening for the trochlear nerve (cnIV) (shared with the orbitosphenoid) and the notch on the anterodorsal corner potentially carried the orbitocerebral vein (Fig. 34D), based on comparable locations in *Pachyrhinosaurus lakustai* (Witmer & Ridgely, 2008b). A short posterior process on the dorsomedial corner of the laterosphenoid contacts the supraoccipital posteriorly (Figs. 34, 41A–41E), as in *Tenontosaurus tilletti* (Thomas, 2015). Contact between the laterosphenoid and the supraoccipital is hidden laterally by the prootic, differing from *Hypsilophodon foxii* (Galton, 1974), *Tenontosaurus tilletti* (Thomas, 2015) and the droysaurids (Galton, 1983), where contact is apparent.

#### Basioccipital

**Figure 42:**
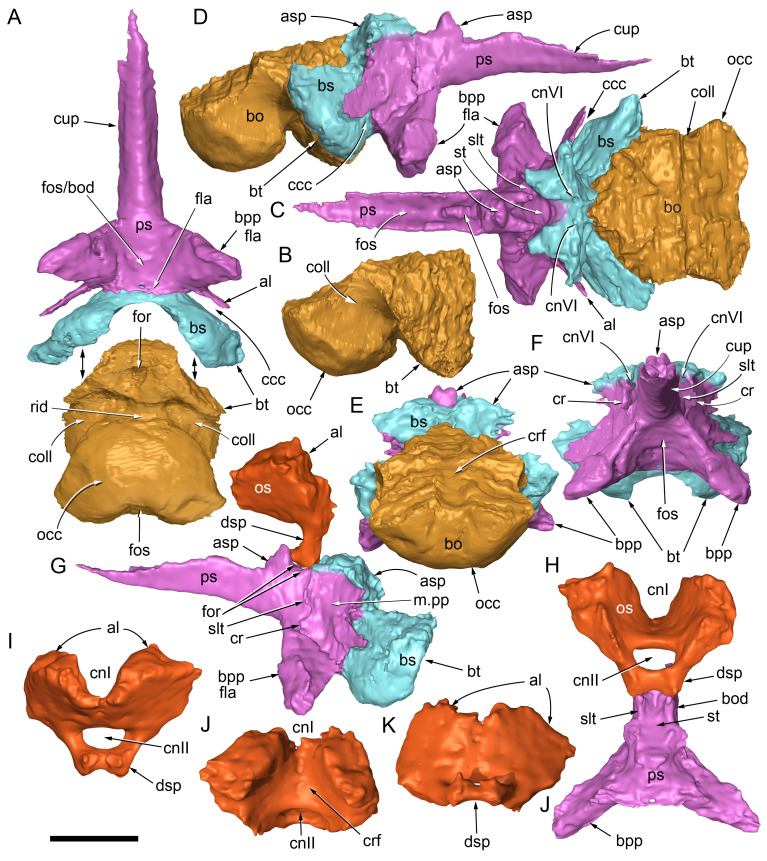
Volume rendered models of the *Muttaburrasaurus langdoni* (QMF6140) anterior and ventral neurocranium and neurocrania. (A) Parabasisphenoid and basioccipital, disarticulated, in ventral view. (B) Basioccipital in right lateral view. (C–E) Basioccipital and parabasisphenoid, articulated, in (C) dorsal, (D) right lateral and (E) posterior views. (F) Parabasisphenoid in anterior view. (G) Parabasisphenoid and orbitosphenoid, articulated, in left lateral view. (H) Parabasisphenoid and orbitosphenoid articulated in posterior view, with posterior part of parabasisphenoid removed to view *sella turcica*. (I–K) Orbitosphenoid in (I) anterior, (J) dorsal and (K) ventral views. Abbreviations: al, ala; asp, ascending process; bod, body; bpp, basipterygoid process; bo, basioccipital; bs, basisphenoid; bt, basal tubera; ccc, cerebral carotid canal; cn# (foramen of) cranial nerve and number; coll, collum; cr, crista; crf, cranial fossa; cup, cultriform process; dsp descending process; fla, flange; for, foramen; fos, fossa; m.pp, *musculus protractor pterygoideus*; occ, occipital condyle; os, orbitosphenoid; ps, parasphenoid; rid, ridge; slt, slot; st, *sella turcica*. Scale bar equals five cm. MorphoSource DOI: 10.17602/M2/M788146; 10.17602/M2/M788140; 10.17602/M2/M788143.

The basioccipital is a midline chondrocranial bone forming the robust posteroventral floor of the neurocranium (Figs. 33, 34, 39, 40, 42A–42E). The anterior margin is broadly wedge-shaped (= tongue-shaped of some authors) with a rounded apex in dorsoventral view that inserts in a complementary notch on the basisphenoid (Figs. 33B, 40, 42A, 42C); however, fusion between the basioccipital and basisphenoid is apparent, particularly medially where separation of the two bones is difficult to identify in the CT data. A similarly wedge-shaped anterior margin occurs in the dryomorphs, *Dryosaurus elderae* (Carpenter & Lamanna, 2015), *Camptosaurus dispar* (Gilmore, 1909) and *Cumnoria prestwichii* (Galton & Powell, 1980), the rhabdodontomorph, *Iani smithi* (Zanno et al., 2023), and most likely the elasmarian *Anabisetia saldiviai* Coria & Calvo, 2002. The wedge-shaped anterior profile differs from the relatively transverse margins in *Hypsilophodon foxii* (Galton, 1974), *Tenontosaurus tilletti* (Thomas, 2015), the hadrosaur *Edmontosaurus regalis* (Xing, Mallon & Currie, 2017), and the two unnamed rhabdodontids from the Haţeg Basin (Augustin et al., 2023) (see also “Basisphenoid” below). Wing-like anterolateral processes on the body of the basioccipital abut the basisphenoid, forming the posterior parts of the basal tubera (= sphenooccipital tubercles) (Figs. 40, 42A–42D). A ventrally concave sulcus broadly separates the basal tubera. The basal tubera border the anterior margins of the collum (neck) that extends to the occipital condyle. Ventrally, the body of the basioccipital anterior to the collum and medially within the sulcus between the basal tubera is thickened and the ventral surface is rugose (Fig. 42A). Viewed anteriorly, the body of the basioccipital is ventrally convex (Fig. 33C), differing from *Tenontosaurus tilletti* (Thomas, 2015), *Dryosaurus elderae* and *Camptosaurus dispar* (Carpenter & Lamanna, 2015, figs. 4, 7), *Dysalotosaurus lettowvorbecki* (Sobral, Hipsley & Müller, 2012), the early diverging ankylopollexian *Mantellisaurus atherfieldensis* ((Norman, 1986), Fig. 10), hadrosaurids (*e.g.*, Xing, Mallon & Currie, 2017, fig. 10) and the isolated basioccipital-basisphenoid of an indeterminate iguanodontian from Lightning Ridge (LRF 267; Bell et al., 2018), where the ventral surface forms a fossa, or is at least concave between the basal tubera (see further below). A blind foramen (= basioccipital foramen) ∼6 mm in diameter occurs ventrally on the body (Figs. 40, 42A), like that in *Tenontosaurus tilletti* (Thomas, 2015) but absent on the indeterminate iguanodontian basioccipital from Lightning Ridge (LRF 267; Bell et al., 2018). In addition, three, small (∼2 mm) ‘blind’ foramina occur ventrally in a transverse line on the collum near its anterior margin (not figured). These foramina extend dorsally roughly into the middle of the basioccipital body.

In lateral view, the collum is extremely short ventrally (22% of the occipital condyle length), but lengthens anteroposteriorly more dorsally because of the posterodorsally angled anterior margin of the occipital condyle (Figs. 42A, 42B, 42D). A broad, low ridge presents ventrally on the collum between the occipital condyle and the thickened anterior body of the basioccipital (Fig. 42A). This ridge is possibly equivalent to the median ridge reported in an unnamed Haţeg Basin rhabdodontid (Augustin et al., 2023, fig. 5c), but differs from the anteriorly pointing, wedge-shaped tuberosity in *Tenontosaurus tilletti* (Thomas, 2015) and the basioccipital tuberosity reported in *Iani smithi* Zanno et al., 2023. A sharp median crest occurs in this location on the collum in *Anabisetia saldiviai* Coria & Calvo, 2002 and *Camptosaurus dispar* (Gilmore, 1909). The ventral morphology of the collum distinctly differs from that in *Dryosaurus elderae*, where a distinct midline fossa extends anteriorly between the basal tubera and onto the basisphenoid (Carpenter & Lamanna, 2015), with similar morphology, although possibly less pronounced, in the elasmarian, *Anabisetia saldiviai* Coria & Calvo, 2002, the early diverging ankylopollexian, *Camptosaurus dispar* (Carpenter & Lamanna, 2015; Gilmore, 1909), the hadrosauroids, *Batyrosaurus roshdestvenskyi* Godefroit et al., 2012 and *Telmatosaurus transylvanicus* (Augustin et al., 2023) and the hadrosaurid *Edmontosaurus regalis* (Xing, Mallon & Currie, 2017). A ventral fossa on the collum also occurs in the rhabdodontomorph, *Iani smithi* (Zanno et al., 2023). The collum on the indeterminate iguanodontian from Lightning Ridge (LRF 267) is anteroposteriorly longer (Bell et al., 2018).

The basioccipital forms the ventral portion of the smoothly rounded occipital condyle (Figs. 33D, 34, 42A–42E), with the condylids of the exoccipitals forming the dorsal-most 45%. The occipital condyle is posteriorly and ventrally directed and reniform in posterior view (Figs. 34, 42A, 42E). Posteriorly, a shallow midline channel indents the occipital condyle leading from the foramen magnum. Dorsally, a shallow channel forms the medio-ventral surface of the cranial fossa (Fig. 41A). The basioccipital contributes ∼30% of the foramen magnum width. The otoccipitals connect along the digitate, dorsolaterally oriented sutural surfaces (Figs. 33D, 34, 39). More posteriorly, the margins between basioccipital and the otoccipitals are difficult to follow and appear fused. Furthermore, a tight groove presents laterally along the sutural margin between the bones, as originally reported (Bartholomai & Molnar, 1981). The distal end of the endosseous cochlear duct (ECD; (Witmer et al., 2008)) penetrates the dorsal surface of the basioccipital at the prootic margin (Fig. 40E).

#### Parabasisphenoid

In mature ornithopods, the basisphenoid and the parasphenoid typically fuse (*e.g.*, Norman, 1980); thus, forming a parabasisphenoid. However, from the CT imagery, a transverse sutural margin presents ventrally between the basisphenoid and parasphenoid, aligned with the posterior wall of the *sella turcica* (pituitary/hypophyseal fossa) (Figs. 40, 42A), as similarly reported in *Iguanodon bernissartensis* and *Mantellisaurus atherfieldensis* (Norman, 1980; Norman, 1986). More dorsally, the basisphenoid and parasphenoid appear ankylosed (see Figs. 40, 41E, 42D, 42F, 42G). The lack of complete fusion between the basisphenoid and parasphenoid could indicate immaturity in the individual. Here, the two bones are considered in terms of the parabasisphenoid, but their descriptions are separated for clarity.

*Basisphenoid*—The basisphenoid is a mediolaterally wide and anteroposteriorly short, midline chondrocranial bone forming the anterior floor of the neurocranium between the basioccipital and the parasphenoid (Figs. 33B–33D, 34, 38O, 40, 41D, 41E, 42A, 42C–42H). Generally, the body of the basisphenoid forms an anteroposteriorly short, median bony isthmus that wedges between the parasphenoid and the basioccipital. The basisphenoid and basioccipital show at least partial fusion, particularly medially (Fig. 40). Although tightly abutting and ankylosed, at least medially, the basisphenoid and basioccipital can be distinguished across their adjoining margins. Viewed dorsoventrally, a deep V-shaped sulcus posteriorly accommodates the basioccipital (Figs. 33B, 40, 41J, 41I), as in *Camptosaurus dispar* and *Dryosaurus elderae* (Carpenter & Lamanna, 2015, figs. 4C, 8C). The basisphenoid forms the pronounced anterior portions of the basal tubera, which closely abut the medial ala of the prootic on each side (Figs. 33B, 34A–34C, 40, 42A–42E; see also “Prootic” above). The posterior flanks of the basal tubera are formed by the basioccipital. The basal tubera thicken towards their lobate distal (ventral) ends. Viewed anteriorly, the ventral margin medially between the basal tubera is concave (Figs. 33C, 41E, 42F); however, the interjecting basioccipital renders the ventral margin as convex giving the appearance of a single basal tuber. Thus, unlike most dryomorphs, the ventral surface between the basal tubera lacks a depression or fossa (*e.g.*, Augustin et al., 2023; Carpenter & Lamanna, 2015; Godefroit et al., 1998; Godefroit et al., 2012; Norman, 1986; Sobral, Hipsley & Müller, 2012; Taquet, 1976; Xing, Mallon & Currie, 2017). Compared to *Muttaburrasaurus langdoni*, the basisphenoid in *Camptosaurus dispar* and the dryosaurids is more elongate (Carpenter & Lamanna, 2015, figs. 4, 7, 8; Sobral, Hipsley & Müller, 2012, fig. 1). The anterolateral faces of the basal tubera are partly flanked by the preotic pendants of the prootics (Figs. 34A–34C, 38O, 40, 42; see also “Prootic” above).

Dorsally, the ankylosed unit of the parabasisphenoid abuts the laterosphenoids and forms the posterior wall of the *sella turcica* (Figs. 40, 41D, 41E, 42C). Immediately posterior to the *sella turcica*, the *dorsum sellae cristae* of the laterosphenoids traverse the dorsal surface of the median isthmus. Posterior to the *dorsum sellae cristae*, a narrow, anteroposteriorly short channel dorsally on the basisphenoid contributes to the ventral floor of the cranial fossa. The channel is penetrated by foramina for the abducens nerve (cnVI; Figs. 33C, 40E, 41E, 42C, 42F). The canal for cnVI exits in foramina on the medial side of the anterolaterally oriented cristae on the anteriorly adjoining parasphenoid, as in *Prosaurolophus blackfeetensis* (Horner, 1992). The basisphenoid is typically considered to form the basipterygoid process in ornithopods. However, the process in *Muttaburrasaurus langdoni* appears to be formed predominantly by the parasphenoid (Figs. 33B, 34, 40B, 42), as suggested in *Iguanodon bernissartensis* and *Mantellisaurus atherfieldensis* (Norman, 1980; Norman, 1986), and a similar condition could occur in *Tenontosaurus tilletti* (Thomas, 2015). The basisphenoid contributes to the ventral portion of the foramen for the pharyngotympanic tube (see also “Prootic”) (Figs. 38O, 40D, 40E).

*Parasphenoid*—The parasphenoid (of the parabasisphenoid) is a midline, dermatocranial bone forming the anteroventral region of the neurocranium. From the central body of the parasphenoid, the cultriform process projects anteriorly, a small ascending process projects dorsally, the alae of the preotic pendant (= alar process or *crista alaris* of some authors) projects posterolaterally and the basipterygoid processes project lateroventrally (Figs. 33B, 33C, 34, 40, 42A, 42B–42G). In addition, cristae project anterolaterally on each side of the body, flanking anterolateral openings into the *sella turcica*. An equivalently oriented crista in the early diverging neornithischian, *Thescelosaurus neglectus*, proximally and lateral to the basipterygoid processes, was identified by Boyd (2014) as the preotic pendant; however, the preotic pendant in *Muttaburrasaurus langdoni* is more posteriorly positioned and posterolaterally oriented (see further below). The cultriform process appears to have formed from a separate centre of ossification than the body of the parasphenoid, as a distinct margin is apparent in the CT imagery (Figs. 40C, 40D). The process is triangular in transverse section with a deep channel formed dorsally, as in *Thescelosaurus neglectus* (Boyd, 2014) and the hadrosaurid *Parasaurolophis blackfeetensis* (Horner, 1992). In contrast, the dorsal surface in *Hypsilophodon foxii* and *Tenontosaurus tilletti* is convex (Galton, 1974; Thomas, 2015). Viewed laterally, the cultriform process is roughly parallel to the skull roof (Fig. 34). However, the anterior tip also recurves dorsally and a distinct kink occurs on the dorsal margin at two-thirds of the process length from the anterior end. Viewed laterally, the dorsal part of the parasphenoid body (*i.e.,* posterior to the cultriform process), together with the ascending process, accommodate the paired ventral tubercles formed at the base of the descending process of the orbitosphenoid (Fig. 42G). A small triangular ascending process is formed anterior to the ventral tubercles of the orbitosphenoid (Figs. 42C–42F). The parasphenoid body and anterolateral cristae form the anterior and lateral walls of the *sella turcica* (Figs. 40, 41E, 42B, 42C, 42E, 42F) and the posterior wall is formed by the parabasisphenoid. A slot formed on each side by the anterolateral cristae was the likely route of the paired sphenoidal arteries to the orbits after branching from the cerebral carotid arteries within the *sella turcica* (see Sampson & Witmer, 2007). Within the body of the parasphenoid, a fossa is formed anteriorly in the *sella turcica* that leads to two foramina on each side on the dorsal margin of the parasphenoid body, ventral to the ventral tubercles of the orbitosphenoid (Figs. 40E, 42G). These foramina, in addition to the anterolaterally oriented slots, were potentially for the paired internal ophthalmic arteries to the eyes and orbital muscles, as in birds (Porter & Witmer, 2016). Differing from *Muttaburrasaurus langdoni*, the body of the parasphenoid in *Dysalotosaurus lettowvorbecki* is poorly developed, resulting in a shallow *sella turcica*, and the cultriform process is ventrally positioned relative to the floor of the neurocranial fossa resulting in a distinct dorsal step (MB.R.1373: Galton, 1989, plate 2.8; Sobral, Hipsley & Müller, 2012, fig. 1). These ventrally depressed features of the parasphenoid in *Dysalotosaurus lettowvorbecki*, markedly differing from *Muttaburrasaurus langdoni*, are comparable to *Hypsilophodon foxii* (Galton, 1989, fig. 2B), with a similar condition apparent in *Camptosaurus dispar* and *Dryosaurus elderae* (Carpenter & Lamanna, 2015, fig. 4, 7). The dorsoventrally deep body of the parasphenoid in *Muttaburrasaurus langdoni*, rising dorsally to contact the orbitosphenoid, is comparable to *Ouranosaurus nigeriensis* Taquet, 1976 (fig. 14). The dorsal position of the cultriform process relative to the braincase floor and anterior enclosure of the *sella turcica* by the parasphenoid body in the early diverging neornithischian *Thescelosaurus neglectus* (Boyd, 2014, figs. 11, 13), is also comparable to *Muttaburrasaurus langdoni*. However, *Thescelosaurus neglectus* lacks an ossified orbitosphenoid (Boyd, 2014), as in *Hypsilophodon foxii* and *Parksosaurus warreni*, but it is present in *Dryosaurus elderae* (Carpenter & Lamanna, 2015, fig. 6). Differing from *Muttaburrasaurus langdoni*, the margin of parasphenoid and orbitosphenoid contact in styracosternans, such as *Bactrosaurus johnsoni*, *Iguanodon bernissartensis*, *Mantellisaurus atherfieldensis*, *Corythosaurus casuarius* and *Edmontosaurus regalis* is comparatively elongate, extending onto the cultriform process, and in many of these taxa, is anterodorsally inclined (Godefroit et al., 1998; Norman, 1980; Norman, 1986; Ostrom, 1961; Xing, Mallon & Currie, 2017). Of the styracosternans, the condition of the parasphenoid body and anteroposteriorly reduced orbitosphenoid contact in *Ouranosaurus nigeriensis* is comparable to *Muttaburrasaurus langdoni*. In contrast to *Muttaburrasaurus langdoni*, the *sella turcica* in *Tenontosaurus tilletti* was assessed by Thomas (2015) as ventral to the base of the ventral tubercles of the orbitosphenoid, which is free from parasphenoid contact. According to Thomas (2015), the pituitary and hypophysis within the *sella turcica* of *Tenontosaurus tilletti* would have been connected by the infundibulum through an opening between the ventral tubercles at the base of the orbitosphenoid. This condition in *Tenontosaurus tilletti* markedly differs from the *sella turcica* enclosed by the parasphenoid body in *Muttaburrasaurus langdoni* and possibly all styracosternans. The condition in *Tenontosaurus tilletti*, as reported, is perhaps part way between the anteriorly open *sella turcica* in *Hypsilophodon foxii* and the *sella* enclosed by the connecting parasphenoid and orbitosphenoid in more derived ornithopods, even though *Muttaburrasaurus langdoni* could represent an early diverging lineage in Ornithopoda, as recovered in the phylogenetic analysis by Fonseca et al. (2024b), and independently considered possible by us from new findings, although without a formal phylogenetic analysis.

The basipterygoid processes are assessed herein, as elements of the parasphenoid (Figs. 40B, 42A, 42C, 42D, 42F–42H), as proposed in *Iguanodon bernissartensis* and *Mantellisaurus atherfieldensis* (Norman, 1980; Norman, 1986) and suggested in *Tenontosaurus tilletti* (Thomas, 2015). In ornithopods, the basipterygoid processes are typically reported as formed by the basisphenoid; however, complete fusion between the parasphenoid and basisphenoid in somatically mature individuals could obfuscate the osteological origin of the processes. For example, Galton (1974) identified the parasphenoid in *Hypsilophodon foxii* as solely consisting of the cultriform process, rather than extending further posteriorly, which would have encompassed the basipterygoid processes. A similar identification was made by Horner (1992) for *Prosaurolophus blackfeetensis*. The basipterygoid process projects ventrolaterally and slightly anteriorly from the parasphenoid body (Figs. 42A, 42D, 42F–42H). An angle of ∼92° is measured between the two processes. This angle is comparable to early diverging ornithopods and more acute than in many styracosternans (*e.g.*, Norman, 1980; Taquet, 1976; Xing, Mallon & Currie, 2017) but not in all (Horner, 1992). The process tapers distally and an elliptical, anterolaterally facing flange is formed at the distal end (= condyle in *Ctenosaura pectinata*; Oelrich, 1956). The flange potentially formed the origin of the inferior segment of the *m. protractor pterygoideus*, as identified in *Ctenosaura pectinata* (Oelrich, 1956; see also Sobral, Hipsley & Müller, 2012). Distally, the basipterygoid process is accommodated in a socket formed by the basipterygoid boss of the pterygoid (Fig. 6A). The smooth lateral surface of the basipterygoid process could have supported the passage of the *vena capitus lateralis* (*sensu* Sobral, Hipsley & Müller, 2012) (= ‘internal jugular vein’ of some authors). The anteroventral surface of the parasphenoid body abutting the basisphenoid between the basipterygoid processes forms a smooth shallow fossa (Figs. 40A, 42A, 42F), as in *Hypsilophodon foxii* (Galton, 1974), *Tenontosaurus tilletti* (Thomas, 2015), *Thescelosaurus neglectus* (Boyd, 2014) and *Ouranosaurus nigeriensis* Taquet, 1976. In contrast, a deep fossa is formed in this location in *Dysalotosaurus lettowvorbecki* (Sobral, Hipsley & Müller, 2012) and comparatively deep fossae are also reported in *Dryosaurus elderae* (Carpenter & Lamanna, 2015) and *Edmontosaurus regalis* (Xing, Mallon & Currie, 2017). The ventral margin of the transverse flange abutting the basisphenoid is straight, as in *Camptosaurus dispar* (Carpenter & Lamanna, 2015), *Hypsilophodon foxii* (Galton, 1974), *Tenontosaurus tilletti* (Thomas, 2015) and *Thescelosaurus neglectus* (Boyd, 2014). In contrast, a narrow median process projects posteriorly over the basisphenoid in styracosternans, such as *Bactrosaurus johnsoni*, *Batyrosaurus rozhdestvenski*, *Eotrachodon orientalis*, *Iguanodon bernissartensis* and *Ouranosaurus nigeriensis* (Godefroit et al., 1998; Godefroit et al., 2012; Norman, 1980; Prieto-Márquez, Erickson & Ebersole, 2016; Taquet, 1976). However, the margin is straight in *Edmontosaurus regalis* (Norman, 1986) and *Mantellisaurus atherfieldensis* (Xing, Mallon & Currie, 2017), indicating variability in this feature among derived ornithopods.

The parasphenoid contributes to the bilaminate preotic pendant with the prootic, which forms the slot-like, posterolaterally oriented cerebral carotid canal (Figs. 33B, 38O, 41C, 41H–41J, 41L 42A, 42C, 42D; see also “Prootic” above). The parasphenoid portion of the preotic pendant consists of a thin, tab-like ala contributing to the anterior wall within the canal, while the abutting ala of the prootic forms the anterior face of the pendant. The cerebral carotid artery entered the *sella turcica* through a foramen at the medial end of the slotted canal (Figs. 38O, 40C, 41E). The deeply slotted canal for the cerebral carotid artery is apparent in the early diverging ankylopollexian, *Camptosaurus dispar* (Carpenter & Lamanna, 2015; Gilmore, 1909) and the styracosternans, *Bactrosaurus johnsoni* (Godefroit et al., 1998), *Ouranosaurus nigeriensis* Taquet, 1976 and *Prosaurolophus blackfeetensis* (Horner, 1992). In contrast, the cerebral carotid artery enters the braincase wall more directly in *Hypsilophodon foxii*, the droysaurids, the early diverging styracosternan, *Mantellisaurus atherfieldensis* and the hadrosaur *Edmontosaurus regalis* (Carpenter & Lamanna, 2015; Galton, 1974; Norman, 1986; Xing, Mallon & Currie, 2017). The cerebral carotid artery enters the braincase in a groove medial to the ala process in the Romanian hadrosauroids *Transylvanosaurus platycephalus* and *Telmatosaurus transylvanicus* (Augustin et al., 2022; Augustin et al., 2023) and a caudolaterally extending preotic pendant is not apparent. The Romanian hadrosauroids show that the dorsoventrally extending ala process on the basisphenoid is equivalent to the unnamed crista (“?”) reported by Thomas (2015) on the braincase of *Tenontosaurus tilletti*. Thus, the ala process on the basisphenoid also forms the posterior ridge of the basipterygoid process (see (Augustin et al., 2022, fig. 3). Differing from *Muttaburrasaurus langdoni*, the cerebral carotid artery in *Tenontosaurus tilletti* enters the braincase medial to the pterygoid process (Thomas, 2015).

#### Orbitosphenoid

The orbitosphenoid is a midline bone of the ossified chondrocranium forming the anterodorsal region of the sphenethmoid region and the anterior wall of the telencephalon, including the infundibulum. The body of the orbitosphenoid is roughly V-shaped in anteroposterior view, triangular in lateral view and trapezoidal in dorsoventral view (Figs. 42G–42K). Internally, the body of the orbitosphenoid forms an anteriorly tapering channel housing the anterior end of the cerebellum leading to the single opening for the paired olfactory tracts (cnI) (Figs. 42I, 42J, 42H). The descending process projects from the body and encloses the midline foramen for the paired optic nerves (cnII; Figs. 33C, 34D, 42I, 42J, 42H). Paired ventral tubercles formed at the base of the descending process contact the dorsal surface of the parasphenoid body. The foramen for the oculomotor nerve (cnIII) passes through the sutural margin between the orbitosphenoid and the laterosphenoid, ventrolateral to the optic nerves (Figs. 33C, 34D). The foramen for the trochlea nerve (cnIV) passes through the sutural margin between the orbitosphenoid and laterosphenoid, dorsolateral to the optic nerves (Figs. 33C, 34D). After entering the foramen, the trochlea nerve rami divide, with the second ramus passing through the orbitosphenoid and exiting anterolateral to the first ramus on the sutural margin (Fig. 33C). Compared to *Muttaburrasaurus langdoni*, contact between the orbitosphenoid and parasphenoid in Styracosterna is typically elongate and extending onto the cultriform process (see “Parasphenoid” above). However, similarly to *Muttaburrasaurus langdoni*, contact between these bones in *Ouranosaurus nigeriensis* Taquet, 1976, is relatively reduced. An ossified orbitosphenoid is typically absent in early diverging ornithischians, including *Hypsilophodon foxii* (Galton, 1974). An ossified orbitosphenoid occurs in *Tenontosaurus* (Thomas, 2015; Winkler, Murry & Jacobs, 1997) and Dryomorpha (Carpenter & Lamanna, 2015; Norman, 1986; Ostrom, 1961; Taquet, 1976; Xing, Mallon & Currie, 2017) and has not been identified in Rhabdodontomorpha, or any taxon assigned to Elasmaria (Herne et al., 2019), previous to the assignment of *Muttaburrasaurus* to that clade by Fonseca et al. (2024b). Differing from *Muttaburrasaurus langdoni*, the orbitosphenoid in *Tenontosaurus tilletti* fails to contact the parasphenoid (Thomas, 2015), which might also be the condition in *Tenontosaurus dossi* Winkler, Murry & Jacobs, 1997 (Fig. 9).

#### Skull openings

As in lambeosaurines (*sensu* Prieto-Márquez & Wagner, 2013), the pseudonaris is identified by a lateral opening anteriorly between the posterodorsal and posteroventral processes of the left premaxilla (Figs. 8, 16, 18). Dorsoventrally elongate slots, previously suggesting narial openings on the muzzle (Bartholomai & Molnar, 1981; Molnar, 1995; Molnar, 1996), conflict with the lateral opening identified here on the left side, as the posterior margin of the pseudonaris. The dorsal slots are reinterpreted as dorsally eroded bony sheets of the prenasal ossifications. The antorbital fossa is small, <5% of anteroposterior cranial length measured from the posterior margin of the ventral quadrate condyles to the presumed anterior point on the premaxilla. The complete shape of the external antorbital fenestra is uncertain but the dorsal margin preserved on the right lacrimal, suggests an elliptical shape with a sloping anterior margin (Figs. 20B, 20D; Table 1), as in *Gasparinisaura cincosaltensis* (Coria & Salgado, 1996). On the left side, the anterior end of the antorbital fossa appears to merge with the lateral surface of the maxilla (Fig. 20B) as in the protoceratopsian, *Protoceratops hellenikorhinus* (Lambert et al., 2001). However, based on the incomplete right side, the anterior margin could have been sharper edged (Fig. 20D). The internal antorbital fenestra opening between the antorbital fossa and the nasal cavity, forms a dorsoventrally low, anteroventrally elongate slot between the antorbital fossa and neurovascular tract of the maxilla (Figs. 20B, 20C). The small, pocket-like morphology of the antorbital fossa resembles the condition in *Iguanodon bernissartensis* (Norman, 1986) and *Gasparinisaura cincosaltensis* Coria & Salgado, 1996, and is unlike the condition in *Galleonosaurus dorisae* (Herne et al., 2019), *Leaellynasaura amicagraphica* (Herne, 2014) and *Hypsilophodon foxii* (Galton, 1974) and the early diverging neornithischians, *Haya griva* (Barta & Norell, 2021) and *Jeholosaurus shangyuanensis* (Barrett & Han, 2009), where the fossae are more extensive and the anterior margins of the fossae are clearly sharp edged. A slot-like opening occurs between the anteroventral process of the nasal and the lacrimojugal process of the maxilla (Fig. 20). Medial to this opening, a gap occurs between the posteroventral process of the premaxilla and the anteroventral process of the lacrimal. These openings form a common fossa into which the anterior opening of the nasolacrimal duct opens, and in addition, the ventral opening of the vertical duct on the medial wall of the nasal. This fossa is potentially equivalent to the pre-antorbital sinus reported in the prosauropod *Plateosaurus engelhardti* (Witmer, 1997a). The orbit forms a roughly square-shaped opening (Fig. 5A) and is anteromedially and ventrally bordered by the palatine. A cartilaginous sheet potentially formed the posterior wall of the orbital fossa, as indicated by the appearance of mineralised material in the scans, which potentially suggests the remnants of osteoid (Figs. 40D, 40E). The mandibular adductor chamber (= temporal fossa) opens to the supratemporal, infratemporal and subtemporal fenestrae and is walled medially by the neurocranium and laterally by the jugal and quadratojugal (Figs. 5A, 6B; Table 1). The temporal fossa would have provided the origin of the principal mandibular adductor muscles (*m. adductor mandibulae externus profundus*, *m. adductor mandibulae externus superficialis*, *m. adductor mandibulae externus medialis*, *m adductor mandibulae posterior*, *m. pseudotemporalis superficialis*; Holliday, 2009). The large size of the temporal fenestra suggests that *Muttaburrasaurus langdoni* had sizable mandibular adductor musculature and a large bite force (see also Molnar, 1995; Molnar, 1996). The interfenestral bar, formed by the postorbital and squamosal, separates the supratemporal and infratemporal fenestrae. However, only a small portion of the interfenestral bar is preserved on the left postorbital (Fig. 6B). The preserved portion, although provisionally identified, suggests that the left supratemporal fenestra is sub-triangular in shape. The infratemporal fenestra is triangular, narrowing anteroventrally (Fig. 5A). The anterior, ventral and posteroventral margins of the infratemporal fenestra are formed by the jugal. The subjugal opening, through which the mandibular adductor musculature passed (Holliday, 2009), is an elongate slot (Fig. 6A). Ventrally, the slot extends anteriorly as a shallow channel on the ventral surface of the jugal shelf of the maxilla (Figs. 6A, 21A, 21B, 21F). The posterodorsal margin of the infratemporal fenestra is formed by the quadratojugal (Fig. 5A). A small, U-shaped, vascular canal extending through the paroccipital process is possibly the remnant of the posttemporal fenestra (see also under “Otoccipital” above; Figs. 33D, 34C, 37A–37C, 37F). A pocket-like, anterior maxillary fossa occurs between the ascending process of the maxilla and the posteroventral process of the premaxilla (Fig. 5A), as in *Changchunsaurus parvus* (Jin et al., 2010) and *Thescelosaurus neglectus* (Boyd, 2014). Within the fossa, foramina that would have connected the maxillary branch of the trigeminal nerve (cn V) in the maxillary and premaxillary branches of the neurovascular tracts, exit laterally (Figs. 13A–13D, 24).

#### Mandible overview

**Figure 43:**
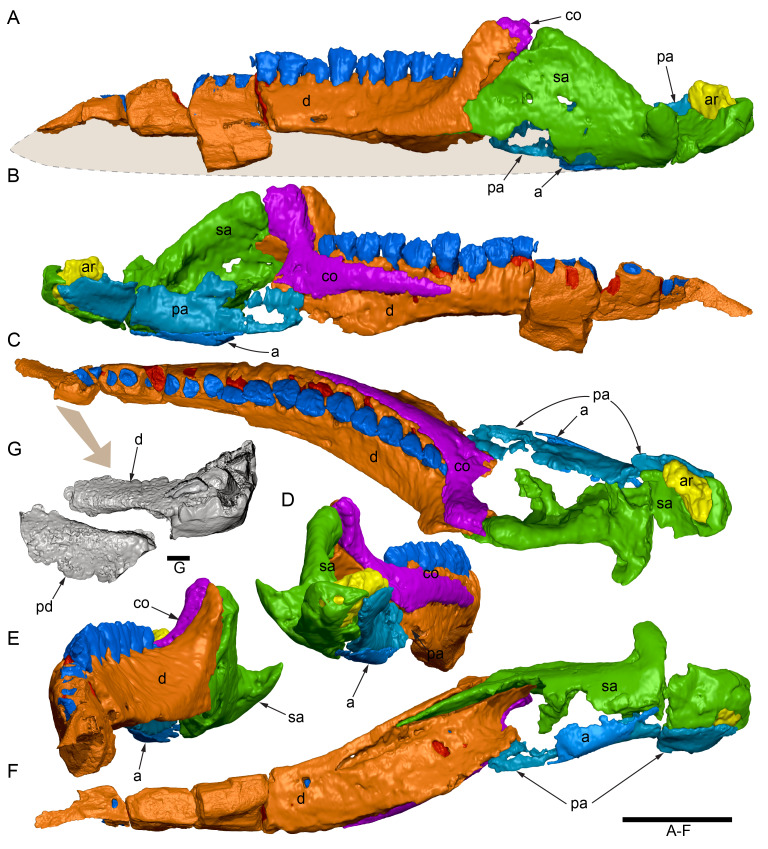
Volume rendered model of *Muttaburrasaurus langdoni* (QMF6140) left mandibular corpus. (A–F) Mandibular corpus in (A) lateral, (B) medial, (C) dorsal, (D) posterior, (E) anterior and (F) ventral views. (G) Anterior end of mandibular corpus (cranial part 3) in dorsal view, showing possible fragment of the predentary preserved in the host matrix, displaced from the predentary facet of the dentary. Functional teeth shown blue and germ teeth shown red. Abbreviations: a, angular; ar, articular; co, coronoid; d, dentary; pa, prearticular; pd, predentary; sa, surangular. Shaded area in A, suggested area eroded ventrally (noting that the ventral profile is unknown). Scale bar A–F equals 10 cm and G equals one cm.

Mandibular corpora are preserved on both sides, with the left more complete than the right and forming the bulk of the description that follows (Figs. 3, 5–7, 43). The partial predentary, dentary, coronoid, prearticular, surangular, angular and articular are preserved on the left side (Fig. 43). The partial dentary and coronoid are preserved on the right side. The splenials are missing. The ventral portion of the dentary is missing on the left side, including the medially projecting symphyseal process and much of the predentary process. Viewed dorsoventrally, the mandibular corpus is sinuous, with a distinct kink at the dentary surangular joint (Figs. 43C, 43F). The mandible is transversely broad at the quadratomandibular joint, through to the posterior end of the dentary and narrower anteriorly (Figs. 43C, 43F).

#### Predentary

No part of the predentary was previously reported. From the CT imagery, a platy fragment in the block of cranial part 3 is provisionally identified as a part of the left lateral wing/process of the predentary (Fig. 43G). The fragment appears to have been displaced from the predentary facet of the dentary and no further material of the predentary is identified. The ornithischian predentary is formed from lateral processes that converge anteriorly to form either a V-shaped, sharply pointed, mandibular part of the rostrum, as in *Thescelosaurus neglectus* (Boyd, 2014), or a rounded to blunt ended mandibular part of the bill, as in iguanodontians (Norman, 2004). Whether the *Muttaburrasaurus langdoni* predentary was anteriorly V-shaped, as in early diverging ornithischians, or rounded/U-shaped to blunt-ended and denticulate, as in more derived ornithopods, such as, *Tenontosaurus tilletti* (Thomas, 2015), rhabdodontomorphs (Weishampel et al., 2003) and ankylopollexia (Norman, 1986; Norman, 1998), is unknown. Nevertheless, the dentulous, transversely narrow premaxilla, suggests that the *Muttaburrasaurus langdoni* predentary was anteriorly narrow, as in early diverging ornithopods and other ornithischians, as opposed to transversely broad and blunt ended (see Nabavizadeh & Weishampel, 2016).

#### Dentary

**Figure 44:**
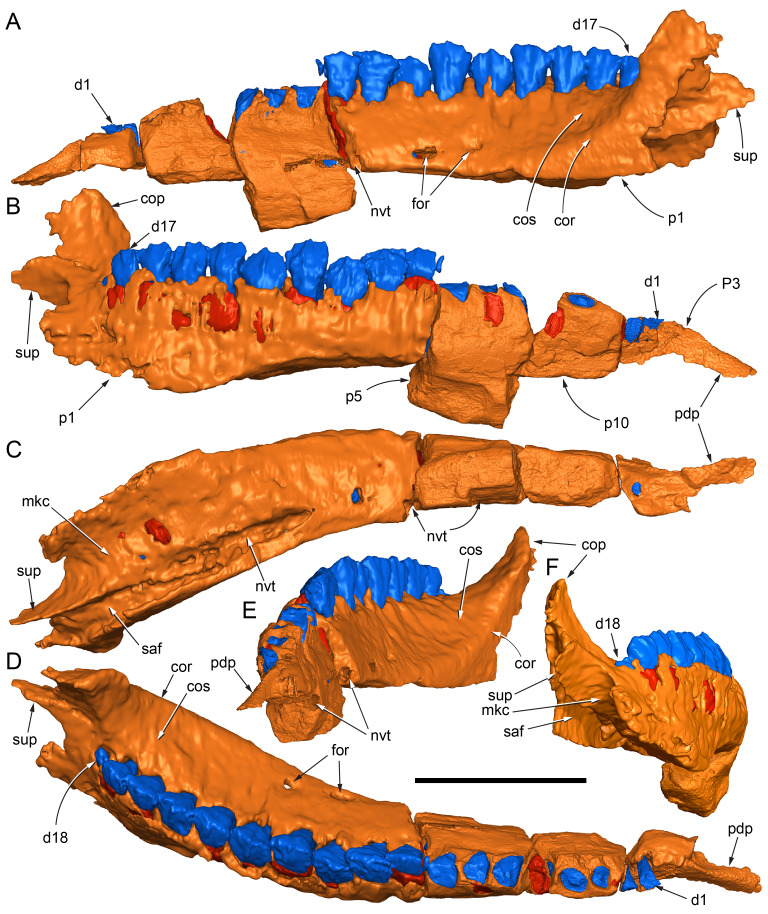
Volume rendered model of the *Muttaburrasaurus langdoni* (QMF6140) left dentary. (A–F) Left dentary in (A) lateral, (B) medial, (C) ventral, (D) dorsal, (E) anterior and (F) posterior views. Abbreviations: cop, coronoid process; cor, coronoid ridge; cos, coronoid shelf; d#, dentary tooth position/family and development number (.1 = functional tooth [blue]; .2 = germ tooth [red]); for, foramen; mkc, Meckelian canal; nvt, neurovascular tract; p#, cranial part and number; pdp, predentary process; saf, surangular fossa; sup, surangular process. Scale bar equals 10 cm. MorphoSource DOI: 10.17602/M2/M786907; 10.17602/M2/M786883; 10.17602/M2/M786887; 10.17602/M2/M787667; 10.17602/M2/M787664; 10.17602/M2/M787670.

**Figure 45:**
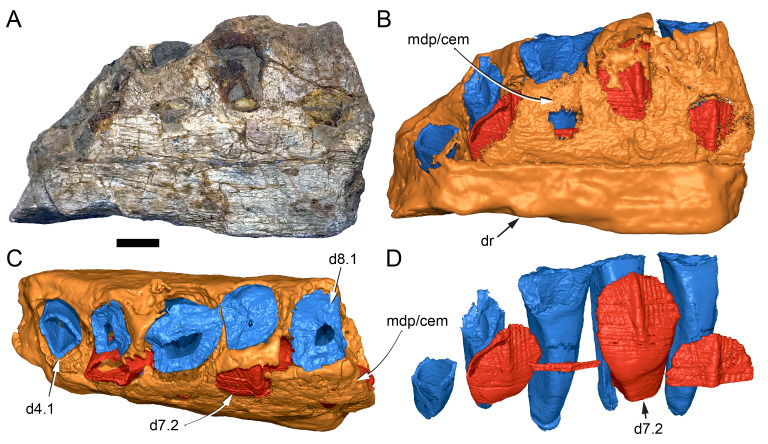
Photograph and volume rendered model of the *Muttaburrasaurus langdoni* (QMF6140) right anterior dentary fragment. (A) Photograph of right dentary fragment (cranial part 11) in medial view. (B, C) Volume rendered model in (B) medial and (C) dorsal views. (E) Dentition in lingual view. Abbreviations: cem, calcified cementum; dr, dental ramus of the dentary; d#, dentary tooth position/family and development number (.1 = functional tooth [blue]; .2 = germ tooth [red]); mdp, medial dental parapet. Scale bar equals one cm. MorphoSource DOI: 10.17602/M2/M786891; 10.17602/M2/M786899; 10.17602/M2/M786903; 10.17602/M2/M786895.

Both dentaries are present, although eroded ventrally (Figs, 44–48). The posterior halves of the dentaries are preserved on the large cranial block (cranial part 1; Fig. 3). In addition, the anterior half of the left dentary is preserved in three connecting parts (cranial parts 3, 5 and 10), while a mid-dental ramus fragment (cranial part 11) and anterior-most fragment (cranial part 3) are preserved on the right side (Figs. 3, 44, 45). The description that follows is mainly based on the better preserved and digitally restored left dentary (Fig. 44). The dental ramus hosts 18 alveoli, determined from the complete left dental arcade. The lower number of tooth families in the dentary than in the maxilla (21 alveoli) is offset by the mesiodistally broader dentary tooth crowns (see “Dentary teeth” below). Ten alveoli are preserved on the right side of the large cranial block (cranial part 1), although not figured. These are assessed as dentary tooth families (d)9–d18. The right dentary fragment (cranial part 11) preserves five alveoli, which are potentially d4–d8 (Fig. 45). The ventral portion of the left dentary fragment, cranial part 10, is missing. Viewed laterally on the left side, d17 is partly obscured by the coronoid process and d18 is located medial to the coronoid process (Fig. 44). Cranial part 10 on the left side indicates that full dorsoventral dentary depth was at least 100 mm in the middle of the tooth row, taken from the ventral margin of the dental ramus to the tooth crown apex (Fig. 44). Viewed dorsoventrally (Figs. 44C, 44D), the dentary is strongly bowed (laterally concave/medially convex) and the medial and lateral margins converge anteriorly. The bowed form of the dentary is comparable to elasmarians (Herne et al., 2019; Rozadilla, Agnolín & Novas, 2019), *Tenontosaurus tilletti* (Thomas, 2015) and non-hadrosaurid dryomorphs (*e.g.*, *Camptosaurus dispar* (YPM 1886, based on YPM supplied images); *Dysalotosaurus lettowvorbecki* (Janensch, 1955); *Ouranosaurus nigeriensis* Taquet, 1976; *Mantellisaurus atherfieldensis* (Norman, 1986)) and differs from the relatively straight condition in the rhabdodontomorphs (Chanthasit, 2010; Osi et al., 2012; Weishampel et al., 2003), which has been considered synapomorphic for the latter clade (Zanno et al., 2023). The transversely broadest width on the dentary of ∼55 mm corresponds to dentary tooth position (d)16. Viewed dorsally, the curved alveolar/tooth arcade axis is only marginally longer (∼5 mm) than the maxillary axis (see Table 2). Viewed mediolaterally, the profiles of the dorsal alveolar and apical dental margins are shallowly convex (Figs. 44A, 44B, 44E, 44F). However, the profile of the ventral margin is unknown. The coronoid shelf is transversely broad and slopes ∼45° from the coronoid ridge to the alveolar margin (Figs. 44A, 44D, 44E). The coronoid process is low, posterodorsally sloping and tapered dorsally (Figs. 44A, 44B). The shape of the coronoid process is comparable to that of *Ouranosaurus nigeriensis* Taquet, 1976 and differs from the relatively vertical and anteriorly expanded process more typically occurring in styracosternans, such as *Altirhinus kurzanovi*, *Brachylophosaurus canadensis*, *Edmontosaurus regalis* and *Lambeosaurus magnocristatus* (Lambe, 1920; Norman, 1986; Norman, 1998). Positioned medial to the surangular, the surangular process (= posterior coronoid cusp) is triangular in mediolateral view and obliquely angled in posterior view (Figs. 44A–44D, 44F). Viewed ventrally, a slot-like fossa is formed posteroventrally for the surangular internal to the lateral wall of the dental ramus (Figs. 44C, 44F). The transversely broad Meckelian canal is medial to the surangular fossa. A sharp ridge divides the Meckelian canal from the surangular fossa (“sup” in Figs. 44A, 44C, 44F). The neurovascular tract, which carried the mandibular branch of the trigeminal nerve (cn V), courses anteriorly at the level of the root apices from its posterior opening in the Meckelian canal (Fig. 44C). Small ducts branch from the neurovascular tract towards the root apices. Neurovascular foramina exit laterally on the dental ramus dorsal to the coronoid ridge (Figs. 44A, 44D). The likely exit of the neurovascular tract at the anterior end of dentary (‘anterior dentary foramen’) is not preserved. Distinction between the bone of the dental ramus and bone and ossified cementum of the medial dental parapet is apparent from the higher resolution micro-CT scan of cranial part 11 (Fig. 45). However, “Special foramina” that were proposed by Edmund (1957) to supply nutrients to the developing dental lamina in some ornithischians, are not apparent along the margin of the dental parapet and bone of the dentary. The left predentary process is incomplete and only represented by a dorsal portion (Figs. 44A–44E). The anterior and ventral extent of the predentary and symphysial processes is unknown. Viewed dorsoventrally, the predentary process recurves medially relative to the laterally concave anteroposterior axis of the dental ramus (Figs. 44C, 44D). The dorsal margin of the predentary process forms a rounded ridge, which is indented laterally at its posterior end. The dorsal margin of the predentary process slopes at a shallow angle anteroventrally. A small diastema of ∼9 mm occurs between the predentary process and the anterior-most tooth position. A reduced diastema is typical of early diverging neornithischians.

#### Dentary teeth

**Figure 46:**
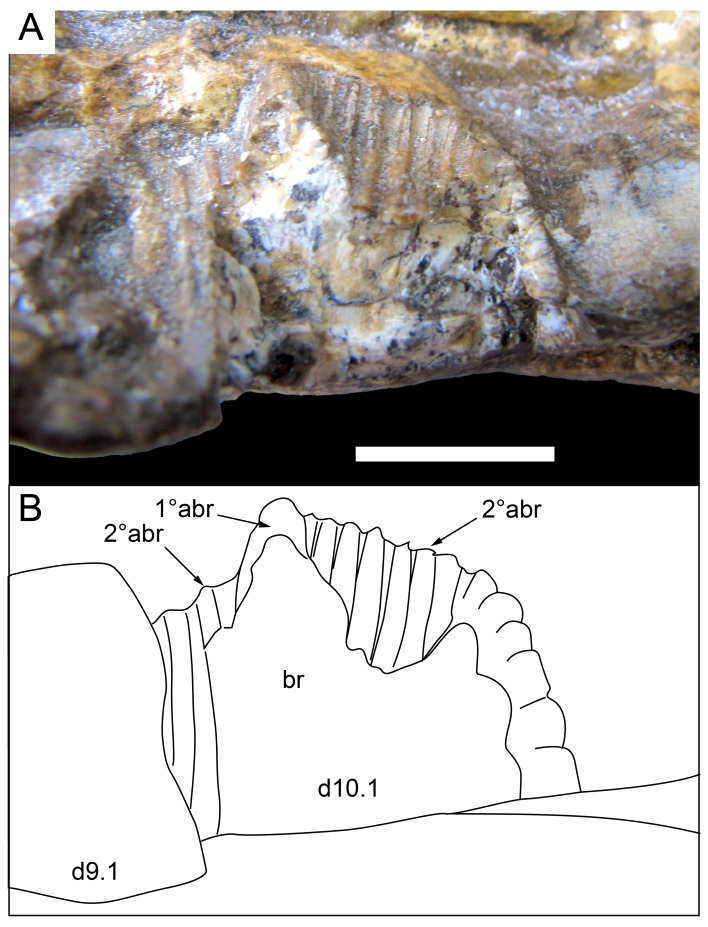
Photograph of *Muttaburrasaurus langdoni* (QMF6140) functional mid-dentary tooth crown. (A, B) Right functional tooth crown d10. 1 (A) in lingual view and (B) explanatory schematic. Abbreviations: 1° abr, primary (apicobasal) ridge; 2° abr secondary apicobasal ridge; br, breakage; d#, dentary tooth position/family and development number (.1 = functional tooth). Scale bar equals one cm.

**Figure 47:**
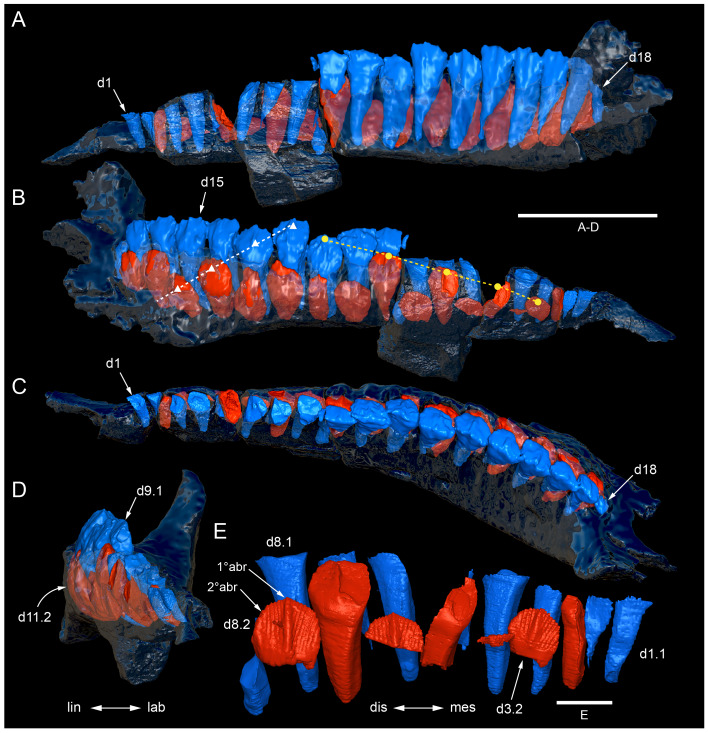
Volume rendered model of the *Muttaburrasaurus langdoni* (QMF6140) left dentary dentition. (A–D) Complete dentition in (A) labial, (B) lingual, (C) apical and (D) mesial views. (E) Anterior dental region (d1–d9) in lingual view. Yellow dots and dashed line in B indicate a posterior to anterior replacement wave across alternate tooth families. White dashed line and triangles in B suggest a Zahnreihe. Abbreviations: 1° abr, primary (apicobasal) ridge; 2° abr secondary apicobasal ridge; d#, dentary tooth position/family and development number (.1 = functional tooth [blue]; .2 = germ tooth [red]); dis, distal; lab, labial; lin, lingual; mes, mesial. Scale bar A–D equals 10 cm and E equals two cm. MorphoSource DOI: 10.17602/M2/M786883; 10.17602/M2/M786887; 10.17602/M2/M787667; 10.17602/M2/M787670.

**Figure 48:**
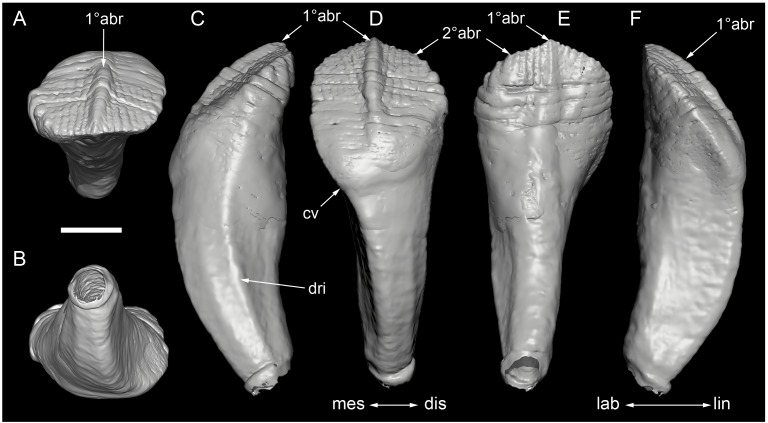
Volume rendered model of the *Muttaburrasaurus langdoni* (QMF6140) right, mid-dentary germ tooth. (A–F) Dentary germ tooth reconstructed from root d6. 1 and crown d7. 2 (cranial part 11) in (A) dorsal, (B) ventral, (C) distal, (D) lingual, (E) labial and (F) mesial views. Abbreviations: 1° abr; 2° abr; cv, cingular vertex; dri, distal ridge; dis, distal; mes, mesial. Scale bar equals one cm. MorphoSource DOI: 10.17602/M2/M786899.

On the originally described cranial blocks, the apical regions of the tooth crowns are only directly visible lingually on the right side of the muzzle block (cranial part 2) (Figs. 3D, 46). The best lingual crown surface directly observable on cranial part 2 is the apical half of right d10 (Fig. 46). On cranial parts 1 and 2, the roots and crowns on the left side were volume rendered from the medical CT data (Fig. 47); however, resolution of the root and crown morphology was poor, particularly with respect to crown ornamentation. On the smaller left dentary fragments (cranial parts 3, 5 and 10), which preserve tooth families d1–d8, better tooth resolution was available from the higher resolution CT scans (Fig. 47E; see “Methods”). However, functional crowns are not preserved on these dentary fragments, although germ crowns and the roots of the broken functional teeth are preserved. On the right mid-dentary fragment (cranial part 11), the germ crown d7.2 and functional root at d6.1 provided adequate detail for the full tooth morphology. Assessment of the complete tooth morphology was enabled by a digital reconstruction utilising right d7.2 and d6.1 (Figs. 45, 48).

One functional tooth and one germ tooth occur at each tooth position (Figs. 45D, 47). In mesiodistal view, the tooth is robust and the angle between the lingual surface of the crown and the root axis is ∼120° (Figs. 48C, 48F). The root curves labially and slightly mesially towards its apical (ventral) end (Figs. 48C–48F). As a result, the lingual face of the dentition is strongly convex (Fig. 47D). The replacement tooth is distal to the root of the functional tooth at the same tooth position, as in the premaxillary and maxillary dentition. Thus, resorption of the functional tooth root is along the linguodistal margin, consistent with resorption in other reptiles (Edmund, 1960). The dental replacement pattern is not as clearly defined as the maxillary dentition. However, from d4 to d11, a posterior to anterior replacement wave across alternating tooth families is identified (Fig. 47B), as in the maxillary dentition. The replacement wave appears to consist of nine tooth families. Zahnreihe are not clearly defined, but at d15-d16, a Z value of 1.8 is suggested (using Osborn, 1977; see further information in Fig. 27G). The rate of dentary tooth replacement could have been less than the maxillary teeth. The mesial surface of the root is labiolingually broad and depressed, while a strong ridge divides the distal side into labial and lingual faces (Figs. 48C, 48F). Near its base, the root bulges labially (Figs. 48C, 48F). Apical to the root base, the root cross-section is roughly D-shaped. An apical neurovascular foramen is present, and the neurovascular root canal extends into the developing and functional crowns.

The crowns are spatulate in labiolingual profile (see further in Herne et al., 2019) and imbricated, with the mesial edge lingually overlapping the distal margin of the mesially adjacent crown (Figs. 47B, 47C). The crowns are offset mesially relative to the root (Figs. 48D, 48E). Lingually, a distinct cingulum occurs at the crown base. The cingular vertex at the crown base is basally (ventrally) depressed and offset mesially relative to the root (Fig. 48D). Thus, the crown is asymmetrical. Mesial offset and basal depression of the cingular vertex is similarly reported in Victorian ornithopod dentary morphotype 3 and an isolated right dentary tooth of an indeterminate small-bodied ornithopod from the Winton Formation, QMF52774 (Herne et al., 2019). Similar asymmetry of the lingual cingular vertex is apparent on the dentary crowns of the small-bodied ornithopod from the Griman Creek Formation at Lightning Ridge, *Weewarrasaurus pobeni* (Bell et al., 2018). Unlike *Muttaburrasaurus langdoni*, the base of the cingulum in *Qantassaurus intrepidus* is nearly central and V-shaped (Herne et al., 2019), with similar symmetrical morphology apparent in *Talenkauen santacrucensis* (based on Rozadilla, Agnolín & Novas, 2019, figs. 9 and 11) and *Kangnasaurus coetzeei* (following Herne et al., 2019), as well as many other ornithopods. Labially, the crown surface merges smoothly with the root without a distinct cingular base.

The dentary crowns are mesiodistally wider than the maxillary crowns (Table 1), with the widest crown at d13 (27.5 mm). The lingual face of the crown is shallowly convex, both apicobasally and mesiodistally, and the labial face is mesiodistally convex and apicobasally straight (Figs. 48C, 48F). Enamel is developed lingually and labially on the crowns; however, differentiation between enamel and dentine is not completely certain from the CT radiographs of the germ crowns in cranial part 11. Nevertheless, from the available data, enamel thickness in the range of 200 µm is suggested. Mesial and distal bounding ridges on the cingulum are undefined, which differs from the thumb-like ridges on the crowns of Victorian ornithopod dentary morphotype 3 and *Qantassaurus* spp., as well as *Kangnasaurus coetzeei* (Herne et al., 2019). Similar bounding ridges occur in the elasmarian *Talenkauen santacrucensis* (Egerton et al., 2013, fig. 3B; Rozadilla, Agnolín & Novas, 2019, fig. 11) and bounding ridges on the dentary crowns of the rhabdodontids, *Zalmoxes robustus* Weishampel et al., 2003 and *Mochlodon vorosi* (Virag & Osi, 2017) are sharp edged. Similarly, the mesial and distal bounding ridges on the dentary crowns of Dryomorpha are typically sharp edged and border smooth paracingular fossae.

Lingually, the secondary apicobasal ridges merge with the cingulum without an ectoloph (= basal step or elevated rim of some authors) (Fig. 48D), differing from the ectoloph in the rhabdodontomorphs, *Iani smithi* Zanno et al., 2023, *Mochlodon vorosi* (Osi et al., 2012; Virag & Osi, 2017), *Rhabdodon* sp. (Chanthasit, 2010, fig. 4.9F), *Zalmoxes robustus* Weishampel et al., 2003 and *Z. shqiperorum* (Godefroit, Codrea & Weishampel, 2009). A shallow primary ridge is present lingually and secondary ridges terminating in apical denticles are developed mesial and distal to the primary ridge (Fig. 48D). The mesiodistal margins of lingual primary ridge are parallel, as opposed to the condition in rhabdodontomorphs where the margins of the primary ridge distinctly flare towards the base at the ectoloph (Chanthasit, 2010, fig. 4.9; Godefroit, Codrea & Weishampel, 2009, fig. 11; Osi et al., 2012, fig. 4; Zanno et al., 2023, fig. 12). The lingual secondary ridges are fine and closely abutting. Lingually, the mid-dentary crowns have nine secondary ridges mesial to the primary ridge and eight or nine secondary ridges distal to the primary ridge. The mesial secondary ridges are convergent towards the primary ridge, whereas distally, the secondary ridges run parallel to the primary ridge (Figs. 47E, 48D, 48E), as in *Talenkauen santacrucensis* (based on Rozadilla, Agnolín & Novas, 2019, fig. 11). The secondary ridges lack the channels between the ridges present on the crowns of *Hypsilophodon foxii* (BMNH R2477; M. Herne, pers. obs., 2009) and iguanodontians, such as, *Altirhinus kurzanovi* (Norman, 1998), *Camptosaurus dispar* (Galton, 2006, fig. 2.18), *Cumnoria prestwichii* (Maidment et al., 2023, fig. 10), *Dryosaurus altus* (Galton, 1983, fig. 4), *Iguanodon bernissartensis* (Norman, 1980), *Gryposaurus latidens* (Prieto-Marquez, 2012), *Tenontosaurus tilletti* (Thomas, 2015) and the rhabdodontids *Zalmoxes robustus* (BMNH R3392; M. Herne, pers. obs., 2009) and *Rhabdodon* sp. (Chanthasit, 2010, fig. 4.9F). Unsupported marginal denticles are absent on the mesial and distal apical margins of the *Muttaburrasaurus langdoni* crowns; thus, differing from the crowns in many ornithopods, such as *Camptosaurus dispar* (YPM 1886; Herne et al., 2019), *Cumnoria prestwichii* (Maidment et al., 2023, fig. 10), *Dryosaurus altus*, (Galton, 1983, fig. 4; *Gryposaurus latidens* (Prieto-Marquez, 2012; *Hypsilophodon foxii* (BMNH R2477; Galton, 2009; M. Herne, pers. obs., 2009), *Mantellisaurus atherfieldensis* (Norman, 1986), *Tenontosaurus tilletti* (Thomas, 2015) and *Zalmoxes robustus* (Weishampel et al., 2003) (BMNH 3395, M. Herne, pers. obs., 2009), where unsupported denticles are developed.

A shallow primary ridge is developed labially on the crown (Fig. 48E). The primary ridge expands mesially and distally towards the crown base merging with the root. Closely abutting secondary ridges with rounded labial edges are developed on the apical part of the labial surface (Fig. 48E), as in *Talenkauen santacrucensis* ((Rozadilla, Agnolín & Novas, 2019, fig. 11) and the rhabdodontomorph, *Iani smithi* Zanno et al., 2023, but not in *Zalmoxes robustus* Weishampel et al., 2003, where the labial surface of the dentary crown is smooth (BMNH 3395, M. Herne, pers. obs., 2009), as in dryomorphans. Labial secondary ridges also occur on the crowns of *Qantassaurus intrepidus* but are sharp-edged and separated by channels (Herne et al., 2019). The occlusal surfaces of the worn functional crowns are angled at 53° and 74° at positions d9 and d14, respectively, relative to their lingual surfaces (Figs. 25C, 25D). The angle of the occlusal surface from the horizontal plane is difficult to assess with certainty, but in the range of 40°. A slight concavity is detected on some of the worn occlusal surfaces. Apart from the number of secondary ridges, dentary tooth crown shape and ornamentation more closely resembles that of the Australian small-bodied ornithopods, *Qantassaurus intrepidus*, Victorian ornithopod dentary morphotypes (VOD) 2 and VOD 3 (Herne et al., 2019), *Weewarrasaurus pobeni* (Bell et al., 2018) and QMF52774 (Herne et al., 2019) and the Argentinian elasmarians, *Anabisetia saldiviai* Coria & Calvo, 2002 (MCF PVPH-74; M. Herne, pers. obs., 2008), *Gasparinisaura cincosaltensis* (MUCPv-213: Salgado, Coria & Heredia, 1997; M. Herne, pers. obs., 2008) and *Talenkauen santacrucensis* (Rozadilla, Agnolín & Novas, 2019, fig. 11) than all other ornithopods.

#### Surangular

**Figure 49:**
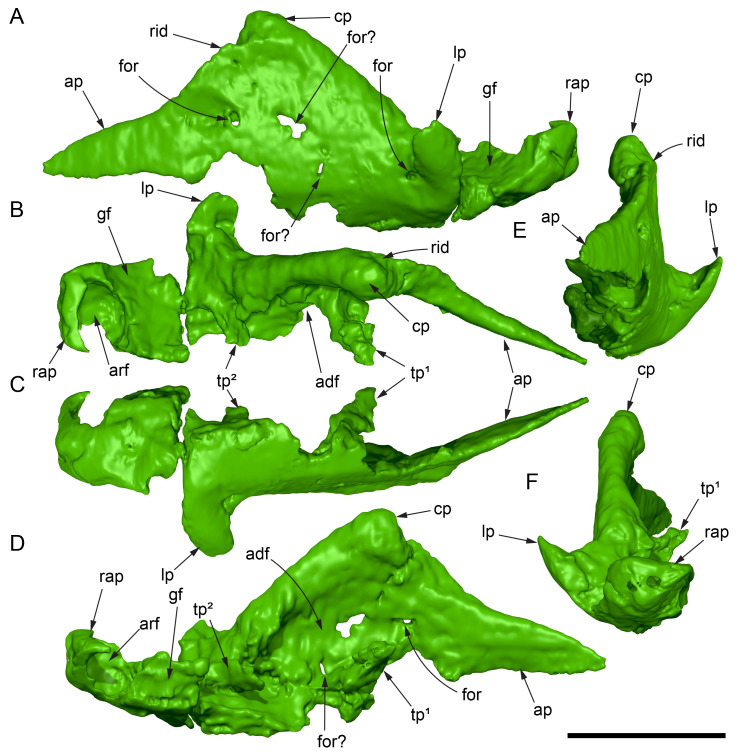
Volume rendered model of the *Muttaburrasaurus langdoni* (QMF6140) left surangular. (A–F) Left surangular in (A) lateral, (B) dorsal, (C) ventral, (D) medial, (E) anterior and (F) posterior views. Abbreviations: adf, adductor fossa; ap, anterior process (anterior angle); arf, articular fossa; cp, coronoid process (dorsal angle); for, foramen; gf, glenoid fossa of mandible; lp, lateral process; rap, retroarticular process; rid, ridge; tp 1 , anterior transverse process; tp 2 , posterior transverse process. Scale bar equals 10 cm. MorphoSource DOI: 10.17602/M2/M787673.

The left surangular is preserved. The surangular is elongate and contacts the dentary and coronoid anteriorly, the angular ventrally and the prearticular posteromedially (Fig. 49). A transverse groove posterior to the glenoid fossa accommodates the articular (Fig. 49). Viewed dorsoventrally, the surangular is laterally convex and medially concave (Fig. 49B, Fig. 49C). The body of the surangular is anteroposteriorly deep (Figs. 49A, 49D). The lateral sheet of the surangular body forms the shape of a right isosceles triangle (Figs. 43A, 43D). Ventrally, the lateral sheet curves medially inwards towards the angular (Fig. 43F). The dorsal peak of the surangular body (= dorsal angle; Thomas, 2015), forming the coronoid process of the surangular, has an included angle of 95°. Posterior to the coronoid process, the dorsal edge slopes 45°. The elongate form of the surangular body posterior to the coronoid process is comparable to that in *Camptosaurus dispar* (Brill & Carpenter, 2006; Gilmore, 1909), dryosaurids (Hübner & Rauhut, 2010; Janensch, 1961), *Gasparinisaura cincosaltensis* Coria & Salgado, 1996, and *Ouranosaurus nigeriensis* Taquet, 1976. By comparison, a steeper angle is apparent on the posterior edge in *Talenkauen santacrucensis* (Rozadilla, Agnolín & Novas, 2019), rhabdodontomorphs, *Tenontosaurus tilletti* (Thomas, 2015) and typically in styracosternans (*e.g.*, *Iguanodon bernissartensis* (Norman, 1980); *Edmontosaurus regalis* (Xing, Mallon & Currie, 2017); lambeosaurines (Ostrom, 1961)), apart from *Ouranosaurus nigeriensis* (Taquet, 1976). The elongate form of the surangular in *Muttaburrasaurus langdoni* and the other taxa mentioned, results in pushing the glenoid fossa further posteriorly relative to the dentary, when compared to taxa with steeper margins. A lobate coronoid process is formed at the dorsal peak, posteriorly abutting the coronoid process of the dentary and dorsal ramus of the coronoid (Figs. 43A–43C, 49A, 49B, 49D–49F). The lobate form of the dorsal peak/coronoid process is comparable to the “condyle” reported in *Dysalotosaurus lettowvorbecki*, which was previously considered unique to that taxon (Hübner & Rauhut, 2010). The coronoid process may be thinner in other dryomorphans but appears to be thickened in *Ouranosaurus nigeriensis* (Taquet, 1976) (Fig. 32). Viewed laterally, a distinct ridge extends anterioposteriorly below the coronoid process protuberance (Figs. 49A, 49B). The ridge is the lateral extension of an obliquely angled narrow shelf more anteriorly, within which the head of the coronoid articulates (Figs. 43A, 43D). The ridge and shelf likely form the base of insertion of the *m. adductor mandibulae externus profundus*, which might have also attached to a posterior part of the coronoid process of the dentary (based on Holliday, 2009). A similar ridge is apparent in *Ouranosaurus nigeriensis* (Taquet, 1976) (Fig. 32), *Thescelosaurus neglectus* (Boyd, 2014, fig. 2; Holliday, 2009) and *Zalmoxes robustus* Weishampel et al., 2003 (fig. 12J). The dorsal peak of the surangular is close to the level of the dorsal-most margins of the coronoid and coronoid process of the dentary (Figs. 43A, 43B, 43D, 43E). In *Hypsilophodon foxii* (Galton, 1974), *Tenontosaurus tilletti* (Thomas, 2015) and styracosternans (*e.g.*, Norman, 1980; Horner, 1992; Xing, Mallon & Currie, 2017), except for *Ouranosaurus nigeriensis* Taquet, 1976, the dorsal peak of the surangular is lower than the peak of the coronoid process on the dentary. Furthermore, the posterodorsal margins of the surangular body descending from the coronoid process in most styracosternans are more steeply sloping than in *Muttaburrasaurus langdoni*. As a result, the surangulars of many styracosternans are less anteroposteriorly elongate than in *Muttaburrasaurus langdoni*. As in *Muttaburrasaurus langdoni*, the surangular of *Ouranosaurus nigeriensis* appears comparatively elongate.

The elongate, anteriorly tapering, anterior process (= anterior angle; Thomas, 2015) is anteromedially deflected from the body of the surangular (Figs. 49B, 49C). The dorsal margin of the anterior process to the coronoid process is shallowly concave, like the margin partly preserved in the rhabdodontomorph, *Iani smithi* Zanno et al., 2023. The anterior process inserts in a groove on the dentary and the surangular process of the dentary overlaps the anteromedial surface of the surangular body (Figs. 43A, 43B, 43F). Hence, articulation with the dentary is tongue-and-groove. An elongate anterior process like that of *Muttaburrasaurus langdoni* occurs in *Thescelosaurus neglectus* (Boyd, 2014). The anterior process in *Tenontosaurus tilletti* bears some resemblance to that of *Muttaburrasaurus langdoni*, although lacking the greater degree of elongation. The anterior ends of the surangular in the elasmarian *Talenkauen santacrucensis* (Rozadilla, Agnolín & Novas, 2019) and the rhabdodontid, *Rhabdodon* sp. are lacking extended anterior processes (Chanthasit, 2010), although breakage cannot be discounted. An anterior process appears to be lacking in styracosternans (*e.g.*, *Batyrosaurus roshdestvenskyi* Godefroit et al., 2012; *Edmontosaurus regalis* (Lambe, 1920; Xing, Mallon & Currie, 2017); *Prosaurolophus blackfeetensis* (Horner, 1992); *Protohadros byrdi* (Head, 1998) and *Ouranosaurus nigeriensis* Taquet, 1976) and is uncertain in *Iguanodon bernissartensis* and *Mantellisaurus atherfieldensis*. However, the anterior end of the process in the latter taxon is reportedly rounded (*sensu* Hooley, 1925), as in the basal hadrosauriform, *Batyrosaurus roshdestvenskyi* Godefroit et al., 2012 (fig. 20.8). The anterior process of the surangular has yet to be described in the elasmarian, *Gasparinisaura cincosaltensis*.

The dorsal margin of the surangular body posterior to the dorsal peak forms a medially thickened lip, anteroventral to which the concave medial surface of the surangular body forms a deep adductor fossa (Fig. 49A, Fig. 49B). Two strut-like, transversely-oriented processes cross the adductor fossa (Figs. 49B, 49C, 49D, Fig. 49F). One strut is positioned approximately midway along the fossa and lacking contact at its distal end, and the other is at the posterior end of the fossa, anterior to the glenoid and connects with the prearticular. These processes have not been previously reported in an ornithischian. Owing to ventral erosion, the nature of connection with the angular is unclear. In the region of the glenoid (mandibular) fossa, the surangular is dorsoventrally compressed and broadens transversely where it forms most of the fossa surface, with medial contribution by the prearticular and posterior contribution from the articular (Fig. 43). Immediately anterior to the glenoid fossa, a prominent, thumb-like dorsolateral process is developed: offset laterally, projecting dorsally and pinching at its base (Fig. 49). The dorsolateral process closely resembles that of *Thescelosaurus neglectus* (Boyd, 2014). A dorsolateral process is also developed in *Tenontosaurus tilletti* (Thomas, 2015) and *Zalmoxes robustus* Weishampel et al., 2003, but is lower and lacks the narrow neck at the process base in *Muttaburrasaurus langdoni* and *Thescelosaurus neglectus*. In other ornithopods, such as *Hypsilophodon foxii* and most early diverging neornithischians, the lateral process of the surangular typically forms a low boss or ridge (Boyd, 2014; and authors within).

Four foramina are present on the surangular body (Fig. 49A). The anterior-most foramen occurs near the dentary margin, as in a broad selection of ornithopods (= external mandibular foramen, Gilmore, 1909; second foramen, Norman, 1998; large anterior foramen, cf Galton, 1974; accessory foramen, Kobayashi & Azuma, 2003; external mandibular fenestra, Weishampel et al., 2003) and opens into the adductor chamber. Laterally, the anterior foramen is positioned at the posterior end of a conspicuous groove, as in *Gasparinisaura cincosaltensis* (Coria & Salgado, 1996), and *Thescelosaurus neglectus* (Boyd, 2014, fig. 15). A second foramen is located immediately anterior to the base of the lateral process, as typically occurs in non-hadrosauriod neornithischians (*e.g.*, Boyd, 2014; Galton, 1974; Norman, 1986; Norman, 1998; Taquet, 1976; Thomas, 2015). This foramen does not appear to open medially into the mandible. The third foramen occurs in the middle of the surangular body, although, this opening could result from breakage of thin bone in this region. However, a foramen occurs in this location in *Thescelosaurus neglectus* (Boyd, 2014), suggesting that the opening in *Muttaburrasaurus langdoni* could be a foramen. A fourth foramen is provisionally identified as a small slot passing through the surangular ventral to the foramen in the middle of the surangular body (Fig. 49A, Fig. 49D). The surangular forms the lateral part of the dorsally upturned retroarticular process, upon which, inserted the mandibular musculature, including the *pterygoideus ventralis* on the posterolateral face and the depressor mandibulae and *pterygoideus dorsalis* on the posteromedial face (based on Ostrom, 1961; see also Nabavizadeh & Weishampel, 2016; Rybczynski et al., 2008). The surangular closely matches that of *Thescelosaurus neglectus* (based on Boyd, 2014).

#### Coronoid

**Figure 50:**
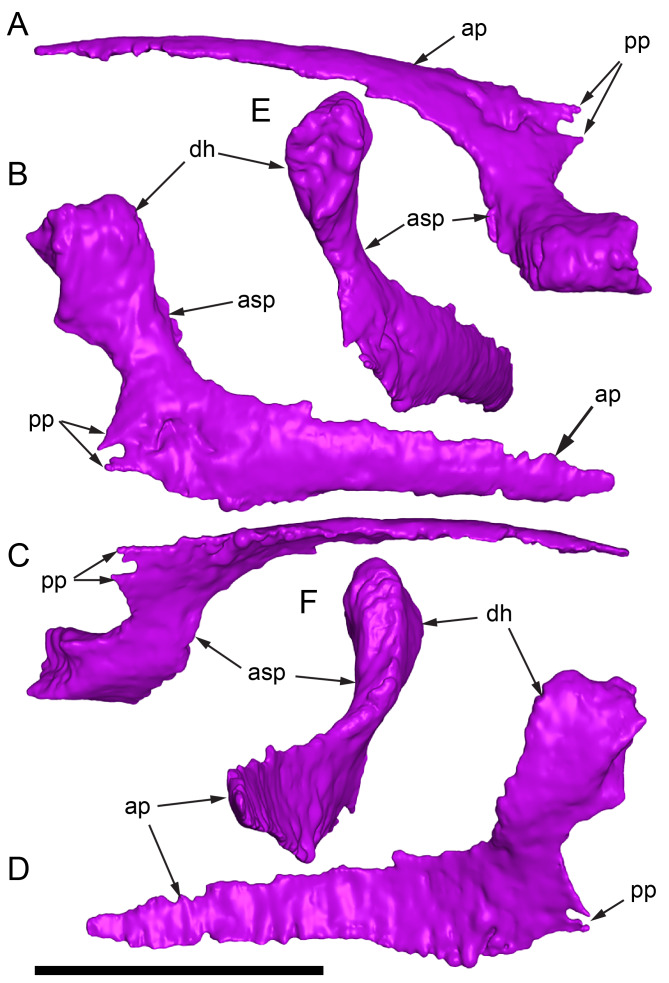
Volume rendered model of the *Muttaburrasaurus langdoni* (QMF6140) left coronoid. (A–F) Left coronoid in (A) dorsal, (B) lateral, (C) ventral, (D) medial, (E) posterior and (F) anterior views. Abbreviations: ap, anterior process; asc, ascending process; asp, ascending process; dh, dorsal head; pp, posterior process. Scale bar equals 10 cm. MorphoSource DOI: 10.17602/M2/M787685.

Both coronoids are preserved attached to the anteroposteriorly convex medial surface of the dentaries. The left coronoid is described (Fig. 50). The coronoid is thin and L-shaped (Fig. 50). The ventral process located ventral to the functional dentary crowns, is strap-like and tapered anteriorly where it extends to a point coinciding with the twelfth alveolus (Figs. 43A, 3B). The posterior process is short, triangular and notched at its posterior end, which aligns with posteromedial end of the dentary (Figs. 43B, 43C, 50). The thickened ascending process closely abuts the posteromedial surface of the coronoid process of the dentary where it forms a narrow, bar-like neck, dorsal to which, it expands anteroposteriorly forming a lobate dorsal head recurving medially (Figs. 43A–43D, 50). The posterodorsal portion of the head is visible laterally. The posterior margin of the head is grooved where it abuts the surangular (Figs. 43B, 43C, 50). The posterior margin of the neck forms a concave embayment that borders the anteromedial portion of the adductor fossa (Figs. 43B, 50). The dorsal head is located posteromedial to the coronoid process on the dentary; thus, differing from the position of the coronoid head located medial to the coronoid process on the dentary in *Hypsilophodon foxii* (Galton, 1974), *Tenontosaurus tilletti* (Thomas, 2015), *Zalmoxes shqiperorum* Godefroit, Codrea & Weishampel, 2009 and less derived styracosternans, such as *Iguanodon bernissartensis* (Norman, 1980). The coronoid of *Muttaburrasaurus langdoni* differs from that of *Mantellisaurus atherfieldensis* (Norman, 1986) and *Zalmoxes shqiperorum* (Godefroit, Codrea & Weishampel, 2009), where the bone is reduced to a teardrop-shaped plate, medial to the head of the coronoid process of the dentary. The morphology of the *Muttaburrasaurus langdoni* coronoid closely resembles that of *Tenontosaurus tilletti* (Thomas, 2015) and *Thescelosaurus neglectus* (Boyd, 2014), although the ventral process is absent in these taxa. The strap-like anterior process, like those of *Changchunsaurus parvus* (Jin et al., 2010), *Hypsilophodon foxii* (Galton, 1974), *Psittacosaurus mongoliensis* (Sereno, 1987), *Thescelosaurus neglectus* (Boyd, 2014) and heterodontosaurids (Sereno, 2012), suggests, in this aspect, stronger affinities between *Muttaburrasaurus langdoni* and early diverging ornithischians, than with more derived ornithopods. The coronoid has yet to be described in any taxon presently assigned to Elasmaria. The coronoid could be absent in hadrosauroids (Prieto-Márquez, 2010b, character 58[0]), distancing *Muttaburrasaurus* from that group.

#### Prearticular, angular, articular

**Figure 51:**
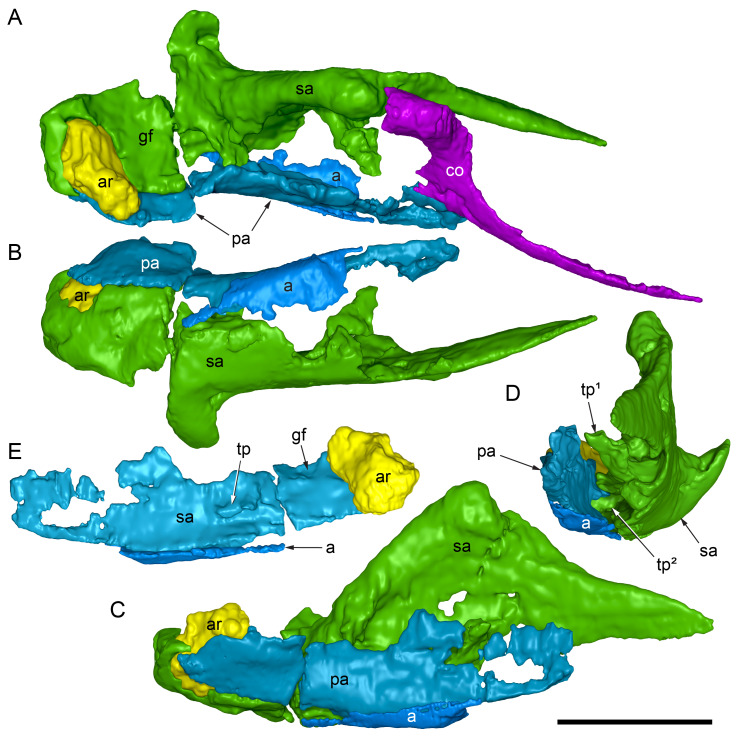
Volume rendered model of the *Muttaburrasaurus langdoni* (QMF6140) posterior bones of the left mandibular corpus. (A) Prearticular, angular, articular, surangular and coronoid complex in dorsal view. (B–D) Surangular, prearticular, angular and articular complex in (B) ventral, (C) medial and (D) anterior views. (E) Prearticular, angular and articular complex in lateral view. Abbreviations: a, angular; ar, articular; co, coronoid; gf, glenoid fossa of mandible; pa, prearticular; sa, surangular; tp, transverse process (of prearticular); tp 1 , anterior transverse process of surangular; tp 2 , posterior transverse process of surangular (contacting transverse process of prearticular). Scale bar equals 10 cm. MorphoSource DOI: 10.17602/M2/M787673; 10.17602/M2/M787685; 10.17602/M2/M787679; 10.17602/M2/M787682; 10.17602/M2/M787676.

The posterior portion of the left prearticular is closely associated with a ventral portion of the left angular and left articular (Fig. 51). Owing to poor preservation and matrix differentiation difficulties, anterior differentiation between the left prearticular and angular is uncertain. Furthermore, association between the left prearticular, angular, dentary and coronoid cannot be ascertained and the splenial is not preserved or identified. Anterior to the glenoid, the prearticular forms a vertically oriented sheet (Fig. 51B). The bone is medially inflected at the glenoid fossa where it forms a dorsoventrally compressed plate for the medial portion of the fossa (Fig. 51A). The small portion of the angular preserved overlaps the ventral margin of the prearticular, as noted by Bartholomai & Molnar (1981). The articular (an element of the splanchnocranium) forms the posterior wall of the glenoid fossa and the medial portion of the retroarticular process (Fig. 51). The arrangement of these mandibular bones is consistent with early diverging ornithischians up to less derived Styracosterna. The prearticular could be absent in hadrosauroids (Prieto-Márquez, 2010b, character 59[0]).

#### Ceratobranchial

**Figure 52:**
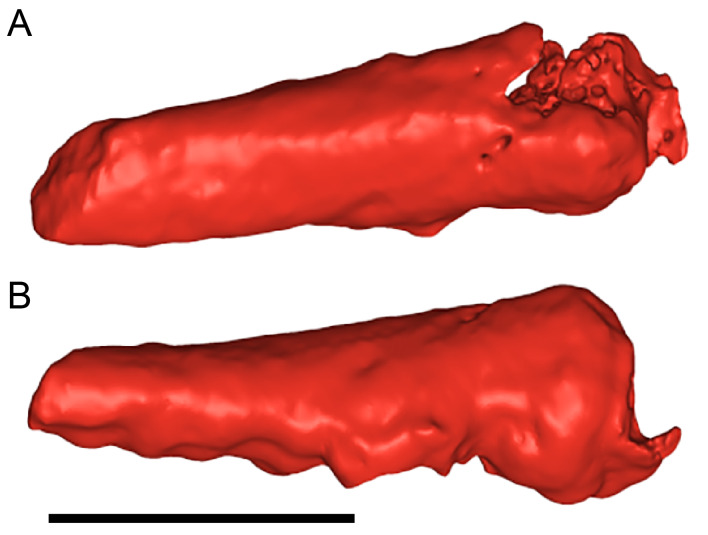
Volume rendered model of the *Muttaburrasaurus langdoni* (QMF6140) left ceratobranchial. (A–B) Left ceratobranchial in (A) medial and (B) ventral views. Scale bar equals five cm. MorphoSource DOI: 10.17602/M2/M787688.

The left ceratobranchial of the hyoid complex (an element of the splanchnocranium) is preserved medial to the left prearticular and coronoid (Fig. 52), as in *Theiophytalia kerri* (Brill & Carpenter, 2006; Gilmore, 1909). The bone is rounded and rod-like, incomplete posteriorly and likely displaced from its life position more ventrally; possibly associated with the larynx, as inferred for *Jeholosaurus shangyuanensis* (Li, Zhou & Clarke, 2018). The proximal end is expanded and thickened. The thickened ends of the paired bones could have attached to a midline basihyal, as reported in the ankylosaurid *Pinacosaurus grange* (Hill et al., 2015), which is not preserved in *Muttaburrasaurus langdoni* and could have been a cartilaginous element. The part preserved gives no indication of being curved, unlike those of *Iguanodon bernissartensis* and *Theiophytalia kerri* (Brill & Carpenter, 2006; Gilmore, 1909), or having been kinked, as in *Ouranosaurus nigerensis*. However, the lack of the posterior portion restricts a more definitive assessment.

**Table 2:** Table of neural endocast measurements for *Muttaburrasaurus langdoni* (QMF6140).

Endocast, anteroposterior length, dorsoventral height (mm)	223.0/120.0
Paired olfactory bulbs, anteroposterior length/transverse width (mm)	62.8/58.2
Cerebrum, anteroposterior length/transverse width (mm)	80.1/57.0
Anterior semicircular canal (ASC), length (mm)	55.8(r), 57.8(l)
Posterior semicircular canal (PSC), length (mm)	38.1(r), 36.9(l)
Endosseous cochlear duct (ECD), length (mm)	19.3(l), 21.5(r)
Foramen magnum, dorsoventral height/transverse width (mm)	42.0/45.0
Endocast volume (cm^3^), including olfactory apparatus	346.1
Endocast volume and mass (cm^3^, g), excluding olfactory apparatus and brain stem posterior to cranial nerve XII	294.6
Cerebral volume and mass (cm^3^, g)	121.9 ± 10%

### Palaeoneurology

#### Gross encephalic organization and description

**Figure 53:**
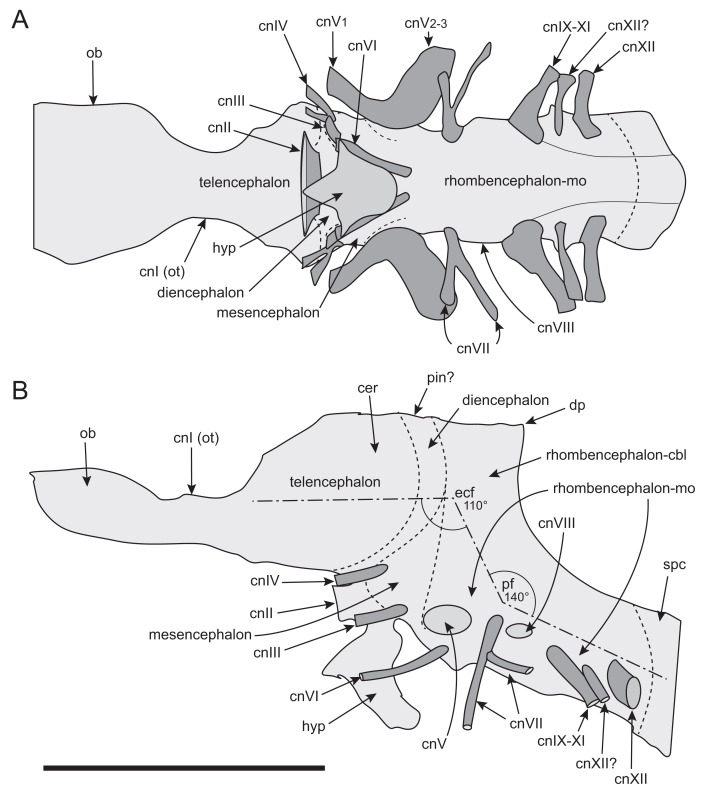
Organization of the *Muttaburrasaurus langdoni* (QMF6140) endocranium, determined from the volume rendered encephalic endocast. Schematic in (A) ventral and (B) left lateral views. Dashed lines indicate hypothesised margins of the encephalic regions. Long dashed lines indicate encephalic axes. Abbreviations: cer, cerebrum; cbl, region of cerebellum; cn#, cranial nerve and number; dp, dural peak; ecf, encephalic flexure and angle; hyp, hypophysis; mo, region of medulla oblongata; ob, olfactory bulb; ot, olfactory tract; pin, pineal; pf, pontine (medullary) flexure and angle; spc, spinal cord. Scale bar equals 10 cm.

The encephalic endocast is remarkably well preserved with the gross external morphology of the brain, inner ear, endocranial vasculature and cranial nerves discernible within the limits of the medical CT scan resolution. Primary dimensions and volumes of the endocast are provided in Table 2. The external surface of the endocast represents the dural envelope/sinus surrounding the brain and not the brain surface itself (*e.g.*, Ferreira-Cardoso et al., 2017), unlike the condition in birds, where the brain fills nearly all the neural fossa (Keirnan et al., 2025). Gross organization and bauplan of the brain, as described herein (Fig. 53), was guided by the endocast shape and endocranial landmarks of the cranial nerves, vascular foramina and regions of the pituitary, epiphysis (pineal) and endosseous labyrinths. Using this methodology, the three main regions of the brain were identified, being the prosencephalon (= diencephalon (forebrain) and telencephalon), mesencephalon (= midbrain) and rhombencephalon (= hindbrain). From these regions, the angles of encephalic flexure were estimated (Fig. 53). Subregions of the brainstem were further identified, including the telencephalon and diencephalon of the prosencephalon and the cerebellum and medulla oblongata of the rhombencephalon (Fig. 53). Terminologically, the rhombencephalon and the metencephalon are almost synonymous. However, the metencephalon encompasses the pons in mammals bridging the mesencephalon (Striedter & Northcutt, 2019). For this reason, rhombencephalon is used here with preference. Although the descriptions of the encephalic regions that follow are descriptions of three-dimensional shapes formed by the matrix infill (*i.e.,* the endocast), for expediency and to avoid continual caveats, we refer to the endocast structures as if they were the soft tissues themselves. In the descriptions of the paired cranial nerves, brief outlines of their assumed functions in *Muttaburrasaurus langdoni* are provided for context, based on the understanding from extant amniotes (*e.g.*, Butler & Hodos, 2005; Nieuwenhuys, Donkelaar & Nicholson, 1998). For a detailed outline of brain centre and cranial nerve functions, the reader is directed to Butler & Hodos (2005) or Striedter & Northcutt (2019). As the evolutionary organization of the central nervous system across Amniota is considered to have been highly conservative (Butler & Hodos, 2005), we anticipate that the functions of the 12 cranial nerves in *Muttaburrasaurus langdoni* would not have deviated from the basic functions in extant amniotes.

#### Telencephalon

**Figure 54:**
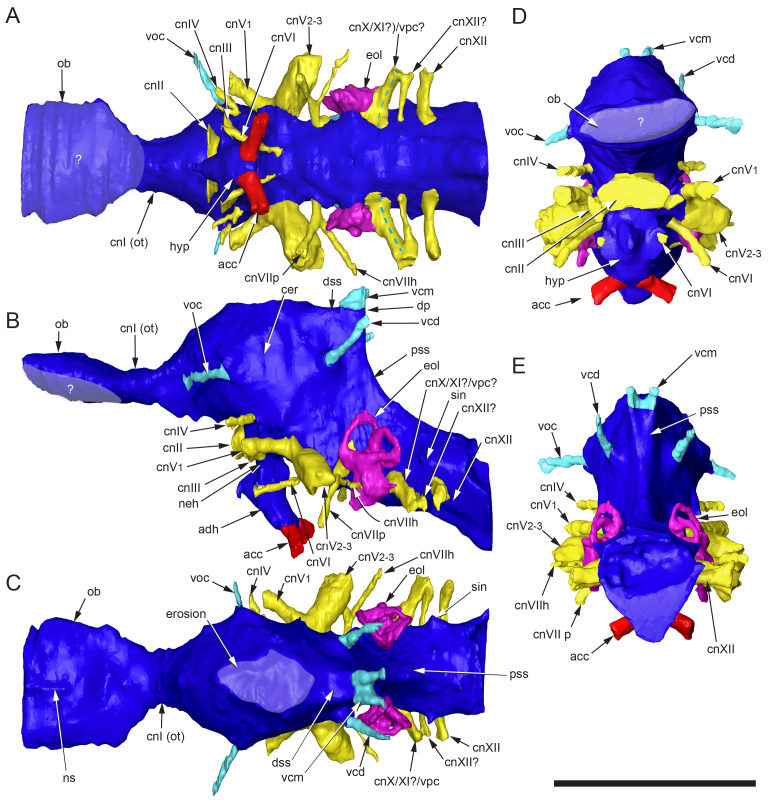
Volume rendered model of the *Muttaburrasaurus langdoni* (QMF6140) neural endocranium. (A–B) Neural endocranium in (A) ventral, (B) left lateral, (C) dorsal, (D) anterior and (E) posterior views. Colours: dark blue, endocranium; yellow, cranial nerves (not including the olfactory tectum); light blue, veins; red, arteries; purple, endosseous labyrinth. Abbreviations: acc, cerebral carotid artery; adh, adenohypohysis; asp, sphenopalatine artery; dp, dural peak; dss, dorsal sagittal sinus; eol, endosseous labyrinth; hyp, hypophysis; in, infundibulum; cn#, cranial nerve and number (h = hyomandibular, p = palatine); neh, neurohypophysis; ns, groove of nasal septum; ob, olfactory bulb; ot, olfactory tracts; pss, posterior sagittal sinus; vcd, vena capitis dorsali; vcm, vena capitis medialis; vdc, dorsal cephalic vein; vpc, posterior cephalic vein; sin, sinus; vpmc, posterior middle cerebral vein; voc, orbitolcephalic vein; ?, uncertain region or extent of volume. Scale bar equals 10 cm. MorphoSource DOI: 10.17602/M2/M788215.

The telencephalic region of the prosencephalon comprises the olfactory apparatus (= paired olfactory bulbs and tracts) and the cerebrum (= cerebral cortex) (Figs. 53, 54). The paired olfactory tracts (cnI) extend anterodorsally from near the ventral margin of the cerebral hemispheres. The olfactory tracts expand laterally and dorsally into the olfactory bulbs. The olfactory bulbs are slightly broader transversely than the cerebrum (Figs. 54A, B; Table 2). A shallow sagittal furrow anteriorly on the dorsal surface slightly divides the olfactory bulb endocast into left and right sides (Fig. 54C). A similar furrow, or anterior notch, is apparent on the endocasts of other dinosaurs (Button & Zanno, 2023; Evans, Ridgely & Witmer, 2009; Müller, 2022; Paulina-Carabajal & Currie, 2012; Sakagami & Kawabe, 2020; Witmer et al., 2008)) and potentially represents the posterior-most part of the cartilaginous nasal septum, as demonstrated in extant crocodilians and the homologous, cartilaginous median septum interpreted in theropods, such as *Tyrannosaurus rex* and maniraptoriforms (Ali et al., 2008; = mesethmoid in birds and non-avialan dinosaurs, Bourke et al., 2014). As in crocodilians, the nasal cartilage likely attached to the median septum in theropods and marks the anterior limit of the olfactory bulbs (Ali et al., 2008). From this information, we consider the furrow anteriorly between the olfactory bulbs as delimiting the anterior extent of the olfactory bulbs. The olfactory nerves would have coursed through fenestra in the olfactory cartilage from the olfactory bulbs and olfactory epithelium to the olfactory capsule (based on Ali et al., 2008). Ventrally, the olfactory bulbs would have been contained in a cartilaginous sphenethmoid trough of the ethmoid complex anterior to the ossified orbitosphenoids (= ossified posterior parts of the *planum supraseptale*, which also formed the sphenethmoid (presphenoid) trough; Ali et al., 2008; Bourke et al., 2014) (see further in “Results and Discussion”). As the sphenethmoid trough was not mineralised bone (evident in the CT imagery), the ventral extent of the olfactory bulbs is unknown, and their volume cannot be determined. However, for the volume rendered reconstruction (Fig. 54), the ventral surface of the olfactory bulbs was constrained to the dorsoventral depth of the olfactory tracts delimited by the ossified orbitosphenoid (see Fig. 34). The septum of the sphenethmoid ventral to its trough would have attached in the dorsal groove of the parasphenoid (Fig. 42C) positioned between the paired descending processes of the vomera.

The cerebrum is poorly expanded dorsally and transversely (Fig. 54), differing from the relatively bulbous cerebra in Styracosterna (*Iguanodon bernissartensis* and hadrosaurs; Cruzado-Caballero et al., 2015; Evans, Ridgely & Witmer, 2009; Hopson, 1979; Lauters et al., 2012; Lauters, Vercauteren & Pascal, 2023; Ostrom, 1961). The posterior extent of the cerebrum and diencephalon are informed by the positioning of the trochlea nerve (cnIV), which issues from the mesencephalon (Fig. 53). The cerebrum is dorsoventrally deep; differing from the relatively shallow, albeit bulbous, form in *Dysalotosaurus lettowvorbecki*, *Hypsilophodon foxii* and *Tenontosaurus tilletti* (Galton, 1989; Lautenschlager & Hübner, 2013). The cerebrum bears more resemblance in its appearance to that of the large-bodied theropod, *Tyrannosaurus rex* (Witmer & Ridgely, 2009), than that of large-bodied styracosternans.

#### Diencephalon

The diencephalic region of the prosencephalon in mature vertebrates differentiates into several neural centres of the brain, including the epithalamus, epiphysis (pineal), thalamus, hypothalamus, optic chiasm and infundibulum, among other centres (*e.g.*, Striedter & Northcutt, 2019). However, only the regions of the infundibulum and optic chiasm are identified on the endocast of the *Muttaburrasaurus langdoni* holotype. The pituitary gland (= hypophysis) is not strictly a region of the brain as its development involves the union of two embryonic sources (Striedter & Northcutt, 2019), but for the purposes of this description, the description of its endocast is provided here. One source of the pituitary is the dorsal outgrowth and pinching out of Rathke’s pouch from the stomodeum (which also forms the mouth) to become the adenohypophysis. The other is the ventral outgrowth of the infundibulum from diencephalic floor to become the neurohypophysis. The neurohypophysis connects the hormonal secretory centre of the hypothalamus (Butler & Hodos, 2005; Striedter & Northcutt, 2019; Romer, 1955) (Fig. 54). The narrow neck of the neurohypophysis is the infundibular stalk, ventral to the optic chiasma, from where, the paired optic nerves (cnII) issue. The adenohypophysis expands anteroventral to the neurohypophysis and into the *sella turcica* (= pituitary fossa; Figs. 34D, 54A, 54B, 54D). The conical ventral region of the adenohypophysis is the *pars distalis* (anterior lobe) (based on information in Butler & Hodos, 2005; Striedter & Northcutt, 2019) (Fig. 54B). The pineal protuberance dorsally on the diencephalon is not identified, as this region of the endocast has been exposed dorsally through erosion of the parietals. Relative to the cerebrum, the diencephalon in *Edmontosaurus regalis* was described as transversely narrow (*sensu* Ostrom, 1961), although this region was considered by the latter author as the “mesencephalon”. However, transverse narrowing of the diencephalon, as assumed in *Edmontosaurus regalis*, is not apparent in *Muttaburrasaurus langdoni*.

#### Mesencephalon

The mesencephalon is superficially identified as a wedge-shaped region ventrally between the diencephalon and the rhombencephalon (Fig. 53). The pituitary-infundibular region of the diencephalon adjoins the anteroventral margin of the mesencephalon. The tectum (optic tecta), motor tegmentum and isthmus are within the mesencephalon (*e.g.*, Butler & Hodos, 2005; Striedter & Northcutt, 2019). The tectum is involved with auditory, visual and somatosensory functions that form a map of sensory space (*sensu* Butler & Hodos, 2005). Lateral expansion of the tectum is not apparent on the endocast. The tegmentum, which primarily controls motor functions (receives incoming sensory information and actions outgoing motor functions to and from the prosencephalon) is located ventrally at the floor of the mesencephalon and runs into the medulla oblongata (based on Butler & Hodos, 2005) (Fig. 53). The oculomotor (cnIII) and trochlea (cnIV) nerves, which enervate extrinsic muscles of the eye, issue from the ventrolateral region of the mesencephalon (Butler & Hodos, 2005) and assist identification of this region on the endocast (Figs. 53, 54).

#### Rhombencephalon

The cerebellum and medulla oblongata are centres within the rhombencephalon, with the cerebellum positioned superior to the medulla oblongata (Hopson, 1979; Striedter & Northcutt, 2019). The anterior extent of the cerebellum would have approached the diencephalic and superior mesencephalic margins (Figs. 53, 54). The cerebellum is estimated to fall within the transversely narrow dorsal (longitudinal) and posterior (occipital) sagittal sinus regions (Figs. 54B, 54C, 54E) as in *Tenontosaurus tilletti* (Thomas, 2015) and *Tyrannosaurus rex* (Witmer & Ridgely, 2009). The cerebellum lacks any distinct lateral or dorsal expansion (Figs. 53, 54). Relative to the prosencephalon, the cerebellum is transversely narrow, as in *Dysalotosaurus lettowvorbecki*, *Hypsilophodon foxii*, *Thescelosaurus neglectus* and *Tenontosaurus tilletti* (Galton, 1989; Thomas, 2015). The cerebellum is similarly narrow in *Tyrannosaurus rex* (Hopson, 1979; Witmer & Ridgely, 2009). The pronounced dural peak forms an angle of 88°, resembling that in *Dysalotosaurus lettowvorbecki* (Galton, 1989) and *Tyrannosaurus rex* (Witmer & Ridgely, 2009) and differing from the more obtuse angle in hadrosaurids (see further under “Supraoccipital” above). A slight protuberance on the endocast in the region of the floccular fossa medial to the semicircular canals presents on the right side but is absent on the left. The distal end of the right floccular protuberance is located at the prootic-opisthotic margin near the middle of the anterior semicircular canal arch, with similar morphology reported in *Tenontosaurus tilletti* (Thomas, 2015). A thin wall of bone separates the floccular fossa and the vestibule of the osseous labyrinth (Fig. 37), as in *Tenontosaurus tilletti* (Thomas, 2015). Relative to the cerebellum, the medulla oblongata expands laterally, ventral to the floccular fossae (Fig. 54).

**Figure 55:**
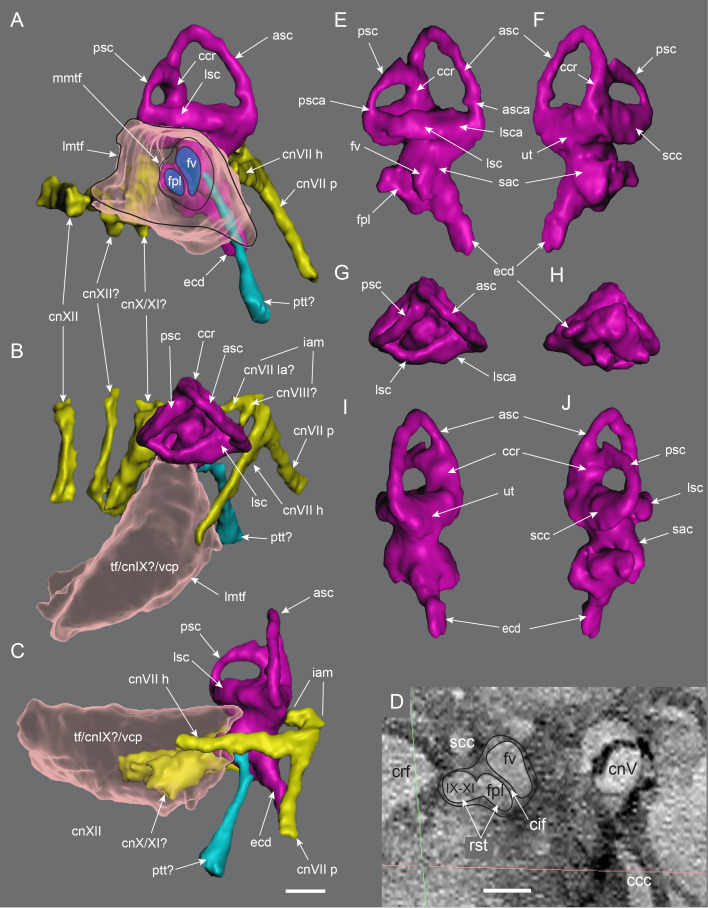
Volume rendered model and sagittal plane radiograph of the *Muttaburrasaurus langdoni* (QMF6140) right endosseous labyrinth. (A–C) Volume rendered model of tympanic fossa, endosseous labyrinth and myelencephalic cranial nerves in (A) lateral, (B) dorsal and (C) anterior views. (D) Sagittal plane radiograph in region of the *fenestra vestibuli*, *fenestra pseudorotunda* (*perilymphatica*) and *apertura anterialis* oblique to the long axis of and medial to the tympanic fossa. (E–J) Volume rendered model of endosseous labyrinth in (E) lateral, (F) posterior, (G) dorsal, (H) ventral (I) anterior and (J) posterior views. Abbreviations: amp, ampulla; asc, anterior semicircular canal; asca, region of ampulla of the anterior semicircular canal; ccr, common crus; ccc, cerebral carotid canal; cif, *crista interfenestralis*; crf, cranial fossa; ecd, endosseous cochlear duct; fpl, *fenestra perilymphaticum*; fv, *fenestra vestibuli*; iam, internal auditory meatus; lmtf, lateral margin of tympanic fossa; lsc, lateral semicircular canal; lsca, region of ampulla of the lateral semicircular canal; mmtf, medial margin of tympanic fossa; n#, cranial nerve and number (h = hyomandibular, p = palatine); ncf, neurocranial fossa; psc, posterior semicircular canal; psca, region of the ampulla of the posterior semicircular canal; ptt, pharyngotympanic tube; rst, *recessus scalae tympani*; sac, region of saccule; scc, secondary common crus; tf, tympanic fossa; ut, region of utricle; vcp, posterior cephalic vein. Scale bar equals one cm. MorphoSource DOI: 10.17602/M2/M788215.

Cranial nerves cnV–VIII issue from the anterior end of the medulla oblongata, which in mammals is the region of the pons (Butler & Hodos, 2005; Striedter & Northcutt, 2019) (Figs. 53, 54, 55). The largest of the cranial nerves, the trigeminal (cnV_1-3_) branches in two rami. The ophthalmic branch (cnV_1_) of the trigeminal nerve, which typically provides afferent sensory innervation of the eye and the cranial integument, including the teeth, mouth and nasal passages in tetrapods (Butler & Hodos, 2005; George & Holliday, 2013; Sneddon, 2002; Striedter & Northcutt, 2019), courses anterolaterally and internally within the laterosphenoid (Figs. 33, 34, 37B). Differing from *Muttaburrasaurus langdoni*, cnV_1_ extended in a groove laterally on the laterosphenoid in *Edmontosaurus regalis* (Xing, Mallon & Currie, 2017), most lambeosaurines (Evans, 2010; Evans, Ridgely & Witmer, 2009), *Tenontosaurus tilletti* (Galton, 1989; Thomas, 2015), *Hypsilophodon foxii* (Galton, 1989) and most likely in *Camptosaurus dispar* (Gilmore, 1909) and *Dysalotosaurus lettowvorbecki* (Sobral, Hipsley & Müller, 2012) and in the early diverging neornithischians, *Thescelosaurus neglectus* (Boyd, 2014; Galton, 1989) and *Lesothosaurus diagnosticus* (Porro, Witmer & Barrett, 2015). Cranial nerve V_1_ also appears to have coursed externally on the laterosphenoid in *Heterodontosaurus tucki* (Norman et al., 2011), suggesting the plesiomorphic condition in ornithischians. An internalised cnV_1_ was previously considered a *Lambeosaurus* autapomorphy (Evans & Reisz, 2007); however, the condition is convergent with *Muttaburrasaurus langdoni*. The path of cnV_1_ is uncertain in *Iguanodon bernissartensis*, *Mantellisaurus atherfieldensis* and *Ouranosaurus nigeriensis*. Like *Muttaburrasaurus langdoni*, an internalised cnV_1_ was reported in the centrosaurine *Pachyrhinosaurus lakustai* (Witmer & Ridgely, 2008b), further suggesting that braincase thickness in neornithischians could be linked to internalisation of cnV_1_, as well as the hyomandibular branch of the facial nerve (cnVII_h_; see further below). Internalisation of cnV_1_ reportedly occurred in saurischians, such as the titanosaur *Sarmientosaurus musacchioi* (Martínez et al., 2016) and Cretaceous theropods such as *Erlikosaurus andrewsi* (Lautenschlager et al., 2012), *Troodon* and tyrannosaurids (Currie & Zhao, 1993) such as *Tyrannosaurus rex* (Witmer & Ridgely, 2009; Witmer & Ridgely, 2010), but not in the carcharodontosaurian *Giganotosaurus carolinii* (Coria & Currie, 2002). Variability in this feature is therefore apparent within Ornithischia and Saurischia and was potentially linked to cranial thickness in both groups. The afferent maxillary branch (cnV_2_) and predominantly efferent mandibular motor branch (cnV_3_) (following Butler & Hodos, 2005; Higashiyama & Kuratani, 2014) extended as a single stem laterally through the laterosphenoid-prootic margin (Figs. 33, 34).

The abducens nerve (cnVI), which innervates extraocular muscles of the eye (Butler & Hodos, 2005), is positioned ventral to cnV and extends anteriorly through the basisphenoid lateral to the pituitary expansion (see “Basisphenoid” above; Figs. 33C, 34B, 40E, 41E, 42C, 42F, 53 and 54). The facial nerve (cnVII), which issues from the medulla oblongata anteriorly adjacent to the vestibulocochlear nerve (cnVIII), has complex afferent, efferent and parasympathetic functions, among which include efferent innervation of facial, stapedius, hyoid and digastric muscles and afferent innervation of the taste buds in the tongue and palate and parasympathetic innervation of the salivary and lacrimal glands (following Butler & Hodos, 2005; Oelrich, 1956). Proximally, cnVII courses through the internal auditory meatus in the prootic, along with the vestibulocochlear nerve (cnVIII) (Figs. 34D, 37B, 38M, 55H–55J). A thin branch of cnVII could extend into the endosseous labyrinth (Figs. 37B, 55B) as the labyrinthine segment (cnVI_l_), as described in humans (Ho et al., 2015; Wadin & Wilbrand, 1987). Alternatively, however, this canal was possibly vascular, or for a second branch of the vestibulocochlear nerve (cnVIII; see below). In the internal auditory meatus, the facial nerve divides into two branches, interpreted as the hyomandibular (cnVII_h_) and palatine (= vidian; cnVII_p_) rami (following terminology in Oelrich, 1956; Willard, 1915; = dorsal and ventrolateral rami in Butler & Hodos, 2005) (Fig. 37; see also under “Prootic” above). The palatine ramus of the facial nerve (cnVII_p_), being the thicker of the two rami, extended ventrolaterally in a long canal to exit in the dorsal roof of the cerebral carotid canal (Figs. 38M, 38O). The hyomandibular branch (cnVII_h_) emerged externally from the braincase in the anterodorsal region of the tympanic fossa (Figs. 34C, 37, 38M, 38O), suggesting its course continued internally in the visceral portion of the tympanic fossa past the tympanic membrane and columella. A branch of cnVII_h_ potentially innervated the stapedius muscle, as occurs in extant reptiles and birds and the homologous feature in mammals, being the *tensor tympani* (Saunders et al., 2000). The passage of the hyomandibular and palatine branches of the facial nerve (cnVII_h,p_) have been reported in *Mantellisaurus atherfieldensis* (Norman, 1986) and some hadrosaurids (Evans, Ridgely & Witmer, 2009; Xing, Mallon & Currie, 2017). However, unlike *Muttaburrasaurus langdoni*, where branching of the facial nerve occurred within the neurocranium, branching occurs externally and laterally in these styracosternans.

The vestibulocochlear nerve (cnVIII) has two rami: the vestibular (cnVIII_v_) and cochlear (cnVIII_c_) rami (Figs. 37, 55). The foramen in the prootic dorsal to the postulated passage of the labyrinthine segment of the facial nerve (cnVII_l_) and slightly anterior to the latter, is alternatively for rami of cnVIII (Fig. 37). The foramen extends a short distance within the prootic-opisthotic to the vestibule of the endosseous labyrinth (Fig. 37B). A similar arrangement of these two cranial nerves at their origin on the endocast was proposed in *Dryosaurus altus*, *Hypsilophodon foxii* and *Thescelosaurus neglectus* (Galton, 1989).

Cranial nerves cnIX–XIII issue from the posterior end of the medulla oblongata (Figs. 53, 54) (Striedter & Northcutt, 2019). The glossopharyngeal nerve (cnIX), vagus nerve (cnX) and possibly the accessory nerve cnXI, initially course laterally through the *fissura metotica*, which divides into two sections at the *recessus scalae tympani* consistent with anatomy in sauropsids (Rieppel, 1985) (Fig. 37A; see further under “Otoccipital” above). The glossopharyngeal nerve (cnIX) courses into the anterior division—the metotic foramen—alongside the *fenestra pseudorotunda*. The vagus nerve (cnX) and its accessory branch (cnXI) courses through the posterior division—the vagus foramen—potentially along with the posterior cephalic vein. In derived tetrapods, cnIX and cnX innervate complex afferent, efferent and parasympathetic functions, including to musculature of the pharynx (Butler & Hodos, 2005). Cranial nerve IX innervates the taste buds, pharynx and salivary glands and in tetrapods particularly, motor functions of the tongue, while cnX, innervates the taste buds, pharynx, larynx and visceral internal organ motor functions of the thorax and abdomen (Butler & Hodos, 2005). Identification of the accessory spinal nerve, cnXI, as a true cranial nerve has been questioned, with the possibility it lacks a cranial root, as functions originally attributed to cnXI could be innervated instead by cnX (Benninger & McNeil, 2010; Campos et al., 2010; Tada & Kuratani, 2015; see also Butler & Hodos, 2005). The two posterior-most cranial nerves are assessed as rami of the hypoglossal nerve (cnXII) (*e.g.*, Hopson, 1979) (Figs. 24, 39—41). Together with cnX, the hypoglossal nerve innervates muscles of the tongue (Butler & Hodos, 2005).

#### Flexures

The pontine and cephalic flexures were determined by connecting centralised axes of the medulla oblongata and prosencephalon (Fig. 53). Connection of these axes was taken between the region corresponding to the junction of the mesencephalic isthmus and the prosencephalic axis (region of the pons in the metencephalon of mammals). From these axes, the angles of pontine and cephalic flexures were estimated at ∼140° and ∼110°, respectively. The acute angles of flexure are consistent with early diverging neornithischians, including less-derived ornithopods and differ from the shallow to near absent flexures in derived iguanodontians, where enlargement and dorsal protrusion of the cerebrum, which is not apparent in *Muttaburrasaurus langdoni*, appear linked (Cruzado-Caballero et al., 2015; Giffin, 1989; Hopson, 1979). The acute angle of the dorsal sagittal sinus at the dural peak (see also under “Supraoccipital” above; Fig. 34D) is a result of the acute angles of pontine and cephalic flexures. The telencephalic axis is roughly parallel to both the horizontal semicircular canals and cranial roof and the myelencephalic axis is angled at 32° relative to the plane of the horizontal semicircular canals (Fig. 53).

#### Cranial vasculature

The cranial vasculature was interpreted from canals through or between bones of the braincase (Figs. 33–35, 36, 37, 40, 54). Each of the colateral cerebral carotid arteries coursed through the cerebral carotid canal to enter the cranial fossa through the cerebral carotid artery foramen leading to the lateral hypophysial sinuses ventrally in the *sella turcica* (based on: Aslan et al., 2006; Porter & Witmer, 2016; Porter, Sedlmayr & Witmer, 2016; Porter & Witmer, 2015; Sampson & Witmer, 2007) (Figs. 33C, 33D, 38O, 40C, 54). The cerebral carotid arteries and veins likely coursed together (Porter, Sedlmayr & Witmer, 2016). Based on information in Porter & Witmer (2015), the sphenopalatine artery potentially branched from the cerebral carotid artery in the cerebral carotid canal (*i.e.,* external to the cranial fossa) and coursed ventrally with the palatine branch of the facial nerve (cnVII_p_) after its exit in the dorsal roof of the cerebral carotid canal (see Fig. 38O). The sphenoidal and internal ophthalmic arteries (= orbital artery; Porter & Witmer, 2016) potentially branched from the cerebral carotid artery in the *sella turcica* as in birds (based on: Baumel, 1993; Baumel & Witmer, 1993; Porter & Witmer, 2016; Sampson & Witmer, 2007) and exited the braincase anteriorly through foramina or slots between the orbitosphenoid and parasphenoid (Figs. 33C, 34A, 34D, 40, 42C, 42G, 42F; see also “Parasphenoid” above). Of the primary paired cerebral veins, the orbitocerebral vein likely exited anterolaterally from the lateral cerebral region of the telencephalon at a foramen between the laterosphenoid and orbitosphenoid, the *vena capitis dorsalis* (= dorsal cephalic vein; posterior middle cerebral vein, Paulina-Carabajal, Ezcurra & Novas, 2019) potentially branched posterodorsally from the mid-metencephalic region and exited posterodorsally from the mid-lateral cerebellar region of the metencephalon through the supraoccipital and the *vena capitis medialis* likely exited posteriorly through the supraoccipital at the dural peak (Figs. 34D, 35K, 36C, 54; see further under “Supraoccipital” and “Laterosphenoid” above). The posterior cephalic vein, a branch of the jugular, likely exited laterally from the myelencephalic region through the vagus canal, posterior to the endosseous labyrinth and *crista tuberalis* (see also “Otoccipital” above) (Figs. 34C, 37A).

#### Middle ear, inner ear and associated cranial nerves

Anatomy of the middle ear, osseous labyrinth and associated cranial nerves was assessed from volume rendering of the matrix filled endosseous elements (Figs. 54, 55). In sauropsids, the columella (= stapes) attaches laterally at the tympanum (ear drum) and medially at the fenestra vestibuli (oval window) (Fig. 55), *via* a cartilaginous accessory columella (*e.g.*, Saunders et al., 2000; Tucker, 2017). The columella is unknown in *Muttaburrasaurus langdoni*; however, numerous columellae preserved in the tympanic regions of dinosaurs such as *Camarasaurus lentis* (Witmer et al., 2008), *Corythosaurus casuarius* (Colbert & Ostrom, 1958), *Dromeosaurus albertensis* (Huene, 1926), *Plateosaurus fraasianus* (Huene, 1926), *Psittacosaurus* sp. (You & Dodson, 2004), *Spinophorosaurus nigerensis* (Knoll et al., 2012); *Thescelosaurus neglectus* (Boyd, 2014; Button & Zanno, 2023), possibly *Jeholosaurus shangyuanensis* (Barrett & Han, 2009) and *Lesothosaurus diagnosticus* (Sereno, 1991), indicate that the tympanum was positioned near the integumentary surface of the head, near the otic notch (You & Dodson, 2004) at the posterior margin of the quadrate column and the distal ventromedial margin of the paroccipital process (Figs. 5A, 38B, 38D). The cartilaginous tympanic canal, which is likely to have been pressure-equalised (*e.g.*, Colbert & Ostrom, 1958; Saunders et al., 2000; Tucker, 2017), would have extended between the lateral opening of the tympanic fossa (= stapedial recess) on the braincase and the membranous tympanum near the otic notch. The tympanic canal would have been up to 145 mm long in the holotype and located in the stapedial groove ventrally on the paroccipital process (Figs. 33B, 34A, 38C, 38D). The tympanic fossa is a conically tapered meatus, roughly rhomboidal in parasagittal cross-section and transversely deep (Figs. 37, 38, 55A–55C; see “Prootic” and “Otoccipital” above). The fossa extends ∼37 mm from the lateral surface of the braincase to the auditory fenestrae of the inner ear. At the medial end of the tympanic fossa open the *fenestra vestibularis* (= *f. vestibuli*, oval window) and *fenestra perilymphaticum* (= *f. rotunda, f. cochleae, f. metoticum*, round window) (Figs. 37, 55A–55C).

The osseous labyrinth (inner ear) is divided into the *pars canalicularis* comprising the utricle and semicircular canals and the *pars cochlearis*, comprising the cochlear duct and saccule (Hughes et al., 2015). The osseous labyrinths are well preserved on both sides (Fig. 54); however, the right side is slightly better resolved than the left (Fig. 55). On the right side, the *fenestra vestibularis* (oval window) and *fenestra perilymphaticum* present in the vestibule of the *pars cochlearis* at the medial end of the tympanic fossa (Figs. 37, 55A–55C; see also assessment and description of the *recessus scalae tympani* and the extracapsular saccule of the *fenestra pseudorotunda* under “Otoccipital” above). The membrane of the cochlea would have attached to the *fenestra vestibularis* and received sound induced vibrations from the columella, while the membrane of the extracapsular *f. pseudorotunda* allowed sympathetic vibration balancing of the perilymphatic system *via* the *f*. *perilymphaticum* (Romer, 1955). The *fenestra vestibularis* and *f. pseudorotunda* are divided by a thin *crista interfenestralis* (Fig. 55D)—similar in appearance to that in the therizinosaur *Erlikosaurus andrewsi* (Lautenschlager et al., 2012) and the Late Triassic archosauriform *Triopticus primus* (Stocker et al., 2016). The canal assessed as the pharyngotympanic tube (see further under “Prootic” and “Parabasisphenoid” above), extends ventrally from the junction of the tympanic fossa and the vestibule to emerge in a small fissure at the dorsolateral base of the basal tubera (Figs. 38M, 38O, 40D, 40E, 55A–55C). Notably, the size of the pharyngotympanic tube, its location either within or outside of the osseous neurocranium and its arrangement associated with the pharynx, varies greatly among extant sauropsids (Dufeau & Witmer, 2015; Saunders et al., 2000; Tucker, 2017). In many sauropsids, the pharyngotympanic tube is in the viscera lateral to the braincase. However, in crocodilians and bird-line archosaurs, the pharyngotympanic tube is internalised in the braincase (based on Dufeau & Witmer, 2015). Therefore, our assessment of osseous enclosure of the pharyngotympanic tube in the braincase of *Muttaburrasaurus langdoni*, is supported by its occurrence in other archosaurs, and consistent with the thickened condition of the braincase.

The *fenestra vestibularis* opens to the vestibule of the *pars cochlearis* in the region of the saccule and the ventroposteriorly-adjacent *f. perilymphaticum* enters the vestibule near the dorsal region of the endosseous cochlear duct (ECD) (Fig. 55). Notably, the ECD houses the lagena or the cochlea, with the latter term used primarily for the snail-shaped structure in mammals. Nevertheless, ECD (sensu Witmer et al., 2008) is in general usage for the osseous duct in fossil vertebrates. The anteroventrally directed axis of the ECD is straight, tapering towards its apex (Fig. 55), as generally reported in sauropsids (*e.g.*, Benson et al., 2017; Cerio & Witmer, 2019; Walsh, Luo & Barrett, 2014; Witmer & Ridgely, 2008b). The ECD is circular to ovoid in transverse section in the dorsal two thirds of the duct. On the better-resolved right side, a slight kink is apparent in the ventral third of the ECD, as in *Hypacrosaurus altispinus* (Evans, Ridgely & Witmer, 2009), as well as the ceratopsian *Pachyrhinosaurus lakustai* (Witmer & Ridgely, 2008b). The apical third of the ECD is compressed and oriented parallel to the anterior semicircular canal (ASC). The EDC is measured from the constriction between the *pars canalicularis* and *pars cochlearis*, immediately dorsal to the *fenestra vestibularis*, to the distal-most point of the duct in the basioccipital (based on Walsh et al., 2009), noting that these authors applied the term *fenestra vestibularis* instead of *f. canalicularis* used here and confusingly considered the saccule instead of the utricle as within the *f. vestibularis*). The mean length of the right and left ECDs (Table 2) is 20.4 mm. The ECD is marginally longer than in *Pawpawsaurus campbelli* and *Triceratops* sp., longer than in lambeosaurines and longer than in *Tyrannosaurus rex* (following Sakagami & Kawabe, 2020, and authors within). The region of the utricle is dorsomedial to the saccule (Figs. 55E–55J). The ampulla of the lateral semicircular canal (LSC) would have been located at the anterior end of the canal close to the utricle and the ampulla of the anterior semicircular canal (ASC) would have been located anteriorly, dorsal to the utricle (Figs. 55E–55J). The ampulla of the posterior semicircular canal (PSC) would have been located posteriorly dorsally to the utricle. The ASC and PSC unite at the common crus, which extends dorsally from the utricle. The LSC is approximately parallel to the cerebral axis. The LSC and PSC unite at the secondary common crus, which is also the region of the ampulla of the LSC. The ASC and PSC are oriented 45° to the sagittal plane and the three canals are roughly orthogonal (∼90°) to each other (Figs. 55E–55J). Thus, the paired left and right canal counterparts provided an excitation and inhibition system that detected and relayed rotational motion across the Cartesian planes (see Malinzak, Kay & Hullar, 2012; Michael-Titus, Revest & Shortland, 2010). The lengths of the semicircular canals were measured along their centroids (see “Methods”). The PSCs and LSCs are approximately equal in length and substantially shorter than the ASCs (Figs. 55E–55J; Table 2). The mean of the right and left ASCs is ∼1.5:1 that of the PSCs. The common crus extends to approximately half the height of the ASC, measured from the level of the saccule and PSC (Figs. 55E–55J). The medial margin of the right vestibule contacts the endocranial fossa.

## Results and Discussion

A dorsally inflated muzzle and transversely broad skull traditionally distinguished the genus *Muttaburrasaurus* from all other ornithopods (Bartholomai & Molnar, 1981; Molnar, 1996). Using CT scans of the originally described cranial blocks of the holotype (QMF6140), together with scans of previously undescribed craniodental material of the holotype held within the QM collections from 1963 and newly discovered material from the reestablished holotype quarry, this investigation describes the skull of *Muttaburrasaurus langdoni* in far greater detail than previously possible. This work reveals greater disparity between the skull of *Muttaburrasaurus langdoni* and other ornithopods than previously indicated by the traditional distinction, based mostly on the dorsally inflated muzzle (‘nasal bulla’) and transversely broad skull (Bartholomai & Molnar, 1981; Molnar, 1996). In addition, endocranial data not previously available, give new insight into the behavioural and sensory ecology of the taxon.

### Body mass of the *Muttaburrasaurus langdoni* holotype

To comparatively assess cognition and olfactory ability between *Muttaburrasaurus langdoni* and other dinosaurs, an estimate of body mass is required. Stylopodial equations, based on the minimum diaphyseal circumferences of the humerus and femur, provide a proxy-based, predictive method to estimate the body mass of terrestrial amniotes (Anderson, Hall-Martin & Russell, 1985; Campione & Evans, 2012; Campione et al., 2014; Dempsey et al., 2025). Here, we estimate the body mass of the *Muttaburrasaurus langdoni* holotype using the stylopodial-based formulae for dinosaur quadrupeds by Campione & Evans (2012), denoted by QE, and dinosaur bipeds by Campione et al. (2014), denoted by cQE. The minimum circumferences of the humeral and femoral diaphyses were measured on the holotype. From these variables, the two estimates of body mass were calculated (Table 3).

**Table 3:** Table of body mass, endocast volume and REQ for selected ornithischians.

Taxon	Body mass (kg) and estimation method	Endocast volume (ml)	REQ 60% BEC	Data source
*Amurosaurus riabinini*	4,790.0 ± 25% (stl)	290.0	2.35	Button & Zanno (2023)
*Edmontosaurus* sp.	6,610.0 ± 25% (stl)	300.0	2.49	Button & Zanno (2023)
*Euplocephalus tutus*	2,330.0 ± 25% (stl)	82.0	0.99	Button & Zanno (2023)
*Camptosaurus dispar*	400.0 ± 25% (mod)	46.0	1.47	Button & Zanno (2023)
*Hypacrosaurus altispinus*	3,690.0 ± 25% (stl)	275.9	2.59	Button & Zanno (2023) and Evans, Ridgely & Witmer (2009)
*Iguanodon bernissartensis*	8,270.0 ± 25% (stl)	357.0	2.14	Button & Zanno (2023) and Porter & Witmer (2015)
*Lurdusaurus arenatus*	4,190.0 ± 25% (stl)	167	1.46	Button & Zanno (2023)
*Mantellisaurus atherfieldensis*	1,430.0 ± 25% (stl)	131+	2.00	Button & Zanno (2023)
*Muttaburrasaurus langdoni*	10,085.7 ± 25% (stl, QE)	294.6	na	This study
*Muttaburrasaurus langdoni*	8,854.0 ± 25% (stl, cQE)	294.6	1.64	This study
*Muttaburrasaurus langdoni*	7,916.0 ± 15% (vol)	294.6	1.74	Bishop et al. (2020)
*Proa valdearinnoensis*	3,560.0 ± 25% (stl)	316.0	2.91	Button & Zanno (2023), Knoll et al. (2021); REQ recalculated this study
*Psittacosaurus lujiatunensis*	250.0 ± 25% (stl)	14.3	2.05	Button & Zanno (2023)
*Stegosaurus stenops*	6,950.0 ± 25% (stl)	56.0	0.36	Button & Zanno (2023); REQ recalculated, this study
*Thescelosaurus neglectus*	339.0 ± 25% (stl)	27.25-28.61	0.94	Button & Zanno (2023)
*Triceratops* sp.	13,540 ± 25% (stl)	140	0.62	Button & Zanno (2023)

**Notes.**

Abbreviations BEC brain-to-endocranial cavity correlation index mod volumetric displacement method using scale model cQE stylopodial method of Campione et al. (2014) for bipeds QE stylopodial method of Campione & Evans (2012) for quadrupeds REQ reptile encephalisation quotient stl stylopodial method vol spline-based, whole-body volumetric reconstruction. Endocast volumes reported with olfactory apparatus and brain stem posterior to cranial nerve CN XII removed. Percentage errors for REQ calculated from body mass error

The estimated body mass of the *Muttaburrasaurus langdoni* holotype as a quadruped (QE: 10,085.7 kg ± 25%) is greater than the bipedal estimate (cQE: 8,854.0 kg ± 25%). Notably, as only the distal part of the left humeral diaphysis is preserved on the holotype, the circumference of the missing mid-diaphysis could have been less than the region measured. If the circumference was ∼23mm less than that measured (*i.e.,* ∼92%), the body mass calculated under QE and cQE would be roughly equal. This test shows the sensitivity of circumferential measurements to the calculations of body mass using stylopodial-based formulae.

Using a spline-based, whole-body volumetric reconstruction, Bishop et al. (2020) estimated a lower body mass for the *Muttaburrasaurus langdoni* holotype (7,916.0 kg ± 15%) than recovered using the stylopodial methods. For their reconstruction, Bishop et al. (2020) used photogrammetry of a 1:1, cast polyurethane skeletal mount of the holotype on public display at the QM. However, as much of the thorax and the complete tail of the skeletal mount were sculpted interpretations (based on *Iguanodon*), the volumetrically based mass estimate by Bishop et al. (2020) was somewhat subjective, as these authors had noted. Although a difference of 938 kg is evident between the spline-based volumetric estimate of body mass by Bishop et al. (2020) and the stylopodial-based body mass retrieved under cQE, these estimates overlap within their margins of error (Table 3).

Recent work by Dempsey et al. (2025), building on slightly earlier work of Macaulay et al. (2023), calculated the body masses of 52 non-avialan dinosaurs using a whole-body, volumetric, segment-specific (head, neck, torso, limbs, tail), convex hull expansion approach, based on extrapolations from empirically grounded data retrieved from skeletal and soft tissue anatomy of extant sauropsids. According to Dempsey et al. (2025), convex hull expansion methods provide a robust, holistic means of estimating the body masses of fossil tetrapods. An important outcome from the work of Dempsey et al. (2025), potentially relevant to *Muttaburrasaurus langdoni*, showed that body mass estimates for ornithischian dinosaurs retrieved using allometric and isometric convex hull expansion methods were significantly lower than retrieved under the stylopodial-based methods, QE and cQE (accessed by those authors *via* the MASSTIMATE package v2.0-1 of Campione (2020)). The body masses of the large-bodied, obligatory ornithischian tetrapods, *Stegosaurus* (USNM 4934) and *Triceratops* (NHMUK PV R36730), retrieved using convex hull expansion, were in the range of 47–58% of the masses retrieved under stylopodial calculations (following Dempsey et al., 2025). The body mass of the facultatively bipedal/quadrupedal ornithopod *Iguanodon bernissartensis* found under convex hull expansion was ∼64% that of the mass found under the stylopodial method and the body mass of the small-bodied, ornithopod biped, *Hypsilophodon foxii*, was ∼67% of that found under the stylopodial method (following Dempsey et al., 2025). Interestingly, Dempsey et al. (2025) found that the body masses of non-avialan theropods retrieved using convex hull expansion methods were markedly higher than retrieved under stylopodial-based methods, while the body masses for large-bodied, quadrupedal sauropodomorphs found using both approaches were comparable. These findings led Dempsey et al. (2025) to propose that ornithischians had proportionally more robust limb bones relative to body mass than theropods and likely reflects differences in femoral biomechanics between the two clades (*sensu*, Dempsey et al., 2025). The implications of these findings are that the stylopodial-based calculations of body mass for ornithischians under QE and cQE, potentially give erroneously high estimates. Notably, if the body mass of *Muttaburrasaurus langdoni* was 64% less than the mass found under cQE (*i.e.,* the difference found by Dempsey et al., 2025, between convex hull expansion and stylopodial-based estimates for *Iguanodon bernissartensis*), the mass would be ∼5,667.0 kg. Considering the work of Dempsey et al. (2025), the volumetrically based body mass estimate of the *Muttaburrasaurus langdoni* holotype by Bishop et al. (2020) of 7,916.0 kg ± 15%, could be closer to the ‘correct’ body mass, although still substantially higher than the findings of Dempsey et al. (2025) would suggest. Undoubtedly, estimation of body mass for the *Muttaburrasaurus langdoni* holotype would benefit from a convex hull expansion approach, when the whole-body form can be confidently reconstructed.

To put the body mass of the *Muttaburrasaurus langdoni* holotype in perspective, an upper body mass of 8000 kg was reported for a large male *Loxodonta africana* individual (African bush or savannah elephant) by Bader, Delapré & Houssaye (2023). The body mass estimates of the *Muttaburrasaurus langdoni* holotype found by cQE and Bishop et al. (2020), are comparable to the largest of African elephants. Based on cQE, the upper body mass of *Tyrannosaurus rex* (FMNH PR 2081) was estimated at 8000 kg ± 25% by Campione et al. (2014) and a body mass of 7926 kg was estimated for AMNH 5027 by Dempsey et al. (2025), using the isometric convex hull expansion method. The current body mass estimates of the *Muttaburrasaurus langdoni* holotype are comparable to the largest individuals of *Tyrannosaurus rex*. Whether the *Muttaburrasaurus langdoni* holotype was a somatically mature individual is unknown. As some of the braincase bones lack complete fusion, the holotype could have been a sub-adult and the upper body mass of *Muttaburrasaurus langdoni* could have been higher than that estimated for the holotype.

Notably, bipedal locomotion in *Muttaburrasaurus langdoni* is supported by our analysis of the endosseous labyrinth, discussed in detail below (see “Head posture, auditory capacity, balance and locomotion”). Thus, the body mass of the holotype estimated under cQE for dinosaur bipeds is used in preference to the QE estimate for quadrupeds, while noting that the volumetrically based estimate of the holotype body mass by Bishop et al. (2020) is additionally applicable. For the comparisons of olfactory ratio (OR) and reptile encephalisation quotient (REQ) in this investigation, both cQE and the volumetric based estimates of the holotype body mass are utilised (see further in “Airway anatomy and function” and “Brain size, cognition and locomotion” below).

### A large-bodied ornithopod with a toothed ornithischian premaxilla

The original description of *Muttaburrasaurus langdoni* presumed that the missing premaxillary region of the rostrum would have been edentulous (Bartholomai & Molnar, 1981), as in Iguanodontia. The discovery of dentulous left and right premaxillary rami in *Muttaburrasaurus langdoni* with well-developed teeth is therefore surprising. The left dental ramus is more complete than the right, with the full complement of alveoli preserved (Figs. 10A, 11, 12). However, the identification of the fragmentary, anterior-most portion of the left premaxillary dental ramus (cranial part 13) is currently provisional as the interalveolar widths of 11.5 mm on this anterior fragment and those between the alveoli on the three parts that make up the posterior portion of the dental ramus (cranial parts 6, 7, 12) of 5.5 mm, significantly differ. Apart from this difference, between all of the dentulous premaxillary fragments (cranial parts 6, 7, 12, 13, 14), the morphology and size of the roots of the functional teeth and the sizes of the germ tooth crowns are congruent. As a part of the holotype, the only other potential location for cranial part 13 was at the posterior end of the right premaxilla, noting that the germ teeth are always medial to the functional teeth. However, the posterior part of the right premaxillary dental ramus is unequivocally represented by cranial part 14 (Figs. 10B, 15), although differences are apparent between the neurovascular features on the posterior left and right sides (Fig. 14). As a part of the holotype and without anatomical overlap, cranial part 13 can only locate at the anterior end of the left dental ramus (Figs. 11, 12). If cranial part 13 were to pertain to a second ornithischian individual deposited along with the *Muttaburrasaurus langdoni* holotype, the taxonomy of all materials currently assigned to *Muttaburrasaurus langdoni* would be in question. Accepting this remote possibility, it seems highly unlikely that two dinosaur carcasses would have been buried together in the same storm deposit on the Eromanga Sea floor, interpreted here as a scour, after floating out individually into the seaway. For this reason, we presently accept that all the ornithischian materials recovered at the *Muttaburrasaurus langdoni* holotype locality (QML1794) pertain to a single individual.

Accepting the placement of cranial part 13 as the anterior part of the left premaxillary dental ramus of the *Muttaburrasaurus langdoni* holotype, five well-developed premaxillary teeth are present, as in the early diverging ornithopod *Hypsilophodon foxii* (Galton, 1974) and the early diverging neornithischians, *Agilisaurus louderbacki* and *Changchunsaurus parvus* (Jin et al., 2010), *Haya griva* Makovicky et al., 2011, *Orodromeus makelai* (Scheetz, 1999) and *Zephyrosaurus schaffi* Sues, 1980. Six premaxillary teeth occur in the early diverging neornithischians, *Thescelosaurus neglectus* Boyd, 2014 and *Jeholosaurus shangyuanensis* Xu, Wang & You, 2000 (Barrett & Han, 2009; Hu et al., 2024). The dentulous premaxilla of *Muttaburrasaurus langdoni* is the plesiomorphic condition for an ornithischian. Although the anterior-most end of the premaxillary dental ramus in *Muttaburrasaurus langdoni* is presently unknown, based on the preserved left side, the complete premaxillary dental ramus was lengthy; estimated to have been ∼30% of the total anteroposterior cranial length (the posterior-most end taken at the posterior tip of the retroarticular process and adjusted for posterior displacement of the left quadrate and mandible by -20 mm). The comparative length of the premaxillary ramus in *Thescelosaurus neglectus* is ∼25%, noting that six premaxillary alveoli are present (based on Boyd, 2014, fig. 2). Although functional premaxillary crowns have not been preserved in the holotype premaxillae of *Muttaburrasaurus langdoni*, the well-preserved germ crowns indicate a conical, caniniform shape with elliptical cross-sections grading to sub-circular sectioned roots (Figs. 12, 13D–13E, 15G–15I). The crown bases of the premaxillary teeth are only slightly expanded from the roots and apart from a distal carina, the crown surfaces are smooth without grooves or ridges. The premaxillary tooth crown morphology of *Muttaburrasaurus langdoni* more closely resembles those of the heterodontosaurids, *Heterodontosaurus tucki* and *Abrictosaurus consors* (Sereno, 2012), than those of the early diverging neornithischians and early diverging ornithopods, whose crowns typically expand mesiodistally from the roots, are labiolingually foliate and ornamented with grooves and/or denticles (see Andrzejewski, Winkler & Jacobs, 2019; Barta & Norell, 2021; Boyd, 2014; Galton, 1974; Jin et al., 2010; Sereno, 1991; Zanno et al., 2023). Two alveoli were reported in the elasmarian *Talenkauen santacrusensis* (Rozadilla, Agnolín & Novas, 2019), although exact details of these alveoli are presently lacking that would allow comparisons with *Muttaburrasaurus langdoni*. Premaxillary teeth are characteristically absent in Dryomorpha (= Ankylopollexia and Dryosauridae; Sereno, 1986).

In Dryomorpha, the premaxilla characteristically forms an edentulous bill and the oral margin is laterally everted, a exemplified in the hadrosaurids (Horner, Weishampel & Forster, 2004; Norman, 2004). Even in the rhabdodontomorph *Iani smithi* Zanno et al., 2023 and the early diverging iguanodontian, *Tenontosaurus dossi* Winkler, Murry & Jacobs, 1997, being two early diverging iguanodontians retaining alveoli, the premaxillary oral margins are laterally everted, as in other iguanodontians. Unlike the typical iguanodontian condition, the oral margin on the premaxilla of *Muttaburrasaurus langdoni* is only slightly everted, as in *Hypsilophodon foxii* and *Thescelosaurus neglectus* (Boyd, 2014; Galton, 1974). However, it is notable that the anatomy of the anterior-most region of the premaxilla in *Muttaburrasaurus langdoni* is presently unknown.

**Figure 56:**
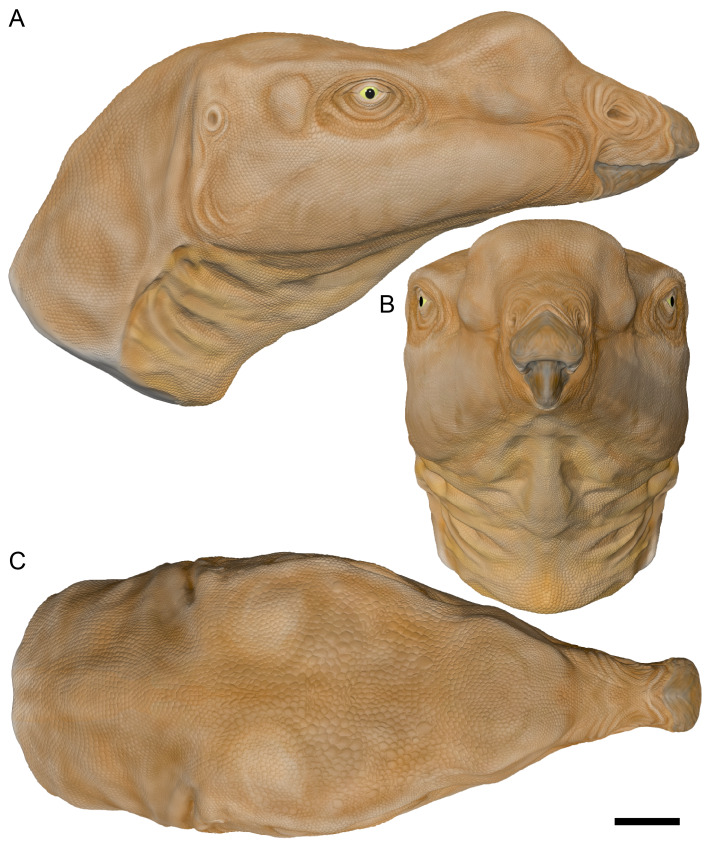
Life reconstruction of the *Muttaburrasaurus langdoni* (QMF6140) head. (A –C) Reconstruction in (A) right lateral, (B) anterior and (C) dorsal views. Scale bar equals 10 cm.

In conclusion, among ornithischians, *Muttaburrasaurus langdoni* retains the plesiomorphic dentulous condition of the premaxilla, present in early diverging non-ornithopod neornithischians and early diverging ornithopods (Andrzejewski, Winkler & Jacobs, 2019; Barta & Norell, 2021; Boyd, 2014; Galton, 1974; Hu et al., 2024). With an estimated body mass in the order of 7,900–8,850 kg, *Muttaburrasaurus langdoni* was the largest ornithischian to retain five premaxillary alveoli and well-developed premaxillary teeth. The well-developed premaxillary dentition of *Muttaburrasaurus langdoni* suggests a large-bodied ornithopod with a feeding strategy differing from Dryomorpha where the premaxilla forms an edentulous everted bill. It seems unlikely that the lineage of *Muttaburrasaurus langdoni* would have re-acquired premaxillary dentition from an edentulous iguanodontian progenitor, suggesting that divergence of the *Muttaburrasaurus langdoni* lineage occurred independently of Dryomorpha and potentially prior to Iguanodontia. From the revised osteology, a life restoration of the *Muttaburrasaurus langdoni* head is attempted (Fig. 56) (see details in “Methods”), from which the visual fields of *Muttaburrasaurus langdoni* are assessed (see “visual fields of *Muttaburrasaurus langdoni*” below).

### Novel bones and lambeosaurine convergence

The original description of *Muttaburrasaurus langdoni* considered that the inflated muzzle was formed from the nasal bones (Bartholomai & Molnar, 1981), although Molnar (1996), during his revision of the *Muttaburrasaurus langdoni* holotype and the description of the second skull he assigned to *Muttaburrasaurus* sp. (QMF14921; known as the ‘Dunluce’ skull), expressed uncertainty about the osteological make-up of the inflated muzzle. Notably, early cranial descriptions of lambeosaurines identified their crests as formed primarily of the paired nasals (Brown, 1914; Brown, 1916), while later studies concluded that the crests were predominately formed of complexly extended processes of the premaxilla (Lambe, 1920; Ostrom, 1961; Ostrom, 1962). In a corollary to the amended anatomical understanding of the anatomical components of the lambeosaurine crests, CT data now reveal that the dorsally inflated muzzle of *Muttaburrasaurus langdoni* is a bone complex consisting of extensively paired posterodorsal and posteroventral processes of the premaxilla and novel, complexly paired ossifications, termed here, prenasals (Figs. 5–8, 16–19). For this reason, we avoid the term ‘nasal bulla’ for *Muttaburrasaurus*. The nasals form the posterior wall and portions of the lateral walls of the dorsally inflated muzzle. The sutural margins identified in the CT imagery, clearly indicate that the prenasals and the nasals are separate bones (see Fig. 18). Sutural definition is further apparent between the posterodorsal process of the left premaxilla and the dorsolateral margin of left prenasal ossification. However, the anterior-most parts of the left prenasal ossification and left premaxilla have been lost and as a result, their true anatomical association is unknown. The prenasal could be a third, complex, posterodorsal process of the premaxilla or, alternatively, a neomorphic bone separate from the premaxilla. However, we consider it more likely that the prenasal is a novel neomorphic bone, as the occurrence of three posteriorly extending processes on the maxilla have not been reported in any dinosaur or amniote, as far as we are aware (see also under “Olfaction” below). The posterodorsal and posteroventral processes of the premaxilla form most of the lateral walls of the muzzle and the prenasals form the dorsal roof of the muzzle. The anterior-most part of the dorsal roof of the muzzle is missing, nevertheless, the preserved internal structures of the muzzle formed by the septa and processes of the prenasals suggest that the latter ossifications also formed the anterodorsal region of the inflated muzzle. The osteological composition of the *Muttaburrasaurus langdoni* muzzle, together with that of *M*. sp., (QMF14921), although not specifically studied in this work, is unique, while sharing some of its morphological makeup with lambeosaurines. The shared features of the cranium between *Muttaburrasaurus langdoni* and the lambeosaurines include a pseudonaris, formed by the expanded posterior processes of the premaxilla (*i.e.,* the posterodorsal and posteroventral processes) that exclude the nasals from the narial opening and lateral displacement of the nasals (see Evans, 2010; Evans & Reisz, 2007; Ostrom, 1961; Prieto-Márquez & Wagner, 2013). These shared features of the muzzle between *Muttaburrasaurus langdoni* and lambeosaurines are convergent, while the prenasal, whether it constitutes a further, complex process of the premaxilla, or a neomorphic element, is unique to *M. langdoni*.

### Airway anatomy and function in *Muttaburrasaurus langdoni*

**Figure 57:**
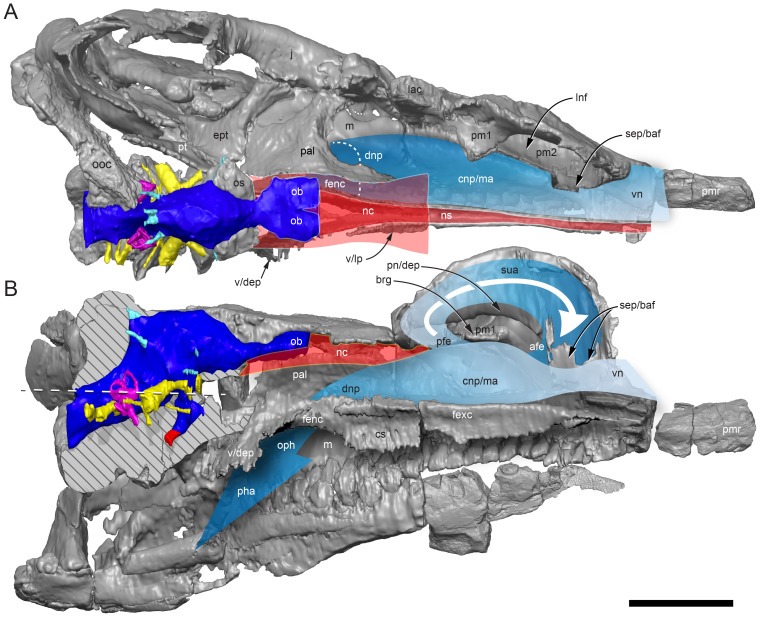
Cranial cross-sections, endocranium and nasopharyngeal airway reconstruction for *Muttaburrasaurus langdoni* (QMF6140). (A) Dorsal cross-section, exposing endocranium and passage of inspired air in main airway (blue shaded area) on the left side in dorsal view, with cranial roof and dorsal neurocrania removed (colour coding of endocranium see B). (B) Sagittal cross-section exposing endocranium and hypothesised passage of inspired air viewed from the right side with the right cheek region and median septa of prenasals removed (brain endocast, dark blue; vestibular canals, magenta; cranial nerves, yellow; cranial veins, light blue; cerebral carotid artery, red; sectioned neurocranium shown cross-hatched). Reconstruction of the cartilaginous nasal septum (red shaded area with “ns”) is indicated in A, dorsal to the vomers and nasal cartilage (red shaded area with “nc”) is indicated dorsal to the palatines in A and B. Dashed line in A indicates border of the *fenestra endochoanalis*. Large arrow in B indicates passage of inspired air in the inferior airway. Small white arrow in B suggests passage of air through fenestra in and out of the left superior airway and hypothesised region of the olfactory meatus. Dashed line in B indicates plane of the horizontal semicircular canal. Abbreviations: afe, anterior fenestra; baf, baffle; brg, transverse bridge (= posteromedial process on posterodorsal process of premaxilla and horizontal septum of prenasal); cho, choana; cnp, *cavum nasi proprium*; cs, choanal septum; dep, descending process; dnp, *ductus nasopharyngeus*; ept, ectopterygoid; fenc, *fenestra endochoanalis*; fexc, *fenestra exochoanalis*; j, jugal; lac, lacrimal; lnf, lateral nasal fossa; lp, lateral process; m, maxilla; ma, main airway; n, nasal; nc, nasal cartilage; ns, nasal septum; ob, olfactory bulb; ooc, otoccipital; oph, oropharynx; os, orbitosphenoid; pal, palatine; pfe, posterior fenestra; pha, pharynx; pm1, posterodorsal process of premaxilla; pm2, posteroventral process of premaxilla; pmr, premaxillary ramus; pn, prenasal; ppn, pendulous process of prenasal; ps, parasphenoid; psn, pseudonaris; pt, pterygoid; sep, septum; sua, superior airway; v, vomer; vn, *vestibulum nasi*. Scale bar equals 10 cm.

The main airway in non-avialan dinosaurs, as in extant amniotes, would have been divided by a midline cartilaginous nasal septum (= mesethmoid) that attached ventrally on the vomera and extended dorsally to the ventral surface of the nasals, with each side further divided into three interconnecting airway zones—the *vestibulum nasi*, the *cavum nasi proprium* and the *ductus nasopharyngeus* (based on: Ali et al., 2008; Bellairs, 1949; Bourke et al., 2014; Jarvik, 1942; Van Valkenburgh, Smith & Craven, 2014). The *vestibulum nasi* is the relatively short meatus leading internally from the fleshy nostril. The *cavum nasi proprium* is the extensive main airway meatus, within which project the nasal turbinals of the nasal capsule for olfaction, respiratory humidification, heat exchange and toxicant removal, and the *ductus nasopharyngeus* (= nasopharynx) connects the *cavum nasi proprium* to the pharynx, *via* the oropharynx (based on: Bourke et al., 2014; Harkema, Fau & Wagner, 2006; Van Valkenburgh, Smith & Craven, 2014). The *fenestra exochoanalis* is the osseous choanal slot formed by the vomer and the maxilla that would have supported the fleshy soft palate, while the region of the *fenestra endochoanalis* between the vomer and the maxilla, posterior to the *fenestra exochoanalis*, represents the fleshy opening between the *ductus nasopharyngeus* and the oropharynx (following Bellairs, 1949; Bourke et al., 2014). The three airway zones are suggested in the skull of *Muttaburrasaurus langdoni* (Fig. 57). However, *Muttaburrasaurus langdoni* differs from all other dinosaurs in the division of the *cavum nasi proprium* into inferior (main/ventral) and superior (dorsal) airway meatuses. The paired inferior meatus constitutes the main airway while the paired superior meatuses form a loop off the main airway into the dorsally hypertrophied region of the muzzle (Fig. 57). The hypothesised function of the sagittally divided superior airway will be discussed below.

The inferior and superior airways of the *cavum nasi proprium* of *Muttaburrasaurus langdoni* are divided into left and right sides. The main airway is likely to have been divided by a cartilaginous nasal septum that extended from the vomera to the ventral surface of the osseous prenasal septa (Figs. 19A–19E, 57). The superior airway is divided by abutting sagittal septa of the prenasals (Figs. 8, 16, 19, 57). Each side of the superior airway is partially separated from the inferior airway by three septal features (Figs. 8, 57): (1) an anteroventral septal lamina of the prenasal connecting the anteroventral process on the posterodorsal process of the premaxilla; (2) a transversely oriented horizontal septum of the prenasal that connects the posteromedial processes on the posterodorsal process of the premaxilla, thus forming a bridge; and (3) a ventral ledge anteriorly on the toroidal channel of the nasal body. These three septa effectively form two fenestral openings between the inferior and superior airways (Fig. 57). From the lateral processes and choanal septum on the vomera of the holotype, the *fenestra exochoanalis* and the region of the soft palate are identified (Figs. 31M–31Q, 57). The location of the *fenestra endochoanalis* is identified by the smoothly rounded surfaces laterally on the vomerine shaft, between the region of the choanal septum and lateral processes, anteriorly, and the descending processes, posteriorly (Fig. 57). The fleshy posterior wall of the *ductus nasopharyngeus* in *Muttaburrasaurus langdoni* would have been supported by the palatine, as in most dinosaurs (*e.g.*, Styracosterna (Heaton, 1972; Norman, 1980); *Tyrannosaurus rex* (Brochu, 2003), although generally not stated in most osteologically focused descriptions. In all ornithopods and possibly all ornithischians, except for *Muttaburrasaurus langdoni*, the posterior wall of the fleshy *ductus nasopharyngeus* would have been partly supported by the anterodorsally projecting palatine processes of the pterygoids. These processes are absent in *Muttaburrasaurus langdoni*. In ornithischians, support for the fleshy posterior wall of the *oropharynx* was likely provided by the anterodorsally projecting palatine processes of the pterygoids. However, in *Muttaburrasaurus langdoni*, this function was potentially provided by the laterally flared descending processes on the vomera that project posteroventrally to contact the pterygoids (Figs. 23A–23D, 31M–31Q, 57). It is apparent that the well-developed descending processes on the vomera of *Muttaburrasaurus langdoni* performed the function of the palatine processes of the pterygoids in other ornithischians, while noting, as a caveat, this palatal region is poorly described in many ornithischians.

*Olfaction—* The *cavum nasi proprium* of extant amniotes house the turbinals (turbinates, conchae) for olfaction and moisture conservation in all amniotes and respiratory heat exchange in endothermic amniotes (mammals and birds) (Martinez et al., 2024; Tada & Tsuihiji, 2021; Van Valkenburgh, Smith & Craven, 2014). The presence of turbinals for respiratory heat exchange is still debated in non-avialan dinosaurs (Bourke et al., 2014; Bourke & Witmer, 2016; Tada & Tsuihiji, 2021). The turbinals are highly complex, finely structured, paper-thin bones and/or cartilage (Martinez et al., 2024). However, in fossil taxa they are typically destroyed postmortem (Bourke et al., 2014; Hillenius, 1994; Martinez et al., 2024). In extant amniotes such as large-bodied mammalian ungulates and palaeognathous birds, turbinals are supported by osseous turbinal attachment ridges on the walls of the *cavum nasi proprium* (Bourke et al., 2014; Fonseca et al., 2024a; Hillenius, 1994; Martinez et al., 2024; Ruf et al., 2014; Tada & Tsuihiji, 2021; Taniguchi & Taniguchi, 2014; Van Valkenburgh, Smith & Craven, 2014). Notably, ridges of similar appearance to those in extant amniotes identified in the *cavum nasi proprium* of fossil amniote skulls can signal the original existence of turbinals, including in non-avialan dinosaurs (Bourke et al., 2014; Bourke, Porter & Witmer, 2018; Fonseca et al., 2024a; Geist, 2000; Hillenius, 1994; Martinez et al., 2024; Owerkowicz, 2015; Tada & Tsuihiji, 2021; Van Valkenburgh, Smith & Craven, 2014; Witmer & Ridgely, 2010). Nevertheless, turbinal attachment ridges are rarely identified in extinct diapsids but have been positively identified in pachycephalosaurs (Bourke et al., 2014).

Turbinals are formed from laminae in the centres of chondrification (Martinez et al., 2024; and authors within). Turbinals for olfaction in extant amniotes (ethmoturbinals) typically develop in diverticula at the posterodorsal end of the *cavum nasi proprium*, posterior to the *ductus nasopharyngeus* in amphibians, lepidosaurs, crocodilians, testudines and mammals (Bellairs, 1949; Harkema, Fau & Wagner, 2006; Hillenius, 1994; Martinez et al., 2024; Van Valkenburgh, Smith & Craven, 2014) and dinosaurs, including birds (Bourke & Witmer, 2016; Martinez et al., 2024; Tada & Tsuihiji, 2021). In contrast to olfaction, the turbinals for respiration are developed in the main respiratory airflow of the *cavum nasi proprium* (Martinez et al., 2024). In extant amniotes, the olfactory diverticulum forms a cul-de-sac that allows inspired air to reside with enough time for efficient odour detection across the olfactory epithelium (Bourke et al., 2014; Van Valkenburgh, Smith & Craven, 2014; Witmer & Ridgely, 2009). The ethmoidal centre of olfaction has been identified in non-avialan dinosaurs such as the non-avialan theropods, *Tyrannosaurus rex* and *Majungasaurus crenatissimus* (Sampson & Witmer, 2007; Witmer & Ridgely, 2008a; Witmer & Ridgely, 2010), the ankylosaurs, *Euoplocephalus*, *Panoplocephalus* and *Kunbarrasaurus iversi* (Leahey et al., 2015; Miyashita et al., 2011; Witmer & Ridgely, 2008a) and the pachycephalosaurs, *Stegocerus validum* and *Sphaerotholus* spp. (Bourke et al., 2014). In these dinosaurs, the olfactory diverticula were in the region of the sphenethmoid lateral to, or anterolateral to the olfactory bulbs. In non-avialan dinosaurs in general, the olfactory diverticula would have been located close to the olfactory bulbs (*e.g.*, Bourke et al., 2014; Evans, 2006; Evans, Ridgely & Witmer, 2009; Miyashita et al., 2011; Witmer & Ridgely, 2008a; Witmer & Ridgely, 2010), as in extant amniotes, and the diverticula would have been separated from the olfactory bulbs by the transverse nasal cartilage (= sphenethmoid/dorsal plate/cribriform plate) continuous with the midline nasal septum (Bourke et al., 2014; Ali et al., 2008; Martinez et al., 2024; Witmer & Ridgely, 2010). Crocodilians, typified by *Alligator mississippiensis* (Hansen, 2007), differ from dinosaurs, as the olfactory epithelia are developed over much of the *cavum nasi proprium*. In non-avialan dinosaurs, multiple rami of the olfactory nerve would have coursed from the olfactory bulbs through fenestra in the nasal cartilage to the sensory epithelium (Ali et al., 2008; Bourke et al., 2014; Evans, Ridgely & Witmer, 2009; Martinez et al., 2024; Witmer & Ridgely, 2010). Support for olfaction in the posterior-most region of the *cavum nasi proprium* in dinosaurs, where the cartilaginous and thin osseous structures typically fail to preserve, has been proposed in a few non-avialan dinosaurs by the identification of mineralised olfactory turbinals. These have been identified in ankylosaurs (Miyashita et al., 2011), pachycephalosaurs (Bourke et al., 2014) and *Tyrannosaurus rex* (Witmer & Ridgely, 2010). Modelling of airflow fluid dynamics through the *cavum nasi proprium* of pachycephalosaurs using extant diapsids as phylogenetically constrained osteological correlates (Bourke et al., 2014), and authors within), provided quantifiable support for olfaction in the blind olfactory diverticula in the group and by extension, was considered as the likely centre of olfaction in other dinosaurs, given the highly conservative nature of this region across vertebrate evolution (Evans, 2006; Maryańska, 1977; Tada & Tsuihiji, 2021; Witmer & Ridgely, 2008a).

Given the expectation of the posteriorly positioned olfactory diverticulum in the *cavum nasi proprium* of ornithischians, no evidence of osseous structures consistent with olfaction, such as turbinal support ridges, are seen in this region of the *Muttaburrasaurus langdoni* holotype skull, although these structures could have been cartilaginous and were not preserved. In *Muttaburrasaurus langdoni*, the transverse nasal cartilage (sphenethmoid) would have been located dorsal to the *ductus nasopharyngeus* anterior to the olfactory bulbs and between the wing-like anterior processes of the palatines, which likely supported the sphenethmoid (Fig. 57). If the olfactory diverticula formed pouches within the nasal cartilage between the palatines, they would have been highly restricted in that location. If the olfactory diverticulum had been formed dorsolateral to the palatine and medial to the lacrimal, we fail to identify mineralised structures in the CT imagery that would hint at this location. In amniotes, transverse laminae divide and protect the ethmoturbinal diverticular from the main airflow (Hillenius, 1992; Hillenius, 1994).

Importantly, the paired superior airways in *Muttaburrasaurus langdoni* separated from the main airway by horizontal septa (Fig. 57B) are consistent with diverticula for olfaction removed from the main airflow. The horizontal septa are formed by the prenasals and the posterodorsal processes of the premaxilla (Fig. 57B). Although turbinals are typically not preserved in the airways of fossil skulls, turbinal support ridges on the roof and walls of the airways have been used to indicate their original presence, as well as the original centre of ethmoturbinal chondrification (Hillenius, 1994; Martinez et al., 2024; Ruf et al., 2014; Sigurdsen, 2006; Tada & Tsuihiji, 2021). The elongate descending processes of the prenasals in the superior airway of *Muttaburrasaurus langdoni* (Figs. 8, 19, 57B) are consistent with the location of the turbanal support ridges. If these structures are turbinal support ridges, as we suggest, their large size would be unique among amniotes and the olfactory epithelia would have been extensive.

Tracking the potential airflow through the *cavum nasi proprium* of *Muttaburrasaurus langdoni* suggests that inspired air was diverted from the main airway into the superior airway meatuses through the posterior fenestra on each side (Fig. 57B). The diverted inspired air potentially exited the superior airway through the anterior fenestrae back to the main inspired airflow. Thus, a circular airflow path is hypothesised through the superior airway meatus. It seems unlikely that inspired air would have passed directly into the superior airway through the anterior fenestrae, as transverse septa of the prenasals and the posterodorsal processes of the premaxillae formed baffles dorsal to the incurrent airflow from the *vestibulum nasi* (Fig. 57B). The toroidal shaped fossae formed by the nasals in the superior airway and the form of the curved turbinal support ridges, which broaden anteriorly and slope anteroventrally towards the anterior fenestrae, further suggest the posterior to anterior direction of air airflow in the superior airways (Figs. 19, 57B). The posterior to anterior movement of inspired air through the superior airway would have been slowed thereby allowing residence time for olfactory reception. Rami of the olfactory nerves could have coursed through small foramina that penetrate the toroidal wall of the nasals (Fig. 19). However, as these foramina appear to project into the thickened bodies of the nasals, rather than towards the region of the olfactory lobes, they could be an artifact of preservation. Alternatively, rami of the olfactory nerves could have coursed from the olfactory bulbs to the superior airway through the nasal cartilage and the posterior prenasal fenestrae.

Notably, the tubular architecture of the lambeosaurine crests (Evans, Ridgely & Witmer, 2009; Weishampel, 1981) differ from the chambered structure of the superior airways in *Muttaburrasaurus langdoni* and the function of the lambeosaurine crest has not been considered for olfaction but vocalisation and possibly display (Evans, Ridgely & Witmer, 2009; Weishampel, 1997). Ethmoturbinal diverticuli and turbinal support ridges, comparable to the size and location of those hypothesised in *Muttaburrasaurus langdoni*, have not been identified in any other dinosaur. In their proposed function for olfaction, the prenasal ossifications of *Muttaburrasaurus langdoni* are conducive to ethmoturbinal chondrification centres (Martinez et al., 2024). However, the homology of the prenasal, if separate from the premaxilla, requires further investigation”. In summary, heightened olfactory reception in *Muttaburrasaurus langdoni* is suggested by the enlarged, paired superior airways and the identification of the descending processes on prenasals as turbinal support ridges. In addition to these features, heightened olfactory acuity is further supported by highly enlarged olfactory bulbs, as discussed in the section that follows.

*Olfactory ratio*—Olfactory bulb size has long been used as a direct indicator of olfactory capabilities in vertebrates (Edinger & Rand, 1908) because its primary role as a conduit for olfactory information in vertebrates reduces its integration with other parts of the brain (Carlisle et al., 2017). Olfactory ratio (OR), calculated by division of the greatest diameter of the olfactory bulbs by the greatest diameter of the cerebrum (both measures regardless of orientation) has been used as an indicator of olfactory acuity in mammals, birds and non-avialan dinosaurs (Bang & Cobb, 1968; Button & Zanno, 2023; Cobb, 1959; Evans, Ridgely & Witmer, 2009; Muller, 2022; Sakagami & Kawabe, 2020; Witmer & Ridgely, 2009; Witmer et al., 2008; Zelenitsky, Therrien & Kobayashi, 2009; Zelenitsky et al., 2011). Higher OR indicates greater olfactory acuity—being the ability to discriminate faint chemical odours (Zelenitsky, Therrien & Kobayashi, 2009, and authors there in). In addition to OR, a strong trend towards increased OR with increased body mass has been shown in non-avialan dinosaurs (Button & Zanno, 2023; Muller, 2022; Sakagami & Kawabe, 2020; Zelenitsky, Therrien & Kobayashi, 2009; Zelenitsky et al., 2011). Log normalised plots of OR against body mass suggest that some theropod, sauropod and ornithischian taxa have higher OR values than their lines of regression predict and some taxa fall below the predicted values (Button & Zanno, 2023; Muller, 2022; Sakagami & Kawabe, 2020; Zelenitsky, Therrien & Kobayashi, 2009; Zelenitsky et al., 2011). The reasons why relative olfactory size increased with increased body size in non-avialan dinosaurs is not entirely clear. This trend is not apparent in birds (Muller, 2022). However, Muller (2022) suggests that a greater reliance on olfaction could have occurred in the ecology of large dinosaurs with decreased reliance on binocular vision.

**Figure 58:**
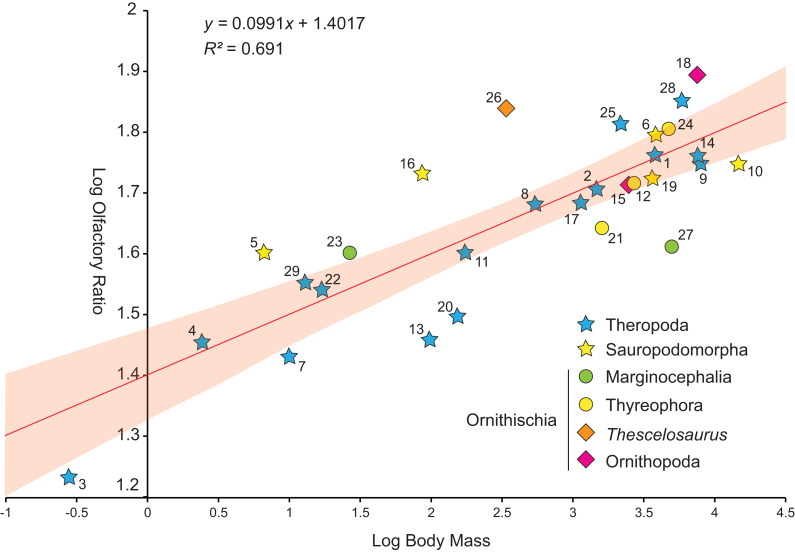
Log normalised plot of olfactory ratio against body mass for selected dinosaurs. Red line indicates the simple (least-squares) linear regression for the minimised sum of the squared residuals (residuals, see Table 5). Shaded area indicates high and low confidence intervals (95% CI). Taxon abbreviations: 1, *Acrocanthosaurus atokensis*; 2, *Allosaurus fragilis*; 3, *Archaeopteryx lithographica*; 4, *Bambiraptor feinbergi*; 5, *Buriolestes schultzi*; 6, *Camarasaurus lentus*; 7, *Carcharodontosaurus saharicus*; 8, *Ceratosaurus nasicornis*; 9, *Dilong paradoxus*; 10, *Diplodocus longus*; 11, *Erlikosaurus andrewsi*; 12, *Euplocephalus* sp.; 13, *Garudimimus brevipes*; 14, *Giganotosaurus carolinii*; 15, *Hypacrosaurus altispinus*; 16, *Macrocollum itaquii*; 17, *Majungasaurus crenatissimus*; 18, *Muttaburrasaurus langdoni*; 19, *Nigersaurus taqueti*; 20, *Ornithomimus edmontonicus*; 21, *Panoplosaurus mirus*; 22, *Saurornitholestes langstoni*; 23, *Stegoceras validum*; 24, *Stegosaurus stenops*; 25, *Tarbosaurus bataar*; 26, *Thescelosaurus neglectus*; 27, *Triceratops* sp.; 28, *Tyrannosaurus rex*; 29, *Velociraptor mongoliensis*. For data sources, see Table 5.

Here, we undertake a comparative analysis of OR between *Muttaburrasaurus langdoni* and selected non-avialan dinosaurs. Uniquely in *Muttaburrasaurus langdoni*, the olfactory bulbs exceed the transverse width of the cerebrum (Fig. 54; Table 2). An OR of 78.4% is calculated for the holotype, suggesting a significant level of olfactory acuity, and potentially the highest among dinosaurs presently reported. The log normalised plot of olfactory ratio against body mass for the holotype (using the volumetric-based estimate by Bishop et al., 2020) and selected dinosaurs (Fig. 58; Table 4), returned a high Pearson Correlation Coefficient (*R*: +0.8313), confirming a strong linear correlation between olfactory ratio and body mass in dinosaurs (Zelenitsky, Therrien & Kobayashi, 2009; Zelenitsky et al., 2011). An *R*^2^ value of 0.691 (*Standard Error*: 0.085 = average distance of the log olfactory ratio data points from the line of regression; *Significance F*: 2.33 ×10^8^, indicating *P* ≤ 0.05) indicates that 69% of the variance in the olfactory ratio is accounted for by body mass (Fig. 58). *Muttaburrasaurus langdoni* lies well above the upper 95% confidence interval of the predicted olfactory ratio (*Residual*: +0.106), returning the highest olfactory ratio among the dinosaurs assessed (Fig. 58), followed by *Tyrannosaurus rex* (OR 71.0%; *Residual*: +0.076) (a theropod previously assessed as having heightened olfactory capacity; Witmer & Ridgely, 2009; Zelenitsky, Therrien & Kobayashi, 2009) and marginally above the early diverging neornithischian *Thescelosaurus neglectus* (OR 69.1%; *Residual*: +0.187), with this latter taxon having the highest olfactory ratio to body mass in dinosaurs (see also Button & Zanno, 2023). It is notable that the analysis of relative OR using the higher body mass estimate for the holotype, retrieved using cQE (*R*: +0.8313; *R*^2:^; 0.6911; *F*: 2.17 × 10^8^), gives a similarly significant residual (+0.101). If the body mass of the holotype were lower than the volumetric- and stylopodial-based estimates used, as the work by Dempsey et al. (2025) suggests (see under “Body mass of the *Muttaburrasaurus langdoni* holotype” above), the relative OR for *Muttaburrasaurus langdoni* would be higher than shown and comparable to *Thescelosaurus neglectus*.

**Table 4:** Table of log olfactory ratio and body mass for selected non-avialan dinosaurs. Numbers and residuals in table correspond to taxa in Fig. 58.

Taxon	Log (Body Mass)	Log (Olfactory Ratio)	Residual	Number (Fig. 58)	Data source
*Acrocanthosaurus atokensis*	3.5773	1.7642	0.008011	1	Zelenitsky, Therrien & Kobayashi (2009)
*Allosaurus fragilis*	3.1670	1.7059	−0.00965	2	Zelenitsky, Therrien & Kobayashi (2009)
*Archaeopteryx lithographica*	−0.5528	1.2330	−0.1139	3	Zelenitsky, Therrien & Kobayashi (2009)
*Bambiraptor feinbergi*	0.3874	1.4548	0.01478	4	Zelenitsky, Therrien & Kobayashi (2009)
*Buriolestes schultzi*	0.8228	1.6021	0.118846	5	Müller (2022)
*Camarasaurus lentus*	3.5842	1.7959	0.039026	6	Müller (2022)
*Carcharodontosaurus saharicus*	3.8980	1.7482	−0.03976	7	Zelenitsky, Therrien & Kobayashi (2009)
*Ceratosaurus nasicornis*	2.7316	1.6821	0.009782	8	Zelenitsky, Therrien & Kobayashi (2009)
*Dilong paradoxus*	1.0000	1.4314	−0.06941	9	Zelenitsky, Therrien & Kobayashi (2009)
*Diplodocus longus*	4.1706	1.7482	−0.06678	10	Müller (2022)
*Erlikosaurus andrewsi*	2.2405	1.6021	−0.02164	11	Lauters et al. (2012)
*Euoplocephalus * sp*.*	3.4275	1.7160	−0.02532	12	Sakagami & Kawabe (2020)
*Garudimimus breviped*	1.9905	1.4594	−0.13953	13	Zelenitsky, Therrien & Kobayashi (2009)
*Giganotosaurus carolinii*	3.8785	1.7612	−0.02484	14	Zelenitsky, Therrien & Kobayashi (2009)
*Hypacrosaurus altispinus*	3.3941	1.7137	−0.02436	15	Sakagami & Kawabe (2020)
*Macrocollum itaquii*	1.9390	1.7324	0.138575	16	Müller (2022)
*Majungasaurus crenatissimus*	3.0531	1.6839	−0.02027	17	Zelenitsky, Therrien & Kobayashi (2009)
*Muttaburrasaurus langdoni*	3.8985	1.8943	0.106329	18	This study
*Nigersaurus taqueti*	3.5611	1.7243	−0.03029	19	Müller (2022)
*Ornithomimus edmontonicus*	2.1847	1.4969	−0.12124	20	Zelenitsky, Therrien & Kobayashi (2009)
*Panoplosaurus mirus*	3.2041	1.6435	−0.07574	21	Hughes & Finarelli (2019)
*Saurornitholestes langstoni*	1.2304	1.5416	0.017972	22	Zelenitsky, Therrien & Kobayashi (2009)
*Stegoceras validum*	1.4265	1.6021	0.059024	23	Sakagami & Kawabe (2020)
*Stegosaurus stenops*	3.6742	1.8062	0.040409	24	Sakagami & Kawabe (2020)
*Tarbosaurus bataar*	3.3355	1.8136	0.081378	25	Zelenitsky, Therrien & Kobayashi (2009)
*Thescelosaurus neglectus*	2.5296	1.8394	0.187078	26	Button & Zanno (2023)
*Triceratops* sp.	3.6958	1.6126	−0.15534	27	Zelenitsky, Therrien & Kobayashi (2009)
*Tyrannosaurus rex*	3.7675	1.8513	0.076239	28	Zelenitsky, Therrien & Kobayashi (2009)
*Velociraptor mongoliensis*	1.1139	1.5527	0.040606	29	Zelenitsky, Therrien & Kobayashi (2009)

Few ornithischian taxa are available for olfactory ratio comparisons with *Muttaburrasaurus langdoni*. Nevertheless, among Hadrosauridae, the olfactory bulbs of lambeosaurines are notably reduced (Evans, Ridgely & Witmer, 2009), as indicated by *Hypacrosaurus* sp., with the nasal chamber complexes thought to have functioned primarily for vocalisation and the crests for sexual display (Evans, Ridgely & Witmer, 2009; Weishampel, 1997). Thus, the function of the complex tubular crests of lambeosaurines substantially differs from the heightened olfactory function proposed herein for *Muttaburrasaurus langdoni*. Although the premaxillae of *Muttaburrasaurus langdoni* and lambeosaurines have followed similar developmental pathways to form complex dorsally protrusive airway chambers with the exclusion of the nasals from the nares, the function of the lambeosaurine crests and the dorsally hypertrophied muzzle of *Muttaburrasaurus langdoni* significantly differ. The suggested sensory function of the superior nasal chambers in *Muttaburrasaurus langdoni* for olfaction is tempered by the lengthy distance of the region from the anterior end of the olfactory bulbs (∼75 mm; Fig. 57). In other amniotes, the olfactory bulbs and olfactory diverticular are closely located (*e.g.*, Bourke et al., 2014; Evans, Ridgely & Witmer, 2009; Witmer & Ridgely, 2008b; Martinez et al., 2024)). If the superior airways functioned for olfactory reception in *Muttaburrasaurus langdoni*, as proposed, afferent signals along the olfactory nerves to the olfactory bulbs would have been slowed by distance.

### Vocalisation

The primary organ of acoustic production (phonation) in tetrapods is the larynx (syrinx in birds), driven by air circulation from the lungs, and is present in most extant tetrapod lineages (Colafrancesco & Gridi-Papp, 2016; Fitch & Suthers, 2016; Fitch & Suthers, 2016; Jorgewich-Cohen et al., 2022). The larynx has been identified in the ankylosaurid, *Pinacosaurus grangeri* and the early diverging neornithischian, *Pulaosaurus qinglong* (Yang, King & Xu, 2025; Yoshida, Kobayashi & Norell, 2023) but not identified in an ornithopod, although Weishampel (1997) considered its presence. The larynges of *Pinacosaurus grangeri* and *Pulaosaurus qinglong* were not considered by Yang, King & Xu (2025) and Yoshida, Kobayashi & Norell (2023); as the source of phonation in these taxa but acted to modify and enhance sound waves from a syrinx located more inferiorly in the trachea. Thus, the syringes in *Pinacosaurus grangeri* and *Pulaosaurus qinglong* were anticipated as the vocal source, as in birds. However, a syrinx has not been identified in a non-avialan dinosaur. Further modification of acoustic sound waves (vocalisation) occurs in regions of the head, such as the nasal cavities, sinuses and oral cavity ((Fitch & Suthers, 2016; Jorgewich-Cohen et al., 2022; Russel & Bauer, 2021). A detailed ancestral-state reconstruction of the choanate vertebrates (Dipnoi and Tetrapoda) by Jorgewich-Cohen et al. (2022) concluded that acoustic communication was an unambiguous homologous trait across the group. With this background, *Muttaburrasaurus langdoni* almost certainly used vocalisation for communication.

Enhanced vocalisation is considered to have been an important function of the crests in lambeosaurines (Weishampel, 1981; Weishampel, 1997). Expired air, pushed through the roughly tubular airways in the lambeosaurine crests, potentially enhanced sound waves from the larynx. Modelling by Weishampel (1981) suggested that the lengthy tubular nasal passages (region of the *vestibulum nasi*) of ∼2.5 m in the adult *Parasaurolophus*, as well as the narrow diameters of the passageways, was consistent with the propagation of very low-frequency sound, in the range of 50–350 Hz. Weishampel (1997) further hypothesised that the lateral diverticula in the crests of mature lambeosaurines supressed higher frequencies of vocalisation (*i.e.,* >400 Hz). As crest lengths in juvenile lambeosaurines were relatively short, the frequencies of vocalisation were likely higher (Weishampel, 1997). According to Weishampel (1997), all lambeosaurines possess extended nasal passages, although not of the length in *Parasaurolophus* and the complexity of the loop systems and chambers, as well as unknown aspects of the soft tissue anatomy, made it difficult to assess the character of their vocalisation.

Unlike lambeosaurines, the *vestibulum nasi* of *Muttaburrasaurus langdoni* is short, without passing through extended tubular passages. Modulating sound waves carried in expired air from the source of phonation in *Muttaburrasaurus langdoni* could have been modified in the domed chambers of the superior airway. If that occurred, expired air would have been directed into the superior airway through the paired posterior fenestrae (Fig. 57B). However, chambers of the superior airway in *Muttaburrasaurus langdoni* are not akin to the lengthy, tubular main airways of adult lambeosaurines (Evans, 2010; Weishampel, 1981). Furthermore, the divided superior airway of *Muttaburrasaurus langdoni* is not a blind meatus like the lateral diverticula in lambeosaurines and not likely to have acted in attenuating high-frequency sound waves, as a proposed function of the diverticula in lambeosaurines (Weishampel, 1997). If the superior airway of *Muttaburrasaurus langdoni* enhanced vocalisation, it is impossible to assess the sound frequencies produced, as the anatomical architecture of the soft tissues are unknown. Furthermore, if soft tissue turbinals extensively filled the superior airway chambers, as our assessment of olfaction suggests (see “Olfaction” above), significant soundwave resonance would seem unlikely.

Extending from studies on vocalisation in extant terrestrial vertebrates (*e.g.*, *alligator mississippiensis*, Garrick, Lang & Herzog, 1978; birds, Morton, 1975), Weishampel (1981) and Weishampel (1997) proposed that the propagation of low-frequency sound in lambeosaurines (<400 Hz) would have been beneficial to long distance communication between individuals in both open and closed habitats. High-frequency sound is attenuated in acoustically cluttered forest habitats and in open habitats degraded more rapidly than low-frequency sound during atmospheric transmission (Charlton, Owen & Swaisgood, 2019; Marten & Marler, 1977; Mitani & Stuht, 1998; Wiley & Richards, 1978). Optimal sound transmission, known as the sound window, falls within the range of 1–10 m above the ground surface (Marten & Marler, 1977; Morton, 1975). Moderately sized to large-bodied ornithopods, such as *Camptosaurus dispar*, hadrosaurids and *Muttaburrasaurus langdoni* were within this window, as were similarly sized saurischians. Below one metre, high and low-frequency sound is attenuated by ground absorption effects and scattering in both open and closed terrestrial environments (Marten & Marler, 1977; Wiley & Richards, 1978). Although low-frequency vocalisation has been regarded as the primary sound form produced by large-bodied ornithopods, low level sound attenuation could have impeded long distance vocal communication in small-bodied, ground-dwelling dinosaurs, such as *Gasparinsaura cincosaltensis* (Coria & Salgado, 1996), *Galleonosaurus dorisae* Herne et al., 2019 and *Hypsilophodon foxii* (Galton, 1974). Higher frequency vocalisation in dinosaurs (>1 kHz), could have been crucial to conspecific interactions and potentially group survival of dinosaur herbivores under visually impeded conditions. Charlton, Owen & Swaisgood (2019) have shown that high-frequency vocalisation assists directional positioning between conspecific animals in visibility poor, dense forest habitat. In addition, from their work on forest dwelling mammals, Charlton, Owen & Swaisgood (2019) showed that boosting the amplitude (energy) of higher frequency sound could overcome attenuation. This finding implies that higher frequency sound propagated at higher vocal energy could have been important to conspecific communication between herding dinosaurs, such as hadrosaurids, particularly when visual cues were impeded. As Yang, King & Xu (2025) and Yoshida, Kobayashi & Norell (2023);) have suggested from their vocalisation work on *Pulaosaurus qinglong* and *Pinacosaurus grangeri*, respectively, high-frequency sound modulations could have been initially propagated in the syrinx of ornithischians, as in birds, and modified further in the larynx. Phylogenetic bracketing (Witmer, 1995) would suggest that these sound propagation and modification traits could have been generally distributed across Dinosauria, from Ornithischia to Avialae.

Enhanced resonance and modulation of soundwaves from the phonation source was possible in *Muttaburrasaurus langdoni*, from the larynx, pharynx, nasal cavities, sinuses and oral cavity (based on information in Fitch & Suthers, 2016; Fitch et al., 2025; Jorgewich-Cohen et al., 2022; Titze, 2023). Lung pressure was crucial to soundwave amplitude (Titze, 2023). It is notable that extant large-bodied palaeognaths, typified by the emu (*Dromaius novaehollandiae*) and cassowary (*Casuarius* spp.) only use very low-frequency sound (possibly as low as 20 Hz) for communication over substantial distances (Corfield, Kubke & Köppl, 2013; Mack & Jones, 2003)—the cassowary in dense rainforest and the emu in open forest and plains. Low-frequency sound (<1 kHz) could have been beneficial to *Muttaburrasaurus langdoni* for long distance, conspecific communication in open and closed (forest) habitats. Higher frequency sound, at optimal acoustic energies to minimise predator detection (Mitani & Stuht, 1998), could have been beneficial to *Muttaburrasaurus langdoni* for close, directional conspecific communication in forest habitats, particularly if *M. langdoni* had been involved in close communal behaviour, such as herding (see also under “Visual fields in *Muttaburrasaurus langdoni*” below) and group nesting, as identified in the hadrosaurids. Specific anatomical characteristics of the airways, cranial chambers and sinuses would have propagated unique vocalisation in *Muttaburrasaurus langdoni* and with it, coevolution of hearing sensitivity to the frequencies and modulations produced (see also under “Head posture, auditory capacity, balance and locomotion” below).

### Extrarenal salt excretion?

With less efficient osmoregulatory functioning of the kidney than in mammals, extrarenal salt excretion mechanisms are necessary in extant sauropsids that ingest excess salts in food and collaterally with water (Cowgill et al., 2023; Dunson, 1976; Hazard, Shoemaker & Grismer, 1998; Schmidt-Nielsen, 1959). Sea birds and many extant reptiles (lizards, sea turtles, sea snakes, neosuchian crocodilians) possess cephalic exocrine glands adapted for the excretion of excess salt loads (primarily the monovalent ions, Na^+^, K^+^, Cl^−^) (Dunson, 1976; Dunson, Packer & Dunson, 1971; Hazard, 2001; Hazard, Shoemaker & Grismer, 1998; Schmidt-Nielsen, 1959; Schmidt-Nielsen, 1960; Schmidt-Nielsen & Fange, 1958; Taplin et al., 1982; Templeton, 1967). Potassium, also, is generally abundant in plant tissues and sodium-chloride can be collaterally ingested with plant food in coastal and brackish water localities, as previously hypothesised for non-avialan dinosaurs by Osmólska (1979). The salt excretion glands of birds and reptiles are highly efficient at removing salt and actively pump out sodium-chloride from the blood stream against the osmotic gradient (see Schmidt-Nielsen, 1959; Schmidt-Nielsen, 1960). Cephalic salt glands in some extant lizards and, as assessed in some early diverging crocodyliforms (notably thalattosuchian metriorhynchids and potentially teleosaurids), are developed within the *cavum nasi proprium* as exaptations of the nasal glands, which typically moisten the airways (Brusatte et al., 2016; Cowgill et al., 2023; Dunson, 1969; Fernández & Gasparini, 2008; Hazard, Shoemaker & Grismer, 1998; Herrera, Fernández & Gasparini, 2013). In extant iguanians and interpreted in metriorhynchids, salt glands in the *cavum nasi proprium* can be markedly hypertrophied (Cowgill et al., 2023; Fernández & Gasparini, 2000; Hazard, Shoemaker & Grismer, 1998). The bones of the cranial roof in the iguanian genus *Uta* are expanded to accommodate hypertrophied salt glands (Grismer, 1994). The enlarged salt glands of the Galapagos marine iguana (*Amblyrhunchus cristatus*) are housed in nasal chambers formed in anterior extensions of the frontals (Paparella & Caldwell, 2022). Hypertrophied salt glands housed by the nasals have been proposed by Fernández & Gasparini (2000), Fernández & Gasparini (2008) in the metriorhynchid *Geosaurus araucanensis*, identified from the rare preservation of glandular lobules in the nasal endocast.

The occurrence of cephalic salt glands in large-bodied herbivorous dinosaurs was proposed by Osmólska (1979) and further hypothesised in hadrosaurids by Whybrow (1981). According to Osmólska (1979), salt glands were potentially developed in the subnarial/prenarial fossae of ceratopsians, hadrosaurids and sauropods—thus, external to the *cavum nasi proprium*. Contrary to Osmólska (1979), Whybrow (1981) argued against salt glands in the anteroventrally located narial fossae of hadrosaurids and speculated on their occurrence more internally in the lateral diverticula in lambeosaurines and in the preorbital, circumnarial depressions of saurolophines, rather than anterior to the nares. In agreement with Maryańska (1977) and Hill, Witmer & Norell (2003), considered a salt gland likely in the premaxilla of the ankylosaurid *Pinacosaurus grangeri*. Notably, Wang et al. (2018) dismissed all earlier suggestions of cephalic salt glands in non-avialan dinosaurs, arguing that the first conclusive evidence of extrarenal salt glands in a dinosaur was evident from fossae in the supraorbital region of an Early Cretaceous (Aptian) ornithurine bird from China. Wang et al. (2018) further argued that salt excretion in non-avialan dinosaurs more likely occurred through the renal system. However, that view differs from established scientific understanding of the functional incapacity of the kidneys in extant sauropsids to remove excess salts. When phylogenetic bracketing is considered (Witmer, 1995; Witmer, 2001), with inferences constrained by extant birds and crocodilians (and also informed by Testudines and Lepidosauria), non-avialan dinosaurs that ingested excess salt in their diets were likely to have required an extrarenal means of salt removal (see also Hill, Witmer & Norell, 2003; Witmer, 1997b). It seems reasonable to suggest that at least some of the herbivorous ornithischian faunae who inhabited near coastal margins and salty or brackish water localities, such as upper Late Cretaceous hadrosaurids and ceratopsians discovered in the Dinosaur Park Formation of North America (Eberth, 2005; Eberth & Brinkman, 1997; Ryan & Evans, 2005) and ornithopods of the upper Late Cretaceous, European archipelago (Csiki-Sava et al., 2015), required extrarenal salt removal. Thus, the occurrence of cephalic salt glands in herbivorous non-avialan dinosaurs should not be dismissed. However, finding convincing evidence for the presence of cephalic glandular soft tissues in dinosaurs is challenging and particularly within the nasal airways (Bourke et al., 2014). Notably, as far as we are aware, evidence for glandular lobules in a fossil vertebrate characteristic of salt-excretion glands (see Babonis & Brischoux, 2012), has only been documented in the Late Jurassic metriorhynchids, *Geosaurus araucanensis* (Fernández & Gasparini, 2000, Fernández & Gasparini, 2008) and *Cricosaurus araucanensis* (Herrera, Fernández & Gasparini, 2013).

Skeletal fossils of *Muttaburrasaurus* (*M. langdoni* (QMF6140) and *M.* spp. (QMF12541, QMF14921)) are only known from shallow marine strata of the epeiric Eromanga Sea (Bartholomai & Molnar, 1981; Molnar, 1996). These individuals of *Muttaburrasaurus* are likely to have floated from land and into the Eromanga Sea as bloated carcasses (Bartholomai & Molnar, 1981), possibly during storm events. This assessment is supported by our interpretation of the holotype locality as a marine storm-surge/tempestite deposit. The habitat of *Muttaburrasaurus* would have been close enough to the coast for the carcasses of the individuals to wash out to sea. *Muttaburrasaurus* potentially lived on the coastal plain, possibly close to the shoreline or in the vicinity of distal rivers, salt marshes and estuaries. In these locations, *Muttaburrasaurus* could have collaterally ingested sodium salt or salt water while consuming food, such as through feeding on salt tolerant plants or plants coated with salt spray residue (*e.g.*, Meng, Zhou & Sui, 2018; Stevens et al., 2025). Equisetaceae (horsetail family), for example, which are thought to have been a highly nutritious and digestible food type for herbivorous dinosaurs (Howel et al., 2023), are known from the mid-Cretaceous of the Eromanga Basin (McLoughlin, Pott & Elliott, 2010). Notably, the extant giant horsetail (*Equisetum giganteum*) is sodium salt tolerant (Husby et al., 2011), suggesting that equisetacean relatives in the Cretaceous could have been salt tolerant in coastal and estuarine locations bordering the Eromanga Sea. Work by Chin, Feldmann & Tashman (2017) found fossil evidence of crustaceans in the diet of hadrosaurid megaherbivores. If *Muttaburrasaurus* had consumed invertebrates in salty areas, salt water could have been collaterally ingested. However, without compelling evidence of plant or animal food types preserved in fossilised stomach contents (cololites) or associated coprolites of *Muttaburrasaurus* to implicate particular plant and animal dietary preferences, proposing the ingestion of excess salt in this genus is hard to support (see also Chin, Feldmann & Tashman, 2017 and Sander et al., 2010 on plant identification issues in cololites and coprolites of herbivorous dinosaurs). Nevertheless, an osseous feature on the skull of *Muttaburrasaurus langdoni* is of interest.

A distinctively protrusive lateral oriented bulla is preserved on the left side of the holotype skull, located on the anterolateral process of the nasal and adjoining posteroventral process of the premaxilla (Figs. 20C, 56, 57). Internally, this bulla forms an enlarged, pocket-like fossa laterally in the *cavum nasi proprium* (Fig. 57A). The lateral fossa in the nasal tract of *Muttaburrasaurus langdoni* is comparable to the location of the hypertrophied salt excretion glands in lizards and metriorhynchids. However, fossilisation of glandular lobules is not apparent in the CT imagery of the lateral fossa and the function of the fossa cannot be categorically ascertained. Furthermore, if the fossa housed glandular tissue, functions such as mucus secretion for moistening, filtering and conditioning inhaled air, aiding olfaction and aiding in the removal of inhaled pathogens and toxicants, were possible (Harkema, Fau & Wagner, 2006). However, if the diet of *Muttaburrasaurus* had included the ingestion of excess salts, a salt-excretion function seems possible. If ingested, excess salt in *Muttaburrasaurus langdoni* was likely removed from arterial blood from the ophthalmic artery, which branches from the cerebral carotid artery in the *sella turcica*, as in birds (Schmidt-Nielsen, 1960) and proposed in metriorhynchids (Brusatte et al., 2016; Herrera, Fernández & Gasparini, 2013) (see Figs. 33C, 34A, 34B, 40D, 40E).

Although speculative, the suggested function of the lateral nasal fossa in *Muttaburrasaurus langdoni* as having accommodated a salt excretion gland is not without reason. It is hoped this preliminary work will encourage further exploration of extrarenal osmoregulation in herbivorous dinosaurs that inhabited near-coastal locations where the collateral ingestion of excess salt was possible. For example, geologically derived stable strontium isotopes taken up by plants have been used to assess the diet and migrations of hadrosaurids from the analysis of their tooth enamel (Terrill, Henderson & Anderson, 2020). The analysis of stable strontium isotopes held in the tooth enamel could offer a future means of testing for the ingestion of marine derived sodium salt in the diet of *Muttaburrasaurus langdoni* using the marine derived strontium signature (^87^Sr/^86^Sr) as a proxy for sodium salt ingestion (Armaroli et al., 2025; Stevens et al., 2025). Marine derived strontium, along with sodium salt, is atmospherically transported from the marine environment onto land in heavy rainfall and sea spray (Stevens et al., 2025; and authors within); thus, resulting in the coating of plants with salt. Notably, strontium isotopes in tooth enamel have been shown to resist diagenetic alteration (Stevens et al., 2025; and authors within), suggesting that a strontium signature could provide a useful proxy of sea salt ingestion if diagenetic alteration can be adequately isolated.

### Brain size, cognition and locomotion

Encephalisation quotient as originally elaborated by Jerison (1973), provides a comparative measure of cognition between mammals across multiple lineages utilizing brain and body mass data. Encephalisation quotient is assessed by deviations (residuals) from the line of regression of expected brain mass to body mass. In effect, this line of regression is often interpreted as relating to somatic (non-cognitive) functions of the body (see Van Schaik et al., 2021). Deviations from the expected slope (after the input of brain and body mass data) are thought to reflect cognitive capabilities, from which comparisons can be made between species (see Van Schaik et al., 2021; Shultz, 2010). Encephalisation quotient, in its original form, was widely used as a proxy for relative cognition between fossil taxa in tetrapod clades of interest (Jerison, 1979). Hurlburt (1996) adjusted the original encephalisation quotient regression for more specific application to reptiles (see also in Hurlburt, Ridgely & Witmer, 2013). The reptile encephalisation quotient (REQ) has been used to compare cognition between non-avian dinosaurs (Brasier et al., 2017; Button & Zanno, 2023; Evans, Ridgely & Witmer, 2009; Hopson, 1979; Hurlburt, 1996; Hurlburt, Ridgely & Witmer, 2013; Lauters et al., 2012).

The usefulness of the encephalisation quotient as a measure of cognition between extant vertebrate taxa has been questioned based upon multiple issues (see Herculano-Houzel, 2017; Shultz, 2010; Storks, Powell & Leal, 2023; Van Schaik et al., 2021; Todorov et al., 2019). Difficulties exist in accurately determining true brain volume in extinct taxa (Brasier et al., 2017; Evans, Ridgely & Witmer, 2009; Knoll et al., 2021; Witmer & Ridgely, 2008b) and unknown differences in neuron density and architecture can lead to error in the comparative assessment of cognition (Caspar et al., 2024; Shultz, 2010). Debate has surrounded estimates of the thickness of the protective dural meninges that would have encased the brain in non-avialan dinosaurs (see Evans, Ridgely & Witmer, 2009; Hurlburt, Ridgely & Witmer, 2013; Knoll et al., 2021; Witmer & Ridgely, 2008b). Different estimates of dural meninges thickness can significantly affect the estimate of brain mass used to calculate encephalisation quotient. The accurate estimate of body mass in fossil taxa is problematic, with body mass estimates in non-avialan dinosaurs varying by orders of magnitude between volumetric-based and stylopodial-based methods (Dempsey et al., 2025). This issue is evident from the body mass estimates for *Muttaburrasaurus langdoni* (see under “Body mass of the *Muttaburrasaurus langdoni* holotype” above). Ideally, the calculation of encephalisation quotient should use the same method of body mass estimation for the taxa of interest. However, such a task is difficult to achieve, as workers undertaking calculations and comparisons of encephalisation quotients are generally reliant on body and brain mass estimates from literature sources. Despite the issues with encephalisation quotient methodology and, in the absence of neural soft tissue, encephalisation quotient has endured as a proxy for comparisons of cognition between fossil taxa (Van Schaik et al., 2021). Accepting the caveats, REQ has been used as a measure of relative cognition between non-avialan dinosaurs (Barker et al., 2017; Button & Zanno, 2023; Evans, Ridgely & Witmer, 2009; Knoll et al., 2021; Sampson et al., 1998; Witmer & Ridgely, 2009), albeit with the presumption that cerebral neuron density and the percentage of brain-fill in the neural fossa are equivalent and that brain and body masses have been accurately estimated.

**Figure 59:**
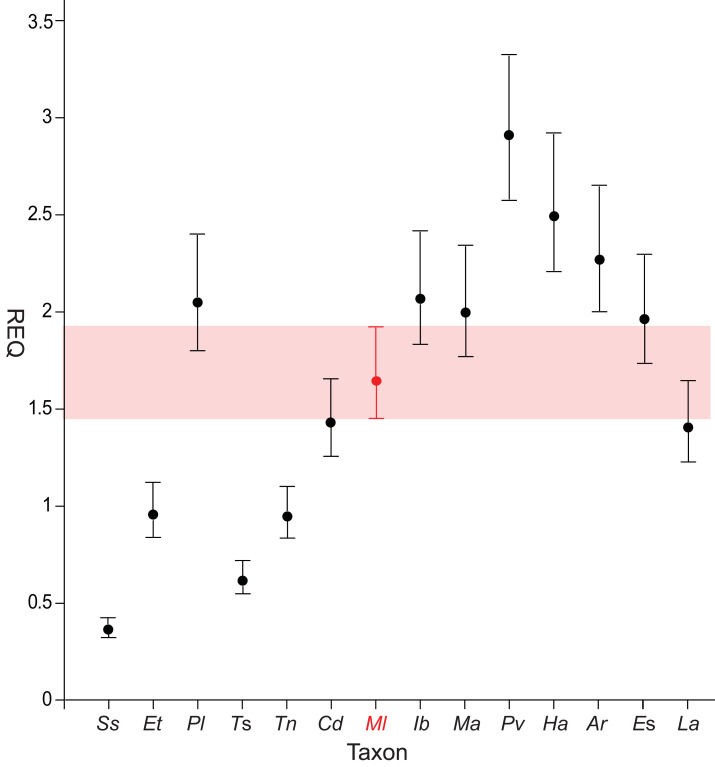
Plot of reptile encephalisation quotients (REQ) for selected neornithischians. Taxon abbreviations: *Ar*, * Amurosaurus riabinini*; *Cd*, *Camptosaurus dispar*; *E*s, *Edmontosaurus*; *Et*, *Euplocephalus tutus*; *Ha*, *Hypacrosaurus altispinus*; *Ib*, *Iguanodon bernissartensis*; *La*, *Lurdusaurus arenatus*; *Ma*, *Mantellisaurus atherfieldensis*; *Ml*, *Muttaburrasaurus langdoni*; *Pv*, *Proa valdearinnoensis*; *Pl*, *Psittacosaurus lujiatunensis*; *Ss*, *Stegosaurus stenops*; *Tn*, *Thescelosaurus neglectus*; *T*s, *Triceratops* sp. For data sources, see Table 3. Error bars equal ±25%. Red shaded area indicates error range for *Muttaburrasaurus langdoni*.

The REQ for *Muttaburrasaurus langdoni* was calculated using the brain-to-endocranial cavity correlation index (BEC) of 60% and the body mass estimate retrieved using cQE (see “Methods”). The results and comparisons are shown in Fig. 59. The REQ for *Muttaburrasaurus langdoni* falls below the lambeosaurines, *Amurosaurus riabinini*, *Hypacrosaurus altispinus* and the earlier diverging hadrosauriform, *Proa valdearinnoensis*. The REQ for *Muttaburrasaurus langdoni* is higher than that of *Lurdusaurus arenatus* but overlaps within error. Although REQs for the early diverging hadrosauriforms, *Iguanodon bernissartensis* and *Mantellisaurus atherfieldensis* and the saurolophine *Edmontosaurus* sp., are higher than that of *Muttaburrasaurus langdoni*, they overlap within error. The REQ for *Muttaburrasaurus langdoni* is higher than that of the early diverging ankylopollexian, *Camptosaurus dispar*, but overlapping within error. The REQ for *Muttaburrasaurus langdoni* is clearly higher than those of the small-bodied, early diverging neornithischian, *Thescelosaurus neglectus*, the large-bodied thyreophorans, *Stegosaurus stenops* and *Euplocephalus tutus* and the large-bodied ceratopsian, *Triceratops* sp. However, the REQ for the relatively small-bodied (∼250 kg) early diverging ceratopsian, *Psittacosaurus lujiatunensis*, is higher than that of *Muttaburrasaurus langdoni*. It is of note that the large brain to body size in psittacosaurs (as well as their acute vision and olfaction and likely bipedal locomotion) is thought to reflect cognition associated with an agile lifestyle and predator avoidance (Zhou et al., 2007); behavioural features consistent with their high REQ. The REQ and cognitive capacity of *Muttaburrasaurus langdoni* likely falls within the level of early diverging Iguanodontia, although more extensive data for this group would help this assessment. However, it is notable that among the taxa plotted (Fig. 59), the early diverging hadrosauriform, *Proa valdearinnoensis*, has the highest REQ. Unlike *Muttaburrasaurus langdoni*, the cerebral region of the endocast in *Proa valdearinnoensis* is markedly expanded (Knoll et al., 2021), which seems to be reflected in the high REQ (but see further discussion below). A bulbous cerebral region has also been noted in hadrosaurids linked to their high REQs relative to other ornithischians (Evans, Ridgely & Witmer, 2009; Hopson, 1979). High REQs in hadrosaurids are thought to reflect a lifestyle involving highly social community behaviour, discussed further below. Although *Proa valdearinnoensis* is not a hadrosaurid, the high REQ suggests behavioural complexity comparable to hadrosaurids.

The cerebrum is the primary centre of cognition in vertebrates (Striedter & Northcutt, 2019). Expansion and reorganisation of the cerebrum during the evolution of vertebrates have been linked to more complex locomotion, such as upright bipedal locomotion, flight and increased cognitive abilities (Shultz, 2010; Spool et al., 2021; Striedter & Northcutt, 2019). Rapid mapping of complex sensory stimuli, encoding numerical information, vocal learning and decision-making, based on abstract rules, have been linked to increased cerebral size in birds and mammals (Spool et al., 2021). Encephalisation quotients do not specifically distinguish between the size of the cerebrum and the rest of the brain. Nevertheless, relative cerebral size has been considered as a more direct measure of relative cognition between closely related ornithischians (Evans, Ridgely & Witmer, 2009; Knoll et al., 2021; Lauters et al., 2012). Comparative cerebral mass to brain mass (MCb:MBr) has been used as a relative measure of cognition among dinosaurs, which we employ here for our assessment of cognition in *Muttaburrasaurus langdoni*, using adjustment factors for archosaurs as outlined by Hurlburt 2013 (see in “Methods”).

A MCb:MBr of 45.3% was calculated for *Muttaburrasaurus langdoni*. Although the REQ of *Muttaburrasaurus langdoni* is lower than the holotypic adult of *Iguanodon bernissartensis* (RBINS 51) (Fig. 59), the MCb:MBr is substantially higher than that of the latter (21%) (Table 5). The comparatively low cerebral masses in *Iguanodon bernissartensis* (RBINS 51) and *Lurdusaurus arenatus* (Table 5) suggests that more of the brain in these early diverging hadrosauriforms had been involved with somatic functions than in *Muttaburrasaurus langdoni*. However, the MCb:MBr values for *Iguanodon bernissartensis* and *Lurdusaurus arenatus* (originally reported as cerebral volume to endocast volume (CRV)s by Lauters et al., 2012; see further in “Methods”), seem extraordinarily low, suggesting that the method for cerebocast measurement differed from that used herein for *Muttaburrasaurus langdoni*, or needs revision. At 49.7%, the MCb:MBrs of the saurolophines *Edmontosaurus* sp. and *Gryposaurus notabilis* are higher than for *Muttaburrasaurus langdoni* and correlate with the relative REQs between *Muttaburrasaurus langdoni* and *Edmontosaurus* sp. The MCb:MBr of 47.5% for the lambeosaurine, *Hypacrosaurus altispinus* (ROM 702), is marginally higher than for *Muttaburrasaurus* and their MCb:MBrs would most likely overlap within error. The most surprising difference in MCb:MBr and REQ was in *Proa valdearinnoensis*. Although the REQ of *Proa valdearinnoensis* (3.02) was higher than the other taxa compared, the MCb:MBr (43.2%) was relatively low (Tables 3, 5). As for *Iguanodon bernissartensis* and *Lurdusaurus arenatus*, the differences between REQ and MCb:MBr in *Proa valdearinnoensis* suggest that more of the brain could have been involved in somatic functions than detected by REQ.

**Table 5:** Table of relative cerebral volume to endocast volume (CRV) and relative cerebral mass to brain mass (MCb:MBr) for selected ornithopods.

Taxon	CRV (%)	MCb:MBr (%)	CRV source
*Corythosaurus* sp. sub-adult	35	39.7	Evans, Ridgely & Witmer (2009)
*Edmontosaurus* sp.	45	51.1	Evans, Ridgely & Witmer (2009)
*Gryposaurus notabilis*	45	51.1	Evans, Ridgely & Witmer (2009)
*Hypacrosaurus altispinus*	43	48.8	Evans, Ridgely & Witmer (2009)
*Iguanodon bernissartensis*	19	21.6	Lauters et al. (2012)
*Lurdusaurus arenatus*	19.2	21.2	Lauters et al. (2012)
*Muttaburrasaurus langdoni* (QMF6140)	41	46.5	This study
*Proa valdearinnoensis*	39.1	44.4	Knoll et al. (2021)

The potentially higher cognitive abilities of hadrosaurids inferred from REQ, relative to early diverging ornithopods, has been thought to reflect more complex social behaviour (Evans, Ridgely & Witmer, 2009; Witmer & Ridgely, 2008b). Hadrosaurid bonebeds, for example, have suggested gregarious herding behaviour and complex communal nesting behaviour has also been identified (*e.g.*, Horner, 1984; Horner & Makela, 1979; Varricchio & Horner, 1993). Herding behaviour in hadrosaurids potentially provided protection against predation (*e.g.*, Botfalvai, Prondvai & Osi, 2021; Lockley, Young & Carpenter, 1983), particularly for juveniles (see Hone & Rauhut, 2010). As the skeletal remains of *Muttaburrasaurus langdoni* and *M*. spp. (QMF12541, QMF14921) have only been found in marine deposits, complex behaviours, such as herding, communal nesting and migration have yet to be ascertained. Such understanding will take the future discovery of *Muttaburrasaurus* remains in terrestrial deposits. MCb:MBr in *Muttaburrasaurus langdoni* resembles that of the lambeosaurines, suggesting *M. langdoni* could have shared at least some of the complex behavioural attributes of the hadrosaurids. However, the complex, community-based behavioural attributes of hadrosaurids seemingly linked to higher cognition, is tempered by some understanding of large-bodied ceratopsian behaviour. Even though brain size in *Pachyrhinosaurus lakustai* was noted by Witmer & Ridgely (2008b) as “small”, suggesting lesser cognitive function than contemporaneous large-bodied ornithopods, herding behaviour, possibly migratory behaviour and complex conspecific display, have been considered likely (Currie, 1992; Currie, Langston Jr & Tanke, 2008; Sampson, 2001; Sampson, Ryan & Tanke, 1997). Similarly to the level of cognition suggested in *Pachyrhinosaurus lakustai*, the REQ for *Triceratops* sp. is relatively low (1.0) (Fig. 59) but complex gregarious behaviour was proposed (Mathews et al., 2009). Thus, herding, migration and complex social interactions were not necessarily behavioural characteristics reflected in the higher cognitive levels of derived large-bodied ornithopods but potentially were systemic behaviours in herbivorous ornithischians, including those with modest brain sizes, linked to protection against predation, particularly for juveniles (Mathews et al., 2009; Hone & Rauhut, 2010), survival across changing seasons and breeding between the fittest mates.

Reptile encephalisation quotients for *Muttaburrasaurus langdoni* and other ornithopods, including those thought to be facultatively bipedal, are higher than those estimated for large-bodied, obligatory quadrupedal dinosaurs, for which data are available. In this latter group, reptile encephalisation quotients for sauropods, such as *Diplodocus carnegeii* and *Nigersaurus taqueti* and the ornithischians, *Euplocephalus tutus*, *Kentrosaurus aethiopicus*, *Stegosaurus stenops* and *Triceratops* sp. are at or below 1.0 (Button & Zanno, 2023; Evans, Ridgely & Witmer, 2009; Hurlburt, 1996) and for some, well below (*Stegosaurus stenops* and *Triceratops* sp; Fig. 59). The lower encephalisation quotients for large-bodied obligatory quadrupedal dinosaurs than comparatively sized ornithopods, has been considered to reflect differences in cognitive behaviour. However, the relatively high REQs and MCb:MBrs in large-bodied ornithopods who, at least for some periods of activity, were facultatively bipedal, particularly during running (Evans, Ridgely & Witmer, 2009; Forster, 1997; Horner, Weishampel & Forster, 2004), suggests the possibility that brain size and locomotory style could have been linked. Notably, complex locomotion and upright posture in birds and hominids is reflected in the evolutionary expansion of the cerebrum, although in the case of extant flying vertebrates (birds and bats), and from the evolutionary perspective, the cerebral cortex progressively reduced in weight through neuron compaction (Herculano-Houzel, 2017). Bipedal locomotion in large-bodied non-avialan dinosaurs potentially required rapid navigational decisions to prevent serious injury from falling, not experienced to the same degree by obligatory quadrupeds (see further under “Head posture, auditory capacity, balance and locomotion”, below).

Given this tentatively suggested link between cerebral size and bipedal locomotion in non-avialan dinosaurs, the expanded cerebrum, relatively high REQ and MCb:MBr for hadrosaurids, such as *Edmontosaurus* sp. and *Gryposaurus notabilis* and *Hypacrosaurus* is notable (Table 5). Although the cerebrum of *Muttaburrasaurus langdoni* does not share the bulbous form in that of the hadrosaurids, the size of the cerebrum, indicated by its MCb:MBr, is comparable to that group. Compared to the hadrosaurids, the low REQ and MCb:MBr for the hadrosauriform, *Lurdusaurus arenatus*, could reflect its stocky, graviportal, predominantly quadrupedal form (Taquet & Russell, 1999). The relatively small, early diverging neornithischian, *Thescelosaurus neglectus* (body mass of ∼340 g; Button & Zanno, 2023), is within a group that have been generally regarded as agile, cursorial bipeds (*e.g.*, Galton, 1971; Herne, 2014; Herne et al., 2018; Thulborn, 1972; Thulborn, 1982). However, the low REQ in *Thescelosaurus neglectus* of ∼1.0 seems consistent with the graviportal posture and facultatively quadrupedal locomotion suggested by its limb proportions (see Button & Zanno, 2023; and authors within).

The REQs and MCb:MBrs of *Muttaburrasaurus langdoni* and most of the hadrosauriforms included in this study, are within the range of those reported for some large-bodied, obligatory bipedal theropods (*Carcharodontosaurus saharicus* (SGM Din-1), REQ 1.73–2.08, MCb:MBr 35.8–42.1%; *Gorgosaurus libratus* (ROM 1247), REQ 2.35, MCb:MBr 49.3%; *Tyrannosaurus rex* (FMNH PR 2081) REQ 2.07–3.07, MCb:MBr 46.6–49.3%; *Allosaurus fragilis* (UUVP 294), REQ 2.29–3.00, MCb:MBr 61.4%; Hurlburt, 2013; noting the BECs are recalculated for 60% and the REQ ranges follow the body mass ranges as reported), supporting the view that higher cognition in bipedal dinosaurs could have facilitated safe bipedal locomotion, while noting that high cognition in theropods has also been considered linked to hunting behaviour (*e.g.*, Witmer et al., 2008). The proportions of the semicircular canals further support suggested locomotion in *Muttaburrasaurus langdoni* as predominantly bipedal, as outlined in the section that follows. The potential link between brain size and locomotion in dinosaurs, suggested herein, is an area for further investigation. However, such a study would require the grounding of REQ and MCb:MBr measurements by using equivalent methods of brain mass and body mass acquisition and an extensive taxon dataset.

Apart from *Lurdusaurus arenatus* Taquet & Russell, 1999; (Lauters et al., 2012), from Niger, the small-bodied ornithopod, *Leaellynasaura amicagraphica* Rich & Rich, 1989, from the Aptian of Victoria in southeastern Australia and *Muttaburrasaurus langdoni* conducted here, assessments of encephalisation quotient have not been attempted on any other Gondwanan ornithopod. The encephalisation quotient of *Leaellynasaura amicagraphica*, reported by Rich & Rich (1989), was not included here, as multiple, cross taxic scaling assumptions were made from the specimens referred to the taxon, as parts of the holotype (see Herne, Tait & Salisbury, 2016, on taphonomic issues with the assignment of materials to *Leaellynasaura amicagraphica*). At present, apart from *Lurdusaurus arenatus*, the only other Gondwanan ornithopods with complete braincases and associated postcrania suitable for comparative analyses of encephalisation, are the small-bodied Elasmarian *Gasparinisaura cincosaltensis* Coria & Salgado, 1996, from Argentina, and the large-bodied hadrosauriform, *Ouranosaurus nigeriensis* Taquet, 1976, from Niger. Endocranial studies have yet to be published on these taxa. The brain endocast was described for the Argentinian hadrosaurid *Secernosaurus koerneri* (Becerra et al., 2018); however, measurements of encephalisation were not conducted.

### Head posture, auditory capacity, balance and locomotion

The endosseous labyrinths of the *Muttaburrasaurus langdoni* holotype are well preserved. From the semicircular and auditory canals, we can make predictions on head posture, locomotory behaviour and hearing capacity for the taxon.

*Semicircular canals*—The six semicircular canals in vertebrates transduce angular accelerations of the head as sensory afferents to the brain from changes in the velocity of endolymphatic movements through the canals, sensed at their cupulae (Angelaki & Cullen, 2008; Malinzak, Kay & Hullar, 2012; Sipla, Georgi & Forster, 2004; Spoor, Wood & Zonneveld, 1994; Spoor et al., 2007). The lateral semicircular canals (LSC) sense yaw (left–right rotation of the head in the dorsal plane); the anterior semicircular canals (ASC) sense pitch (up and down motion of the head in the sagittal plane) and the posterior semicircular canals (PSC) sense roll (tilting of the head to the left or right in the transverse plane). The sensory cues to the brain result in auto-vestibulo-ocular and auto-vestibulo-collic reflex responses to coordinate body and head movements during locomotion and to stabilise gaze through innovation of the extraocular and ciliary musculature (Bronzati et al., 2021; Fritzsch, 2024; Land & Nilsson, 2012; Spoor et al., 2007; and authors within). As work on human subjects has shown, the semicircular canals control balance, spatial orientation and propagate cues for cognitive navigational mapping (Angelaki & Cullen, 2008; Fitzpatrick, Butler & Day, 2006). Longer or larger semicircular canals have greater sensitivity to motion than relatively shorter canals and can induce rapid, early, fine-scale, vestibulo-ocular and vestibulo-collic adjustments in response to head motion. Longer or larger canals have been linked to an upright limb posture agility in birds and mammals (Bronzati et al., 2021; Benson et al., 2017; Hanson et al., 2021; Spoor et al., 2007 and authors within; but see Hullar, 2006 on contradictory issues). Notably, bipedal animals are inherently more unstable than quadrupeds (Georgi, Sipla & Forster, 2013). The anterior semicircular canals detect pitching/bobbing of head in the sagittal plane—particularly experienced during bipedal locomotion (Sipla, Georgi & Forster, 2004). Notably, longer ASCs, relative to the PSCs, correlate with bipedal locomotion in mammals and birds (Bronzati et al., 2021) and have been used to infer bipedal locomotion in non-avialan dinosaurs (Benson et al., 2017; Bronzati et al., 2021; Georgi, Sipla & Forster, 2013; Hanson et al., 2021; Sipla, Georgi & Forster, 2004; Spoor et al., 2007; Witmer & Ridgely, 2009), including in ornithopods (Cruzado-Cabellaro et al., 2015; Thomas, 2015). The three semicircular canals of dinosaurs, in general, are close to orthogonally oriented (Bronzati et al., 2021), a trait that has been correlated with agility in primates (Malinzak, Kay & Hullar, 2012). Furthermore, because of the highly conserved nature of the semicircular canals across vertebrates, Malinzak, Kay & Hullar (2012) considered that closely orthogonal canals were a likely indicator of agility in other vertebrate clades.

**Figure 60:**
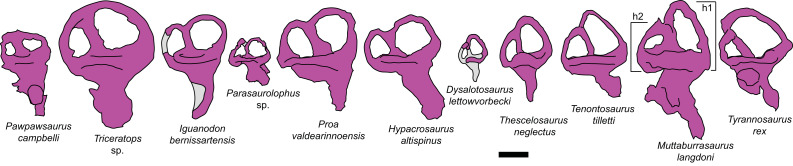
Schematic endosseous labyrinths of selected dinosaurs in right lateral view. Endoseous labyrinths aligned at the lateral semicircular canals. Specimen sources: *Dysalotosaurus lettowvorbecki* (sub-adult; Lautenschlager & Hübner, 2013, fig. 2); *Hypacrosaurus altispinus* (Evans, Ridgely & Witmer, 2009, fig. 8; left reflected to right); *Iguanodon* sp. (Norman, 2004, fig. 19.9); *Muttaburrasaurus langdoni* (this study); *Parasaurolophus* sp. (juvenile; Farke et al., 2013, fig. 16); *Pawpawsaurus campbelli* (Paulina-Carabajal, Lee & Jacobs, 2016), fig. 6; left reflected to right); *Proa valdearinnoensis* (Knoll et al., 2021), fig. 5); *Tenontosaurus tilletti* (Thomas, 2015); *Thescelosaurus neglectus* (Button & Zanno, 2023, fig. 2); *Triceratops* sp. (Sakagami & Kawabe, 2020, fig. 3); *Tyrannosaurus rex* (Witmer & Ridgely, 2009, fig. 8). Abbreviations: h1, height of anterior semicircular canal; h2, height of posterior semicircular canal. Scale bar equals one cm.

The semicircular canals of *Muttaburrasaurus langdoni* are orthogonally oriented, consistent with other dinosaurs, as well as agility. The ASC of *Muttaburrasaurus langdoni* is markedly longer than the PSC (Table 2). The ASC axial length is 1.52:1 of the PSC length (based on the mean of the left and right sides) (Fig. 60). The height of the ASC relative to the PSC is 1.45:1, measured from the canal base (h1 and h2 in Fig. 60). From the longer and taller ASC in *Muttaburrasaurus langdoni*, an upright limb posture and bipedal locomotion are inferred (based on information in Bronzati et al., 2021). Comparisons between the semicircular canals of *Muttaburrasaurus langdoni* and selected non-avialan dinosaurs are shown in Fig. 60. The height of the ASCs to PSCs in the facultative bipeds, *Dysalotosaurus lettowvorbecki* (1.41:1), *Tenontosaurus tilletti* (1.30:1) and *Thescelosaurus neglectus* (1.41:1) are comparable to *Muttaburrasaurus langdoni*. The height of the ASCs to PSCs in the large-bodied, obligatory bipedal theropod, *Tyrannosaurus rex* (∼1.67:1) is greater than the ornithopodan facultative bipeds. In the facultatively quadrupedal/part-time (facultatively) bipedal hadrosauriforms (Horner, Weishampel & Forster, 2004; Norman, 2004), *Iguanodon* sp. (1.07:1), *Hypacrosaurus altispinus* (1.25:1) *Parasaurolophus* sp. (1:1) and *Proa valdearinnoensis* (1.16:1), the relative height of the ASCs are closer or sub-equal to the PSCs, noting that the specimen of *Parasaurolophus* sp. (Farke et al., 2013) reported is a juvenile and the vestibular canals don’t necessarily represent the adult form. The shorter ASCs in the hadrosauriforms are consistent with the findings of Georgi, Sipla & Forster (2013), who found closer relative ASC and PSC sizes in the hadrosaurids, *Corythosaurus casuarius* and *Edmontosaurus regalis*, than in facultative and obligatory dinosaur bipeds, based on their estimates of semicircular canal area to head mass. According to Georgi, Sipla & Forster (2013), the neurosensory systems of these hadrosaurids were adapted to quadrupedal locomotion. The vertical canals in the obligatory ornithischian quadrupeds, *Pawpawsaurus campbelli* (1:1), an ankylosaur and the large-bodied ceratopsian, *Triceratops* sp. (1.05:1), are similar in height (Fig. 60).

Based on the inference of facultative bipedality from the vertical canal proportions, *Muttaburrasaurus langdoni* would have been among the largest facultatively bipedal ornithischians known from body fossils, if not the largest. This finding suggests a corollary to the trackways of large-bodied ornithopod ichnogenera, *Amblydactylus* and *Walmadanyichnus*, from the Broom Sandstone in Western Australia, whose trackways lack manual impressions (Romilio & Salisbury, 2011; Romilio & Salisbury, 2014; Salisbury et al., 2017). Although none of the Australian dinosaur trackways attributed to large-bodied ornithopods can be considered as pertaining to *Muttaburrasaurus langdoni*, they coincide with the predominantly bipedal mode of locomotion we infer for the taxon. Notably, trackway ichnogenera attributed to hadrosauriods and less derived styracosternans (*Caririchnium*, *Iguanodontipus*, *Hadrosauropodus*), indicate manus impressions of quadrupedal locomotion at slow and fast speeds (Diedrich, 2004; Kim, Lockley & Chun, 2016; Lockley, Nadon & Currie, 2003; Xing et al., 2015). Thus, the locomotory behaviour of *Muttaburrasaurus langdoni* and other large-bodied Australian ornithopods represented by the ichnogenera, appear to differ from their Laurasian cousins. Quadruped posture and locomotion in *Muttaburrasaurus langdoni* were likely adopted for slow movement activities, such as low-level browsing and standing at rest, while bipedal locomotion, as inferred, was used for walking and running.

The angle of the lateral semicircular canal (LSC) relative to the horizon has been considered an indicator of the alert/rest head orientation in dinosaurs and other archosaurs (Sakagami & Kawabe, 2020; Witmer et al., 2003; Witmer & Ridgely, 2009; but see Marugán-Lobón, Chiappe & Farke, 2013). However, the study by Hullar (2006) reported that the stable head position in mammals and birds is when the plane of the LSC is directed dorsally upwards by up to 15°. This angle of LSC tilt relative to the horizon, was further suggested by Hullar (2006) in the theropod *Allosaurus fragilis* and was considered a relatively constant finding across bilaterally symmetrical vertebrates. The LSCs in *Muttaburrasaurus langdoni* are roughly parallel to the skull roof (Fig. 57B). Thus, the alert/rest position of the *Muttaburrasaurus langdoni* head could have been anywhere from horizontal to tilted upwards, up to 15°.

*Hearing capacity*—The basilar papilla within the lagena is the membranous endolymph-filled duct that receives vibrations from the membrane of the *fenestra vestibuli* (Fig. 55) transferred through the surrounding perilymphatic duct system (Manley, 2011; Romer, 1955). Sensory hair cell afferents imbedded in the wall of the basilar papilla transduce sound vibrations into electrosensory signals to the brain (Manley, 2011). In early-diverging Archosauria, increased elongation of the lagena and, thus, the perilymph- and endolymph-filled ducts contained within, gave increased hearing sensitivity to middle and high-frequency sound in the >1 kHz range, departing from the lower frequencies that were detectable in less-derived archosauromorphs and other Reptilia (see Hanson et al., 2021; Manley, 2000; Walsh et al., 2009). Higher frequency hearing sensitivity through increased lagena/cochlea length in archosaurs and mammals has been associated with increased eco-behavioural characteristics, such as vocal communication complexity, conspecific social interactions, parental care, prey localization and predator avoidance (Choiniere et al., 2021; Hanson et al., 2021; Manley, 2000; Vergne, Pritz & Mathevon, 2009; Walsh et al., 2009). Hearing range in extinct amniotes has been previously assessed using audiogram-based, line-of-regression equations generated from hearing response tests in extant taxon subjects (Gleich, Dooling & Manley, 2005; Walsh et al., 2009; Barker et al., 2023). The estimated lengths of the endosseous cochlear duct (ECD) (Witmer et al., 2008) and basilar papilla have been used as proxies for hearing frequency range and sensitivity in extinct archosaurs, including in non-avialan dinosaurs where assessments from audiograms are, of course, not possible (Barker et al., 2023; Gleich, Dooling & Manley, 2005; Hanson et al., 2021; Walsh et al., 2009; Witmer et al., 2008). Owing to the vast size differences between non-avialan dinosaurs, line-of-regression equations have used the scaling factors of either estimated body mass (Gleich, Dooling & Manley, 2005) or basicranial length (Walsh et al., 2009; see also Barker et al., 2023). Basal papilla length, estimated as two thirds of total ECD length has also been used in a regression equation for the approximation of hearing range without the use of body or head scaling variables (Gleich, Dooling & Manley, 2005).

Walsh et al. (2009) calculated the mean best hearing range (MHR) and best hearing band width (BHR) for extinct taxa using ECD length divided by the scaling factor of basicranial length, with their quotients log transformed (see details in “Methods”). Using this method, the mean frequency of best hearing for the *Muttaburrasaurus langdoni* holotype is ∼1,162 Hz and the band of best hearing frequencies is centred at ∼1,741 Hz (<30 dB sensitivity level). Thus, the range of best hearing in *Muttaburrasaurus langdoni* is ∼291–2,032 Hz, with vocalization likely to have been propagated within this range (see “Vocalisation” above). The lowest and highest thresholds of hearing (*i.e.,* absolute hearing range) are not determined using this method, but would have been above and below best hearing, which in humans and other mammals, is determined by the lowest and highest frequencies detectable at 60 dB (Heffner & Heffner, 2007). The range of best hearing frequencies in *Muttaburrasaurus langdoni* is lower than assessed in extant and extinct birds (see Gleich, Dooling & Manley, 2005; Walsh et al., 2009), but within the low to middle frequency range (in mammals, low frequencies are in the range of ≤1.0 kHz and middle frequencies are in the range of 1.0–4.0 kHz; Quam et al., 2012). It is notable that Gleich, Dooling & Manley (2005) proposed a high-frequency hearing limit in other large-bodied non-avialan dinosaurs at, or below 3 kHz (see also: Barker et al., 2023; Hanson et al., 2021; Sakagami & Kawabe, 2020; Witmer et al., 2008). Hearing in *Muttaburrasaurus langdoni* was within this range. In amniotes in general, including in non-avialan dinosaurs, hearing range assessed from EDC length, can also infer the frequencies of phonation for vocalization (Russell & Bauer, 2021). To place hearing in *Muttaburrasaurus langdoni* in context, best hearing sensitivity in humans, within the 10 dB sensitivity range, is between 250 Hz to 8.1 kHz, with the level of best frequency around 3 kHz (Heffner & Heffner, 2007; Quam et al., 2012; Sivian & White, 1933). Conversational speech in humans occurs within this range (Quam et al., 2012). *Muttaburrasaurus langdoni* would have been sensitive to a frequency range comparable to conversational speech in humans. Phonation and vocalization in *Muttaburrasaurus langdoni* is likely to have occurred in the range of best hearing (*i.e.,* ∼291–2,032 Hz). *Muttaburrasaurus langdoni* potentially detected the lower frequencies of hearing for conspecific communication (*i.e.,* ≤ 1,000 Hz) in both closed and open habitats, particularly when visual cues were limited, and the higher frequencies of hearing with high vocal energy for near distance communication with conspecifics (see “Vocalisation” above).

### Visual fields in *Muttaburrasaurus langdoni*

The vision-linked behavioural characteristics of vertebrates that help to drive their ecological outcomes are controlled by the soft tissue components of their eyes, their visual fields and ultimately neurology and cognition (Knudsen, 2020; Land & Nilsson, 2012). Although the soft tissues of the eyes are not preserved in fossil vertebrates, the visual fields can be analysed from the orientation of their paired eyes in their osseous orbits if the cranium is adequately enough preserved or reconstructed (*e.g.*, Stevens, 2006). However, little quantitative assessments of the visual fields in fossil vertebrate taxa have been conducted. Differences in the monocular and binocular visual fields are integral to the vision-linked behaviour of vertebrates. The degree of the left and right monocular fields and binocular overlap of the paired monocular fields is controlled by the orientation of the optic axes of the eyes relative to the sagittal plane of the skull (Heesy, 2004; Land & Nilsson, 2012; Walls, 1963). The binocular field allows a 3D anterior/frontal percept, from within which the depth and shape of objects can be determined through steropsis (Nityananda & Read, 2017; Read, 2021; Stevens, 2006; Walls, 1963;Wilcox & Harris, 2010). Three-dimensional vision in the binocular field allows the distance of and timing to objects and targets, such as food items, conspecifics and obstacles, to be accurately judged (Nityananda & Read, 2017; Read, 2021; Stevens, 2006; Wilcox & Harris, 2010). A wide panoramic view in the monocular field allows ‘prey’ species, such as mammalian granivores and herbivores and many bird species, to scan for potential predators broadly across their surroundings (Land & Nilsson, 2012; Martin & Osorio, 2010)). A wide monocular field is further beneficial to conspecific grouping behaviour, such as flocking in birds, herding in mammalian herbivours and pack hunting in canids (Barber et al., 2020; Fernández-Juricic, Erichsen & Kacelnik, 2004; Hughes, 1977). A wide binocular field through greater convergence of the optic axes, gives a broader and clearer three-dimensional experience, but comes at the expense of the monocular field (Stevens, 2006). Thus, evolutionary tradeoffs occur in the relative degree of the monocular and binocular fields (Heesy, 2004; Martin, 2009; Martin & Osorio, 2010; Stevens, 2006). Here, using virtual perimetry and reconstruction of the head of the holotype, we quantitatively assess the visual fields of *Muttaburrasaurus langdoni* to help shed light on the visual ecology of the taxon.

**Figure 61:**
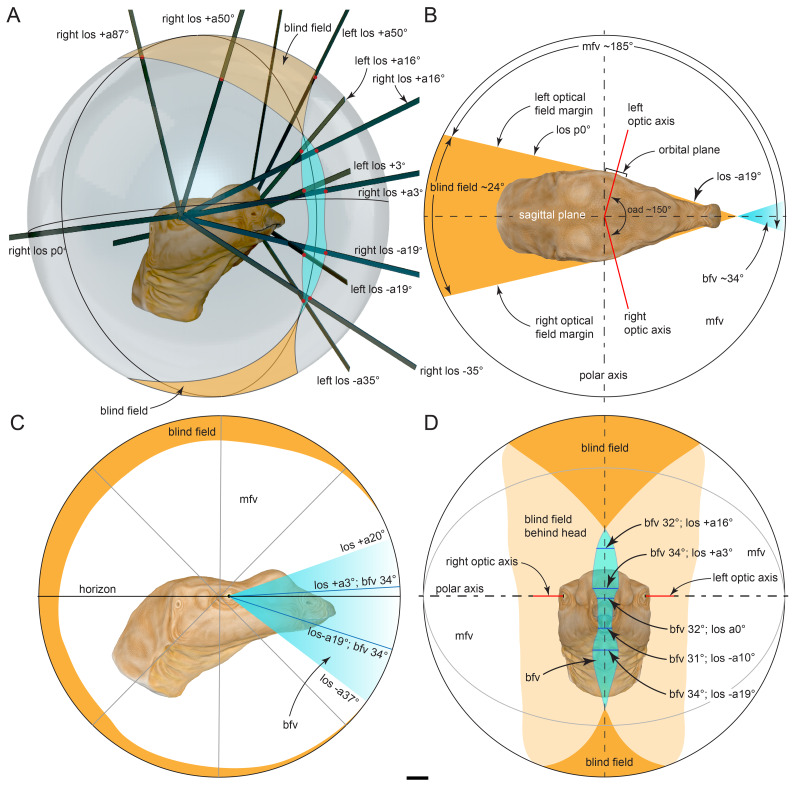
Virtual visual field perimetry for *Muttaburrasaurus langdoni* (QMF6140) from 3D life restoration of the head. (A) 3D perimetry arrangement for optical field margin measurements in ventro-anterolateral view, showing virtual sphere, lines of sight projections (6 pairs shown out of 18 in total) and optical field margins. (B) Optical field margins for widest monocular field in dorsal view. (C) Optical field margins in lateral (sagittal plane) view. (D) Optical field margins in anterior (transverse plane) view. Orange shaded area is blind field. White areas inside sphere are monocular fields. Blue shaded area is binocular field. Abbreviations: bfv, binocular field of view with amount of monocular field overlap (#) in degrees; los, line of sight above (+) or below (-) horizontal, anterior to (a) or posterior to (p) the polar axis; mfv, monocular field of view; oad, optic axes divergence. Scale bar equals 10 cm.

Eye diameter for the *Muttaburrasaurus langdoni* holotype was calculated at ∼78 mm. Viewed dorsally, divergence of the optic axes in *Muttaburrasaurus langdoni* is ∼150° (Fig. 61B). Interocular separation (=binocular disparity) is in the order of 370 mm. Binocular overlap occurs dorsoventrally in the region between +20° to −37° from the horizontal plane (Fig. 61C). Lateral obstruction to the anterior lines-of-sight caused by the lateral nasal bullae and the hypothesised extent of lateral expansion of the premaxillary rostrum, occurs approximately from the horizontal plane to −10° (Figs. 61C, 61D). The widest binocular overlap of ∼34° is measured at both +3° and −19° from the horizontal/dorsal plane, occurring immediately dorsal and ventral to the lateral nasal bullae and hypothesised lateral projections of the premaxillae (Figs. 61C, 61D). The total vertical extent of binocular overlap is ∼57°, with the binocular field biased towards the ground (Fig. 61C). The dorsal, ventral and posterior monocular margins are delimited by the fleshy circumorbital and brow margins, as reconstructed. Viewed dorsally (Fig. 61B), the greatest posterior extent of monocular vision is ∼91° from the optic axis. Viewed laterally, this posterior region of monocular vision occurs between 0° to −30° (Fig. 61C). Based on the posterior-most optic margin and the region of widest binocular overlap, the total monocular field of view for each eye is in the order of ∼185°. Thus, the greatest total field of view (= cyclopean field) is ∼336° (Fig. 61B). With head turning of ∼15° to the left and right, a vista of 360° could have been achieved. However, as previously noted, the actual monocular retinal field in *Muttaburrasaurus langdoni* is likely to have been lower than the total optic field assessed (*i.e.,* <185°), consistent with findings of the difference between the optic and retinal fields in birds, and particularly in the binocular field (*e.g.*, Martin, 2022; Martin & Katzir, 1999). Retinal binocular overlap could have been half the optic field measured and potentially closer to 16°. Nevertheless, the full binocular field could have been accessed through convergent mobility of the eyes (Land, 2015; Martin, 2009).

The analysis conducted here, although based on assumptions from the life reconstruction, confirms that vision in *Muttaburrasaurus langdoni* was lateral. *Muttaburrasaurus langdoni* had a wide monocular field and a narrow binocular field, as in lizards, turtles, crocodilians, most birds and mammalian herbivores (Fleishman, 2024; Hanggi & Ingersoll, 2012; Heesy, 2004; Heesy, 2009; Martin, 2007; Martin & Katzir, 1999; Mass & Supin, 2009; Nagloo et al., 2016; Read, 2021; Walls, 1963). Highest optical quality would have been attained in the monocular field at the zone around the fovea centralis (Land & Nilsson, 2012; Voss & Bischof, 2009; Read, 2021). Vision in the ‘unattended’ binocular field of *Muttaburrasaurus langdoni* (the retinal zone distant from the fovea centralis; Voss & Bischof, 2009) would have been peripheral, with lower optical quality, although binocular retinal light capture would have been doubled. However, if *Muttaburrasaurus langdoni* had extended photoreceptor cell density in the temporal retinal field, such as the retinal/foveat streak in some squamates, crocodilians, birds and equines (Coimbra, Collin & Hart, 2014; Fleishman, 2024; Hanggi & Ingersoll, 2012; Land, 2015; Nagloo et al., 2016; Voss & Bischof, 2009), higher optical quality could have been experienced in the peripheral binocular field, although not to the degree around the optic axis in the monocular field (Read, 2021).

Stereoscopic vision in the binocular field of *Muttaburrasaurus langdoni*, although narrow (Fig. 61), would have fulfilled the visual requirements of maintaining target direction, obstacle avoidance and neurological judgment of the distance and timing to targets, such as water, food items and conspecifics during forward locomotion (based on information in Land & Nilsson, 2012; Martin, 2009; Martin, 2014; Martin, 2022; Martin & Katzir, 1999; Martin & Osorio, 2010; Read, 2021). *Muttaburrasaurus langdoni* potentially experienced a pronounced 3D percept of objects and their relative positions through stereopsis in the binocular field (see Read, 2021) resulting from the transversely broad interocular separation of ∼370mm on the holotype. Broad interocular separation, combined with lens accommodation in the binocular field, potentially aided the selection, manipulation and prehension of food items (Martin & Osorio, 2010; Read, 2021) and could have also aided the resolution of distant object (Stevens, 2006; Read, 2021). Bias of the binocular field towards the ground potentially aided obstacle avoidance during locomotion, and the binocular field of ∼32° at 16° above the horizon (Fig. 61C) could have aided food identification and selection during high browsing.

The panoramic monocular field in *Muttaburrasaurus langdoni* potentially allowed predator detection, object discrimination, conspecific awareness and grouping. Birds can selectively gaze with one eye on a target of interest in the monocular field through head turning, while momentarily blocking processing of the visual information from the other eye (Voss & Bischof, 2009). The eye with fixed gaze is known as the ‘attended’ eye (Voss & Bischof, 2009). Gazing with the attended eye aims the zone of highest visual acuity on the optic axis towards the target. Switching between the attending eyes in birds appears to be under neurological, target priority control (Voss & Bischof, 2009). Head turning also occurs in lizards and equines with broad monocular fields (Fleishman, 2024; Hanggi & Ingersoll, 2012) and it seems reasonable to assume that *Muttaburrasaurus langdoni* would have used head turning to gaze with the attending eye on targets with priority (predators, conspecifics, food items).

In the visual landscape of *Muttaburrasaurus langdoni*, a sense of movement among objects, such as plants, trees, terrain, paths and other animals, was likely experienced through optic flow (= flow-field) in the monocular and binocular fields during forward locomotion (Land & Nilsson, 2012; Martin, 2009; Read, 2021). Optic flow is the percept of objects expanding as the individual approaches during forward locomotion. During locomotion, guidance to a target in the binocular field, with gaze fixed, would have been experienced through the symmetrical expansion of objects in the optic flow-field as they were approached (Land & Nilsson, 2012; Martin, 2009; Martin & Osorio, 2010; Read, 2021). In addition, the relative distance of objects near and far from an individual of *Muttaburrasaurus langdoni* could have been judged during locomotion in the monocular field through motion parallax—the perception that objects closer to the head are being approached faster than objects more distant, which appear to be moving in the same direction (Lazareva, 2021; Martin & Osorio, 2010). Pictorial cues in the broad monocular field, such as shading, perspective and light strength, potentially gave *Muttaburrasaurus langdoni* the percept of landscape depth and the distance to and between objects (based on information in Cavoto & Cook, 2006; Nityananda & Read, 2017).

The large eye diameter in *Muttaburrasaurus langdoni* (∼78 mm) agrees with the work of Lautenschlager et al. (2024), who calculated large eye sizes in all herbivorous dinosaurs, and among dinosaurs, the greatest diameters in megaherbivorous ornithopods. Although large eye diameters in extant vertebrates have been linked to heightened visual performance (visual acuity and low light sensitivity) under low light conditions (Warrant, 2004), Lautenschlager et al. (2024) pointed out that these visual performance characteristics are also under the control of the soft tissue components of aperture (iris, pupil) and lens size, as well as focal length (*i.e.,* eye depth) (Warrant, 2004), not preserved in the fossil remains of dinosaurs. As a result, Lautenschlager et al. (2024) considered that activity patterns in dinosaurs could not be categorically determined by eye diameter alone. In an earlier study, Schmitz & Motani, 2011, assessed activity patterns in non-avialan dinosaurs using scleral ring data for extant vertebrates as a proxy for eye shape and aperture size. However, according to Schmitz & Motani (2011), the eyes of dinosaur megaherbivores, together with assessments of their thermoregulatory constraints and energetic requirements (an expectation of the foraging period exceeding 12 hours/day), suggested a mesopic activity pattern (selective day/night activity). Scleral rings were not preserved in the *Muttaburrasaurus langdoni* holotype or *M*. sp. (QMF14921). However, large eye size and the mesopic activity pattern proposed in other megaherbivorous ornithopods, suggest that activity in *Muttaburrasaurus* was potentially mesopic.

The overall milieux of visual cues from objects and their placement (*i.e.,* landmarks), topography, light and shade and ‘compass’ information (*e.g.*, sun angle, stars) potentially aided cognitive mapping in *Muttaburrasaurus langdoni*, as in other vertebrates, for memory-based navigation (based on information in Breed & Moore, 2016; De Guinea et al., 2021). Navigation through visual cues in *Muttaburrasaurus* was potentially augmented by olfactory cues (see “Airway anatomy and function”), as suggested in hadrosaurids (Lautenschlager et al., 2024). Auditory cues could also have assisted conspecific awareness and contact when vision was inhibited by obstacles or limited by poor light (see also “Head posture, auditory capacity, balance and locomotion” and “Vocalisation”, above).

### Cranial kinematics and mastication

Early work on the dentition of *Muttaburrasaurus langdoni* proposed that the occlusal surfaces of the upper and lower cheek teeth formed an acute angle of contact (Bartholomai & Molnar, 1981; Molnar, 1995), consistent with dentition primarily for shearing plant material, as in ceratopsians (based on Ostrom, 1964). This early assessment of oral processing in *Muttaburrasaurus langdoni* contrasted with the notable characteristic of grinding mastication assessed in all other ornithopods (Norman & Weishampel, 1985; Norman & Weishampel, 1991; Weishampel, 1983, Weishampel, 1984). However, our CT imagery now show that the occlusal surfaces of the cheek teeth in *Muttaburrasaurus langdoni* are blunt angled (∼83° on the maxillary teeth and ∼69° on the dentary teeth relative to their labial and lingual faces, respectively; Figs. 25C–25E). The bluntly angled occlusal surfaces of the cheek teeth is consistent with grinding mastication involving orthal (dorsally directed occlusion) and long axis rotation of the mandibular corpus during mandibular adduction (powerstroke), as assessed in other ornithopods (Bell, Snively & Shychoski, 2009; Nabavizadeh & Weishampel, 2016). In addition to orthal occlusion of the cheek and hemimandibular rotation of the mandibular corpus, circular grinding mastication in ornithopods is understood to result from the addition of kinetic palinal (posterior directed) occlusion, enabled by streptostylic (anteroposterior) motion of the quadrate at the otic joint between the quadrate head and squamosal (Nabavizadeh, 2020a; Nabavizadeh & Weishampel, 2016; Norman & Weishampel, 1985; Norman & Weishampel, 1991; Weishampel, 1983; Weishampel, 1984; Wilken et al., 2020). The occurrence of palinal occlusion in derived ornithopods has been quantitatively supported from analyses of dental microwear (Nabavizadeh & Weishampel, 2016; Varriale, 2016; Virag & Osi, 2017; Williams, Barrett & Purnell, 2009).

In ornithopods the occluding edges of the cheek teeth sliding transversely over the obliquely sloping occlusal surfaces (lingually on the maxillary teeth and labially on the dentary teeth) would have acted like bolt-cutters that sheared through tough plant material as pressure was applied during orthal closure of the jaws (based on information in Nabavizadeh, 2014; Nabavizadeh & Weishampel, 2016; Weishampel, 1984). During mandibular adduction, sliding orthal contact required transverse mobility of the dentary teeth relative to the maxillary teeth. Notably, transverse occlusive pressure is generally accepted to have resulted from long axis rotation of the mandibular corpus (Nabavizadeh, 2014; Nabavizadeh, 2020a; Nabavizadeh, 2020b; Nabavizadeh & Weishampel, 2016; Varriale, 2016; Virag & Osi, 2017), rather than rotation of the maxillae originally proposed under the model of pleurokinesis (Weishampel, 1984). Under the pleurokinetic model, axial rotation of the maxillae required hinged joints with the premaxilla and the lacrimal, or between the lacrimal and prefrontal, and between the palatine and pterygoid (Weishampel, 1983; Weishampel, 1984). However, most workers now conclude that the maxilla in ornithopods was part of the akinetic skull (see Cuthbertson et al., 2012; Holliday & Witmer, 2008; Virag & Osi, 2017; and authors within). Furthermore, three-dimensional modelling by Rybczynski et al. (2008) of the hypothesised pleurokinetic-hinge complex in *Edmontosaurus regalis*, found that many of the joints between the hinged units required under the model opened to unnaturally excessive degrees. Examination of the maxilla of *Muttaburrasaurus langdoni* show that the maxilla is rigidly attached to the akinetic unit of the cranial roof and neurocranium through firm connections with the nasal and lacrimal, with the latter firmly abutting the prefrontal. These observations confirm that pleurokinesis in *Muttaburrasaurus langdoni* involving mobility of the maxilla was unlikely.

Orthal occulsion of the cheek teeth in *Muttaburrasaurus langdoni*, as in all other sauropsids, was isognathous (symmetrically even jaw closure), as opposed to anisognathous (asymmetrical jaw closure), which only occurs in mammalian herbivores uniquely under the control of their masseter musculature (Nabavizadeh, 2020a; Nabavizadeh, 2020b; Nabavizadeh & Weishampel, 2016; Norman & Weishampel, 1985; Norman & Weishampel, 1991). Axial rotation of the mandibular corpus in ornithischians was likely facilitated by ligamentous flexibility at the symphyseal joint between the unfused dentaries (Nabavizadeh, 2014; Nabavizadeh & Weishampel, 2016). Although the symphyseal regions of the dentaries are not preserved on the holotype, a flexible symphyseal margin parsimoniously agrees with that assessment, given that the dentaries of all ornithischians, where known, are unfused, except for derived Ceratopsia, where the dentaries are fused to the predentary (Nabavizadeh & Weishampel, 2016). Hemimandibular rotation of the mandibular corpus in *Muttaburrasaurus langdoni* was likely constrained by the wing-like lateral processes of the predentary, as proposed in all other ornithopods (Nabavizadeh & Weishampel, 2016) (noting that only a fragment of the lateral process of the left predentary is preserved on the holotype; Fig. 43G) and would have been under the control of the three muscle bodies of the *m. adductor mandibulae externus* (*m. adductor mandibulae externus medialis*, *m. a. m. e. profundus*, *m. a. m. e. superficialis*), as well as the *m. pseudotemporalis*, all of which originated in the temporal fossa and extended anteroventrally through the subjugal opening (based on information in Holliday, 2009; Nabavizadeh, 2020a, Nabavizadeh, 2020b; Nabavizadeh & Weishampel, 2016) (for schematic placement of these muscle bodies, see Fig. S4). Where the *m. adductor mandibulae externus medialis* and *m. a. m. e. profundus* and *m. pseudotemporalis* in ornithischians attached to the dorsal margins of the coronoid and surangular (Holliday, 2009; Nabavizadeh, 2020a; Nabavizadeh, 2020b), Nabavizadeh (2020a) and Nabavizadeh (2020b) proposed that the *m. adductor mandibulae externus superficialis* attached to the buccal ridge of the dentary with the anterior extent of attachment constrained by the subjugal opening. According to Nabavizadeh (2020a) and Nabavizadeh (2020b), the *m. adductor mandibulae externus superficialis* in ceratopsians attached along much of the length of the buccal ridge of the dentary. In hadrosaurids, however, attachment was limited to the posterior zone of the buccal ridge (Nabavizadeh, 2020a; Nabavizadeh, 2020b). As in hadrosaurids, attachment of the *m. adductor mandibulae externus superficialis* in *Muttaburrasaurus langdoni* would have been restricted to the posterior part of the buccal ridge by the subjugal opening (Fig. S4; see also in “Comments on the feeding behaviour of *Muttaburrasaurus langdoni*” below)*.* Stabilization of the mandible against significant torsional forces during adduction and rotation was potentially controlled by the *M. pterygoideus ventralis*, which originates on the pterygoid and inserts medially on the surangular (see Fig. S4) (based on Bell, Snively & Shychoski, 2009; Holliday, 2009; Nabavizadeh, 2020a; Nabavizadeh, 2020b; Rybczynski et al., 2008). In addition, the *M. pterygoideus ventralis* potentially provided counter rotation during abduction (Bell, Snively & Shychoski, 2009).

Palinal occlusion of the cheek teeth in *Muttaburrasaurus langdoni* would have required anteroposterior mobility of the mandibular corpora through streptostylic (anteroposterior) rotation of the quadrate at the otic (quadrato-squamosal) joint, as hypothesised in other ornithopods (Mallon & Anderson, 2013; Nabavizadeh & Weishampel, 2016; Norman & Weishampel, 1985; Norman & Weishampel, 1991; Varriale, 2016; Weishampel, 1984). Mobility of the quadrate would have further required syndesmotic (sliding) joints with the cheek and palatal regions. A syndesmotic joint with the jugal was possible *via* the quadratojugal. Although the left quadrate on the holotype was displaced dorsally and laterally from the left pterygoid (Fig. 30G). These two bones would have closely articulated along roughly planar mating alae. Although the mating surfaces of the left pterygoid and ectopterygoid are displaced (Fig. 23B; restoration shown in Fig. 23A), a syndesmotic joint between these two bones is possible. The obliquely angled orientation of the mating faces between these two bones, further suggests that streptostylic rotation of the quadrate, if it had occurred, would have been posterolateral-anteromedial directed. Mobility of the pterygoid in the same plane would have been dependent on its ability to move against the adjoining basipterygoid process of the parabasisphenoid (*i.e.,* neurokinesis; see Weishampel, 1984). The rounded distal ends of the basipterygoid processes on the holotype, loosely locate in fossae on the pterygoid and, in ventral view, the basipterygoid processes are obliquely oriented approximately in the direction of the quadrate (Figs. 30E, 30K, 33B, 34). It is apparent from the joint between the pterygoids and basipterygoid processes that mobility of the quadrate would have not been restricted, while maintaining linkage to the neurocranium. From our assessment of the joints in the suspensorium, mobility of the quadrate in *Muttaburrasaurus langdoni* (as the columnar element of the suspensorium) was possible, consistent with cranial kinesis in sauropsids (Wilken et al., 2020). Occlusion of the cheek teeth in *Muttaburrasaurus langdoni* was transverse-isognathous (Bell, Snively & Shychoski, 2009) and features of the mandible and suspensorium suggest that orthopalinal occlusion was possible. Determination of palinal occlusion of the cheek teeth in *Muttaburrasaurus langdoni* would be better supported by an analysis of tooth microwear, as conducted on other ornithopods (*e.g.*, Mallon & Anderson, 2013; Varriale, 2016; Virag & Osi, 2017; Williams, Barrett & Purnell, 2009). However, such a study is not possible on the dental materials of *Muttaburrasaurus* presently known.

### Comments on the feeding behaviour of *Muttaburrasaurus langdoni*

Comparisons have been previously drawn on the feeding preferences between broad and narrow muzzled megaherbivorous dinosaurs (Carrano, Janis & Sepkoski, 1999; Mallon & Anderson, 2013; Whitlock, 2011), with some inferences guided by information from extant mammalian macropodoids and ungulates (*e.g.*, Janis, 1995; Janis, 1970; Janis & Ehrhardt, 1988). Notably, extant, narrow-muzzled, mammalian herbivores show a preference for selective browsing in stand vegetation (*sensu* Janis & Ehrhardt (1988). Among the hadrosaurids, Carrano, Janis & Sepkoski (1999) proposed that broader muzzled saurolophines were adapted for less-selective feeding/browsing on harder plant materials in open habitats, while narrower muzzled lambeosaurines undertook more selective feeding/browsing on succulent plants in closed forest habitats. An ecomorphological analysis by Mallon & Anderson (2013), suggested niche partitioning occurred between co-occurring ankylosaurian, ceratopsian and hadrosaurid megafaunas on the Late Cretaceous island continent of Laramidia, partly based on disparity between the forms of their muzzles and rostra. The broad bill-like form of the saurolophine and lambeosaurine muzzles, consistent with a grazing strategy on leafy plant materials, differed from the narrow-pointed beak of ceratopsians, who potentially browsed on low-level, woody herbaceous plants, such as early angiosperms.

Muzzle form and dentition in *Muttaburrasaurus langdoni* allow some insight into the feeding style of the taxon. Although the anterior-most ends of the paired premaxillae and the predentary on the holotype are missing, the morphology of the preserved regions of the premaxillae and left maxilla indicate a narrow dentulous rostrum, as in early diverging ornithischians (Fig. 6; see life restoration, Fig. 56). Based on information in Nabavizadeh & Weishampel (2016), a dentulous premaxilla suggests that the predentary of *Muttaburrasaurus langdoni* was likely to have been narrow and pointed. The narrow dentulous rostrum of *Muttaburrasaurus langdoni* differs from the broad predominantly edentulous, bill-like form across Iguanodontia, exemplified by the hadrosaurids (Norman, 2004; Horner, Weishampel & Forster, 2004), and is consistent with a feeding strategy that involved selective browsing (Carrano, Janis & Sepkoski, 1999; Mallon & Anderson, 2013; Nabavizadeh & Weishampel, 2016; Whitlock, 2011).

The narrow dentulous rostrum of *Muttaburrasaurus langdoni* potentially allowed selective browsing on woody plant material, as suggested for ceratopsians (Mallon & Anderson, 2013). Cretaceous angiosperms, identified in the Eromanga Basin (Dettmann, Clifford & Peters, 2009), could have supplied a source of woody plant browse for *Muttaburrasaurus langdoni*. However, unlike the low arboreal feeding envelope of ceratopsians, *Muttaburrasaurus langdoni* could have browsed at higher levels. With an orbit height of 3.2 m above the ground, estimated from the cast skeletal reconstruction of the holotype in a natural bipedal standing posture at the National Museum of Australia (Fig. S5), an upper feeding envelope of ∼4 m was possible, as in hadrosaurids (see Mallon & Anderson, 2013). The premaxillary teeth of *Muttaburrasaurus langdoni* potentially allowed the prehension of food items (see also Galton, 1973) and could have facilitated the stripping of hard fibrous outer layers of cones and pods to access to the seeds/nuts of plants, such as those of Araucariaceae and Bennettitales and possibly the fruits of Cycadophyta and Podocarpaceae, all of which were diverse forest plants in Australia during the Cretaceous (Dettmann et al., 1992; Kershaw & Wagstaff, 2001). The grinding cheek teeth and potentially strong bite force in *Muttaburrasaurus langdoni*, as indicated by the large size of the mandibular adductor muscle chambers (see also Molnar, 1995), suggest that *M. langdoni* had the ability to masticate and comminute tough, fibrous plant materials (see “Cranial kinematics and mastication” above). Work by Chin, Feldmann & Tashman (2017) found evidence of crustacean remains in coprolites attributed to hadrosaurids, indicating partial carnivory in that group. According to Chin, Feldmann & Tashman (2017) hadrosaurids potentially acquired crustaceans by foraging in rotting logs. This discovery suggests that a component of the *Muttaburrasaurus langdoni* diet could have included carnivory on invertebrates. The narrow muzzle and premaxillary dentition of *Muttaburrasaurus langdoni* could have aided the prehension of invertebrates in rotting logs or in burrows along the intertidal zone. Decapods, for example, are known from marine strata of the epeiric Eromanga Sea (Jell & Cook, 2025; and references within).

The occurrence of two skeletons of *Muttaburrasaurus* spp. (QMF6140, QMF14921), and possibly a third (QMF12541), in strata of the epeiric Eromanga Sea, as well as three isolated teeth (AMF81565, QMF14420, QMF14421) referred to the genus from freshwater lagoon strata opening to the Eromanga Sea (Bartholomai & Molnar, 1981; Bell et al., 2019b; Molnar, 1996), suggests that *Muttaburrasaurus* lived on the coastal plain close to the shoreline, intertidal zones or near brackish-water lagoons and estuaries. From these zones, carcasses of *Muttaburrasaurus* could have washed into the Eromanga Sea during storm events. The suggested possession of extra-renal, cranial salt glands in *Muttaburrasaurus langdoni* is consistent with the need to excrete excess sodium chloride ingested in these habitats. In a near coastal habitat, excess salt could have been ingested with food items, such as from depositional coatings on plants, from halophytes (salt tolerant plants) (*e.g.*, Meng, Zhou & Sui, 2018), from macro-algae, which can occur over varying salinity gradients (Larsen & Sand-Jensen, 2006) and possibly from marine invertebrates, if they had been a dietary preference. Given these possibilities, the dietary preferences of *Muttaburrasaurus langdoni* will only be understood with congruent data, such as from cololites or coprolites associated with skeletal remains (*e.g.*, Brown et al., 2020; Chin, Feldmann & Tashman, 2017; Poropat et al., 2025). However, given the extreme rarity of *Muttaburrasaurus* spp. in the fossil record with no fossil remains known from terrestrial deposits, the future discovery of cololites and particularly coprolites is difficult to envisage. Future analysis of stable strontium isotopes with a marine signature in tooth enamel (^87^Sr/^86^Sr) could provide a means to test for the dietary intake of sea salt, if diagenetic alteration can be accounted for (see further under “Extrarenal salt excretion?” above).

### Extraoral soft-tissue in *Muttaburrasaurus langdoni* ?

The deeply inset and sloping maxillary and dentary buccal margins on the skull of *Muttaburrasaurus langdoni* suggests that food items, if forced labially from the cheek teeth during comminution, would have been expelled from the mouth if extraoral tissue had not been present for containment. This expectation of food loss during oral processing in ornithischians if ‘cheeks’ had not been present, led some previous workers to argue for their presence (Galton, 1973; Norman & Weishampel, 1985; Sereno, 2012). However, the development of extraoral soft-tissue (‘cheeks’) over the oral cavity in ornithischians has been regularly debated (for detailed synopses, see Galton, 1973; Knoll, 2008; Morhardt, 2009; Nabavizadeh, 2020b; Witmer, 1995). Galton (1973) suggested that ‘cheeks’ in ornithischians could have been formed by musculature like, but not homologous to, the *m. buccinatoris* in mammals. Galton (1973) additionally posited that novel musculature for the ‘cheeks’ in ornithischians could have been innervated by the maxillary branch of the trigeminal nerve (cn V). The strongest argument against the presence of cheeks in Ornithischia has been attributed to extant phylogenetic bracketing (Nabavizadeh, 2020b), as elaborated by Witmer (1995). Muscular cheeks are not developed in crocodilians and most extant birds and thus, most parsimoniously, were unlikely to have occurred in non-avialan dinosaurs. Furthermore, muscular cheeks or lips, analogous to the tissues in mammals, are not developed in extant sauropsids (Nabavizadeh, 2020b; and authors within). According to Nabavizadeh (2020b), p. 350), extant phylogenetic bracketing infers that the development of ‘cheeks’ in ornithischians would have required an “unlikely degree of differentiation in muscular attachment sites and reorientation of muscle fibres”. Nevertheless, pseudomasseter musculature (*m. pseudomassiter*) uniquely evolved in Psittaciformes (parrots) (Faillace et al., 2025; Tokita, 2004)—morphology that led Sereno (2012) to speculate that similar musculature could have developed in Psittacosauridae, as well as Heterodontosauridae.

A study by Morhardt (2009) quantified the densities of neurovascular foramina on the premaxillae, maxillae and dentaries of extant amniotes in terms of “bare” (little superficial covering of teeth and gums, as in crocodilians), “epidermal tooth cover” (as in the ‘lips’ of extant lizards and snakes), “cornified beak” (as in the keratinised rhamphotheca of birds and turtles) and “muscular cheek” (as in mammals) and then compared these rankings among ornithischian and non-avialan saurischians. Morhardt (2009) found statistical support for either epidermal tooth covering (lips) or muscular cheeks across non-avialan dinosaurs. Notably, these two ranks correlate with foramina densities for the premaxilla, maxilla or dentary of <50. *Muttaburrasaurus langdoni* falls within this group (∼23 neurovascular foramina on the left maxilla and ∼3 neurovascular foramina on the observable region of the complete left dentary). Most recently, Cullen et al. (2023) proposed that extraoral lips were developed in large-bodied theropods, differing from earlier assumptions that the oral margins in this group would have been ‘bare’ as in crocodilians.

Accepting that extraoral soft-tissue in the form of lips had occurred in non-avialan saurischians, the deeply inset dentition and transversely broad buccal shelves that overhang the dentition on the maxillae and dentaries are uniquely ornithischian (see also Galton, 1973). A deep buccal fossa is particularly developed in derived ornithischians in Thyreophora, Ceratopsia and in all Ornithopoda (Galton, 1973; Nabavizadeh, 2020b), as exemplified by *Muttaburrasaurus langdoni*, and indicates a functional relationship with feeding not experienced by saurischians or other sauropsids. Transverse-isognathous mastication in ornithopods suggests an additional need for lateral food retention, perhaps not experienced by dentition configured for slicing in early diverging ornithischians, such as *Lesothosaurus diagnosticus* (see Knoll, 2008). If extraoral cheeks had developed in ornithischians as a means of retaining food during comminution, as would seem necessary, their presence has not been categorically supported. However, it is difficult to envisage the development of lips, as in Theropoda (Cullen et al., 2023), having been developed on the steeply sloping buccal shelves of the maxillae and dentaries of ornithopods, such as *Muttaburrasaurus langdoni*. Likewise, the development of lips aligned with the vertical plane between the buccal ridges of the maxillae and dentaries, seems unreasonably distant from the dentition. Notably, in *Muttaburrasaurus langdoni*, the ventral surface of the jugal shelf and dental parapet laterally on the maxilla forms a deep fossa overhanging the dentition (Figs. 5A, 6A, 21). The development of lips in this location seems incongruous and a thicker tissue pack, such as a muscular cheek, seems more likely.

A study by Nabavizadeh (2020b) proposed that the *m. adductor mandibulae externus superficialis* in ornithischians could have attached to the buccal ridge on the lateral margin of the dentary (perhaps equivalent to ‘*pars superficialis*’ in Galton, 1973), differing from the attachment on the dorsolateral surface of the surangular proposed by Holliday (2009). According to Nabavizadeh (2020a) and Nabavizadeh (2020b), the subjugal opening, through which the adductor musculature passed, controlled the anterior extent of the *m. adductor mandibulae externus superficialis*. Nabavizadeh (2020b) further proposed that the attachment of the *m. adductor mandibulae externus superficialis* in ceratopsians extended along most of the dentary, which effectively would have formed a fleshy, extraoral cheek. However, Nabavizadeh (2020b) showed that the *m. adductor mandibulae externus superficialis* in hadrosaurids was restricted to the posterior end of the dentary; thus, failing to form the anteriorly extended extraoral wall he had proposed in derived Ceratopsia. As in hadrosaurids, the *m. adductor mandibulae externus superficialis* in *Muttaburrasaurus langdoni* would have been restricted to the posterior end of the dentary (Fig. 5S). Therefore, if an extraoral cheek had been developed in *Muttaburrasaurus langdoni*, and other ornithopods, it can only have been formed by uniquely derived musculature, comparable to the *m. pseudomassiter* of Psittaciformes. Thus, departing from strict adherence to phylogenetic bracketing, a separate evolutionary episode of musculature evolution dedicated to extraoral function in Ornithischia is hypothesised. However, testing this hypothesis will require further investigation. Future examination of the cortical structure in the buccal shelf and ridge regions of maxillae and dentaries for the presence of Sharpey’s fibres (*e.g.*, Bell, Snively & Shychoski, 2009; Petermann & Sander, 2013) could provide an additional means to test the occurrence of extraoral musculature in ornithischians. The debate over the presence of cheeks in ornithischians might be more conclusively resolved when mummified skin impressions in the buccal region are discovered in the future—a possibility demonstrated by several recent discoveries (Bell et al., 2014; Sereno et al., 2025). Extraoral soft-tissue in *Muttaburrasaurus langdoni* is reflected in the fleshed-out reconstruction of the head (Fig. 56).

### Comments on the phylogenetic placement of *Muttaburrasaurus langdoni* in Rhabdodontomorpha

A formal phylogenetic analysis of *Muttaburrasaurus langdoni* was not undertaken here. As new postcranial data for the holotype will be presented in future work, phylogenetic analyses will be better informed when these data have been presented. However, as *Muttaburrasaurus langdoni* has been recovered in several recent phylogenetic analyses as an early diverging rhabdodontomorph (Barta & Norell, 2021; Coria et al., 2025; Dieudonné et al., 2016; Madzia, Boyd & Mazuch, 2018; McDonald, 2011; McDonald et al., 2010b; Zanno et al., 2023), but not in all analyses (Fonseca et al., 2024b; Herne et al., 2019; Madzia, Jagt & Mulder, 2020; Rozadilla, Agnolín & Novas, 2019), the following craniodental comparisons extending from this study are relevant to future phylogenetic analyses.

The cheek teeth of *Muttaburrasaurus langdoni* clearly differ from those of the rhabdodontomorphs in the six following aspects. (1) The maxillary crowns lack the distinct step from the paracingular fossae onto the cingulum (ectoloph; = elevated rim; Virag & Osi, 2017; Chanthasit, 2010), present in the rhabdodontomorphs and *Tenontosaurus tilletti* (Thomas, 2015), which has been recovered as a rhabdodontomorph in some recent analyses (*e.g.*, Zanno et al., 2023). An ectoloph is also absent on the crowns of *Galleonosaurus dorisae*, *Leaellynasaura amicagraphica* (Herne et al., 2019), *Gasparinisaura cincosaltensis* (MUCPv-208; M. Herne, pers. obs., 2008) and *Talenkauen saltacrucensis* (based on Cambiaso, 2007), Fig. 17), which have been variously recovered as elasmarians (Fonseca et al., 2024b; Herne et al., 2019)). (2) The labial surfaces of the maxillary tooth crowns of rhabdodontomorphs lack a primary ridge (see Virág & Ősi, 2017; Weishampel et al., 2003), although a protrusive central ridge is apparent on the crowns of *Zalmoxes robustus* Weishampel et al., 2003 (BMNH R.4901 and BMNH R.3395; M. Herne, pers. obs., 2009) and reported in *Iani smithi* Zanno et al., 2023. Differing from the rhabdodontomorphs, a primary ridge occurs on the maxillary crowns of the *Muttaburrasaurus langdoni* holotype and *M.* sp. (QMF14921), although more strongly developed on the latter (Herne et al., 2019). In addition, the secondary ridges are convergent with the primary ridge on the *Muttaburrasaurus* maxillary crowns, as in elasmarians (Herne et al., 2019). Differing from *Muttaburrasaurus langdoni*, the apicobasal ridges lack convergence with the prominent central ridge on the rhabdodontomorph crowns. (3) The primary ridge on the maxillary crowns of *Muttaburrasaurus langdoni* and *M.* sp. (QMF14921) is strongly offset distally, as in elasmarians (Herne et al., 2019), as well as in *Camptosaurus dispar* (Galton, 2006). This feature is absent on the crowns of the rhabdodontomorphs, including *Tenontosaurus tilletti* (Thomas, 2015). (4) The secondary ridges labially on the maxillary and dentary crowns of the rhabdodontomorphs and *Tenontosaurus tilletti*, are separated by channels, whereas the secondary ridges and primary ridge are closely abutting on the crowns of *Muttaburrasaurus langdoni*, as in elasmarians (Herne et al., 2019). (5) The labial, mesial and distal margins of the *Zalmoxes robusta* crowns (BMNH R.4901, BMNH R.3395; M. Herne, pers. obs., 2009) are rolled forming lip-like edges. This feature is absent on the crowns of *Muttaburrasaurus langdoni* and *M.* sp. (QMF14921). (6) The mesial and distal margins on the maxillary and dentary crowns of the rhabdodontomorphs, *Mochlodon vorosi* (Virág & Ősi, 2017), *Zalmoxes robustus* Weishampel et al., 2003, *Rhabdodon* sp. (Chanthasit, 2010) and *Tenontosaurus tilletti* (Thomas, 2015), have unsupported marginal denticles. Unsupported denticles are absent on the cheek tooth crown margins of *Muttaburrasaurus langdoni* and *M.* sp. (QMF14921). In lacking a primary ridge and possessing channels between the secondary ridges, the cheek tooth crowns of *Hypsilophodon foxii* more closely resemble the crowns of *Tenontosaurus tilletti* and the rhabdodontomorphs than those of *Muttaburrasaurus langdoni* and the elasmarians. Apart from differences between the cheek tooth crowns of *Muttaburrasaurus* and the rhabdodontomorphs, the posttemporal fenestra of *Muttaburrasaurus langdoni* extends through the paroccipital process, as in *Hypsilophodon foxii* (Galton, 1974), differing from perforation of the squamosal, considered unique for the rhabdodontomorphs (*sensu* Zanno et al., 2023). Viewed dorsally, the lateral margin of the *Muttaburrasaurus langdoni* dentary is clearly concave, differing from the straight lateral margin in the rhabdodontomorphs (Zanno et al., 2023). The ascending process on the supraoccipital of *Muttaburrasaurus langdoni* lacks the anterodorsally directed slope on the supraoccipitals of rhabdodontomorphs and most other ornithopods. Modification of the premaxilla in *Muttaburrasaurus langdoni* to form a pseudonares and lateral displacement of the nasals, features that are convergent with lambeosaurines, are unknown in the rhabdodontomorphs. Finally, the paired prenasal ossifications in *Muttaburrasaurus langdoni*, whether they are novel neomorphic elements or highly unusual, additional processes of the premaxillae, are unknown in the rhabdodontomorphs, or any other ornithischian. The highly unusual anatomy of the *Muttaburrasaurus langdoni* and *M.* sp. (QMF14921) (Molnar, 1996) muzzle, suggests a cryptic lineage of Gondwanan Ornithopoda without any obvious progenitor at present.

### Geochronological age of *Muttaburrasaurus langdoni*

Previous chronostratigraphic understanding of the Mackunda Formation placed *Muttaburrasaurus langdoni* in the upper Early Cretaceous (Albian) (Cook, McKeller & Draper, 2013; Tucker et al., 2013; Tucker et al., 2016). Radiometric dating undertaken in this work from detrital zircons collected close to the holotype locality of *Muttaburrasaurus langdoni*, now constrain the oldest depositional age of the Mackunda Formation in the region of the holotype in the Cenomanian at a maximum likelihood age (MLA) of 96.3 ± 8.6 Ma. The age range of *Muttaburrasaurus* spp. extends across the boundary of the Early and Late Cretaceous, from the upper Albian of the Allaru Formation (Cook, McKeller & Draper, 2013; Molnar, 1996) to the lower Cenomanian of the Griman Creek Formation (see Bell et al., 2019b; Molnar, 1996) and mid-Cenomanian of the Mackunda Formation. Whether *Muttaburrasaurus* extended into the depositional age of the Winton Formation (<(MLA) 93.5 ± 1.2 Ma) is presently unknown (see under “Geochronology” above).

## Conclusions

Radiometric dating of U/Pb in detrital zircon places the *Muttaburrasaurus langdoni* holotype in the Cenomanian at (MLA) 96.3 ± 8.6 Ma. The *Muttaburrasaurus langdoni* skull presents a mosaic of plesiomorphic, convergent and unique morphological features. Differing from all other large-bodied ornithopods, *Muttaburrasaurus langdoni* possesses well-developed premaxillary dentition with five premaxillary teeth—the plesiomorphic condition for an ornithischian, and the largest ornithischian with premaxillary teeth. The dorsally inflated muzzle is formed from modified posterior processes of the premaxillae, which exclude the nasals from the nares, and novel, complex, paired ossifications, termed prenasals. The nasals form the posterior-most end and posterolateral sides of the dorsally inflated muzzle. Exclusion of the nasals from the nares by the premaxilla, forming pseudonares, and laterally divergent anterolateral processes of the nasals, are features convergent with lambeosaurines. The prenasals are either unique neomorphic bones or additional processes of the premaxillae. Absence of the anterior-most end of the premaxillae leaves this question open. However, neomorphic elements seem more likely, as three posterior processes on the premaxilla are unknown in any other amniote. The dorsally hypertrophied muzzle houses complex paired superior airways that are interpreted as having an olfactory rather than a respiratory function. Looping airflow through the superior airways potentially slowed inspired air, consistent with olfactory reception. Enlarged surfaces for olfaction and enlarged olfactory bulbs congruently support high olfactory acuity in *Muttaburrasaurus langdoni*, which potentially assisted with navigation, selective browsing, predator avoidance and group interactions. The suggested olfactory function of the superior airways is tempered by the lengthy distance between this region from the olfactory bulbs, which in other tetrapods, are closely located. Differing from all other ornithischians, contact between the pterygoid and palatine is absent. Instead, contact between the vomera and the basipterygoid bosses of the pterygoids suggest that the vomera, rather than the palatine processes of the pterygoids, supported the *oropharynx*.

A body mass of 8,854.0 kg ± 25% for the *Muttaburrasaurus langdoni* holotype is retrieved using the stylopodial-based formula for dinosaur bipeds (cQE). The cQE body mass is 938 kg higher than an earlier spline-based, volumetric estimate of 7,916.0 kg ±15% but overlaps within error. The body mass of *Muttaburrasaurus langdoni* is presently uncertain and would benefit from a future convex hull expansion estimate, when a full-body restoration is confidently supported. The narrow dentulous premaxillary rostrum suggests that *Muttaburrasaurus langdoni* was a selective browser. Premaxillary teeth could have assisted the prehension of and access to nuts and seeds, the removal of bark to access cambium and access to invertebrates, if they had been a dietary preference. The cheek teeth of *Muttaburrasaurus langdoni* were not replaced *en masse*, but in a wave-like, alternating tooth pattern, as in other ornithischians. The robust cranium, large adductor musculature and cheek teeth configured for transverse-isognathus and possibly palinal grinding mastication, suggest *Muttaburrasaurus langdoni* could have processed tough, fibrous plant material, such as angiosperm and gymnosperm leaves and seeds, fern fronds, bark and cambium and the fruits of cycads. *Muttaburrasaurus* potentially inhabited coastal plains where the collateral ingestion of excess sodium salt was possible. A laterally projecting nasal bulla on the holotype is suggestive of a nasal salt excretion gland that could have allowed the excretion of excess salt beyond the ability of the renal system.

*Muttaburrasaurus langdoni* had a wide monocular visual field (∼336°) and relatively narrow binocular overlap (up to 34°), consistent with megaherbivorous tetrapods and ‘prey’ species to enable the early detection of predators and aid conspecific grouping behaviour. Broad interocular separation and stereoscopic depth perception in the binocular field potentially assisted navigation and obstacle avoidance during forward locomotion and the prehension of food items at the muzzle. Reptile encephalisation quotient and cerebral mass suggest that cognitive ability in *Muttaburrasaurus langdoni* was comparable to early diverging iguanodontians but lower than hadrosaurids. How cognition in *Muttaburrasaurus langdoni* related to behaviour is unknown. Although, social behaviour, such as communal nesting and herding, have been reported in the hadrosaurids, social behaviour in *Muttaburrasaurus langdoni* cannot be assessed until extensive terrestrial fossil sites for the taxon are discovered. We hypothesise that cerebral size in *Muttaburrasaurus langdoni* could have been linked to bipedal locomotion; however, future work is needed to test this proposition further. The proportions of the vertical semicircular canals suggest *Muttaburrasaurus langdoni* was a facultative biped and used quadrupedal locomotion for slow speed activities, such as low browsing and rest. From endosseous cochlear duct (ECD) length, a low to middle frequency hearing range (297–2,166 Hz) for *Muttaburrasaurus langdoni* is inferred. Low-frequency range hearing and phonation/vocalization (<1 kHz) could have been used for communication with conspecifics in open and closed habitats and when visual cues were limited. A phylogenetic analysis was not undertaken in this study. However, contrary to several recent phylogenetic analyses, the craniodental features revealed in this work, such as the highly unusual architecture of the muzzle, suggest that *Muttaburrasaurus* was distant from the rhabdodontomorphs. The phylogenetic relationships of *Muttaburrasaurus* are presently enigmatic but will be better informed by the new information presented herein.

## Methods

*Field collection of samples*—The holotype quarry (QML1794) is on private land. Access to the site followed written approval from the current landowner allowing the collection of vertebrate fossil materials for geoscientific study and their accession to the Queensland Museum. All new materials of the *Muttaburrasaurus langdoni* holotype (QMF6140) in this work have been accessioned to the Queensland Museum.

*Geochronology*—Portions of the QML1794, QML1794N and QM1817 (∼2,500 g) samples were cleaned, crushed, wet filtered to 300µm and the magnetics removed from the heavy fraction using a Frantz magnetic barrier separator. The remaining heavy fraction was heavy liquid separated in a Lithium hetero-polytungstate solution, which concentrated the higher density zircon fraction. Zircon was not recoverable from the holotype host rock sample (QML1794) but recovered from QML1794N and QML1817. The isolated zircon grains were mounted in epoxy resin and polished with oneµm diamond paste to reveal their internal grain structure. The zircon crystals were ablated with a NewWave193nm excimer laser system with a 2-volume cell coupled with an Agilent 8800 ICP-MS at the Queensland University of Technology Central Analytical Research Facility (CARF). Laser fluence was 3 J/cm^2^ and spot size 40 microns. Ablation was in He atmosphere and that carrier gas was blended with Ar *via* a Y-junction, then the gas introduced to the ICP-MS. Four reference materials were used before analysing 10 unknown grains accumulating about 23 measurements in each. Temora 2 (416.78 ± 0.33 Ma; (Black et al., 2004)) was used as the primary zircon age calibration standard, and Plešovice (337.13 ± 0.37 Ma; Sláma et al., 2008) was used as a monitor standard. An additional monitor standard, 91500 (1,065.4 ± 0.6 Ma; Wiedenbeck et al., 1995), was used. For trace element composition, NIST glass was used as the calibrant, and the zircons were assumed to have a Si content of 15.22%. For detrital samples, a common Pb correction is allowed if the grain becomes more concordant. This style of common Pb correction is ^208^Pb based with the underlying assumption that both the Pb/U and Pb/Th systems are in equilibrium. Only 5 of the 21 Cretaceous grains were common Pb corrected. Additionally, several trace element filters were invoked to ensure the zircons were inclusion free: La<2 ppm, Ti<200 ppm, P<2000 ppm. Also grains with great noise on ^207^Pb/^235^U (30%) were omitted. An analysis is deemed concordant when the ^206^Pb/^238^U and ^207^Pb/^235^U ages agree at better than 10%. The data were processed using Iolite 3 (Paton et al., 2011). The uncertainties used in calculations are propagated meaning they include a within-analysis term but also a term derived from the fit to Temora data, making comparison of this lab’s work to another viable. This extra uncertainty pushes the MSWD of these well-behaved reference materials to <1.0. This propagation exaggerates a within-grain uncertainty by roughly 30%. Plešovice, understood to be 337 Ma, returned a concordia age of 338.3+/−2.3 with MSWD of 0.69, a highly accurate result for *n* = 23. Zircon 91500 gave a concordia age of 1058+/−8.8 with MSWD of 0.52 for 22 analyses, a result that just overlaps the accepted age.

*Anatomical*—The skull of the *Muttaburrasaurus langdoni* holotype (QMF6140) was measured and visualised using standard methods (vernier callipers, digital photography using Canon 40D SLR; hand sketches), photogrammetry and computed tomographic (CT) imagery. Photogrammetry of the main skull blocks (cranial parts 1, 2) was produced from a sequence of ∼2400 photographs using Canon PowerShot SX200IS, Panasonic Lumix T7, Olympus Tough digital cameras and processed in Agisoft (https://www.agisoft.com) using the parameters: (1) Metashape 1.4: Align Photos = HIGH; Key Point Limit = 40,000; Tie Point Limit = 10,000. (2) Preselection: Generic; Dense Point Cloud; Quality = High. (3) Filtering = Aggressive; Point Colour = Enabled. Build Mesh: Source = Dense Cloud; Surface Type = Arbitrary; Face Count = High. (4) Texture Generation: Mapping = Generic; Blending = Mosaic. Volume rendered models of the crania, dentition and endocranium were produced from the output of four computed tomographic sources. (1) The main skull blocks (cranial parts 1, 2) were scanned at the Queensland XRay facility at Greenslopes Private Hospital, Queensland, on their Siemens Somatom Definition Flash, Dual Energy CT scanner. Data set one (DINOSAUR_0_5_BONE_THINS_B_SN140KV_0009) was retrieved at 140 kVP, X-ray tube current 357 µA, slice thickness 500 µm, 1961 slices and a window width of 350 mm (causing clipping of the widest cranial region). Data set two (LARGE_ABDOMEN_0_5_I26F_2_0011) was retrieved at 140 kVP, X-ray tube current 780 µA, slice thickness 500 µm, 1961 slices and a window width of 1500 mm (allowing full skull scanning). Dual Energy CT scan data were collected at 100 kVP and X-ray tube current of 22 µA but were not used owing to poorer resolution. (2) Cranial parts 3, 5–9, 11 were scanned at the University of New England on their GE Phoenix v—tome—x µCT scanner (cranial parts 5 and 6 at 210 kVP, current 95.3 µA; voxels 54.560 µm; cranial part 7 at 210 kVP, current 95.3 µA, 52.075 µm; cranial part 8 at 200 kVP; current 90 µA; 77.176 µm). (3, 4) Cranial parts 3, 5, 8–14, were scanned at Australia’s Nuclear Science and Technology Organisation (ANSTO) facilities (see below).

*Synchrotron data acquisition and reconstruction protocols*—An X-ray microtomographic measurement of cranial part 3 was performed using the Imaging and Medical Beamline (IMBL) at the Australian Nuclear Science and Technology Organisation’s Australian Synchrotron, Melbourne, Australia. For this measurement, the beamline’s superconducting wiggler magnet was set to 4T, and the beam filtered to achieve a pink beam of synchrotron X-rays, with a peak intensity at 250 keV. The Xenia flat-panel detector with a native pixel size of 200 µm was employed, and the specimen mounted vertically between carbon fibre rods at a sample-to-detector distance of 2,000 mm. A total of 1,000 equally spaced, 0.033s exposure shadow-radiographs of obtained every 0.18° as the specimen was rotated continuously 180° about its vertical axis. Both dark (closed shutter) and beam profile (open shutter) images were obtained for calibration before initiating each shadow-radiograph acquisition. As the height of the specimen exceeded the detector field-of-view, the specimen was imaged using six consecutive scans. These raw 16-bit scan data were cropped, normalised and stitched, using the in-house IMBL Stitch software, to cover the entire specimen. Reconstruction of the tomographic dataset was achieved by the filtered-back projection method using the CSIRO X-TRACT software, a 3D Median filter of isotropic radius 2.0 applied to the reconstructed data. For measurements of cranial parts 5 and 11, the IMBL instrument was configured with a monochromatic beam energy of 80 keV, a sample-to-detector distance of 1,000 mm and use of the “Ruby” detector consisting of a PCO.edge sCMOS camera (16-bit, 2,560 × 2,160 pixels) and a Nikon Makro Planar 100 mm lens coupled with a 20 µm thick Gd_2_O_3_/CsI(Tl)/CdWO_4_ scintillator screen to achieve a pixel size of 35.02 × 35.02 µm. As the height of the specimens exceeded the detector field-of-view, the specimens were aligned axially relative to the beam and imaged using five consecutive scans, each consisting of 1,800 equally spaced angle shadow-radiographs with an exposure length of 0.30 s, obtained every 0.10° as the samples were continuously rotated 180° about its vertical axis. Vertical translation of the specimens between tomographic scans was 20 mm. 100 dark (closed shutter) and beam profile (open shutter) images were obtained for calibration before and after shadow-radiograph acquisition. Total time for the scans were 42 min. The raw 16-bit radiographic series were normalised relative to the beam calibration files and stitched using the CSIRO X-TRACT software to yield a 32-bit series with a field-of-view of 89.6 × 96.8 mm. Reconstruction of the 3D dataset was achieved by the filtered-back projection method using ImageJ 1.51 h. Measurements for cranial part 8 followed the same methods as for cranial parts 5 and 11, but consisted of six consecutive scans with a field-of-view of 90.0 × 129.6 mm and a duration of 42 min.

*Neutron scattered data acquisition and reconstruction protocols*—Neutron tomographic measurements was conducted using the DINGO thermal-neutron imaging instrument (Garbe et al., 2015) at the Australian Nuclear Science and Technology Organisation’s (ANSTO) 20 MW OPAL nuclear research reactor, Sydney, Australia. For measurements of cranial parts 9, 10, 12–14, the instrument was equipped with a MARANA sCMOS camera (2048 ×2048 pixels, 16 bit) and Zeiss Ikon 100 mm f/2.0 Makro Planar lens, and configured with a 100 µm thick ZnS(Ag)/^6^LiF scintillating screen (RC Tritec AG) to achieve a pixel size of 45.6 × 45.6 µm and Field-of-View of 93.4 × 93.4 mm. To maximise signal-to-noise in the tomographic reconstruction, the instrument was configured in high-intensity mode with a collimation ratio (*L*/*D*) of 500, where *L* is the neutron aperture-to-sample length and *D* is the neutron aperture diameter. The specimen was vertically mounted between aluminium support poles, and a total of 1280 equally spaced 2s angle shadow-radiographs were acquired as the sample was continuously rotated 180° about its vertical axis. Both dark (closed shutter) and beam profile (open shutter) images were obtained for calibration before shadow-radiograph acquisition. Total scan time was of 130 min. The raw radiographs were normalised and cleaned using ImageJ and binned by a factor of two to yield a tomographic reconstruction with 91.7 µm cubic voxels using Octopus Reconstruction v.8.8. For the measurement of cranial part 13, the instrument was equipped with a ZWO ASI2600MM Pro camera (4,176 × 6,248 pixel, 16 bit) and Zeiss Ikon 50 mm f/2.0 Makro Planar lens, and configured with a 50 µm thick ZnS(Ag)/^6^LiF scintillating screen (RC Tritec AG) to achieve a pixel size of 42.7 × 42.7 µm and Field-of-View of 178 × 200 mm. To maximise signal-to-noise in the tomographic reconstruction, the instrument was configured in high-intensity mode with a collimation ratio (*L*/*D*) of 500, where *L* is the neutron aperture-to-sample length and *D* is the neutron aperture diameter. The specimen was vertically mounted between aluminium support poles. A total of 1,200 equally spaced, 6s angle shadow-radiographs were acquired every 0.15° as the specimen were rotated 180° about its vertical axis. Both dark (closed shutter) and beam profile (open shutter) images were obtained for calibration before initiating each shadow-radiograph acquisition. Total scan time was of 3.5 h. The raw radiographs were normalised and cleaned using ImageJ and binned by a factor of two to yield a tomographic reconstruction with 84.4 µm cubic voxels using Octopus Reconstruction v.8.8.

*Data visualisation*—Volume rendered mesh files of the crania were generated in ORS Dragonfly 2019 4.1, and 2021.1 Build 977 (http://www.theobjects.com). The neural endocast was volume rendered in Mimics v21 (http://www.materialize.com). Segmentation in Dragonfly and Mimics was carried out manually and separate meshes were produced for each element. Segmentation of the neural endocast in Mimics used the interpolate function for approximately 10 slices at a time following manual painting of the ROI (regions of interest). Segmentation in Dragonfly utilizing the machine learning and deep learning functions was not used owing to computer processor limitations. Furthermore, osteological knowledge and operator experience was necessary for decisions on the boundaries between the bones and mudrock matrix in the thickest blocks with low resolution. Thresholding in Dragonfly utilized the ‘Range’ function, where possible. However, in some instances, differentiation of bone and matrix was not found using Range, even though the margins could be visually seen. In these instances, manual segmentation of the edges was conducted. The slice function in view mode, was mostly used and occasionally slab mIP (minimum), which provides a view through several layers in an orthogonal view. Some of the CT generated radiographic slices used slab mIP to help clarify the anatomy. The 3D brush tool was used through a spacing of ∼3 slices. Medical CT scans of the large skull blocks (cranial parts 1, 2) had poorer resolution between bone and matrix than the higher resolution µCT, synchrotron and neutron generated imagery from the smaller craniodental fragments (cranial parts 3–14). However, the poorly defined margins between the bones on cranial parts 1 and 2 were mostly resolved by visual interpolation by the operator using the better-defined margins viewed across the orthogonal slices. The ability to rapidly adjust the orthogonal plane orientations in Dragonfly assisted this process. Metrological data for elements of interest were collected using the length measurement and angle tools. Several measurements (approximately 3) were generally taken to help ensure accuracy. The meshes generated were smoothed in Dragonfly (one iteration) and exported for post-processing in Autodesk Meshmixer v3.5.474 (http://www.meshmixer.com) and ZBrush (http://www.pixologic.com), where surface artefacts were smoothed and extraneous geometry (noise) was removed (automated in Meshmixer under analyse geometry and repair). Post-processing was aimed at improving model readability without altering the morphological integrity. The bones were digitally reconstructed and coloured in ZBrush and production of the figures utilized Adobe CC (http://www.adobe.com) software.

*Body mass*—Body mass of the *Muttaburrasaurus langdoni* holotype was estimated using the formula by Campione & Evans (2012) for quadrupedal dinosaurs, denoted by QE and the formula by Campione et al. (2014) for dinosaurian bipeds, denoted by cQE, which is a mathematical correction of QE, applicable to bipeds. QE is a phylogenetically corrected formula for estimating the body mass of quadrupedal terrestrial tetrapods, extending from the pioneering work of Anderson, Hall-Martin & Russell (1985), utilising the minimum stylopodial circumferences of the humeral (*C*_*H*_) and femoral (*C*_*F*_) diaphyses (cm), where: *logBM*=* 1.54 x logC*_*H*_ *+ 1.195 x log C*_*F*_ *–0.234*. cQE uses the single stylopodial variable of *C*_*F*_, where: *log*_10_*BM*=*2.754 x log*_10_*C*_*F*_ *–0.683*. An error of ±25% is applicable (for additional information on error analyses, see Campione et al., 2014). The minimum measurable circumferences of the left humeral diaphysis (the right is not preserved) and left and right femoral diaphyses were taken using a taut nylon cord (diameter 1.5 mm) and measured along a steel rule. Three measurements were taken for each element and their averages taken. The average of the left and right femoral circumferences were used. It is notable that the middle part of the left humeral diaphysis is missing and the circumference measured might not be the narrowest part of the original diaphysis.

*Life restoration of the head and eye size*—A digitally generated, 3D life restoration of the *Muttaburrasaurus langdoni* head was sculpted in ZBrush (https://www.pixologic.com) around the volume rendered cranial osteology of the holotype. The better-preserved left side of the volume rendered skull was mirrored to the right side to produce a complete cranial model. Extensive retrodeformation of the skull and myological reconstruction were not performed. However, as the mandible appears to have rotated postmortem along its anteroposterior axis and compacted up to 30 mm towards the skull roof, the reconstruction depressed the quadratomandibular joint and ventral margin of the mandible by ∼20 mm. The sculpted integumentary surface followed the bone surfaces, with the addition of up to 15 mm thickness added for skin and connective tissue. The eye was sized based on orbit length in the regression formula for birds by Schmitz (2009): *y* = *1.34x*^0.87^, where *y* is eye diameter and *x* is orbit length. The anterior-most end of the paired premaxillae is missing but would have formed the edentulous beak anterior to the premaxillary dentition. Reconstruction of the beak was conjectural, but guided by taxa such as *Hypsilophodon foxii* (Galton, 1974) and *Thescelosaurus neglectus* (Boyd, 2014), who possess well-developed premaxillary dentition. Notably, the premaxillary rostra of early diverging neornithischians and ornithopods with well-developed dentition, are transversely narrow. The laterally concave surface preserved anterior to the dentition on the more complete left premaxillary fragment (cranial part 13) was used to guide the shape towards the postulated anterior tip of the beak. Extraoral soft-tissue in the form of cheeks was reconstructed (based on information in Morhardt, 2009; Nabavizadeh, 2020b; Galton, 1973; Sereno, 2012). The restoration was used to assess visual fields.

*Brain size and Cognition in Muttaburrasaurus langdoni*—The digitally extracted brain endocast of the *Muttaburrasaurus langdoni* holotype was used to calculate the volume and mass of the brain and sub-regions for assessments of reptile encephalisation quotient (REQ; Hurlburt, 1996) and relative cerebral volume (CRV) from which the ratio of cerebral mass to brain mass (MCb:MBr) was calculated (Hurlburt, Ridgely & Witmer, 2013). From the CT generated mesh model, the endocast volume was calculated using the slicing application Chitubox v1.9.3 (http://www.chitubox.com). The endocast volume was further calculated after the removal of the olfactory apparatus (lobes and tracts) and brain stem immediately posterior to cnXII (see; Jerison, 1969; Jerison, 1979), with digital separation of these regions performed in Autodesk Meshmixer v3.5.474 (http://www.meshmixer.com) prior to the volumetric measurement. The cerebrocast (cerebrum + meninges) was additionally separated digitally from the remaining endocast based on the cerebral boundaries identified (Figs. 53, 54) and its volume measured. It is notable that the cerebrum was separated at the boundary of the diencephalon, as noted also in the methods of Hurlburt, Ridgely & Witmer (2013). A brain-to-endocranial cavity index (BEC) (see Balanoff et al., 2015 and authors within) of 60% was used to estimate the volume of the brain filling the neural fossa (excluding the olfactory apparatus and brain stem posterior to cnXII), which allowed standardised comparisons with BEC 60% values reported for other ornithischians. Endocast volume to mass used the convention: 1.000 ml =1.000 g (see Hurlburt, Ridgely & Witmer, 2013). Calculation of the encephalisation quotient used the adjustment for reptiles by Hurlburt (1996; see also (Hurlburt, Ridgely & Witmer, 2013): *REQ* = *BrM/(0.0155 x BM*^0.553^; where *BrM* is brain mass (excluding the olfactory apparatus and brain stem posterior to cnXII) and *BM* is body mass. Body mass used the stylopodial-based cQE estimate for bipedal dinosaurs (see also under “Body mass” above) with REQ error additionally calculated from body mass error (±25%). The cQE estimate was used for REQ comparisons with other ornithischians in the literature (*e.g.*, Button & Zanno, 2023). A plot REQ of comparisons utilised brain and stylopodial-based body mass data reported by Button & Zanno (2023), As the REQs reported by Button & Zanno (2023) lacked the inclusion of error for body masses, error of ±25% were added based on Campione & Evans (2012) and Campione et al. (2014). In addition to body mass error, endocast error (endocast distortion, incomplete endocasts, poor digital resolution, uncertainty in the point of abscission of the olfactory peduncles from the telencephalic palladium, differentiation of the cerebrum from diencephalon) would increase REQ error. Endocast volume error for the *Muttaburrasaurus langdoni* holotype was likely but not added to the calculations of REQ, as error for the taxa compared was unknown. It is of note that the body mass for *Camptosaurus dispar*, as reported by Button & Zanno (2023), was based on an estimate by Colbert (1962), *via* Edinger (1964) (values also used by Jerison, 1973), from a model with unknown error derived from a plate of the mount in Gilmore (1909). Given the uncertain methodology linking the brain mass and endocast mass to the estimates for *Camptosaurus* by Colbert (1962), these values can only be considered provisional. Cerebral relative volume (CRV) was calculated for the *Muttaburrasaurus langdoni* holotype from the volume of the cerebrocast relative to the brain endocast (excluding the olfactory apparatus and the brain stem posterior to cnXII). From CRV, the ratio of cerebral mass to brain mass (excluding the olfactory apparatus and the brain stem posterior to cnXII) (*i.e.,* MCb:MBr) was calculated based on information in Hurlburt, Ridgely & Witmer (2013). From their work on extant alligators, Hurlburt, Ridgely & Witmer (2013) showed that cerebrum mass to cerebrocast volume and brain mass to brain volume were disproportionate. According to Hurlburt, Ridgely & Witmer (2013), the cerebrum of alligators filled more of the cerebrocast than full brain filled the endocast. The ratio of cerebrum mass to cerebrocast volume exceeded brain mass to endocast volume by 5.6%. As a result, to determine the ratio MCb:MBr (cerebrum mass to full brain mass, excluding the olfactory apparatus) in non-avialan dinosaurs of unknown sex, Hurlburt, Ridgely & Witmer (2013) applied 37% for the ratio of brain mass to endocast volume (MBr:EV) and 42% for the ratio of cerebrum mass to cerebrocast volume. These ratios are used in this study and to calculate the MCb:MBr for comparative taxa from their CRVs reported, used: *MCb:MBr* = *(CRV x 0.42)/0.37*.

*Olfactory ratio*—Using methods previously outlined (Bang & Cobb, 1968; Cobb, 1959; Zelenitsky, Therrien & Kobayashi, 2009; Zelenitsky et al., 2011), the olfactory ratio (OR) of the *Muttaburrasaurus langdoni* holotype was calculated by the greatest diameter of the olfactory bulbs divided by the greatest diameter of the cerebrum (regardless of orientations). The measurements were taken directly from the CT generated radiographs viewed in ORS Dragonfly, using the volume rendered model for reference (Table 2). Log transformed olfactory ratio was plotted against log transformed body mass for the holotype and selected non-avialan dinosaurs utilising the previously published dataset of Button & Zanno (2023; and authors within), modified with the removal of the ankylosaur *Euoplocephalus tutus* (as it was unclear where the data for olfactory ratio was sourced). A simple linear regression (= least-squares regression, trend line) for the minimised sum of square residuals was produced in Microsoft Excel (2019). Notably, body masses for individuals of the taxa in the dataset of Button & Zanno (2023) included a mixture of stylopodial-based and volumetric-based estimates from two primary sources (Sakagami & Kawabe, 2020; Zelenitsky, Therrien & Kobayashi, 2009; and authors within). The issue with mixing methods of obtaining body masses in non-avialan dinosaurs was discussed under “Brain size, cognition and locomotion”. For the analysis of OR the *Muttaburrasaurus langdoni* holotype, the volumetric-based body mass estimate (Bishop et al., 2020) was used. Some previous analyses have used phylogenetically independent contrasts to counter the weighting effects of taxon co-dependence in the dependent variable (y) data (Zelenitsky, Therrien & Kobayashi, 2009). However, out of the 25 total taxa in the dataset, only six are ornithischians. Furthermore, these ornithischians were dispersed over the plot without clustering. Examination of the initial plot indicated that the five ornithomimosaurs formed a cluster below the line of regression. Ornithomimosaurs formed the only closely clustered group. To reduce the weighting effect of the co-dependent ornithomimosaur cluster four of the ornithomimids were excluded, leaving *Ornithomimus edmontonicus* as a roughly central data point among the ornithomimid cluster (the non-ornithomimid ornithomimosaur *Garudimimus brevipes* was retained).

*Semicircular canal length*—The lengths of the anterior and posterior semicircular canals were calculated from the medical-CT generated radiographs aligned to the planes-of-best-fit by visually adjusting the x-y-z planes in ORS Dragonfly (http://www.theobjects.com) (Fig. S2). Using this method, a snapshot of the clearest complete loop was captured after adjustment. Lines following the peripheral margins of the canals were drawn in Adobe Illustrator (http://www.adobe.com) and circles of three mm diameter were visually placed over canals to locate the central axes (Fig. S2). Canal lengths were measured from the complete canal loops including through the common crus, secondary common crus and region of the utricle with the adjoining ampullae. It is notable that discrete expansions of the ampullae from the utricle were not evident in the CT output). The canal lengths were calculated from the sum of the three mm circles plus the fraction of the overlapping circle. The mean of the left and right sides was calculated, from which, the relative lengths of the anterior and posterior semicircular canals were reported. For the semicircular canals of the *Muttaburrasaurus langdoni* holotype, we considered using a two-dimensional planes-of-best-fit an appropriate method for measurement as negligible torsion in the canals was apparent, which would otherwise have required fitting of axial splines.

*Hearing range*—The range of best hearing frequencies for the *Muttaburrasaurus langdoni* holotype was estimated using the regression equations of Walsh et al. (2009), which use the variables of endosseous cochlear duct (ECD) length and the scaling factor of basicranial length, which accounts for differences in body size/mass among the species compared. The quotient was log transformed. The band of best hearing frequency range (BHF) was found by: BHF = 6,104.3 (log[ECD/basicranial length]) + 6,975.2. Mean hearing frequency (MHF), falling within the band best hearing frequency, was found by: MHF =3,311.3 (log[ECD/basicranial length]) + 4,000.8. ECD length was taken as the distance from the constriction between the *pars canalicularis* and *p. cochlearis* to the ventral-most (distal-most) point of the duct (following Walsh et al. (2009); noting these authors used *pars vestibularis* for *p. canalicularis* used herein), which was clearly seen in the radiographs (Fig. 55). The lengths of the left and right ECDs were measured using the measuring tool in ORS Dragonfly (http://www.theobjects.com) from the CT generated radiographs using the orthogonal planes adjusted to the planes of best fit in each duct(distances checked at least three times per side). It was found that the ECDs lacked any appreciable curvature, which, as a result, a single axis was measured; hence not requiring curve fitting tools. The mean of the left and right ECD lengths was used. Basicranial length was taken as the distance from the posterior-most point on the occipital condyle to the anterior end of the parasphenoid, excluding the cultriform process (Fig. 6A) (see also: Barker et al., 2023; Dudgeon et al., 2020), which was taken as the anterior-most point of merger at the proximal ends of the left and right basipterygoid processes. Basicranial length was measured in ORS Dragonfly by manually adjusting one of the orthogonal planes for alignment to the sagittal plane. The distance was taken from the mean of several measurements (not less than 3 measurements) using the measuring tool. The upper and lower limits of the best hearing frequency band were found by MHF ±BHF/2. Another widely used method for determining the hearing range of non-avialan dinosaurs, being that of Gleich, Dooling & Manley (2005), incorporates body mass as the scaling factor. However, we opted to use the scaling factor of basicranial length within the regression equations of Walsh et al. (2009), as we considered the margin of error from our calculations of body mass were too broad for a worthwhile estimate of hearing range using body mass as a scaling factor, let alone for comparisons, even though the estimates from these equations only provide approximations of hearing range (Barker et al., 2023). A margin of error would, of course, accompany the estimate of basicranial length, particularly in terms of accurately identifying the anterior end of parasphenoid, excluding the cultriform process. Distortion of the basicranium could also be an issue in some specimens; however, in the case of the *Muttaburrasaurus langdoni* holotype, distortion did not appear significant. In our study, we regarded basicranial length as a more reliable scaling factor than body mass, although error around our measurement of basicranial length was not assessed.

*Visual fields*—The visual fields of *Muttaburrasaurus langdoni* were assessed using virtual perimetry, inspired by inverse perimetry (Stevens, 2006) and ophthalmoscopic mapping methods (Cerio & Witmer, 2020; Martin, 2009; Martin & Katzir, 1999; Martin & Osorio, 2010) and utilised the digitally generated, 3D, life restoration of the holotype head, produced in ZBrush (http://www.maxon.net) (see above), which was set in a digitally generated virtual sphere (Fig. 61). The eyes were positioned according to their optic axes, each of which passes symmetrically through the cornea and lens to the fovea centralis of the retina. The optic axis was set in the ‘relaxed’ mid-orbit position taken as orthogonal to the orbital plane (based on Heesy, 2004; Heesy, 2009) (Fig. 61B). The head was oriented in the ‘alert’ position parallel to horizon, indicated by the orientation of the lateral semicircular canals. The virtual sphere size, although arbitrary, was set to a radius of ∼84 cm. Being beyond the point of convergence of the paired monocular fields (*i.e.,* the binocular field), this sphere size allowed the binocular field to be visualised. The polar axis of the sphere was set to pass through the point of convergence of the optic axes and the sagittal plane, which differs from that of Cerio & Witmer (2020), who set the polar axis to meet the optic axes at the corneal surfaces. However, the location of the sphere does not have a bearing on lines of sight measurement and is only illustrative. Twenty-two lines of sight distributed around the head were digitally produced in ZBrush on the right side as thin virtual blades and copied to the left side using the mirror tool (Fig. 61; Data S2; Fig. S3). The lines of sight (blades) penetrated the sphere’s surface allowing mapping of the visual fields. The blades were manipulated using the widget tool in ZBrush, which was centred on the cornea (Fig. 61; Fig. S3). This positioning of the widget tool allowed the manual global manipulation of the lines-of-sight from the cornea to points that brushed past contacts with the fleshed-out surfaces (*i.e.,* obstructions) (Fig. 61A). The angles were chosen according to visual assessment of the most informative trajectories. The head and the widget tool were preset to the 0,0,0 cartesian planes in ZBrush, which allowed alignment of the planes to be automatically reset. The angles of the blades viewed in the sagittal/lateral plane were measured directly from the widget tool during rotation relative to the dorsal (horizontal) and transverse planes. However, the convergent angles formed between the left and right line-of-sight blades in the region of binocular overlap were measured using a protractor after manually rotating the head to have the planes correspond to the horizontal plane in the ZBrush environment. An error of 1° was expected. After each convergent line-of-sight was measured, the head was reset to the cartesian planes. The divergence angle between the posterior most paired lines-of-sight on the horizon was similarly measured, although without the need to manipulate the head from the standard position. The dorsal, sagittal/lateral transverse and right anteroventral 3D views were visualised using Adobe CC (http://www.adobe.com). Notably, the optic field margins, as indicated by the lines-of-sight blades projecting through the sphere, were mapped onto the sphere surface in Adobe Illustrator. To mark the points on the surface from the projecting blades, a version of the sphere was made solid in ZBrush so the intersection between the blades and the sphere could be clearly seen and marked. The field margin map was formed by interpolation between the points on the sphere surface. Notably, as work on vision in birds has shown (Martin, 2022; Martin & Katzir, 1999; Martin & Osorio, 2010), the optic and light capturing retinal field margins are not equivalent. Although the pupil of the eye (= optic aperture) can be seen at the extreme circumferential edges of the head by an observer (with the eyes set in a fixed central gaze), the actual light gathering retinal surface is typically less, particularly in the anterior binocular field (Martin, 2022; Martin & Katzir, 1999). Hence, in our virtual simulation, only the optic field margins could be measured. Although we use the term ‘lines of sight’, the digital projections (*i.e.,* lines of sight) represent the optic field margins, not the photoreceptive retinal margins.

##  Supplemental Information

10.7717/peerj.20794/supp-1 Supplemental Information 1 Sedimentological features in the region of the *Muttaburrasaurus langdoni* holotype locality (QML1794) (A) Block of a fragmented coquina concretion collected at QML1817, from which detrital zircon was extracted for 238 U/ 207 Pb dating, ∼50 m from QML1794 (red dashed line on surface indicates a lamniform shark tooth) (photograph: M. C. Herne). (B) Drone image of concretions exposed in the Thomson River channel ∼275 m from QML1794. (C, D) Photographs of stratigraphic layers in carbonate concretions showing hummocky/swaley cross-stratified storm sand with low-angle erosion surfaces highlighted in yellow from viewpoints indicated in (C) as view 1 in B and (D) view 2 in B (photographs: A. M. Tait). Scale bar in B equals 10 m. Geopick in C and D, 28 cm long. Drone image in B, with permission: C. Rohan.

10.7717/peerj.20794/supp-2 Supplemental Information 2 Semicircular canal length measurement method for *Muttaburrasaurus langdoni* (QMF6140) Left anterior semicircular canal (A) and posterior semicircular canal (B) on planes of best-fit. Right anterior semicircular canal (C) and posterior semicircular canal (D) on planes of best-fit. Circle diameters equal 3 mm. Scale bars equal 1 cm.

10.7717/peerj.20794/supp-3 Supplemental Information 3 Line of sight manipulation method in ZBrush for virtual visual field perimetry around life restoration of the *Muttaburrasaurus langdoni* (QMF6140) head in lateral view Lines of sight and widget tool shown centred on the optic axis at the virtual cornea. As demonstrated, the widget tool is aligned with the anterior line of sight, -35 ° from horizontal (dorsal plane).

10.7717/peerj.20794/supp-4 Supplemental Information 4 Schematic, hypothesised superficial and middle mandibular musculature for *Muttaburrasauruslangdoni.* (A) Middle external adductor musculature of the mandible in left lateral view. (B) Superficial external adductor and depressor musculature of the mandible in left lateral view. Dashed line in A indicates dorsal margins of coronoid and surangular and grey shaded area indicates missing region of mandibular corpus. Dashed line in B indicates lateral surface of origin of mAMES on upper temporal bar (missing). Abbreviations: eost?, extra-oral soft-tissue; j, jugal; mDM, musculus depressor mandibulae; mAMEM, musculus adductor mandibulae externus medialis; mAMEP, musculus adductor mandibulae externus profundus; mAMES, musculus adductor mandibulae externus superficialis; mPTv, musculus pterygoideus ventralis; qj, quadratojugal. Sources: Holliday (2009); Nabavizadeh (2020); see main text. Scale bar equals 10 cm.

10.7717/peerj.20794/supp-5 Supplemental Information 5 Cast skeletal mount of the *Muttaburrasaurus langdoni* holotype at the National Museum of Australia Skeletal mount in natural bipedal pose suggesting height of the orbit from ground level. Image: M. C. Herne.

10.7717/peerj.20794/supp-6 Supplemental Information 6 Zircon age data results for QML1794N and QML1817 in the Mackunda Formation, Eromanga Basin, Queensland, near the Muttaburrasaurus langdoni holotype locality

10.7717/peerj.20794/supp-7 Supplemental Information 7 Herne et al 2026 Muttaburrasaurus Cranium DOI Links

10.7717/peerj.20794/supp-8 Supplemental Information 8 Queensland Fossicking Act 1994 current at 2019

10.7717/peerj.20794/supp-9 Supplemental Information 9 *Muttaburrasaurus langdoni*, lines of sight relative to the horizon and sagittal plane

10.7717/peerj.20794/supp-10 Supplemental Information 10 Complete MorphoSource DOI list

## Institutional abbreviations

AM: Australian Museum, Sydney, New South Wales, Australia
FMNH: Field Museum of Natural History, Chicago, Illinois, USA
LRF: Australian Opal Centre, Lightning Ridge, New South Wales, Australia
MCF PVPH: Museo Carman Funes, Paleontología de Vertebrados, Plaza Huincul, Neuquén Province, Argentina
MNHN: Muséum National d’Historie Naturelle, Paris, France
MUCPv: Museo de Geología y Paleontología de la Universidad Nacional del Comahue, Paleontología de Vertebrados, Neuquén Province, Argentina
NMV: National Museum of Victoria (Museums Victoria), Melbourne, Victoria, Australia
NHMUK: Natural History Museum, London, United Kingdom
QM: Queensland Museum, Brisbane, Queensland, Australia
RBINS: Royal Belgian Institute of Natural Sciences, Brussels, Belgium
ROM: Royal Ontario Museum, Toronto, Ontario, Canada
SGM: Minestere de l’Energie et des Mines, Rabat, Morocco
UUVP: University of Utah, Salt Lake City, USA
YPM VP: Yale Peabody Museum (Vertebrate Paleontology), New Haven, Connecticut, USA

## Additional Information and Declarations

### Field Study Permissions

The following information was supplied relating to field study approvals (i.e., approving body and any reference numbers): not applicable

### Data Availability

The following information was supplied regarding data availability: Raw data for zircon dating in the geochronology is available in the Supplemental File. The 3D mesh files of the CT data, individual crania, complete skull and skull photogrammetry are available in Morphosource: (DOIs) DINOSAUR_0_5_BONE_THINS_B_SN140KV_0004 (cranial parts 1, 2) 10.17602/M2/M788508; DE 140KVA, Large Abdomen (cranial parts 1, 2), 10.17602/M2/M787635; cranium photogrammetry (cranial parts 1, 2), 10.17602/M2/M786923; right premaxilla dental ramus (cranial part 14), 10.17602/M2/M786841; right premaxilla germ teeth (cranial part 14), 10.17602/M2/M786844; right premaxilla functional teeth (cranial part 14), 10.17602/M2/M786847; left premaxilla dental ramus (cranial parts 6, 7, 12), 10.17602/M2/M786859; left premaxilla germ teeth (cranial parts 6, 7, 12), 10.17602/M2/M786862; left premaxilla functional teeth (cranial parts 6, 7, 12), 10.17602/M2/M786887; left premaxilla dental ramus (cranial part 13), 10.17602/M2/M786850; left premaxilla germ teeth (cranial part 13), 10.17602/M2/M786853; left premaxilla functional teeth (cranial part 13), 10.17602/M2/M786856; left premaxilla posterodorsal process (cranial part 2), 10.17602/M2/M787709; left premaxilla posteroventral process (cranial part 2), 10.17602/M2/M787712; right maxilla dental ramus (cranial part 8), 10.17602/M2/M786911; right maxilla germ teeth (cranial part 8), 10.17602/M2/M786915; right maxilla functional teeth (cranial part 8), 10.17602/M2/M786919; right maxilla dental ramus (cranial part 9), 10.17602/M2/M771412; right maxilla germ teeth (cranial part 9), 10.17602/M2/M786868; right maxilla functional teeth (cranial part 9), 10.17602/M2/M786871; left maxilla (anterior half) dental ramus (cranial part 2), 10.17602/M2/M787646; left maxilla (anterior half) germ teeth (cranial part 2), 10.17602/M2/M787655; left maxilla (anterior half) functional teeth (cranial part 2), 10.17602/M2/M787661; left maxilla (posterior half) dental ramus (cranial part 1), 10.17602/M2/M787649; left maxilla (posterior half) germ teeth (cranial part 1), 10.17602/M2/M787652; left maxilla (posterior half) functional teeth (cranial part 1), 10.17602/M2/M787658; left prenasal (cranial part 2), 10.17602/M2/M787694; right prenasal (cranial part 2), 10.17602/M2/M787691; prenasal septa (cranial part 2), 10.17602/M2/M787697; prenasal septa additional (cranial part 2), 10.17602/M2/M787700; left jugal (cranial part 1), 10.17602/M2/M787715; left lacrimal anterior half (cranial part 2), 10.17602/M2/M787721; left lacrimal posterior half (cranial part 1), 10.17602/M2/M787718; left lacrimal complete (cranial parts 1, 2), 10.17602/M2/M787734; left quadratojugal (cranial part 1), 10.17602/M2/M787743; left quadrate (cranial part 1), 10.17602/M2/M788110; left nasal (cranial parts 1, 2), 10.17602/M2/M787703; right nasal (cranial part 1), 10.17602/M2/M787706; left prefrontal (cranial part 1), 10.17602/M2/M788113; left frontal (cranial part 1), 10.17602/M2/M788116; right frontal (cranial part 1), 10.17602/M2/M788121; left postorbital (cranial part 1), 10.17602/M2/M788124; left intrapostorbital (cranial part 1), 10.17602/M2/M788131; left pterygoid (cranial part 1), 10.17602/M2/M787756; right pterygoid (cranial part 1), 10.17602/M2/M787749; vomers (cranial parts 1, 2), 10.17602/M2/M787746; left palatine (cranial part 1), 10.17602/M2/M790005; left ectopterygoid (cranial part 1), 10.17602/M2/M790002; basioccipital (cranial part 1), 10.17602/M2/M788140; parabasisphenoid (cranial part 1), 10.17602/M2/M788143; orbitosphenoid (cranial part 1), 10.17602/M2/M788146; left laterosphenoid (cranial part 1), 10.17602/M2/M789996; right laterosphenoid (cranial part 1), 10.17602/M2/M789999; left prootic (cranial part 1), 10.17602/M2/M788155; right prootic (cranial part 1), 10.17602/M2/M788158; left otoccipital (cranial part 1), 10.17602/M2/M788152; right otoccipital (cranial part 1), 10.17602/M2/M788149; supraoccipital (cranial part 1), 10.17602/M2/M788137; left and right squamosals (cranial part 1), 10.17602/M2/M790008; parietals (cranial part 1), 10.17602/M2/M788134; left dentary ramus, anterior half (cranial parts 3, 5, 10), 10.17602/M2/M786907; left dentary germ teeth, anterior half (cranial parts 3, 5, 10), 10.17602/M2/M786883; left dentary functional teeth, anterior half (cranial parts 3, 5, 10), 10.17602/M2/M786887; left dentary ramus, posterior half (cranial part 1), 10.17602/M2/M787664; left dentary germ teeth, posterior half (cranial part 1), 10.17602/M2/M787667; left dentary functional teeth, posterior half (cranial part 1), 10.17602/M2/M787670; right dentary ramus, anterior half (cranial part 11), 10.17602/M2/M786891; right dentary germ teeth, anterior half (cranial part 11), 10.17602/M2/M786899; right dentary germ teeth, anterior half (cranial part 11), 10.17602/M2/M786903; right dentary dental parapet, anterior half (cranial part 11), 10.17602/M2/M786895; left surangular (cranial part 1), 10.17602/M2/M787673; left angular (cranial part 1), 10.17602/M2/M787676; left coronoid (cranial part 1), 10.17602/M2/M787685; left prearticular (cranial part 1), 10.17602/M2/M787679; left articular (cranial part 1), 10.17602/M2/M787682; left ceratobranchial (cranial part 1), 10.17602/M2/M787688; neural endocranium (cranial part 1), 10.17602/M2/M788215; cranium CT surface mesh (cranial parts 1, 2), 10.17602/M2/M788197.
