# Supplementary figures and images for: Cranial anatomy, palaeoneurology, palaeobiology and stratigraphic age of the large-bodied ornithopod, Muttaburrasaurus langdoni Bartholomai and Molnar, 1981, from the mid-Cretaceous of Australia
Source: PeerJ. 2026 Apr 9;14:e20794. doi: 10.7717/peerj.20794 (PMC13070326; doi:10.7717/peerj.20794)

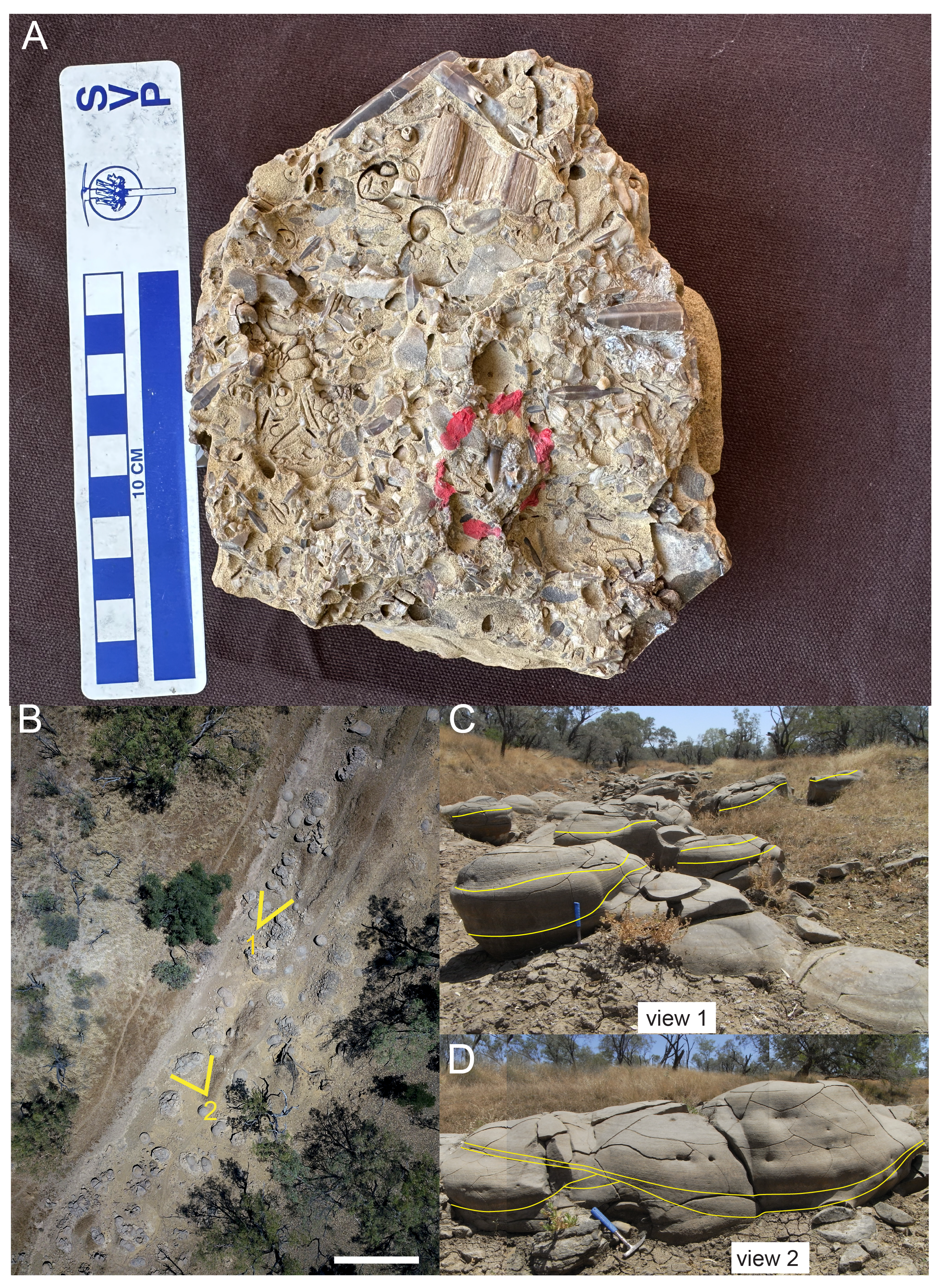

Supplement: Supplemental Information 1 — (A) Block of a fragmented coquina concretion collected at QML1817, from which detrital zircon was extracted for 238 U/ 207 Pb dating, ∼50 m from QML1794 (red dashed line on surface indicates a lamniform shark tooth) (photograph: M. C. Herne). (B) Drone image of concretions exposed in the Thomson River channel ∼275 m from QML1794. (C, D) Photographs of stratigraphic layers in carbonate concretions showing hummocky/swaley cross-stratified storm sand with low-angle erosion surfaces highlighted in yellow from viewpoints indicated in (C) as view 1 in B and (D) view 2 in B (photographs: A. M. Tait). Scale bar in B equals 10 m. Geopick in C and D, 28 cm long. Drone image in B, with permission: C. Rohan. [file peerj-14-20794-s001.png]

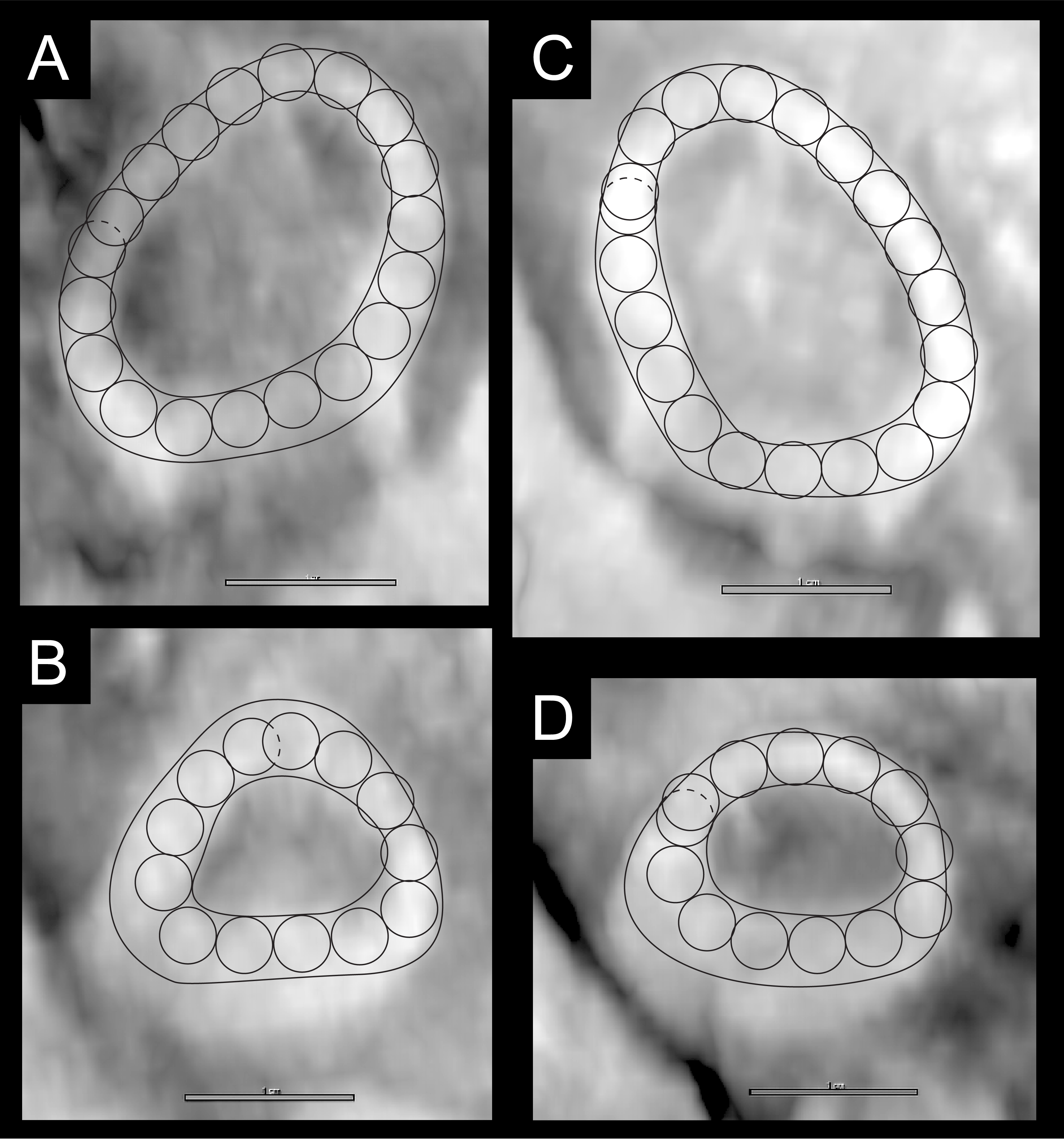

Supplement: Supplemental Information 2 — Left anterior semicircular canal (A) and posterior semicircular canal (B) on planes of best-fit. Right anterior semicircular canal (C) and posterior semicircular canal (D) on planes of best-fit. Circle diameters equal 3 mm. Scale bars equal 1 cm. [file peerj-14-20794-s002.png]

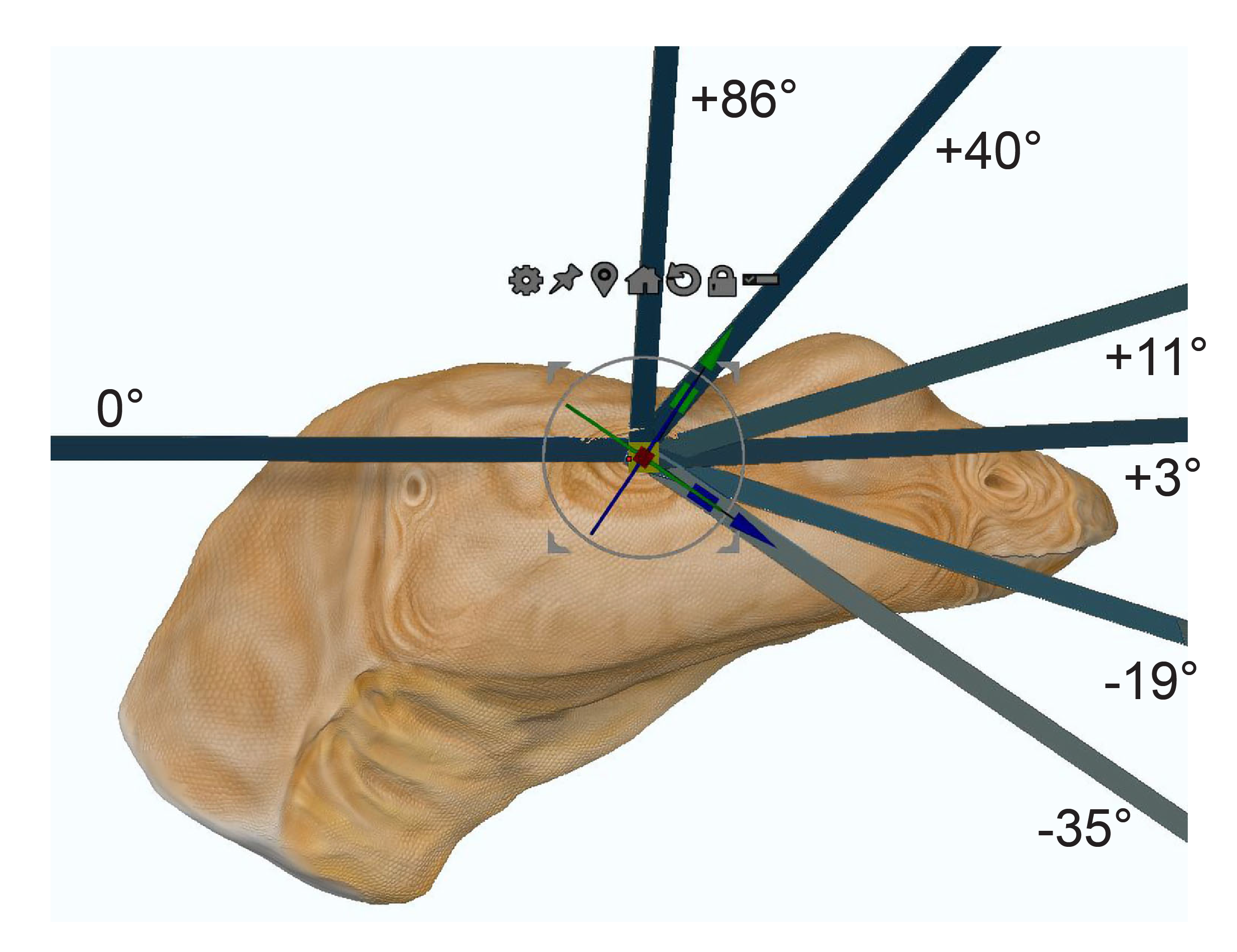

Supplement: Supplemental Information 3 — Lines of sight and widget tool shown centred on the optic axis at the virtual cornea. As demonstrated, the widget tool is aligned with the anterior line of sight, -35 ° from horizontal (dorsal plane). [file peerj-14-20794-s003.png]

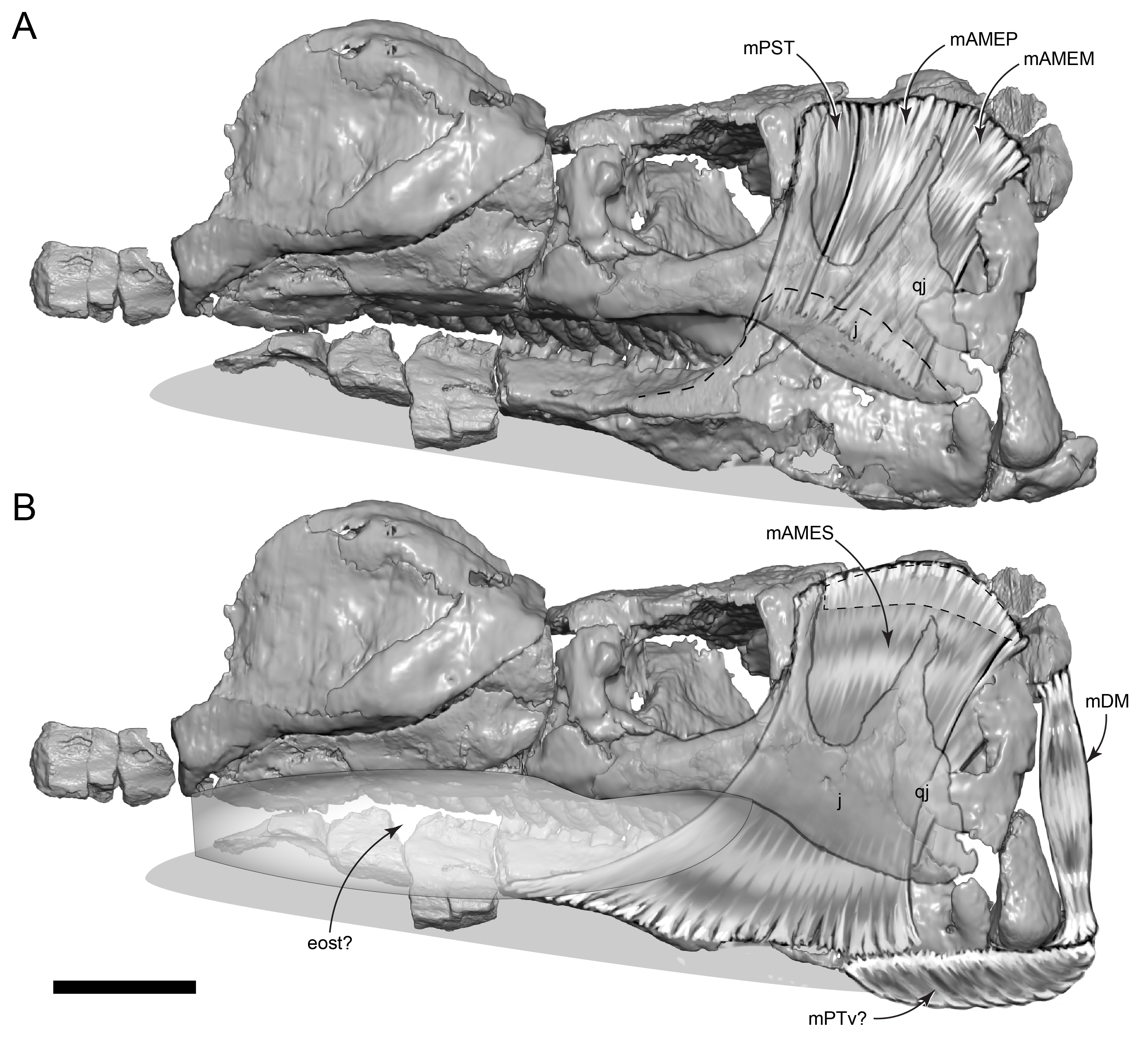

Supplement: Supplemental Information 4 — (A) Middle external adductor musculature of the mandible in left lateral view. (B) Superficial external adductor and depressor musculature of the mandible in left lateral view. Dashed line in A indicates dorsal margins of coronoid and surangular and grey shaded area indicates missing region of mandibular corpus. Dashed line in B indicates lateral surface of origin of mAMES on upper temporal bar (missing). Abbreviations: eost?, extra-oral soft-tissue; j, jugal; mDM, musculus depressor mandibulae; mAMEM, musculus adductor mandibulae externus medialis; mAMEP, musculus adductor mandibulae externus profundus; mAMES, musculus adductor mandibulae externus superficialis; mPTv, musculus pterygoideus ventralis; qj, quadratojugal. Sources: Holliday (2009); Nabavizadeh (2020); see main text. Scale bar equals 10 cm. [file peerj-14-20794-s004.png]

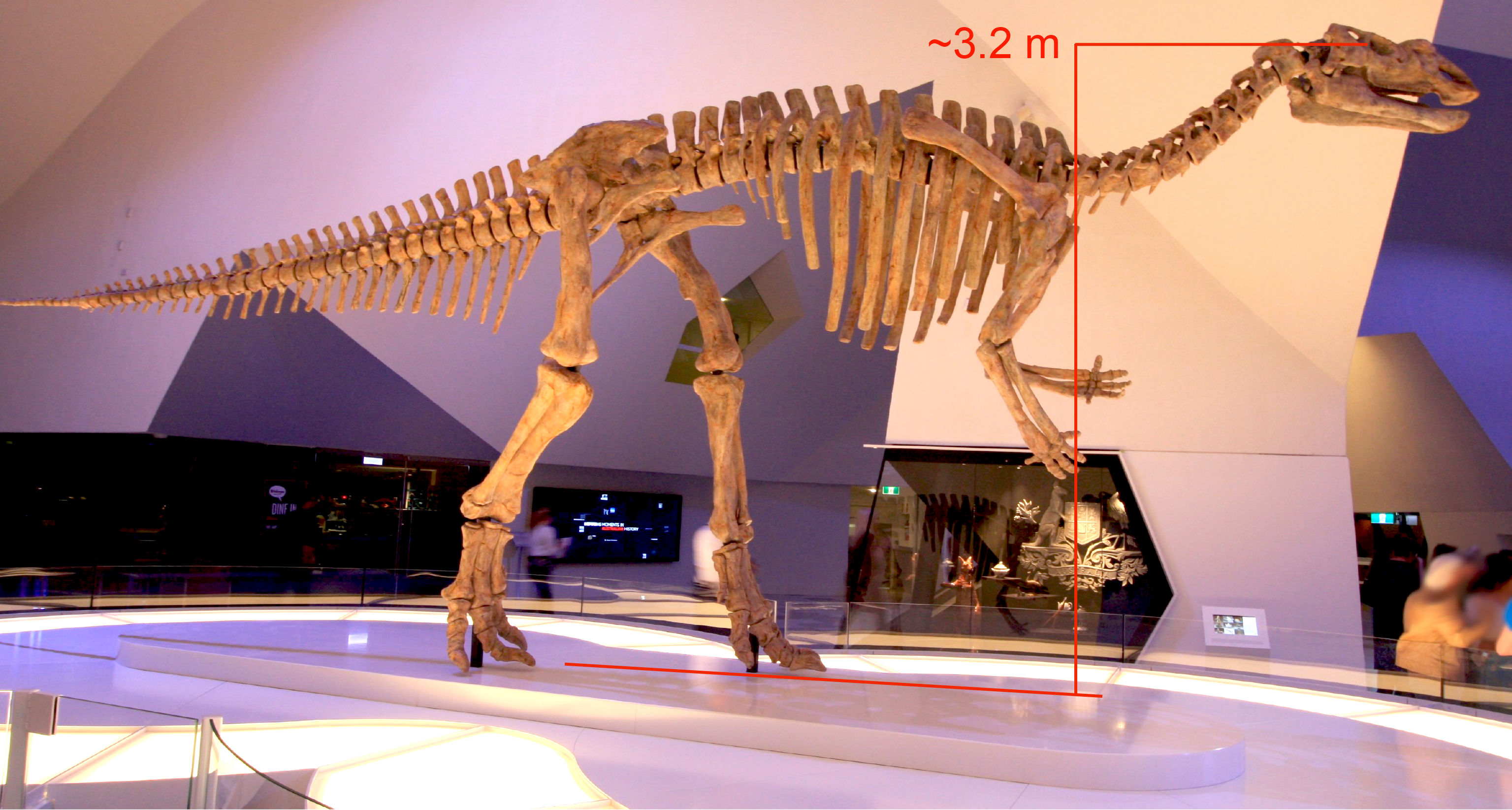

Supplement: Supplemental Information 5 — Skeletal mount in natural bipedal pose suggesting height of the orbit from ground level. Image: M. C. Herne. [file peerj-14-20794-s005.png]
