# Supplementary material for: Cranial anatomy, palaeoneurology, palaeobiology and stratigraphic age of the large-bodied ornithopod, Muttaburrasaurus langdoni Bartholomai and Molnar, 1981, from the mid-Cretaceous of Australia
Source: PeerJ. 2026 Apr 9;14:e20794. doi: 10.7717/peerj.20794 (PMC13070326; doi:10.7717/peerj.20794)
Supplement: Supplemental Information 7 [file peerj-14-20794-s007.pdf]

CRANIAL ANATOMY, PALAEONEUROLOGY, PALAEOBIOLOGY AND STRATIGRAPHIC AGE OF THE LARGE-BODIED ORNITHOPOD, *MUTTABURRASAURUS LANGDONI* BARTHOLOMAI AND MOLNAR, 1981, FROM THE MID-CRETACEOUS OF AUSTRALIA

Matthew C. Herne, Joseph J. Bevitt, Luke Milan, Scott A. Hocknull, Alan M. Tait, Charlotte Allen, Andrew Rozefelds, Ralph Molnar, Vera Weisbecker and Phil Bell

SUPPLEMENTAL LINK L1: MORPHOSOURCE DOIs

#	Item	Type	Cranial Part number	DOI
**1**	DINOSAUR_0_5_BONE_THINS_B_SN140KV_0004	CT scan	1, 2	10.17602/M2/M788508
**2**	CT scan: DE 140KVA; Large Abdomen	CT scan	1, 2	10.17602/M2/M787635
**3**	Cranium photogrammetry	Mesh	1, 2	10.17602/M2/M786923
**4**	Right premaxilla dental ramus	Mesh	14	10.17602/M2/M786841
**5**	Right premaxilla germ teeth	Mesh	14	10.17602/M2/M786844
**6**	Right premaxilla functional teeth	Mesh	14	10.17602/M2/M786847
**7**	Left premaxilla dental ramus	Mesh	6,7,12	10.17602/M2/M786859
**8**	Left premaxilla germ teeth	Mesh	6,7,12	10.17602/M2/M786862
**9**	Left premaxilla functional teeth	Mesh	6,7,12	10.17602/M2/M786887
**10**	Left premaxilla dental ramus	Mesh	13	10.17602/M2/M786850
**11**	Left premaxilla germ teeth	Mesh	13	10.17602/M2/M786853
**12**	Left premaxilla functional teeth	Mesh	13	10.17602/M2/M786856
**13**	Left premaxilla posterodorsal process	Mesh	2	10.17602/M2/M787709
**14**	Left premaxilla posteroventral process	Mesh	2	10.17602/M2/M787712
**15**	Right maxilla dental ramus	Mesh	8	10.17602/M2/M786911
**16**	Right maxilla germ teeth	Mesh	8	10.17602/M2/M786915
**17**	Right maxilla functional teeth	Mesh	8	10.17602/M2/M786919
**18**	Right maxilla dental ramus	Mesh	9	10.17602/M2/M771412

**19**	Right maxilla germ teeth	Mesh	9	10.17602/M2/M789993
**20**	Right maxilla functional teeth	Mesh	9	10.17602/M2/M789990
**21**	Left maxilla (anterior half) dental ramus	Mesh	2	10.17602/M2/M787646
**22**	Left maxilla (anterior half) germ teeth	Mesh	2	10.17602/M2/M787655
**23**	Left maxilla (anterior half) functional teeth	Mesh	2	10.17602/M2/M787661
**24**	Left maxilla (posterior half) dental ramus	Mesh	1	10.17602/M2/M787649
**25**	Left maxilla (posterior half) germ teeth	Mesh	1	10.17602/M2/M787652
**26**	Left maxilla (posterior half) functional teeth	Mesh	1	10.17602/M2/M787658
**27**	Left prenasal	Mesh	2	10.17602/M2/M787694
**28**	Right prenasal	Mesh	2	10.17602/M2/M787691
**29**	Prenasal septa	Mesh	2	10.17602/M2/M787697
**30**	Prenasal septa additional	Mesh	2	10.17602/M2/M787700
**31**	Left jugal	Mesh	1	10.17602/M2/M787715
**32**	Left lacrimal (anterior half)	Mesh	2	10.17602/M2/M787721
**33**	Left lacrimal (posterior half)	Mesh	1	10.17602/M2/M787718
**34**	Left lacrimal (complete)	Mesh	1, 2	10.17602/M2/M787734
**35**	Left quadratojugal	Mesh	1	10.17602/M2/M787743
**36**	Left quadrate	Mesh	1	10.17602/M2/M788110
**37**	Left nasal	Mesh	1, 2	10.17602/M2/M787703
**38**	Right nasal	Mesh	1	10.17602/M2/M787706
**39**	Left prefrontal	Mesh	1	10.17602/M2/M788113
**40**	Left frontal	Mesh	1	10.17602/M2/M788116
**41**	Right frontal	Mesh	1	10.17602/M2/M788121
**42**	Left postorbital	Mesh	1	10.17602/M2/M788124
**43**	Left intrapostorbital	Mesh	1	10.17602/M2/M788131
**44**	Left pterygoid	Mesh	1	10.17602/M2/M787756
**45**	Right pterygoid	Mesh	1, 2	10.17602/M2/M787749
**46**	Vomers	Mesh	1	10.17602/M2/M787746
**47**	Left palatine	Mesh	1	10.17602/M2/M790005
**48**	Left ectopterygoid	Mesh	1	10.17602/M2/M790002
**49**	Basioccipital	Mesh	1	10.17602/M2/M788140
**50**	Parabasisphenoid	Mesh	1	10.17602/M2/M788143
**51**	Orbitosphenoid	Mesh	1	10.17602/M2/M788146
**52**	Left laterosphenoid	Mesh	1	10.17602/M2/M789996
**53**	Right laterosphenoid	Mesh	1	10.17602/M2/M789999
**54**	Left prootic	Mesh	1	10.17602/M2/M788155

**55**	Right prootic	Mesh	1	10.17602/M2/M788158
**56**	Left otoccipital	Mesh	1	10.17602/M2/M788152
**57**	Right otoccipital	Mesh	1	10.17602/M2/M788149
**58**	Supraoccipital	Mesh	1	10.17602/M2/M788137
**59**	Left / right squamosals	Mesh	1	10.17602/M2/M790008
**60**	Parietals	Mesh	1	10.17602/M2/M788134
**61**	Left dentary (anterior half) ramus	Mesh	3,5,10	10.17602/M2/M786907
**62**	Left dentary (anterior half) germ teeth	Mesh	3,5,10	10.17602/M2/M786883
**63**	Left dentary (anterior half) functional teeth	Mesh	3,5,10	10.17602/M2/M786887
**64**	Left dentary (posterior half) ramus	Mesh	1	10.17602/M2/M787664
**65**	Left dentary (posterior half) germ teeth	Mesh	1	10.17602/M2/M787667
**66**	Left dentary (posterior half) functional teeth	Mesh	1	10.17602/M2/M787670
**67**	Right dentary (anterior half) ramus	Mesh	11	10.17602/M2/M786891
**68**	Right dentary (anterior half) germ teeth	Mesh	11	10.17602/M2/M786899
**69**	Right dentary (anterior half) functional teeth	Mesh	11	10.17602/M2/M786903
**70**	Right dentary (anterior half) dental parapet	Mesh	11	10.17602/M2/M786895
**71**	Left surangular	Mesh	1	10.17602/M2/M787673
**72**	Left angular	Mesh	1	10.17602/M2/M787676
**73**	Left coronoid	Mesh	1	10.17602/M2/M787685
**74**	Left prearticular	Mesh	1	10.17602/M2/M787679
**75**	Left articular	Mesh	1	10.17602/M2/M787682
**76**	Left ceratobranchial	Mesh	1	10.17602/M2/M787688
**77**	Neural endocranium	Mesh	1	10.17602/M2/M788215
**78**	Cranium CT	Mesh	1,2	10.17602/M2/M788197
