# Supplementary material for: Cranial anatomy, palaeoneurology, palaeobiology and stratigraphic age of the large-bodied ornithopod, Muttaburrasaurus langdoni Bartholomai and Molnar, 1981, from the mid-Cretaceous of Australia
Source: PeerJ. 2026 Apr 9;14:e20794. doi: 10.7717/peerj.20794 (PMC13070326; doi:10.7717/peerj.20794)
Supplement: Supplemental Information 8 [file peerj-14-20794-s008.pdf]

Queensland

Fossicking Act 1994

Current as at 29 March 2019

© State of Queensland 2021

This work is licensed under a Creative Commons Attribution 4.0 International License.

Queensland

Fossicking Act 1994

Contents

		Page
**Part 1**	**Preliminary**	
1	Short title	7
2	Commencement	7
3	Definitions	7
4	Meaning of club	11
5	Meaning of fossick	12
6	Meaning of member	12
7	Meaning of watercourse	13
8	Meaning of expressions used in this and other Acts	13
**Part 2**	**Land excluded from Act’s application**	
9	Act’s application to protected areas	14
10	Act’s application to State forests, timber reserves and forest entitlement areas	14
12	Other land may be excluded from operation of Act	14
13	Act’s application to scientific organisations	14
**Part 3**	**Licences**	
**Division 1**	**Licences**	
14	Licences	15
15	Applications	15
16	How issuing officer may deal with applications for licence	16
17	License conditions must not be contravened	17
18	Licence not transferable	17
19	Term of licence	17
20	Replacement licences	17
21	Suspension and cancellation of licences—grounds	18
22	Suspension and cancellation of licences—procedures	18
23	Return of licence	19

**Division 2**	**Licensees' entitlements and duties**	
24	Meaning of licensee in division	20
25	Licence needed to fossick	20
26	Licensee's right to enter unoccupied land to fossick	21
27	Licensee must get permission to fossick on occupied land etc.	21
28	General permissions	22
29	Licensee must comply with conditions of permission	23
30	Commercial tour operator sometimes needs other permissions	24
31	Other limits on commercial tour operator's fossicker licence	24
32	Limits on club and educational organisation fossickers licence	24
33	Records of land mentioned in general permission to be kept	25
**Division 3**	**General**	
34	When fossicking materials become licensee's property	25
35	Payment of royalties to State	25
36	Sale or use of fossicking material in trade or commerce	26
37	Volume, weight or number of specimens may be restricted	26
38	Use of machinery etc. prohibited	26
39	Limits on digging	27
40	Discovery of minerals does not confer rights	27
**Part 4**	**Designated fossicking land and fossicking areas**	
**Division 1**	**Designated fossicking land**	
41	Proposal for declaration of designated fossicking land	28
42	Declaration of designated fossicking land	28
**Division 2**	**Fossicking areas**	
43	Proposal for declaration of fossicking area	29
44	Declaration of fossicking area	30
**Division 3**	**General**	
45	Declaration does not normally affect person's title to land	31
46	No fee payable	31
47	Restriction on alienation of unallocated State land	31
48	Some agreements run with land	32
49	When does agreement end?	32
50	Amendment of agreements	32
51	When agreement is not needed	33
52	Chief executive may erect signs and carry out works	33
53	Management plans	33

54	Minister may appoint manager	34
55	Trading and commercial activities on designated fossicking land or fossicking areas generally not allowed	34
56	Living on designated fossicking land and fossicking areas	35
57	Conduct on designated fossicking land and fossicking areas	35
**Part 5**	**Camping**	
**Division 1**	**Permits**	
58	Fossickers camping permits	36
59	Applications for fossickers camping permits	36
60	How issuing officer may deal with applications for permit	37
61	Permit conditions must not be contravened	37
62	Permit not transferable	37
63	Term of permit	38
**Division 2**	**Camping**	
64	Division does not apply to some individuals	38
65	Prohibited camping land	38
66	Regulated camping land	38
67	Permit needed to camp on regulated camping land	39
68	Camping on non-regulated designated fossicking land and fossicking areas	40
69	Camping on other land	40
70	Breach of conditions of permission	40
**Part 6**	**Administration and enforcement**	
**Division 1**	**Administration**	
71	Chief executive may appoint issuing officers	41
72	Chief executive may appoint authorised officers	42
73	Limitation of authorised officer's powers	42
74	Authorised officer's conditions of appointment	43
75	Authorised officer's identity card	43
76	Production or display of authorised officer's identity card	44
77	Powers of authorised officer	44
**Division 2**	**General powers of authorised officer**	
78	Dealing with person contravening Act	44
79	Safety of fossicking sites	45
80	Authorised officer may restrict activities	45
81	Production of licence or permit	46

82	Entry of place	46
83	Warrants	47
84	Warrants—applications made other than in person	48
85	Entry of vehicles	49
86	General powers in relation to places and vehicles	50
87	Power to seize evidence	53
88	Power to seize evidence after entering a vehicle	53
89	Procedure after thing seized	54
90	Power to require name and address	55
91	Power to seize weapons etc.	56
**Division 3**	**Offences**	
92	Obstruction of authorised officer	57
93	Re-entering land after direction to leave	57
94	Proceedings for offences	57
**Division 4**	**Proceedings**	
95	Forfeiture on conviction	57
96	Dealing with forfeited things	58
97	Disposal of abandoned property	58
98	Evidentiary provision	60
**Part 7**	**Appeals**	
99	Appeals to Land Court	62
100	Starting appeal	62
101	Stay of operation of decisions etc.	63
102	Hearing procedures	63
103	Powers of Land Court on appeal	63
**Part 8**	**General**	
104	Records to be kept by registrar	64
105	Delegation	65
106	Protection against liability	65
107	Regulation-making power	65
**Part 9**	**Repeal and transitional provisions before Mining and Other Legislation Amendment Act 2013**	
119	References to Mining (Fossicking) Act 1985	66
120	Expiry	66
**Part 10**	**Transitional provisions for Mining and Other Legislation Amendment Act 2013**	

121	Definitions for pt 10	67
122	Continuing effect of general permissions	67
123	Continuing effect of permissions under s 56	67
124	Appeals	67

Fossicking Act 1994

An Act about recreational and tourist fossicking for minerals, gemstones and ornamental stones, and for related purposes

Part 1 Preliminary

1 Short title

This Act may be cited as the *Fossicking Act 1994*.

2 Commencement

This Act commences on a day to be fixed by proclamation.

3 Definitions

In this Act—

authorised officer means—

(a) an authorised officer under the *Mineral Resources Act 1989*; or

(b) a person who is appointed as an authorised officer under section 72(1).

camp includes—

(a) pitch, place or erect a tent, caravan, hut or camping structure; and

(b) place other camping equipment in position; and

(c) stay overnight.

commercial tour means a tour, involving fossicking, conducted as part of a business activity.

Authorised by the Parliamentary Counsel

commercial tour operator means a person who conducts, offers to conduct, agrees to conduct, or arranges for someone else to conduct, a commercial tour.

designated fossicking land means land declared to be designated fossicking land.

Note—

Designated fossicking land declarations are made under section 42(1).

educational organisation means—

(a) a school, college or registered higher education provider; or

(b) a museum; or

(c) a department of government involved in supplying educational services.

exploration permit see section 8(1).

explosive has the meaning given by the *Explosives Act 1999*.

forest entitlement area see section 8(2).

fossick see section 5.

fossicking area means land declared to be a fossicking area.

Note—

Fossicking area declarations are made under section 44(1).

fossicking material means—

(a) a gemstone; or

(b) an ornamental stone; or

(c) a mineral specimen; or

(d) alluvial gold; or

(e) a fossil (other than a fossil of a vertebrate animal); or

(f) a substance prescribed by regulation to be fossicking material;

but does not include a meteorite, tektite, or impact or ejected material associated with a meteorite impact structure.

Authorised by the Parliamentary Counsel

gemstone means a precious stone.

general permission for fossicking or camping, means permission, given under section 28 to the chief executive, for anyone—

(a) to fossick on land mentioned in the permission under a licence; or

(b) to camp on land mentioned in the permission when fossicking on the land under a licence.

government owned corporation see section 8(3).

hand tool means—

(a) a pick, shovel, hammer, sieve, shaker, or electronic detector; or

(b) a tool declared by regulation to be a hand tool.

issuing officer means a person who is appointed as an issuing officer under section 71(1).

known fossil site means land of particular palaeontologic significance because of the presence of fossils on the land.

lake see the *Water Act 2000*, schedule 4.

land manager see section 54(2).

licence means a fossickers licence.

member see section 6.

mineral see section 8(1).

mineral development licence see section 8(1).

mining claim see section 8(1).

mining district see section 8(1).

mining lease see section 8(1).

obstruct includes hinder or resist and attempt to obstruct.

occupied land see section 8(1).

occupier of a place includes a person who reasonably appears to be the occupier, or in charge, of the place.

Authorised by the Parliamentary Counsel

owner see section 8(1).

permit means a fossickers camping permit.

person in control of a vehicle includes the vehicle's driver or the person who reasonably appears to be the vehicle's driver.

place includes premises, but does not include a vehicle.

premises includes—

(a) a building or other structure; and

(b) a part of a building or other structure; and

(c) land where a building or other structure is situated.

prohibited camping land means land declared to be prohibited camping land.

Note—

Regulated camping land declarations are made under section 66(1).

prospect see section 8(1).

prospecting permit see section 8(1).

protected area means—

(a) any of the following under the *Nature Conservation Act 1992*—

 (i) a national park (scientific);

 (ii) a national park;

 (iii) a national park (Aboriginal land);

 (iv) a national park (Torres Strait Islander land);

 (v) a national park (Cape York Peninsula Aboriginal land);

 (vi) a conservation park;

 (vii) a special wildlife reserve; or

(b) an area of regional interest under the *Regional Planning Interests Act 2014*.

Authorised by the Parliamentary Counsel

public place means a place that the public is entitled to use, is open to the public, or is used by the public, whether or not on payment of money.

registered higher education provider see the *Tertiary Education Quality and Standards Agency Act 2011* (Cwlth), section 5.

registrar means the person responsible for keeping a register of interests in land.

regulated camping land means land declared to be regulated camping land.

Note—

Regulated camping land declarations are made under section 66(1).

reserve see section 8(1).

sell includes—

(a) sell by wholesale, retail or auction; and

(b) supply in trade or commerce under an arrangement; and

(c) agree, attempt or offer to sell; and

(d) keep or expose for sale; and

(e) cause or permit to be sold.

State forest see section 8(2).

timber reserve see section 8(2).

watercourse see section 7.

weapon see section 8(4).

4 Meaning of *club*

An entity is a ***club*** under this Act if, under its constitution, it is established for the educational, scientific or recreational study of minerals, rocks or natural history, even though it may also be established for a different purpose.

Examples of a club—

1 a gem or lapidary club

Authorised by the Parliamentary Counsel

2 a geological or gemological association

3 a natural history association

5 Meaning of *fossick*

(1) ***Fossick*** means—

(a) search for fossicking materials in a systematic or unsystematic way—

(i) on the ground's surface; or

(ii) by digging with a hand tool; or

(b) collect fossicking materials.

(2) However, a person does not ***fossick*** merely because the person picks up a specimen of fossicking material the person finds by chance when doing something other than fossicking.

6 Meaning of *member*

(1) ***Member*** of a club means an individual who is a member of the club in any capacity.

(2) ***Member*** of a commercial tour group means—

(a) a person taking part in a commercial tour; or

(b) an operator, or the operator's employee or agent, actually engaged in conducting a commercial tour; or

(c) a person transporting group members on a commercial tour.

(3) ***Member*** of an educational organisation means a staff member or student of the organisation.

(4) ***Member*** of a licensee's family means—

(a) the licensee's spouse; or

(b) a child, or a student under 23, living with the licensee as a member of the licensee's household.

Authorised by the Parliamentary Counsel

7 Meaning of *watercourse*

(1) A ***watercourse*** is a river, creek or stream in which water flows permanently or intermittently.

(2) A river, creek or stream is a ***watercourse*** even if the water flows—

(a) in an artificially improved channel; or

(b) in an artificial channel that has changed the path of the river, creek or stream.

(3) ***Watercourse*** includes the beds and banks of a river, creek or stream, and elements of a river, creek or stream that may confine or contain water.

8 Meaning of expressions used in this and other Acts

(1) The following expressions have the meaning given by the *Mineral Resources Act 1989*—

- exploration permit
- mineral
- mineral development licence
- mining claim
- mining district
- mining lease
- occupied land
- owner
- prospect
- prospecting permit
- reserve.

(2) The following expressions have the meaning given by the *Forestry Act 1959*—

- forest entitlement area
- State forest

Authorised by the Parliamentary Counsel

- timber reserve.

(3) ***Government owned corporation*** has the meaning given by the *Government Owned Corporations Act 1993*.

(4) ***Weapon*** has the meaning given by the *Weapons Act 1990*.

Part 2 Land excluded from Act’s application

9 Act’s application to protected areas

This Act does not apply to a protected area.

10 Act’s application to State forests, timber reserves and forest entitlement areas

This Act applies to a State forest, timber reserve or forest entitlement area that is not part of a protected area only if—

(a) the forest, reserve or area becomes designated fossicking land or a fossicking area; or

(b) the chief executive of the department in which the *Forestry Act 1959* is administered gives a general permission for fossicking or camping in the forest, reserve or area.

12 Other land may be excluded from operation of Act

A regulation may exclude land from the Act’s operation.

Editor’s note—

The land may, for example, contain rare or significant minerals, be in an environmentally sensitive area, a catchment area for a reservoir or an urban area, or have major public works on it.

13 Act’s application to scientific organisations

This Act does not apply to fossicking, for a scientific or research purpose, by or for—

Authorised by the Parliamentary Counsel

(a) the Queensland Museum; or

(b) a scientific organisation actually engaged in a geoscience research program.

Part 3 Licences

Division 1 Licences

14 Licences

An issuing officer may grant the following kinds of fossickers licences—

(a) individual fossickers licences;

(b) family fossickers licences;

(c) club fossickers licences;

(d) educational organisation fossickers licences;

(e) commercial tour operator fossickers licences;

(f) another kind of fossickers licence prescribed by regulation.

15 Applications

(1) An application for a licence must be made to an issuing officer for the land mentioned in the application.

(2) The application must be in the approved form and accompanied by the fee prescribed by regulation for the licence.

(3) However, if the chief executive, by gazette notice, directs that an application for a licence for a stated mining district, designated fossicking land or fossicking area must be made to a stated issuing officer, an application of that kind must be made to the stated issuing officer.

Authorised by the Parliamentary Counsel

Note—

Generally, an issuing officer may grant a licence for all land other than land excluded from the Act's application under part 2. However, in some cases, the issuing officer will only be able to grant a licence for a limited area. If the issuing officer can not deal with the application, the applicant must be told where to apply for the licence.

16 How issuing officer may deal with applications for licence

(1) An issuing officer must consider an application for a licence and either—

(a) grant the licence, with or without conditions; or

(b) refuse to grant the licence.

(2) If the terms of the issuing officer's appointment prevent the officer granting the licence, the officer—

(a) must not grant the licence; and

(b) must tell the applicant where to apply for the licence.

(3) The issuing officer must not grant a licence to—

(a) a child; or

(b) an authorised officer; or

(c) someone who held a licence cancelled not more than 2 years before the day of the application.

(4) If the issuing officer decides to grant the licence, the officer must promptly give the applicant—

(a) the licence; and

(b) if a condition is stated on the licence—a written notice stating—

(i) the reasons for the condition; and

(ii) that the applicant may appeal against the imposition of the condition within 28 days to the Land Court.

Authorised by the Parliamentary Counsel

(5) If the issuing officer decides not to grant the licence, the officer must promptly give the applicant a written notice stating—

(a) the decision; and

(b) the reasons for the decision; and

(c) that the applicant may appeal against the decision within 28 days to the Land Court.

17 License conditions must not be contravened

A licensee must not contravene the licence conditions.

Maximum penalty—20 penalty units.

18 Licence not transferable

A licence is not transferable.

19 Term of licence

(1) A licence is for the term, of not more than 1 year, stated in the licence.

(2) A regulation may authorise the issue of a stated kind of licence for a stated shorter term.

20 Replacement licences

(1) A licensee may apply to an issuing officer for the replacement of a lost, stolen or destroyed licence.

(2) The issuing officer must consider the application and either—

(a) replace the licence; or

(b) refuse to replace the licence.

(3) If the issuing officer is satisfied a licence has been lost, stolen or destroyed, the officer must replace the licence.

Authorised by the Parliamentary Counsel

(4) If the issuing officer decides to refuse to replace the licence, the officer must give the applicant a written notice stating—

(a) the decision; and

(b) the reasons for the decision; and

(c) that the applicant may appeal against the decision within 28 days to the Land Court.

21 Suspension and cancellation of licences—grounds

A ground for the suspension or cancellation of a licence exists if—

(a) the licensee has contravened a provision of this Act or a condition of the licence; or

(b) the licence was obtained by fraud or misrepresentation.

22 Suspension and cancellation of licences—procedures

(1) If the chief executive considers there is a ground to suspend or cancel a licence (the ***proposed action***), the chief executive may give the licensee a written notice—

(a) stating the proposed action; and

(b) stating the grounds for the proposed action; and

(c) outlining the facts and circumstances forming the basis of the grounds; and

(d) if the proposed action is suspension of the licence—stating the proposed suspension period; and

(e) inviting the licensee to show, within a stated time of at least 28 days, why the proposed action should not be taken.

(2) If, after considering all written representations made within the stated time, the chief executive still considers there is a ground to take the proposed action, the chief executive may—

Authorised by the Parliamentary Counsel

(a) if the proposed action was to suspend the licence for a stated period—suspend the licence for not longer than the proposed suspension period; or

(b) if the proposed action was to cancel the licence—either cancel the licence or suspend it for a period.

(3) The chief executive must inform the licensee of the decision by written notice.

(4) If the chief executive decides to suspend or cancel the licence, the notice must state—

(a) the reasons for the decision; and

(b) that the licensee may appeal against the decision within 28 days to the Land Court.

(5) The decision takes effect on the later of—

(a) the day when notice is given to the licensee; or

(b) the day of effect stated in the notice.

(6) However, if the licence is suspended or cancelled because of the conviction of a person for an offence—

(a) the suspension or cancellation does not take effect until—

(i) the end of the time to appeal against the conviction; and

(ii) if an appeal is made against the conviction—the appeal is finally decided; and

(b) the suspension or cancellation has no effect if the conviction is quashed on appeal.

23 Return of licence

(1) The holder of a suspended licence, or the former holder of a cancelled licence, must return the licence to the chief executive within 7 days after the suspension or cancellation takes effect, unless the person has a reasonable excuse for not returning it or not returning it within that time.

Authorised by the Parliamentary Counsel

Maximum penalty—20 penalty units.

(2) If a suspended licence is returned to the chief executive, the chief executive must return it to the licensee at the end of the suspension period.

Division 2 Licensees' entitlements and duties

24 Meaning of *licensee* in division

In this division—

licensee includes a member of a club, commercial tour group, educational organisation or licensee's family.

25 Licence needed to fossick

(1) A person must not fossick for fossicking materials unless the person—

(a) holds a licence; or

(b) is a member of a club holding a licence; or

(c) is taking part in a commercial tour under the commercial tour operator's fossickers licence; or

(d) is a member of—

(i) an educational organisation holding a licence; or

(ii) a licensee's family.

Maximum penalty—50 penalty units.

(2) Subsection (1) does not apply to a person fossicking on land to which a mining claim or mining lease applies—

(a) on payment to the claim or lease holder of an admission fee; or

(b) with the claim or lease holder's permission, for a mineral for which the claim or lease is granted.

Authorised by the Parliamentary Counsel

26 Licensee's right to enter unoccupied land to fossick

(1) Despite any other Act, a licensee may enter and fossick on unoccupied land.

(2) However, a person must not fossick on excluded land.

Maximum penalty—100 penalty units.

(3) In this section—

excluded land means land excluded from the Act's operation under part 2.

unoccupied land means land other than occupied land.

27 Licensee must get permission to fossick on occupied land etc.

(1) A licensee must not fossick—

(a) on occupied land (other than a road reserve, designated fossicking land or a fossicking area) without the owner's written permission; or

(b) on land to which a mining claim or mining lease applies without the claim or lease holder's written permission; or

(c) on land where a person may take quarry materials under a quarry materials permit without the permit holder's written permission; or

(d) on land the subject of an exclusive possession determination without the written permission of the native title holders for the determination.

Maximum penalty—50 penalty units.

(2) Permission under subsection (1)—

(a) may be given on conditions; and

(b) must be written on or attached to the licensee's licence.

(3) If the owner, claim, lease or permit holder, or native title holder, (the ***permitter***) withdraws the permission, the

Authorised by the Parliamentary Counsel

permitter must give a licensee on the land reasonable written notice of its withdrawal.

(4) If the licensee does not leave the land within the reasonable period stated in the notice, the licensee commits an offence against this Act.

Maximum penalty—50 penalty units.

(5) Subsection (4) does not affect a right or remedy the permitter may have against a licensee apart from this section.

(6) In this section—

exclusive possession determination, for land, means an approved determination of native title under the *Native Title Act 1993* (Cwlth) that includes a determination to the effect that native title rights and interests under the determination confer possession of the land on native title holders to the exclusion of all others.

quarry materials includes controlled quarry materials under the *Water Act 2000*.

quarry materials permit means—

(a) an agreement, contract, licence, permit, or other authority under the *Forestry Act 1959*; or

(b) an allocation of quarry material under the *Water Act 2000*.

28 General permissions

(1) A person whose permission is needed to allow someone else to fossick on land may give the chief executive permission (***general permission***) for—

(a) fossicking on the land; and

(b) camping on the land by persons fossicking under a licence.

(2) If a person gives a general permission for fossicking on particular land, a licensee does not need the person's written permission to fossick on the land.

Authorised by the Parliamentary Counsel

(3) If a person gives a general permission for camping on particular land by persons fossicking on the land under a licence, a licensee does not need the person's written permission under the following provisions to camp on the land.

Note—

Unless a general permission for camping is given, written permission is needed for camping on non-regulated designated fossicking land and fossicking areas, and other areas on which a person fossicking under a licence may camp.

(4) However, if the general permission requires the licensee to get another type of permission before entering the land, the licensee must get the other permission before entering the land.

29 Licensee must comply with conditions of permission

(1) This section applies if permission under this Act to fossick on land is given on conditions.

(2) A licensee must comply with the conditions of the permission.

(3) If a licensee does not comply with a condition of the permission—

(a) the licensee's right to remain on the land ends; and

(b) an authorised officer, or the person who gave the permission, may require the licensee to leave the land immediately.

(4) If the licensee does not immediately leave the land, the licensee commits an offence against this Act, unless the licensee has a reasonable excuse.

Maximum penalty—50 penalty units.

(5) This section does not affect a right or remedy the person who gave the permission may have against a licensee apart from this section.

Authorised by the Parliamentary Counsel

30 Commercial tour operator sometimes needs other permissions

A commercial tour operator's fossickers licence does not replace the need for a licence, permit or authority under another Act to enter occupied land.

31 Other limits on commercial tour operator's fossicker licence

A commercial tour operator must not conduct a commercial tour in which—

(a) the members of the commercial tour group are allowed to fossick in more than 1 area at the same time; or

(b) there are more than 50 members of the commercial tour group who are allowed to fossick under the licence at the same time; or

(c) members of the commercial tour group are allowed to fossick for fossils.

Maximum penalty—20 penalty units.

32 Limits on club and educational organisation fossickers licence

(1) A club must not allow—

(a) the club's members to fossick under the club's licence in more than 1 area at the same time; or

(b) more than 50 members to fossick under the club's licence at the same time.

(2) An educational organisation must not allow—

(a) the organisation's members to fossick under the organisation's licence in more than 1 area at the same time; or

(b) more than 50 members to fossick under the organisation's licence at the same time.

Maximum penalty—20 penalty units.

Authorised by the Parliamentary Counsel

33 Records of land mentioned in general permission to be kept

The chief executive must keep records of general permissions for land available for inspection at the places the chief executive considers appropriate.

Division 3 General

34 When fossicking materials become licensee's property

(1) If fossicking materials lawfully collected under a licence are the property of the State, the materials become the licensee's property on collection.

(2) However, the licensee must pay royalties on the fossicking materials.

(3) Subsection (1) applies despite the following provisions—

- *Mineral Resources Act 1989*, section 8.
- *Forestry Act 1959*, section 45
- *Water Act 2000*, section 279.

35 Payment of royalties to State

(1) This Act does not affect a requirement under another Act to lodge royalty returns for, and pay royalties on, fossicking materials collected by a licensee.

(2) However, despite the *Mineral Resources Act 1989*, a licensee need not file a royalty return under the Act for minerals collected by the licensee in a royalty period if no royalty is payable under the Act for the minerals for the period.

(3) In this section—

licensee means—

(a) the holder of an individual or family fossickers licence; or

(b) an individual fossicking under another kind of fossickers licence.

36 Sale or use of fossicking material in trade or commerce

(1) This section applies to fossicking material collected under a licence.

(2) A licensee must not—

(a) in trade or commerce, sell the material; or

(b) use the material in the production of something else for sale in trade or commerce.

Maximum penalty—400 penalty units.

(3) However, this section does not apply to—

(a) an occasional sale or use of fossicking material; or

(b) a sale or use prescribed by regulation.

37 Volume, weight or number of specimens may be restricted

(1) A regulation may restrict the volume, weight or number of fossicking material specimens an individual may collect on particular land.

(2) A licensee must not contravene a restriction prescribed by regulation.

Maximum penalty—50 penalty units.

38 Use of machinery etc. prohibited

A person fossicking under a licence must not use machinery or equipment (other than a hand tool) to fossick.

Maximum penalty—400 penalty units.

Authorised by the Parliamentary Counsel

39 Limits on digging

(1) A person fossicking under a licence must not dig below ground level—

(a) to a depth, measured from the highest point at the top of the land dug, of more than—

(i) 0.5m in a watercourse; or

(ii) 2m on other land; or

(iii) a depth fixed under subsection (2); or

(b) if the digging involves tunnelling under land or creating an overhang; or

(c) in a road reserve.

Maximum penalty—20 penalty units.

(2) If, in the Minister's opinion, digging to a depth mentioned in subsection (1)(a)(i) or (ii) on particular land may be unsafe, the Governor in Council may fix a reduced depth for the land by regulation.

40 Discovery of minerals does not confer rights

To remove any doubt, a licence does not give a person who discovers a mineral deposit while fossicking a right to do anything other than fossick for the mineral in the deposit under the licence.

Note—

The licensee must get a permit, claim, licence or lease under the *Mineral Resources Act 1989* to be able to develop the mineral deposit for commercial purposes and has no prior right to a permit, claim, licence or lease merely because of the discovery.

Authorised by the Parliamentary Counsel

Part 4 Designated fossicking land and fossicking areas

Division 1 Designated fossicking land

41 Proposal for declaration of designated fossicking land

(1) If the chief executive is satisfied particular land should be declared designated fossicking land, the chief executive must prepare a proposal for the declaration.

(2) The proposal must describe the land proposed to become designated fossicking land.

(3) The chief executive must give written notice to owners of land proposed to become designated fossicking land and the local government for the land.

(4) The notice must state a day by which an owner or the local government may make submissions to the chief executive about the proposal.

(5) The chief executive may give notice of the proposal in the newspaper the chief executive considers appropriate if—

(a) the chief executive considers it is impracticable to give notice to each owner of a particular class; or

(b) after making the inquiries the chief executive considers appropriate, an owner's name can not easily be found out.

(6) In this section—

owner includes a person having an interest in the land.

42 Declaration of designated fossicking land

(1) A regulation may declare particular land to be designated fossicking land, and give the land a name.

Authorised by the Parliamentary Counsel

(2) A regulation must not make a declaration for occupied land without the owner’s written agreement or for a known fossil site.

(3) The agreement of 1 owner who is a joint tenant or tenant in common with other owners, is, in the absence of evidence to the contrary, taken to be agreement of each other owner who is a joint tenant or tenant in common for subsection (2).

(4) A declaration under subsection (1) does not affect—

(a) the rights under the *Mineral Resources Act 1989* of a holder of a prospecting permit, exploration permit, mineral development licence, mining claim or mining lease for land stated in the declaration; or

(b) a right obtained under the *Mineral Resources Act 1989* after the declaration, under an application made before the declaration, for a permit, licence, claim or lease mentioned in paragraph (a) for land stated in the declaration.

Division 2 Fossicking areas

43 Proposal for declaration of fossicking area

(1) If the chief executive is satisfied particular land should be declared a fossicking area, the chief executive must prepare a proposal for the declaration.

Note—

A fossicking area differs from designated fossicking land. An interest under the *Mineral Resources Act 1989* may be granted for land in a fossicking area only if an application for the interest is made before the land becomes a fossicking area or there is an existing interest under that Act. Further interests can be granted for designated fossicking land.

(2) The proposal must describe the land proposed to become a fossicking area.

(3) The chief executive must give written notice to the following persons—

Authorised by the Parliamentary Counsel

(a) owners of land proposed to become a fossicking area;

(b) applicants for, and holders of, an exploration permit, mineral development licence, mining claim or mining lease for the land;

(c) the local government for the land.

(4) The notice must state a day by which a person mentioned in subsection (3) may make submissions to the chief executive about the proposal.

(5) The chief executive may give notice of the proposal in the newspaper the chief executive considers appropriate if—

(a) the chief executive considers it is impracticable to give notice to each owner of a particular class; or

(b) after making the inquiries the chief executive considers appropriate, an owner's name can not easily be found out.

(6) In this section—

owner includes a person having an interest in the land.

44 Declaration of fossicking area

(1) A regulation may declare particular land to be a fossicking area, and give the area a name.

(2) A regulation must not make a declaration—

(a) for occupied land—without the owner's written agreement; or

(b) for land in an exploration permit, mineral development licence, mining claim or mining lease—without written agreement of the permit, licence, claim or lease holder; or

(c) for land in an application under the *Mineral Resources Act 1989* for an exploration permit, mineral development licence, mining claim or mining lease that has not been rejected or withdrawn when the declaration is made—without the applicant's written agreement; or

Authorised by the Parliamentary Counsel

(d) for a known fossil site.

(3) However, a declaration may be made for land in an application mentioned in subsection (2)(c) for which the applicant's agreement was not obtained if the application is rejected or withdrawn before the declaration is made.

(4) The agreement of 1 owner who is a joint tenant or tenant in common with other owners, is, in the absence of evidence to the contrary, taken to be agreement of each other owner who is a joint tenant or tenant in common for subsection (2)(a).

Division 3 General

45 Declaration does not normally affect person's title to land

A declaration of land as designated fossicking land or a fossicking area does not affect a person's title to land unless the owner's agreement to the declaration contains terms binding on the State and the owner and the owner's successors in title.

Note—

Under section 48 (Some agreements run with land) an owner's agreement to a declaration of land as designated fossicking land or a fossicking area may be made binding on the State, the owner and successors in title.

46 No fee payable

An owner of designated fossicking land or land in a fossicking area must not charge a fee for fossicking on the land.

Maximum penalty—10 penalty units.

47 Restriction on alienation of unallocated State land

(1) This section applies if unallocated State land under the *Land Act 1994* is part of designated fossicking land or a fossicking area.

Authorised by the Parliamentary Counsel

(2) Unallocated State land mentioned in subsection (1) must not be alienated, leased or occupied without the Minister's approval.

(3) However, failure to obtain the Minister's approval does not invalidate an alienation, lease or occupancy.

48 Some agreements run with land

An owner's agreement to the declaration of land as designated fossicking land or a fossicking area may contain terms binding on the State, the owner and the owner's successors in title.

49 When does agreement end?

(1) An owner's agreement to the declaration of land as designated fossicking land or a fossicking area has effect until it ends under its terms unless—

(a) it is earlier ended under subsection (2); or

(b) the declaration of the land as designated fossicking land or a fossicking area is repealed.

(2) The agreement may be ended if—

(a) the owner asks for it to be ended; or

(b) the Minister considers the land to which the agreement applies is no longer needed, or suitable for use under the agreement, as designated fossicking land or a fossicking area.

50 Amendment of agreements

An owner's agreement to the declaration of land as designated fossicking land or a fossicking area may be amended by a later agreement between the State and the owner, including, for example, by removing from the designated fossicking land or fossicking area, if the owner asks, land no longer needed, or

Authorised by the Parliamentary Counsel

suitable for use as designated fossicking land or a fossicking area.

51 When agreement is not needed

If land that is already designated fossicking land or a fossicking area is proposed to be included with other land in a later declaration of land as designated fossicking land or a fossicking area, the owner's agreement to the inclusion of the land in land to be covered by the declaration is not needed.

52 Chief executive may erect signs and carry out works

(1) The chief executive may erect signs and carry out works on designated fossicking land and fossicking areas for administrative purposes, hygiene reasons or public enjoyment and protection.

(2) However, if the land is occupied land, the chief executive must not carry out works (other than erecting a sign) on the land without the owner's consent.

(3) A sign does not affect an owner's access to the land or prevent the exercise of the owner's rights on the land.

53 Management plans

(1) The chief executive may prepare a draft management plan for designated fossicking land or a fossicking area if, in the chief executive's opinion, the use of the designated fossicking land or fossicking area should be regulated because of the extent of use of, or the impact of activities on, the land.

(2) In preparing the draft management plan, the chief executive must consult with landowners, any land manager for the land, the local government for the land and anyone else the chief executive considers may be affected by the plan.

(3) In preparing a final management plan, the chief executive must consider submissions properly made to the chief executive.

Authorised by the Parliamentary Counsel

(4) If the chief executive approves a final management plan, the chief executive must ensure the approved plan is available for public inspection at the places the chief executive considers appropriate.

54 Minister may appoint manager

(1) This section applies to land that is designated fossicking land or a fossicking area (the ***land***).

(2) The Minister may, with the owner's approval, appoint a manager for the land (the ***land manager***).

(3) The terms of the appointment are as stated in the instrument of appointment.

(4) The Minister must not appoint a person as the land manager if the owner does not agree to the person's appointment.

(5) The land manager must—

(a) care for and administer the land; and

(b) ensure the management plan for the land is properly carried out; and

(c) keep records of all amounts received by the land manager under this Act; and

(d) account to the chief executive for the amounts.

(6) The *Public Sector Management and Employment Act 1988* does not apply to the appointment or employment of the land manager.

55 Trading and commercial activities on designated fossicking land or fossicking areas generally not allowed

(1) A person must not carry out a trading or commercial activity on designated fossicking land or a fossicking area.

Maximum penalty—400 penalty units.

Authorised by the Parliamentary Counsel

(2) Subsection (1) does not apply to lawful activities by or for the owner of the land or the holder of a mining claim or lease over the land.

(3) Also, subsection (1) does not prevent a commercial tour group visiting the land under a commercial tour operator's fossicking licence.

56 Living on designated fossicking land and fossicking areas

(1) A person must not live on designated fossicking land or a fossicking area without the chief executive's written permission.

Maximum penalty—400 penalty units.

(2) Subsection (1) does not apply to—

(a) an owner of occupied land in designated fossicking land or a fossicking area; or

(b) a member of the owner's family, a lessee or sub-lessee from the owner, or anyone living on the land with the owner's permission; or

(c) the holder of a mining claim or lease; or

(d) an agent or employee of the holder of a mining claim or lease; or

(e) anyone else living on the land when it became designated fossicking land or a fossicking area.

57 Conduct on designated fossicking land and fossicking areas

A person on designated fossicking land or a fossicking area must not—

(a) obstruct a person lawfully on the land or area, unless the person has a reasonable excuse; or

(b) be disorderly; or

Authorised by the Parliamentary Counsel

(c) create a disturbance, unless the person has a reasonable excuse.

Maximum penalty—20 penalty units.

Part 5 Camping

Division 1 Permits

58 Fossickers camping permits

(1) An issuing officer may grant the following kinds of fossickers camping permits—

(a) individual fossickers camping permits;

(b) family fossickers camping permits;

(c) club fossickers camping permits;

(d) educational organisation fossickers camping permits;

(e) commercial tour operator fossickers camping permits;

(f) another kind of fossickers camping permit prescribed by regulation.

(2) A regulation may provide for camping under self-registration procedures provided in the regulation.

59 Applications for fossickers camping permits

(1) An application for a permit must be made to an issuing officer for the land to which the application applies.

(2) The application must be in the approved form and accompanied by the fee prescribed by regulation for the permit.

(3) However, if the chief executive, by gazette notice, directs that an application for a permit for particular designated fossicking land or a particular fossicking area must be made to a

particular issuing officer, an application of that kind must be made to that issuing officer.

(4) If a person may camp on particular land under self-registration procedures, this section does not prevent the person applying for a permit under the self-registration procedures.

60 How issuing officer may deal with applications for permit

(1) An issuing officer must consider an application for a permit and either—

(a) grant the permit, with or without conditions; or

(b) refuse to grant the permit.

(2) If the terms of the issuing officer's appointment prevent the officer granting the permit, the officer—

(a) must not grant the permit; and

(b) must tell the applicant where to apply for the permit.

(3) If the issuing officer decides to grant the permit, the officer must promptly give the applicant the permit.

(4) However, the issuing officer must refuse to grant a permit for camping on regulated camping land if granting it would allow the applicant to camp continuously on the same regulated camping land for longer than the maximum term of a permit.

61 Permit conditions must not be contravened

The permit holder and anyone else camping under the permit must comply with the permit conditions.

Maximum penalty—20 penalty units.

62 Permit not transferable

A permit is not transferable.

Authorised by the Parliamentary Counsel

63 Term of permit

(1) A permit is for the term stated in the permit.

(2) A regulation may prescribe a maximum term for permits.

Division 2 Camping

64 Division does not apply to some individuals

This division does not apply to camping on designated fossicking land or a fossicking area if the person camping on the land is—

(a) the owner; or

(b) an agent of, or lessee or sub-lessee from, the owner; or

(c) someone else camping on the owner's land with the owner's permission; or

(d) the holder of a mining claim or mining lease over the land where the person is camping; or

(e) an agent of a mining claim or lease holder of the land where the person is camping.

65 Prohibited camping land

(1) A regulation may declare designated fossicking land or a fossicking area to be prohibited camping land.

(2) A person must not camp on prohibited camping land.

Maximum penalty for subsection (2)—20 penalty units.

66 Regulated camping land

(1) A regulation may declare designated fossicking land or a fossicking area to be regulated camping land.

(2) A regulation must not declare land to be regulated camping land without the agreement of—

Authorised by the Parliamentary Counsel

(a) if camping on the land is regulated under a local law—the local government for the land; or

(b) if the land is occupied land—the owner of the land.

(3) If land mentioned in subsection (2)(a) or (b), or land on which camping is regulated under another Act, becomes regulated camping land—

(a) a provision of the Act or local law regulating camping on the land ceases to apply to the land to the extent stated in the regulation; and

(b) no camping fee is payable to the owner by persons camping on the land under this Act.

67 Permit needed to camp on regulated camping land

(1) A person must not camp on regulated camping land unless the person—

(a) holds a permit; or

(b) is a member of a club holding a permit; or

(c) is taking part in a commercial tour under the commercial tour operator's permit; or

(d) is a member of—

(i) an educational organisation holding a permit; or

(ii) a permit holder's family.

(2) Also, if the regulated camping land is land to which a mining claim or lease applies, the person must not camp on the land without the claim or lease holder's permission.

Maximum penalty—20 penalty units.

Note—

A permission mentioned in this section and sections 68 and 69 includes a general permission under section 28 (General permissions).

Authorised by the Parliamentary Counsel

68 Camping on non-regulated designated fossicking land and fossicking areas

(1) This section applies to camping on designated fossicking land or a fossicking area (other than prohibited camping land and regulated camping land).

(2) If the land is occupied land or land to which a mining claim or lease applies, a person fossicking under a licence must not camp on the land without the owner's or claim or lease holder's permission.

Maximum penalty—20 penalty units.

69 Camping on other land

(1) This section applies to camping on land other than designated fossicking land and a fossicking area.

(2) If the land is occupied land or land to which a mining claim or mining lease applies, a person fossicking under a licence must not camp on the land without the owner's, or claim or lease holder's, permission.

Maximum penalty—20 penalty units.

70 Breach of conditions of permission

(1) This section applies to a person camping on land (other than prohibited camping land) while fossicking on the land under a licence.

(2) If the person does not comply with the conditions of the permission to camp on the land—

(a) the person's right to remain on the land ends; and

(b) an authorised officer, the landowner, claim holder or lease holder may require the person to leave the land immediately.

(3) If the person does not leave the land immediately, the person commits an offence against this Act.

Maximum penalty—50 penalty units.

Authorised by the Parliamentary Counsel

(4) This section does not affect a right or remedy the owner may have against the person apart from this section.

Part 6 Administration and enforcement

Division 1 Administration

71 Chief executive may appoint issuing officers

(1) The chief executive may appoint an appropriate person as an issuing officer under this Act.

(2) The chief executive may, in the appointment—

(a) authorise the person to grant a licence or permit for any land to which this Act applies, or a particular mining district, designated fossicking land or fossicking area; or

(b) limit the person's authority to grant a licence or permit to—

(i) a particular kind of licence or permit; or

(ii) a shorter term (stated in the appointment) than the maximum term prescribed by regulation for the licence or permit; or

(c) authorise employees of the person to grant licences and permits of a particular kind.

(3) The chief executive may authorise an issuing officer (other than an officer of the public service or member or employee of a local government) to charge commission or keep a specified part of the prescribed fee for granting a licence or permit.

(4) The commission that may be charged, or part of the prescribed fee that may be kept, must be fixed by regulation.

Authorised by the Parliamentary Counsel

72 Chief executive may appoint authorised officers

(1) The chief executive may appoint any of the following persons as authorised officers—

(a) officers and employees of the public service;

(b) employees of a government owned corporation;

(c) employees of a rail government entity under the *Transport Infrastructure Act 1994*;

(d) members or employees of a local government;

(e) other persons prescribed by regulation.

(2) The chief executive may appoint a person as an authorised officer only if—

(a) in the chief executive's opinion, the person has the necessary experience or expertise to be an authorised officer; or

(b) the person has satisfactorily finished training approved by the chief executive.

(3) If the person is a member or employee of a local government, the chief executive must not make the appointment without the agreement of the local government's chief executive officer.

(4) The chief executive may restrict an authorised person's powers by written notice given to the person.

73 Limitation of authorised officer's powers

The powers of an authorised officer may be limited—

(a) under a regulation; or

(b) under a condition of appointment; or

(c) by written notice of the chief executive given to the authorised officer.

Authorised by the Parliamentary Counsel

74 Authorised officer's conditions of appointment

(1) An authorised officer holds office on the conditions specified in the instrument of appointment.

(2) An authorised officer—

(a) if the appointment provides for a term of appointment—ceases holding office at the end of the term; and

(b) may resign by signed notice of resignation given to the chief executive; and

(c) if the conditions of appointment provide—ceases holding office as an authorised officer on ceasing to hold another office stated in the conditions of appointment.

(3) In this section—

authorised officer means a person appointed as an authorised officer.

75 Authorised officer's identity card

(1) The chief executive must give each authorised officer an identity card.

(2) The identity card must—

(a) contain a recent photograph of the authorised officer; and

(b) be signed by the authorised officer; and

(c) identify the person as an authorised officer under this Act.

(3) A person who ceases to be an authorised officer must, as soon as practicable, but within 21 days after ceasing to hold the office, return the identity card to the chief executive, unless the person has a reasonable excuse for not returning it.

Maximum penalty—20 penalty units.

(4) This section does not apply to an officer of another department or an employee of a government owned

Authorised by the Parliamentary Counsel

corporation who holds an identity card issued by that department or corporation.

(5) Nothing in this section prevents the issue of a single identity card to a person for this and other Acts.

76 Production or display of authorised officer's identity card

(1) An authorised officer may exercise a power in relation to someone else only if the officer—

(a) first produces his or her identity card for the other person's inspection; or

(b) has his or her identity card displayed so that it is clearly visible to the person.

(2) However, if, for any reason, it is not practicable to comply with subsection (1), the authorised officer must produce the identity card for the person's inspection at the first reasonable opportunity.

77 Powers of authorised officer

(1) An authorised officer has the powers given under this or another Act.

(2) A regulation may limit the powers of authorised officers.

Division 2 General powers of authorised officer

78 Dealing with person contravening Act

(1) If an authorised officer believes on reasonable grounds a person is contravening or has just contravened a provision of this Act, the officer may direct the person—

(a) to leave the land within a stated reasonable time; or

(b) if the officer believes on reasonable grounds the contravention is serious—to leave the land immediately;

Authorised by the Parliamentary Counsel

and not to re-enter the land for a stated reasonable period of not more than 7 days.

(2) The person must comply with a direction given to the person under subsection (1), unless the person has a reasonable excuse for not complying with it.

Maximum penalty—50 penalty units.

(3) Subsection (1)(b) does not apply to the owner of the land.

(4) A permit held by a person who is directed to leave land under this section is cancelled by force of this subsection when the person is required to have left the land.

79 Safety of fossicking sites

(1) An authorised officer or an inspector under the *Mining and Quarrying Safety and Health Act 1999* may direct a person fossicking under a licence to take stated reasonable steps within a stated reasonable time to make land used by the person safe.

(2) The person must comply with a direction given to the person under subsection (1), unless the person has a reasonable excuse for not complying with it.

Maximum penalty—20 penalty units.

80 Authorised officer may restrict activities

(1) An authorised officer may prohibit fossicking, camping or the use of fire on designated fossicking land or a fossicking area for a stated period by notice displayed on the land if the officer considers it necessary or desirable—

(a) for safety or hygiene reasons; or

(b) to allow land to be rehabilitated; or

(c) to prevent inconvenience to anyone on the land.

Examples of paragraph (c)—

1 there may be too many people fossicking on the land

Authorised by the Parliamentary Counsel

2 the owner may want to muster stock on the land

(2) A person must comply with a notice displayed under subsection (1), unless the person has a reasonable excuse for not complying with it.

Maximum penalty—20 penalty units.

81 Production of licence or permit

(1) An authorised officer may ask a person apparently fossicking under a licence or camping on regulated camping land to produce the person's licence or permit immediately for inspection.

(2) The person must produce the licence or permit, unless the person has a reasonable excuse for not producing it.

Maximum penalty—5 penalty units.

82 Entry of place

An authorised officer or an inspector under the *Mining and Quarrying Safety and Health Act 1999* may enter a place if—

(a) it is designated fossicking land or a fossicking area and the land entered is not part of a living area; or

(b) a general permission to fossick or camp on the land is in force and the land entered is not part of a living area; or

(c) its occupier consents to the entry or the purpose of the entry is to get the occupier's consent; or

(d) it is a public place and the entry is made when it is open to the public; or

(e) it is—

(i) not within a city or town under the *Local Government Act 2009* and is used for rural purposes; or

(ii) town land used for rural purposes or an extractive industry; or

(iii) vacant town land;

and, in all the circumstances, it is not reasonably practicable to obtain the consent of the owner of the place; or

(f) the entry is authorised by a warrant.

83 Warrants

(1) An authorised officer may apply to a magistrate for a warrant for a place.

(2) An application must be sworn and state the grounds on which the warrant is sought.

(3) The magistrate may refuse to consider the application until the authorised officer gives the magistrate all the information the magistrate requires about the application in the way the magistrate requires.

Example—

The magistrate may require additional information supporting the application to be given by statutory declaration.

(4) The magistrate may issue a warrant only if the magistrate is satisfied there are reasonable grounds for suspecting—

(a) there is a particular thing (the ***evidence***) that may provide evidence of the commission of an offence against this Act; and

(b) the evidence is, or may be within the next 7 days, at the place.

(5) The warrant must state—

(a) that the authorised officer may, with necessary and reasonable help and force, enter the place or vehicle and exercise the authorised officer's powers under this Act; and

(b) the evidence for which the warrant is issued; and

(c) the hours of the day when entry may be made; and

Authorised by the Parliamentary Counsel

(d) the day (within 14 days after the warrant's issue) when the warrant ends.

84 Warrants—applications made other than in person

(1) An authorised officer may apply for a warrant by phone, fax, radio or another form of communication if the officer considers it necessary because of—

(a) urgent circumstances; or

(b) other special circumstances, including, for example, the authorised officer's remote location.

(2) Before applying for the warrant, the authorised officer must prepare an application stating the grounds on which the warrant is sought.

(3) The authorised officer may apply for the warrant before the application is sworn.

(4) After issuing the warrant, the magistrate must immediately fax a copy to the authorised officer if it is reasonably practicable to fax the copy.

(5) If it is not reasonably practicable to fax a copy of the warrant to the authorised officer—

(a) the magistrate must—

(i) tell the authorised officer what the terms of the warrant are; and

(ii) tell the authorised officer the date and time the warrant was signed; and

(iii) record on the warrant the reasons for issuing the warrant; and

(b) the authorised officer must—

(i) complete a form of warrant (***warrant form***) in the same terms as the warrant issued by the magistrate; and

Authorised by the Parliamentary Counsel

(ii) write on the warrant form the name of the magistrate and the date and time the magistrate signed the warrant.

(6) The facsimile warrant, or the warrant form properly completed by the authorised officer, is authority for the entry and the exercise of the other powers authorised by the warrant issued by the magistrate.

(7) The authorised officer must send to the magistrate—

(a) the sworn application; and

(b) if a warrant form was completed by the officer—the completed warrant form.

(8) The sworn application form and any completed warrant form must be sent to the magistrate at the earliest practicable opportunity.

(9) When the magistrate receives the application and any warrant form, the magistrate must attach them to the warrant issued by the magistrate.

(10) If—

(a) in a proceeding a question arises whether the exercise of a power was authorised by a warrant issued under this section; and

(b) the warrant is not produced in evidence;

the court must presume the exercise of a power was not authorised by a warrant unless the contrary is proved.

85 Entry of vehicles

(1) An authorised officer may enter a vehicle if the authorised officer has reasonable grounds for suspecting—

(a) the vehicle is being, or has been, used in the commission of an offence against 1 of the following provisions—

- section 36
- section 37

Authorised by the Parliamentary Counsel

- section 38
- section 55
- section 91(3); or

(b) the vehicle, or a thing in or on the vehicle, may provide evidence of the commission of an offence against a provision mentioned in paragraph (a).

(2) If the vehicle is moving or about to move, the authorised officer may signal the person in control of the vehicle to stop the vehicle or not to move it.

(3) To enable the vehicle to be entered, the authorised officer may—

(a) act with necessary and reasonable help and force; and

(b) require the person in control of the vehicle to give reasonable help to the officer.

(4) A person must obey a signal under subsection (2), unless the person has a reasonable excuse for not obeying it.

Maximum penalty—20 penalty units.

(5) A person must comply with a requirement under subsection (3)(b), unless the person has a reasonable excuse for not complying with it.

Maximum penalty—20 penalty units.

(6) It is a reasonable excuse for a person to disobey a signal under subsection (2) if—

(a) the person reasonably believes that to obey the signal immediately would have endangered the person or someone else, or the vehicle; and

(b) the person obeys the signal as soon as it is practicable to obey it.

86 General powers in relation to places and vehicles

(1) An authorised officer who enters a place or vehicle under this part may—

Authorised by the Parliamentary Counsel

(a) search any part of the place or vehicle; or

(b) examine, inspect, test, photograph or film anything in or on the place or vehicle; or

(c) take samples of or from anything in or on the place or vehicle; or

(d) take extracts from, or make copies of, a document in or on the place or vehicle; or

(e) take into or onto the place or vehicle any persons, equipment and materials the authorised officer reasonably requires for exercising a power under this Act; or

(f) require the occupier of the place, or a person in or on the place or vehicle, to give the authorised officer reasonable help to exercise the powers mentioned in paragraphs (a) to (e); or

(g) if the authorised officer enters a vehicle—by written notice given to the person in control of the vehicle, require the person—

(i) to take the vehicle to a stated reasonable place by a stated reasonable time; and

(ii) if necessary, to remain in control of the vehicle at the place for a reasonable time;

to enable the officer to exercise the powers mentioned in paragraphs (a) to (e); or

(h) if the authorised officer enters a vehicle—require the person in charge of the vehicle to accompany the authorised officer to enable the authorised officer to comply with subsection (8).

(2) If the entry is made to a place other than under a warrant, the authorised officer may exercise the power mentioned in subsection (1)(a) only if the occupier consents to the exercise of the power.

(3) A person who is required by an authorised officer under subsection (1)(f) to give the officer reasonable help for the

Authorised by the Parliamentary Counsel

exercise of a power must comply with the requirement, unless the person has a reasonable excuse for not complying with it.

Maximum penalty—20 penalty units.

(4) If the help is required to be given to a person by—

(a) answering a question; or

(b) producing a document (other than a licence or permit);

it is a reasonable excuse for the person to fail to answer the question, or produce the document, if complying with the requirement might tend to incriminate the person.

(5) A person who is required by an authorised officer under subsection (1)(g) to take action in relation to a vehicle must comply with the requirement, unless the person has a reasonable excuse for not complying with it.

Maximum penalty—20 penalty units.

(6) If, for any reason, it is not practicable to make a requirement under subsection (1)(g) by written notice, the requirement may be made orally and confirmed by written notice as soon as practicable.

(7) Nothing in this section prevents an authorised officer making a further requirement under subsection (1)(g) of the same person or another person in relation to the same vehicle, if it is necessary and reasonable to make the requirement.

(8) The authorised officer must not enter a part of a vehicle used only as a living area, or exercise a power under subsection (1)(a) to (d) in relation to that part, unless the authorised officer is accompanied by the person in control of the vehicle.

(9) Subsection (8) does not apply if the person in control of the vehicle is unavailable or unwilling to accompany the authorised officer or the authorised officer is unable for another reason to comply with the subsection.

(10) This section does not apply to an authorised officer who enters a place to get the occupier's consent unless the consent is given or the entry is otherwise authorised.

Authorised by the Parliamentary Counsel

87 Power to seize evidence

(1) An authorised officer who enters a place under this part under a warrant may seize the evidence for which the warrant was issued.

(2) An authorised officer who enters a place under this part with the occupier's consent may seize the particular thing for which the entry was made if the officer believes on reasonable grounds that the thing is evidence of an offence against this Act.

(3) An authorised officer who enters a place under this part under a warrant, or with the occupier's consent, may also seize anything else if the officer believes on reasonable grounds—

(a) the thing is evidence of the commission of an offence against this Act; and

(b) the seizure is necessary to prevent—

(i) the concealment, loss or destruction of the thing; or

(ii) the use of the thing in committing, continuing or repeating the offence.

(4) An authorised officer who enters a place under this part other than under a warrant or with the occupier's consent, may seize a thing if the officer believes on reasonable grounds—

(a) the thing is evidence of the commission of an offence against this Act; and

(b) the seizure is necessary to prevent—

(i) the concealment, loss or destruction of the thing; or

(ii) the use of the thing in committing, continuing or repeating the offence.

88 Power to seize evidence after entering a vehicle

An authorised officer who enters a vehicle under a warrant under this part may seize—

(a) a thing in or on the vehicle; or

Authorised by the Parliamentary Counsel

(b) the vehicle itself;

if the authorised officer believes, on reasonable grounds, the thing or vehicle is evidence of the commission of an offence against 1 of the following provisions—

(c) for a vehicle—

- section 36
- section 37
- section 38
- section 55; or

(d) for a thing—

- a provision mentioned in paragraph (c)
- section 91(3).

89 Procedure after thing seized

(1) As soon as possible after a thing is seized by an authorised officer under this part, the authorised officer must give a receipt for it to the person from whom it was seized.

(2) The receipt must describe generally each thing seized and its condition.

(3) If, for any reason, it is not practicable to comply with subsection (1), the authorised officer must—

(a) leave the receipt where the thing was seized; and

(b) ensure the receipt is left in a reasonably secure way and in a conspicuous position.

(4) The authorised officer must return the thing seized to the person at the end of—

(a) 6 months; or

(b) if a prosecution for an offence involving the thing is started within the 6 months—the prosecution for the offence and any appeal from the prosecution.

Authorised by the Parliamentary Counsel

(5) Despite subsection (4), the authorised officer must return the seized thing to the person immediately the authorised officer stops being satisfied its retention is necessary.

(6) However, the authorised officer may keep the seized thing if the authorised officer believes, on reasonable grounds, that its continued retention is necessary to prevent its use in committing an offence against this Act.

90 Power to require name and address

(1) An authorised officer may require a person to state the person's name and address if the officer—

(a) finds the person committing an offence against this Act; or

(b) finds the person in circumstances that lead, or has information that leads, the authorised officer to suspect on reasonable grounds that the person just committed an offence against this Act; or

(c) has information that leads the authorised officer to suspect, on reasonable grounds, that a person has just committed an offence against this Act.

(2) When making the requirement, the authorised officer must warn the person it is an offence to fail to state the person's name and address, unless the person has a reasonable excuse.

(3) The authorised officer may require the person to give evidence of the correctness of the person's name or address if the officer suspects, on reasonable grounds, that the stated name or address is false.

(4) A person must comply with a requirement under subsection (1) or (3), unless the person has a reasonable excuse for not complying with it.

Maximum penalty—20 penalty units.

(5) The person does not commit an offence against this section if—

Authorised by the Parliamentary Counsel

(a) the authorised officer required the person to state the person's name and address on suspicion of the person having committed an offence against this Act; and

(b) the person is not proved to have committed the offence.

91 Power to seize weapons etc.

(1) This section applies if a person brings a weapon or explosive onto designated fossicking land or a fossicking area.

(2) An authorised officer may require a person using, about to use, or in possession of a weapon or explosive on designated fossicking land or a fossicking area—

(a) to remove the weapon or explosive from the land or area; or

(b) to surrender the weapon or explosive to the officer.

(3) A person must not contravene a requirement under subsection (2), unless the person has a reasonable excuse for not complying with it.

Maximum penalty—20 penalty units.

(4) If the person surrenders the weapon or explosive to the authorised officer, the authorised officer must—

(a) notify a police officer of the surrender to the authorised officer of the weapon or explosive; and

(b) either—

(i) keep the weapon or explosive until the owner of the weapon or explosive leaves the land or area; or

(ii) if required by the police officer, give it to the police officer to be dealt with under the *Police Powers and Responsibilities Act 2000*.

(5) An authorised officer does not, by merely keeping a weapon or explosive under this section, contravene the *Weapons Act 1990*.

Authorised by the Parliamentary Counsel

Division 3 Offences

92 Obstruction of authorised officer

A person must not obstruct an authorised officer, or a person helping an authorised officer, in the exercise of a power, unless the person has a reasonable excuse.

Maximum penalty—50 penalty units.

93 Re-entering land after direction to leave

A person given a direction under section 78 to leave land where the person is fossicking must not re-enter the land—

(a) within the period stated in the direction; and

(b) after the end of the period without the owner's permission;

unless the person has a reasonable excuse for the re-entry.

Maximum penalty—20 penalty units.

94 Proceedings for offences

A proceeding for an offence against this Act may be instituted in a summary way under the *Justices Act 1886*.

Division 4 Proceedings

95 Forfeiture on conviction

(1) On the conviction of a person for an offence against this Act, the court convicting the person may order the forfeiture to the State of any of the following—

(a) anything used to commit the offence;

(b) anything else the subject of the offence.

Authorised by the Parliamentary Counsel

(2) Also, the court may order the retention of the thing by an authorised officer for a stated time.

(3) The court may make any order to enforce the forfeiture or retention that it considers appropriate.

(4) This section does not limit the court's powers under the *Penalties and Sentences Act 1992* or any other law.

96 Dealing with forfeited things

(1) On the forfeiture of a thing, the thing becomes the State's property and may be dealt with by the State as it considers appropriate.

(2) Without limiting subsection (1), the State may—

(a) sell it to its previous owner or a person who had a legal or beneficial interest in it; or

(b) sell it to anyone else (by auction, tender or otherwise); or

(c) destroy it or give it away.

97 Disposal of abandoned property

(1) In this section—

abandoned property means property (including a vehicle and anything attached to, or contained in a vehicle) an authorised officer believes on reasonable grounds has been abandoned.

(2) If an authorised officer finds abandoned property on designated fossicking land or a fossicking area and intends to take action under this section, the authorised officer—

(a) must take reasonable steps to locate the owner of the property; and

(b) may move the property to a place the authorised officer considers appropriate.

(3) As soon as is practicable but within 14 days after finding the abandoned property and deciding to take action under this

Authorised by the Parliamentary Counsel

section, the authorised officer must give to the owner of the property a written notice describing the property, stating that the property has been found, explaining how it may be recovered and stating that it may be sold or disposed of if it is not recovered.

(4) If the owner of the property can not be located within the 14 days mentioned in subsection (3), the notice may be given by publishing it in a newspaper circulating generally throughout the State.

(5) Subsection (4) does not apply if the authorised officer believes, on reasonable grounds, the property has no value or a value less than an amount prescribed by regulation.

(6) If a person claims the abandoned property within 1 month after the notice is given, the authorised officer must return the property to the person if the person—

(a) satisfies the authorised officer that the person is the owner of the property; and

(b) pays the expenses reasonably incurred by the authorised officer in dealing with the property.

(7) If a person does not claim the abandoned property within 1 month after the notice is given, the chief executive may—

(a) sell the property; or

(b) dispose of the property in the way the chief executive considers appropriate if the proceeds of sale of the property are not likely to cover the total of—

(i) the expenses reasonably incurred by the chief executive in selling the property; and

(ii) the expenses reasonably incurred by the authorised officer in dealing with the property under this section; and

(iii) any other expenses owing to the chief executive in relation to the property.

(8) If the abandoned property is sold, the proceeds of the sale must be applied in the following order—

Authorised by the Parliamentary Counsel

(a) in payment of the expenses reasonably incurred by the chief executive in selling the property;

(b) in payment of the expenses reasonably incurred by the authorised officer in dealing with the property under this section;

(c) if the removal of the property caused damage to the land where it was found—in payment of the reasonable cost of rectifying the damage, including by rehabilitating the land;

(d) in payment of any other expenses owing to the chief executive in relation to the property;

(e) in payment of any balance to the owner of the property.

(9) Despite anything else in this section, if the abandoned property has no value or insufficient value to justify its sale, the chief executive may dispose of the property in the way the chief executive considers appropriate.

(10) Compensation is not recoverable against the chief executive for a payment under this section.

98 Evidentiary provision

(1) This section applies to a proceeding under this Act.

(2) The appointment or power of an authorised officer must be presumed unless a party, by reasonable notice, requires proof of the appointment or the power of the authorised officer to do anything under this Act.

(3) A signature purporting to be the signature of any of the following persons is evidence of the signature it purports to be—

(a) the chief executive;

(b) an authorised officer;

(c) the chief executive of the department in which the *Land Act 1962* is administered;

Authorised by the Parliamentary Counsel

(d) the person holding office in the department as chief government geologist.

(4) A certificate, purporting to be signed by the chief executive of the department in which the *Land Act 1962* is administered, stating that specified land is occupied land is evidence of the things stated.

(5) A certificate purporting to be signed by a person mentioned in subsection (3)(d) and stating a substance is a mineral, gemstone, ornamental stone or fossil is evidence of the thing stated.

(6) A certificate purporting to be signed by the chief executive or an authorised officer and stating any of the following matters is evidence of the matter—

(a) a stated document is a copy of—

(i) a licence or permit or a copy of a licence or permit; or

(ii) a direction, requirement or decision or a copy of a direction, requirement or decision given or made under this Act; or

(iii) a notice, or a copy of a notice, given under this Act; or

(iv) a record, or a copy of a record, kept under this Act; or

(v) a document, or a copy of a document, kept under this Act;

(b) on a stated day, or during a stated period, a stated person was or was not the holder of a licence or permit;

(c) a stated licence or permit was or was not in force on a stated day or during a stated period;

(d) on a stated day—

(i) a licence was suspended for a stated period; or

(ii) a licence or permit was cancelled;

(e) on a stated day, a stated person was given a stated notice requirement or direction under this Act;

(f) anything else prescribed by regulation.

Part 7 Appeals

99 Appeals to Land Court

(1) An applicant for a licence or permit may appeal to the Land Court against an issuing officer's decision to refuse to grant the licence or permit.

(2) A licensee may appeal against—

(a) a decision of an issuing officer—

(i) to impose a condition on a licence; or

(ii) to refuse to replace a licence; or

(b) a decision of the chief executive to suspend or cancel a licence; or

(c) a decision of an authorised officer resulting in the cancellation of a person's permit under section 78(5).

100 Starting appeal

(1) An appeal is started by filing a written notice of appeal with the chief executive.

(2) The notice of appeal must be filed within 28 days after the appellant receives notice of the decision appealed against.

(3) The Land Court may at any time extend the period for filing the notice of the appeal.

(4) The notice of appeal must state the grounds of the appeal.

Authorised by the Parliamentary Counsel

101 Stay of operation of decisions etc.

(1) The Land Court may stay a decision appealed against to secure the effectiveness of the appeal.

(2) A stay—

(a) may be given on conditions the Land Court considers appropriate; and

(b) operates for the period specified by the Land Court; and

(c) may be revoked or amended by the Land Court.

(3) The period of a stay specified by the Land Court must not extend past the time when the Land Court decides the appeal.

(4) The starting of an appeal against a decision affects the decision, or the carrying out of the decision, only if the decision is stayed.

102 Hearing procedures

(1) An appeal is to be by way of rehearing unaffected by the decision of the authorised officer, issuing officer or chief executive.

(2) In deciding an appeal, the Land Court—

(a) is not bound by the rules of evidence; and

(b) must observe natural justice.

103 Powers of Land Court on appeal

(1) In deciding an appeal, the Land Court may—

(a) confirm the decision appealed against; or

(b) set aside the decision and substitute another decision; or

(c) set aside the decision and return the decision to the authorised officer, issuing officer or the chief executive with directions the court considers appropriate.

Authorised by the Parliamentary Counsel

(2) In substituting another decision, the Land Court has the same powers as an authorised officer, issuing officer or the chief executive.

Example—

The Land Court may decide an unsuccessful applicant for a licence be granted the licence either unconditionally or on particular conditions.

(3) If the Land Court substitutes another decision, the substituted decision is taken, for the purposes of this Act, to be the decision of the authorised officer, issuing officer or the chief executive.

Part 8 General

104 Records to be kept by registrar

(1) Within 14 days after an agreement mentioned in section 48 for the declaration of land as designated fossicking land or a fossicking area is entered into, the chief executive must give written notice of the agreement to the registrar for the land.

(2) The registrar must keep records that—

(a) show the land subject to an agreement mentioned in section 48; and

(b) state where particulars of the agreement may be inspected.

(3) The registrar must keep the records in a way that ensures a search of a register kept by the registrar under any Act about the land will show the existence of the agreement.

(4) The chief executive must, within 14 days after an agreement is ended or land is excluded from the operation of the agreement, give written notice to the registrar that the agreement has ended or the land has been excluded from its operation.

(5) On receiving the notice, the registrar must—

Authorised by the Parliamentary Counsel

(a) remove the particulars of the agreement from the registrar's records; or

(b) if the agreement has ended in relation to some, but not all the land—change the record to show the land still subject to the agreement.

105 Delegation

The chief executive may delegate the chief executive's powers under this Act to an officer of the public service.

106 Protection against liability

(1) This section applies to the following persons—

(a) the chief executive;

(b) an authorised officer;

(c) an issuing officer;

(d) a land manager;

(e) an owner of designated fossicking land or land in a fossicking area;

(f) a person who has given someone else permission under this Act to fossick or camp on the person's land, mining claim or mining lease.

(2) A person to whom this section applies does not incur civil liability for an act done, or omission made, honestly and without negligence under this Act.

(3) If subsection (2) prevents a civil liability attaching to the person, the liability attaches instead to the State.

107 Regulation-making power

(1) The Governor in Council may make regulations under this Act.

(2) A regulation may be made about the following matters—

Authorised by the Parliamentary Counsel

(a) the conduct of fossickers;

(b) the conduct of persons other than owners or fossickers on designated fossicking land and fossicking areas;

(c) controlling pollution on designated fossicking land and fossicking areas;

(d) naming, and defining the boundaries of, a miners common;

(e) the appointment of a controller of a miners common;

(f) the powers and functions of a controller of a miners common;

(g) the management of a miners common, including the appointment of staff;

(h) fees to be paid under the Act.

(3) A regulation may provide that a contravention of a regulation is an offence and prescribe a maximum penalty for the offence of not more than 80 penalty units.

Part 9 Repeal and transitional provisions before Mining and Other Legislation Amendment Act 2013

119 References to Mining (Fossicking) Act 1985

(1) This section applies to references in Acts and documents in existence on its commencement.

(2) A reference to the *Mining (Fossicking) Act 1985* is taken to be a reference to this Act.

120 Expiry

Sections 109 and 111 to 118 expire 1 year after they commence.

Authorised by the Parliamentary Counsel

Part 10 Transitional provisions for Mining and Other Legislation Amendment Act 2013

121 Definitions for pt 10

In this part—

commencement means the day this part commences.

mining registrar means a mining registrar under the *Mineral Resources Act 1989* as in force before the commencement.

122 Continuing effect of general permissions

(1) A general permission in effect immediately before the commencement continues as a general permission under this Act after the commencement.

(2) The general permission is subject to the same conditions as those in effect for the permission immediately before the commencement.

123 Continuing effect of permissions under s 56

(1) A permission of a mining registrar given under section 56 and in effect immediately before the commencement continues as if it were a permission given by the chief executive under section 56 as in force after the commencement.

(2) The permission is subject to the same conditions as those in effect for the permission immediately before the commencement.

124 Appeals

(1) Subsection (2) applies if—

(a) a person has appealed to the Land Court against a decision of a mining registrar before the commencement; and

Authorised by the Parliamentary Counsel

(b) the appeal has not been decided before the commencement.

(2) The Land Court must hear, or continue to hear, and decide the appeal under this Act as in force after the commencement.

(3) Subsection (4) applies if—

(a) immediately before the commencement, a person could have appealed to the Land Court against a decision of a mining registrar; and

(b) the person has not appealed before the commencement.

(4) The person may appeal and the Land Court must hear and decide the appeal under this Act as in force after the commencement.

(5) For hearing and deciding an appeal under subsection (2) or (4), the decision appealed against is taken to be a decision of the chief executive.

Authorised by the Parliamentary Counsel
