# Supplementary material for: Cranial anatomy, palaeoneurology, palaeobiology and stratigraphic age of the large-bodied ornithopod, Muttaburrasaurus langdoni Bartholomai and Molnar, 1981, from the mid-Cretaceous of Australia
Source: PeerJ. 2026 Apr 9;14:e20794. doi: 10.7717/peerj.20794 (PMC13070326; doi:10.7717/peerj.20794)
Supplement: Supplemental Information 9 [file peerj-14-20794-s009.pdf]

CRANIAL ANATOMY, PALAEONEUROLOGY, PALAEOBIOLOGY AND STRATIGRAPHIC AGE OF THE LARGE-BODIED ORNITHOPOD, *MUTTABURRASAURUS LANGDONI* BARTHOLOMAI AND MOLNAR, 1981, FROM THE MID-CRETACEOUS OF AUSTRALIA

Matthew C. Herne, Joseph J. Bevitt, Luke Milan, Scott A. Hocknull, Alan M. Tait, Charlotte Allen, Andrew Rozefelds, Ralph Molnar, Vera Weisbecker and Phil Bell

SUPPLEMENTAL DATA F2: LINES-OF-SIGHT

**Angle from horizon (A = anterior; P = posterior)**	**Angle from sagittal plane**
P0	+14
P+15	+17
P+30	+19
P+45	+28
P+50	+21
P+75	+20
+90	+21
P-15	+14
P-30	+14
P-45	+15
P-75	+16
-90	+17
A0	-16
A+3	-17
A+16	-16
A+22.5	-5

A+50	+20
A+75	+21
A-10	-15
A-17	-17
A-34	-16
A-72	+18
