# Supplementary material for: Cranial anatomy, palaeoneurology, palaeobiology and stratigraphic age of the large-bodied ornithopod, Muttaburrasaurus langdoni Bartholomai and Molnar, 1981, from the mid-Cretaceous of Australia
Source: PeerJ. 2026 Apr 9;14:e20794. doi: 10.7717/peerj.20794 (PMC13070326; doi:10.7717/peerj.20794)
Supplement: Supplemental Information 10 [file peerj-14-20794-s010.docx]

CRANIAL ANATOMY, PALAEONEUROLOGY, PALAEOBIOLOGY AND STRATIGRAPHIC

AGE OF THE LARGE-BODIED ORNITHOPOD, *MUTTABURRASAURUS LANGDONI*

BARTHOLOMAI AND MOLNAR, 1981, FROM THE MID-CRETACEOUS OF AUSTRALIA

Matthew C. Herne, Joseph J. Bevitt, Luke Milan, Scott A. Hocknull, Alan M. Tait, Charlotte

Allen, Andrew Rozefelds, Ralph Molnar, Vera Weisbecker and Phil Bell

SUPPLEMENTAL DATA: MORPHOSOURCE LINKS

| # | Item | Type | Cranial Part number | DOI |
| --- | --- | --- | --- | --- |
| 1 | DINOSAUR_0_5_BONE_THINS_B_SN140KV_0004 | CT scan | 1, 2 | 10.17602/M2/M788508 |
| 2 | CT scan: DE 140KVA; Large Abdomen | CT scan | 1, 2 | 10.17602/M2/M787635 |
| 3 | Cranium photogrammetry | Mesh | 1, 2 | 10.17602/M2/M786923 |
| 4 | Right premaxilla dental ramus | Mesh | 14 | 10.17602/M2/M786841 |
| 5 | Right premaxilla germ teeth | Mesh | 14 | 10.17602/M2/M786844 |
| 6 | Right premaxilla functional teeth | Mesh | 14 | 10.17602/M2/M786847 |
| 7 | Left premaxilla dental ramus | Mesh | 6,7,12 | 10.17602/M2/M786859 |
| 8 | Left premaxilla germ teeth | Mesh | 6,7,12 | 10.17602/M2/M786862 |
| 9 | Left premaxilla functional teeth | Mesh | 6,7,12 | 10.17602/M2/M786887 |
| 10 | Left premaxilla dental ramus | Mesh | 13 | 10.17602/M2/M786850 |
| 11 | Left premaxilla germ teeth | Mesh | 13 | 10.17602/M2/M786853 |
| 12 | Left premaxilla functional teeth | Mesh | 13 | 10.17602/M2/M786856 |
| 13 | Left premaxilla posterodorsal process | Mesh | 2 | 10.17602/M2/M787709 |
| 14 | Left premaxilla posteroventral process | Mesh | 2 | 10.17602/M2/M787712 |
| 15 | Right maxilla dental ramus | Mesh | 8 | 10.17602/M2/M786911 |
| 16 | Right maxilla germ teeth | Mesh | 8 | 10.17602/M2/M786915 |
| 17 | Right maxilla functional teeth | Mesh | 8 | 10.17602/M2/M786919 |
| 18 | Right maxilla dental ramus | Mesh | 9 | 10.17602/M2/M771412 |
| 19 | Right maxilla germ teeth | Mesh | 9 | 10.17602/M2/M786868 |
| 20 | Right maxilla functional teeth | Mesh | 9 | 10.17602/M2/M786871 |
| 21 | Left maxilla (anterior half) dental ramus | Mesh | 2 | 10.17602/M2/M787646 |
| 22 | Left maxilla (anterior half) germ teeth | Mesh | 2 | 10.17602/M2/M787655 |
| 23 | Left maxilla (anterior half) functional teeth | Mesh | 2 | 10.17602/M2/M787661 |
| 24 | Left maxilla (posterior half) dental ramus | Mesh | 1 | 10.17602/M2/M787649 |
| 25 | Left maxilla (posterior half) germ teeth | Mesh | 1 | 10.17602/M2/M787652 |
| 26 | Left maxilla (posterior half) functional teeth | Mesh | 1 | 10.17602/M2/M787658 |
| 27 | Left prenasal | Mesh | 2 | 10.17602/M2/M787694 |
| 28 | Right prenasal | Mesh | 2 | 10.17602/M2/M787691 |
| 29 | Prenasal septa | Mesh | 2 | 10.17602/M2/M787697 |
| 30 | Prenasal septa additional | Mesh | 2 | 10.17602/M2/M787700 |
| 31 | Left jugal | Mesh | 1 | 10.17602/M2/M787715 |
| 32 | Left lacrimal (anterior half) | Mesh | 2 | 10.17602/M2/M787721 |
| 33 | Left lacrimal (posterior half) | Mesh | 1 | 10.17602/M2/M787718 |
| 34 | Left lacrimal (complete) | Mesh | 1, 2 | 10.17602/M2/M787734 |
| 35 | Left quadratojugal | Mesh | 1 | 10.17602/M2/M787743 |
| 36 | Left quadrate | Mesh | 1 | 10.17602/M2/M788110 |
| 37 | Left nasal | Mesh | 1, 2 | 10.17602/M2/M787703 |
| 38 | Right nasal | Mesh | 1 | 10.17602/M2/M787706 |
| 39 | Left prefrontal | Mesh | 1 | 10.17602/M2/M788113 |
| 40 | Left frontal | Mesh | 1 | 10.17602/M2/M788116 |
| 41 | Right frontal | Mesh | 1 | 10.17602/M2/M788121 |
| 42 | Left postorbital | Mesh | 1 | 10.17602/M2/M788124 |
| 43 | Left intrapostorbital | Mesh | 1 | 10.17602/M2/M788131 |
| 44 | Left pterygoid | Mesh | 1 | 10.17602/M2/M787756 |
| 45 | Right pterygoid | Mesh | 1, 2 | 10.17602/M2/M787749 |
| 46 | Vomers | Mesh | 1 | 10.17602/M2/M787746 |
| 47 | Left palatine | Mesh | 1 | 10.17602/M2/M790005 |
| 48 | Left ectopterygoid | Mesh | 1 | 10.17602/M2/M790002 |
| 49 | Basioccipital | Mesh | 1 | 10.17602/M2/M788140 |
| 50 | Parabasisphenoid | Mesh | 1 | 10.17602/M2/M788143 |
| 51 | Orbitosphenoid | Mesh | 1 | 10.17602/M2/M788146 |
| 52 | Left laterosphenoid | Mesh | 1 | 10.17602/M2/M789996 |
| 53 | Right laterosphenoid | Mesh | 1 | 10.17602/M2/M789999 |
| 54 | Left prootic | Mesh | 1 | 10.17602/M2/M788155 |
| 55 | Right prootic | Mesh | 1 | 10.17602/M2/M788158 |
| 56 | Left otoccipital | Mesh | 1 | 10.17602/M2/M788152 |
| 57 | Right otoccipital | Mesh | 1 | 10.17602/M2/M788149 |
| 58 | Supraoccipital | Mesh | 1 | 10.17602/M2/M788137 |
| 59 | Left / right squamosals | Mesh | 1 | 10.17602/M2/M790008 |
| 60 | Parietals | Mesh | 1 | 10.17602/M2/M788134 |
| 61 | Left dentary (anterior half) ramus | Mesh | 3,5,10 | 10.17602/M2/M786907 |
| 62 | Left dentary (anterior half) germ teeth | Mesh | 3,5,10 | 10.17602/M2/M786883 |
| 63 | Left dentary (anterior half) functional teeth | Mesh | 3,5,10 | 10.17602/M2/M786887 |
| 64 | Left dentary (posterior half) ramus | Mesh | 1 | 10.17602/M2/M787664 |
| 65 | Left dentary (posterior half) germ teeth | Mesh | 1 | 10.17602/M2/M787664 |
| 66 | Left dentary (posterior half) functional teeth | Mesh | 1 | 10.17602/M2/M787670 |
| 67 | Right dentary (anterior half) ramus | Mesh | 11 | 10.17602/M2/M786891 |
| 68 | Right dentary (anterior half) germ teeth | Mesh | 11 | 10.17602/M2/M786899 |
| 69 | Right dentary (anterior half) functional teeth | Mesh | 11 | 10.17602/M2/M786903 |
| 70 | Right dentary (anterior half) dental parapet | Mesh | 11 | 10.17602/M2/M786895 |
| 71 | Left surangular | Mesh | 1 | 10.17602/M2/M787673 |
| 72 | Left angular | Mesh | 1 | 10.17602/M2/M787676 |
| 73 | Left coronoid | Mesh | 1 | 10.17602/M2/M787685 |
| 74 | Left prearticular | Mesh | 1 | 10.17602/M2/M787679 |
| 75 | Left articular | Mesh | 1 | 10.17602/M2/M787682 |
| 76 | Left ceratobranchial | Mesh | 1 | 10.17602/M2/M787688 |
| 77 | Neural endocranium | Mesh | 1 | 10.17602/M2/M788215 |
| 78 | Cranium CT | Mesh | 1,2 | 10.17602/M2/M788197 |
